# Integrating ChatGPT in Orthopedic Education for Medical Undergraduates: Randomized Controlled Trial

**DOI:** 10.2196/57037

**Published:** 2024-08-20

**Authors:** Wenyi Gan, Jianfeng Ouyang, Hua Li, Zhaowen Xue, Yiming Zhang, Qiu Dong, Jiadong Huang, Xiaofei Zheng, Yiyi Zhang

**Affiliations:** 1 The First Clinical Medical College of Jinan University, The First Affiliated Hospital of Jinan University Guangzhou China; 2 Department of Joint Surgery and Sports Medicine, Zhuhai People's Hospital (Zhuhai Hospital Affiliated With Jinan University) Zhuhai, Guangdong China; 3 Department of Orthopaedics, Beijing Jishuitan Hospital Beijing China; 4 Jinan University-University of Birmingham Joint Institute, Jinan University Guangzhou China

**Keywords:** ChatGPT, medical education, orthopedics, artificial intelligence, large language model, natural language processing, randomized controlled trial, learning aid

## Abstract

**Background:**

ChatGPT is a natural language processing model developed by OpenAI, which can be iteratively updated and optimized to accommodate the changing and complex requirements of human verbal communication.

**Objective:**

The study aimed to evaluate ChatGPT’s accuracy in answering orthopedics-related multiple-choice questions (MCQs) and assess its short-term effects as a learning aid through a randomized controlled trial. In addition, long-term effects on student performance in other subjects were measured using final examination results.

**Methods:**

We first evaluated ChatGPT’s accuracy in answering MCQs pertaining to orthopedics across various question formats. Then, 129 undergraduate medical students participated in a randomized controlled study in which the ChatGPT group used ChatGPT as a learning tool, while the control group was prohibited from using artificial intelligence software to support learning. Following a 2-week intervention, the 2 groups’ understanding of orthopedics was assessed by an orthopedics test, and variations in the 2 groups’ performance in other disciplines were noted through a follow-up at the end of the semester.

**Results:**

ChatGPT-4.0 answered 1051 orthopedics-related MCQs with a 70.60% (742/1051) accuracy rate, including 71.8% (237/330) accuracy for A1 MCQs, 73.7% (330/448) accuracy for A2 MCQs, 70.2% (92/131) accuracy for A3/4 MCQs, and 58.5% (83/142) accuracy for case analysis MCQs. As of April 7, 2023, a total of 129 individuals participated in the experiment. However, 19 individuals withdrew from the experiment at various phases; thus, as of July 1, 2023, a total of 110 individuals accomplished the trial and completed all follow-up work. After we intervened in the learning style of the students in the short term, the ChatGPT group answered more questions correctly than the control group (ChatGPT group: mean 141.20, SD 26.68; control group: mean 130.80, SD 25.56; *P*=.04) in the orthopedics test, particularly on A1 (ChatGPT group: mean 46.57, SD 8.52; control group: mean 42.18, SD 9.43; *P*=.01), A2 (ChatGPT group: mean 60.59, SD 10.58; control group: mean 56.66, SD 9.91; *P*=.047), and A3/4 MCQs (ChatGPT group: mean 19.57, SD 5.48; control group: mean 16.46, SD 4.58; *P*=.002). At the end of the semester, we found that the ChatGPT group performed better on final examinations in surgery (ChatGPT group: mean 76.54, SD 9.79; control group: mean 72.54, SD 8.11; *P*=.02) and obstetrics and gynecology (ChatGPT group: mean 75.98, SD 8.94; control group: mean 72.54, SD 8.66; *P*=.04) than the control group.

**Conclusions:**

ChatGPT answers orthopedics-related MCQs accurately, and students using it excel in both short-term and long-term assessments. Our findings strongly support ChatGPT’s integration into medical education, enhancing contemporary instructional methods.

**Trial Registration:**

Chinese Clinical Trial Registry Chictr2300071774; https://www.chictr.org.cn/hvshowproject.html ?id=225740&v=1.0

## Introduction

ChatGPT, a natural language processing model developed by OpenAI, is based on a sophisticated machine learning algorithm that can be iteratively updated and optimized to accommodate the changing and complex requirements of human verbal communication [1-3]. ChatGPT-4.0 is significantly superior to ChatGPT-3.5 in terms of language comprehension, context comprehension, generation speed, and interpretability [4]. It can be applied to a variety of natural language processing duties and provides individuals with more precise, efficient, and intelligent natural language processing services [5,6]. Numerous researchers have reported that ChatGPT can achieve satisfactory results on multidisciplinary medical practitioner examinations in a variety of countries as well as provide detailed evidence-based explanations when responding to input clinical scenarios [7-10]. These studies have investigated the function of ChatGPT in the vast field of medicine and demonstrated the feasibility of investigating the application of ChatGPT in medical education.

Due to the vast medical knowledge system, diverse content, and lengthy learning cycle, it is difficult to accomplish a quality breakthrough in medical education [11,12]. Both general practitioners and specialists must keep relearning medical knowledge to avoid forgetting some minor but essential knowledge points during clinical practice [13,14]. The internet has become a common learning resource for physicians and medical students due to its convenience. However, the internet’s general search results are vast and complex, necessitating the use of very specific search terms so that users can find the answers they seek [15]. Designing a specific learning application based on the network can increase the output of knowledge points, but it may not be able to respond promptly to the personalized user query input [16]. By identifying keywords in queries and analyzing their relevance, search engines provide users with a variety of search results; however, users must frequently determine the authenticity and veracity of the answers [17,18]. It has been indicated that while ChatGPT and professional forum answers exhibit comparable levels of logic and accuracy in their specific responses, ChatGPT demonstrates significantly greater empathy than the answers provided by verified individuals on forums, despite the fact that ChatGPT relies on big data [8,19]. Therefore, ChatGPT’s response is more akin to a web-based answer retrieval, with the veracity of the retrieved answer being filtered based on the user’s personalized query and the expression being intensively processed prior to outputting the result.

Although many academics believe that it is a double-edged sword to use ChatGPT as an auxiliary instrument in medical education [20-22], the development and promotion of ChatGPT will undoubtedly fuel the innovation of medical education [23,24]. The major reason why academics are worried about ChatGPT’s inadequacies in medical education is that its potent text creation feature brings the risk of making students too reliant on it as a writing tool [25,26]. However, through interactive learning and immediate feedback, ChatGPT as a virtual teaching assistant can increase learning efficiency [23]. Artificial intelligence (AI)–assisted learning breaks the mode of 1-way input of theoretical knowledge in the previous stage of basic medical knowledge education, and its ability to generate different clinical scenarios according to different diseases can assist medical students in constructing a bridge between professional medical theory and clinical practice [27-29].

Therefore, we conducted a prospective randomized controlled trial concerning the application of ChatGPT in learning orthopedics. We first validated ChatGPT-4.0’s accuracy in answering multiple-choice questions (MCQs) related to orthopedics and then used it as a learning aid intervention and conducted short- and long-term follow-up. Through the results of the randomized controlled trial and short-term follow-up, the ultimate objective was to determine whether ChatGPT can be used as an effective learning tool for undergraduate medical students.

## Methods

### Study Design

**Figure 1:**
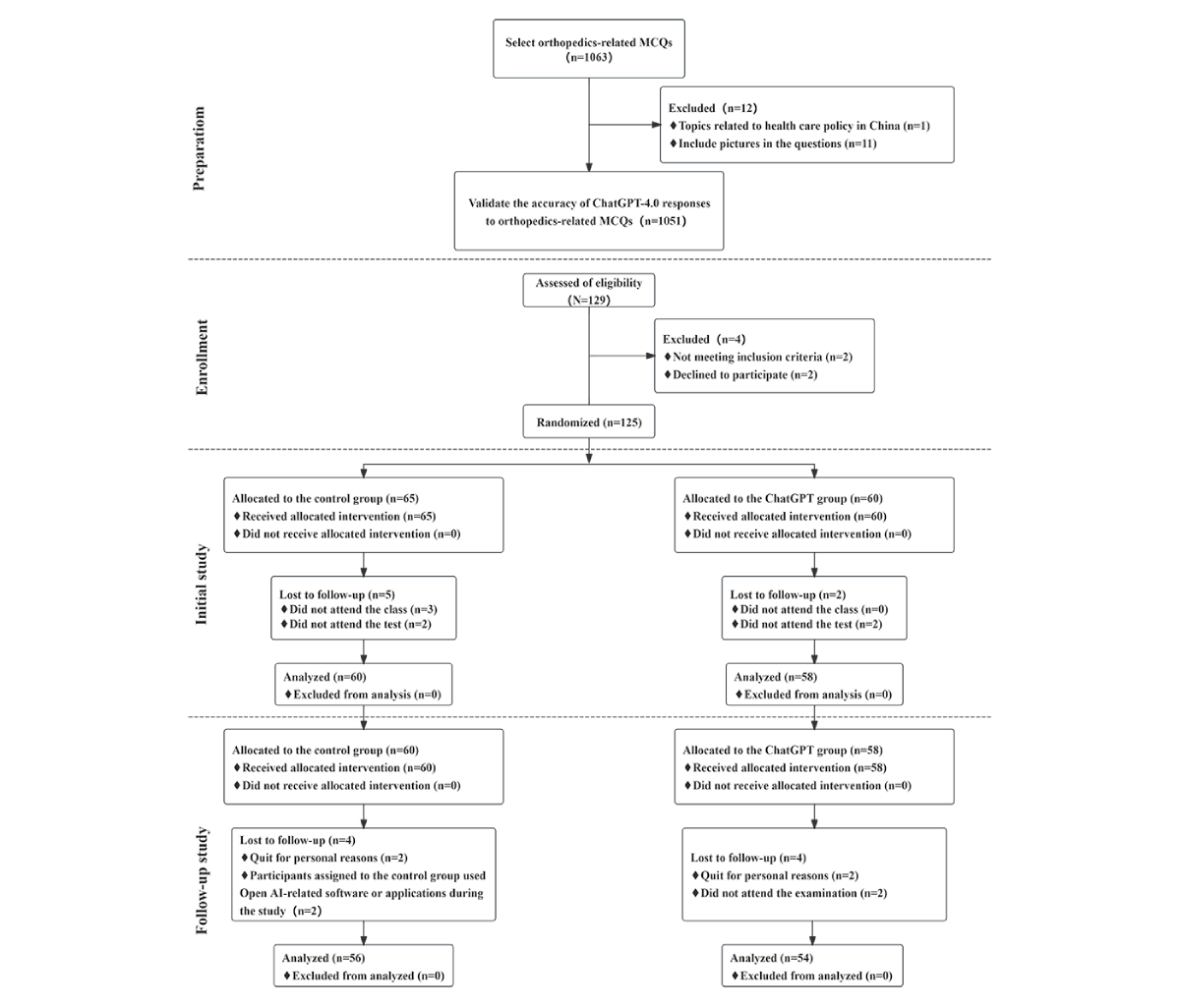
CONSORT (Consolidated Standards of Reporting Trials) 2010 flow diagram showing the randomized controlled trial process. MCQs: multiple-choice questions.

A parallel-design randomized controlled trial was used for this investigation. First, the accuracy of ChatGPT-4.0’s responses to orthopedics-related MCQs was examined. In addition, for a group experiment, 129 third-year medical students from Jinan University’s Medical College were recruited. They were divided into 2 groups at random, namely, the control group and the ChatGPT-4.0–assisted learning group (ChatGPT group). The internet-using students in the control group were not permitted to use any OpenAI-related software or programs, whereas those in the ChatGPT group used only ChatGPT-4.0 as the learning tool. Only after completing the orthopedics course, the orthopedics exercises, the review of the fundamental concepts in orthopedics, using the internet or ChatGPT, the orthopedic examination, and the final examination for the semester’s teaching task did the participants finish the experiment. The detailed process of experimental arrangement is shown in Figure 1. Both the CONSORT (Consolidated Standards of Reporting Trials) checklist (Multimedia Appendix 1) and the CONSORT-EHEALTH (Consolidated Standards of Reporting Trials of Electronic and Mobile Health Applications and online TeleHealth; version 1.6.1) checklist (Multimedia Appendix 2) were used for this trial [30,31].

### Participants

In China’s undergraduate medical education curriculum, third-year students have already completed coursework in foundational subjects such as anatomy, histology, embryology, and physiology, and are presently immersed in acquiring theoretical knowledge pertaining to clinical disciplines. Consequently, to investigate the efficacy of ChatGPT-4.0 in aiding undergraduate medical students’ learning of concepts in orthopedics, we recruited third-year undergraduate medical students from Jinan University.

We used a convenience sampling approach to recruit as many participants as possible from the target population. Recruitment was conducted through collective promotional efforts during class meetings in each individual class. This approach allowed us to efficiently disseminate information about the study and encourage widespread participation among the third-year undergraduate medical students.

### Inclusion Criteria

Participants were included if they had completed courses in human anatomy, physiology, biochemistry, pathology, pathophysiology, and diagnostics.

### Exclusion Criteria

The exclusion criteria were as follows: failing final examinations for 6 courses in human anatomy, physiology, biochemistry, pathology, pathophysiology, and diagnostics that were completed before; switching majors, stopping classes, or dropping out during the current academic year; refusing to join or leaving in the middle for private reasons; failing to finish the multiple-choice exercises or the orthopedics course; using OpenAI-related software or apps in the control group; not completing the orthopedics multiple-choice examination; and missing subjects on the semester’s final test.

### Preparation

**Figure 2:**
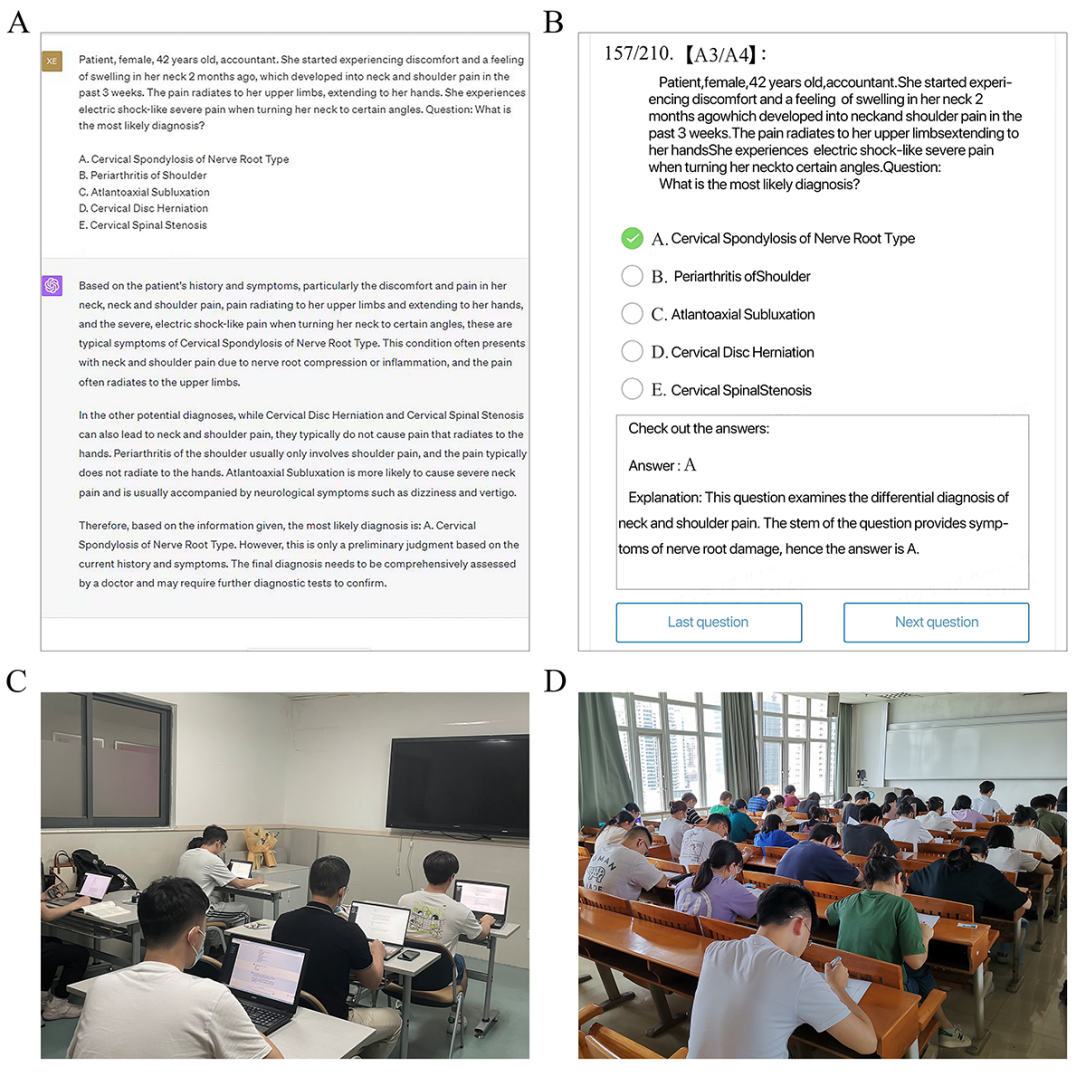
Scenarios for performing the educational clinical trial. (A) ChatGPT interface for answering orthopedics-related multiple-choice questions; (B) the “National Medical Electronic Schoolbag” is the multiple-choice practice software (English schematic diagram); (C) the participants in the ChatGPT group using ChatGPT to assist in learning orthopedics knowledge; and (D) the participants in 2 groups doing the orthopedics examination.

The “National Medical Electronic Schoolbag” (People’s Military Medical Press) is the multiple-choice practice software for the standardized training theory examination of China’s medical industry (Figure 2B). It is the first digital teaching reform project in medical higher education funded by China. After screening by 2 orthopedics specialists (WYG and ZWX), we removed 12 questions from the 1075 orthopedics-related MCQs (Multimedia Appendix 3) that were downloaded from the “National Medical Electronic Schoolbag” software (1 question was related to medical policies with Chinese characteristics, and 11 inquiries were disqualified because they contained images). A1, A2, A3/A4, and case analysis questions are the different categories of inquiries. Type A1 questions are primarily about the fundamental knowledge of orthopedics. A2 questions have a brief medical history as the topic stem. Type A3/A4 questions describe a simple patient-centered clinical situation (eg, questions 169 and 170 from the first set of questions in Multimedia Appendix 3), and case analysis questions describe a clinical situation focused on a single patient or family (eg, questions 208-217 from the first set of questions in Multimedia Appendix 3). Chinese was used as the text input language for ChatGPT in this investigation. Finally, 1051 orthopedics-related MCQs were sequentially entered into ChatGPT-4.0 (Figure 2A), and ChatGPT-4.0’s responses were recorded.

### Intervention

#### Initial Study

Participants were enrolled in a 1-week orthopedics course. Upon completion of the course, they took a multiple-choice test on orthopedics-related topics, and each participant’s accuracy rate was recorded. The control group and the ChatGPT group were subsequently formed out of the participants. In the final step, participants in various groups used the internet and ChatGPT (Figure 2C) to evaluate the omissions both in the course and the exercises during the week following the completion of the orthopedic course.

#### Follow-Up Study

For orthopedics, there was a 7-day review period. The ChatGPT group’s students were required to use ChatGPT-4.0 to search up knowledge points to help their learning. Other web-based search engines and message boards were not allowed to be used by the students in this group to search for information. The students in the control group were required to use the internet to support their study by scouring forums and search engines for relevant information. However, they were not allowed to use any software connected to OpenAI. After a week of study using the internet or the ChatGPT, the subjects completed an orthopedics multiple-choice examination. There were 66 questions of type A1, 88 questions of type A2, 29 questions of type A3/A4, and 31 questions requiring case analysis. Participants in diverse groups provided the correct response and total score for each kind of question, which were recorded.

Following the orthopedics examination, the final examination review period was scheduled in accordance with the semester learning plan. We did not obstruct the participants’ learning to complete their reviews at this time. The results of this semester’s internal medicine, surgery, pediatrics, obstetrics and gynecology, and infectious diseases final examinations (Figure 2D) were systematically gathered via the academic affairs office with the participants’ informed consent. Multimedia Appendix 4 contrasts all interventions between the experimental group and the control group.

### Outcomes

In the preparation phase, 2 orthopedics specialists (WYG and ZWX) curated and inputted 1075 orthopedics-related MCQs into ChatGPT-4.0, with the aim of evaluating the accuracy of ChatGPT-4.0 in answering these orthopedics MCQs, while also recording its performance across different question types. We had logged answers from “National Medical Electronic Schoolbag” MCQs and ChatGPT-4.0 responses, uploading the data as Multimedia Appendix 5.

Next, after students completed a 1-week prestudy course in orthopedics and used different methods to review the knowledge for 1 week, we assigned ChatGPT-4.0 as an auxiliary learning review tool to students in the ChatGPT-4.0 group, while students in the control group were not allowed to use large language models (LLMs) for auxiliary review. After the review, we tested all students with the same test questions, enabling us to gauge the quality of short-term revision using different learning auxiliary tools. We recorded the performance of the different student groups in the orthopedics MCQ test, which served as the basis for the short-term impact analysis of the learning intervention.

Subsequently, at the end of the current semester, we accessed and recorded the clinical subject examination scores of the different student groups through the academic system. During the period from the end of our intervention to the end of the final examination, our follow-up content consisted of recording the use of LLMs by the ChatGPT group and the control group. We did not carry out any additional interventions during this time. The scores in the subject examinations at the end of the semester served as the basis for long-term impact analysis of the changes in students’ learning methods after using ChatGPT-4.0. In this study, the accuracy of ChatGPT-4.0, short-term impact analysis, and long-term impact analysis are all primary outcomes.

### Blinding

To eradicate subjective bias in the grading process, the collector was unaware of the classification of the participants when collecting the results from the orthopedics-related multiple-choice exercises and examinations. Other information relied on data from the school’s pedagogical administration system, and the personnel who collected the data did not know the group information of the experiment participants.

### Randomization

After completing the orthopedics-related multiple-choice exercises, the participants were randomly assigned to different groups using the sealed envelope method to minimize systemic bias. To ensure balanced group sizes, a clinician not involved in the program prepared sealed envelopes containing group assignment information. The participants then selected envelopes to determine their group allocation without knowing the group information beforehand. This approach served as a blocking method to achieve balanced group sizes while reducing artificial bias.

### Statistics

For statistical analysis, SPSS software (version 26.0; IBM Corp) was used. The chi-square test was used to analyze gender differences between different groups. The Kolmogorov-Smirnov test was used to determine whether the data exhibited a normal distribution. If the data did not conform to a normal distribution, the Mann-Whitney *U* test was used for analysis. In addition, the data were processed according to the Levene test, and it was determined that the variance was homogeneous; accordingly, an independent-samples 2-tailed *t* test was conducted. When the *P* value was <.05, the difference was considered statistically significant. GraphPad Prism 8 was used to create bar charts. The results of continuous variables were displayed as follows: mean difference between the experimental group and the control group (mean, SD of the difference, *P* value), whereas the accuracy rate of ChatGPT was displayed as: correct number/total number.

### Ethical Considerations

This study was approved by the First Affiliated Hospital of Jinan University’s Ethics Committee (KY-2023-171), and it was also registered in the Chinese Clinical Trials Registry (Chictr2300071774). To ensure confidentiality of the participants, access to the original experimental data necessitates a valid request that is sent to the email address of the corresponding author. Prior to manuscript submission, the research team was required to submit the content of the uploaded materials to the Science and Technology Department of the First Affiliated Hospital of Jinan University for review. This step ensured that no personal information of the participants was disclosed inadvertently. Concurrently, the department was also able to verify the implementation of the participants’ rewards. In accordance with the Declaration of Helsinki, the participants’ written informed consent was obtained before any information about them was acquired.

## Results

### Overview

We began collectively recruiting the Jinan University third-year undergraduate class of medical students on April 1, 2023, and finished the recruitment process on April 7, 2023. We finished the “initial study” and short-term follow-up through the course of the next 2 weeks. The long-term follow-up work was then conducted by gathering the final examination results of the participating students after the semester ended in June 2023. From April 1, 2023, to April 7, 2023, a total of 129 eligible candidates from the Medical College of Jinan University were assessed for participation. During the recruitment phase, 4 individuals dropped out, 7 more during the initial study, and 8 during the follow-up, resulting in a total of 110 participants who completed the study, with 56 in the control group and 54 in the ChatGPT group (Figure 1). As part of our follow-up study, we carried out telephone interviews to assess the extent of ChatGPT usage among the participants in both the control and experimental groups. The interviews revealed that, prior to the final examination, only 2 individuals from the control group had engaged with LLMs. In contrast, every participant in the experimental group had used different types of LLMs to varying degrees.

All of the participants mentioned their ages and grade point averages as of the school year to the researchers after completing an informed consent form for this study. We analyzed age (mean –0.02, SD 0.14 years; *P*=.89), sex (*P*=.44), grade point average (mean 0.10, SD 0.11; *P*=.38), and orthopedic practice accuracy rate (mean 0.12, SD 1.34; *P*=.93) across the 2 groups and found no significant differences after omitting those who were lost to follow-up (Table 1).

**Table 1:** Baseline characteristics of the final participants.

Characteristics	Control group (n=56)	ChatGPT group (n=54)	Total (n=110)	*P* value
Age (years), mean (SD), range	22.46 (0.79), 21-24	22.44 (0.69), 21-24	22.45 (0.74), 21-24	.89
Male sex, n (%)	28 (50)	23 (43)	51:59	.44
Grade point average, mean (SD), range	3.23 (0.51), 2.5-4.3	3.23 (0.68), 2.4-4.4	3.28 (0.60), 2.4-4.4	.38
Orthopedic MCQs^a^ practice accuracy rate, mean (SD), range	56.08 (7.34), 42.94-70.91	56.19 (6.74), 41.44-73.92	56.13 (7.02), 41.44-73.92	.93

^a^MCQ: multiple-choice question.

### The Accuracy of ChatGPT-4.0 Responses to Orthopedics-Related MCQs

**Figure 3:**
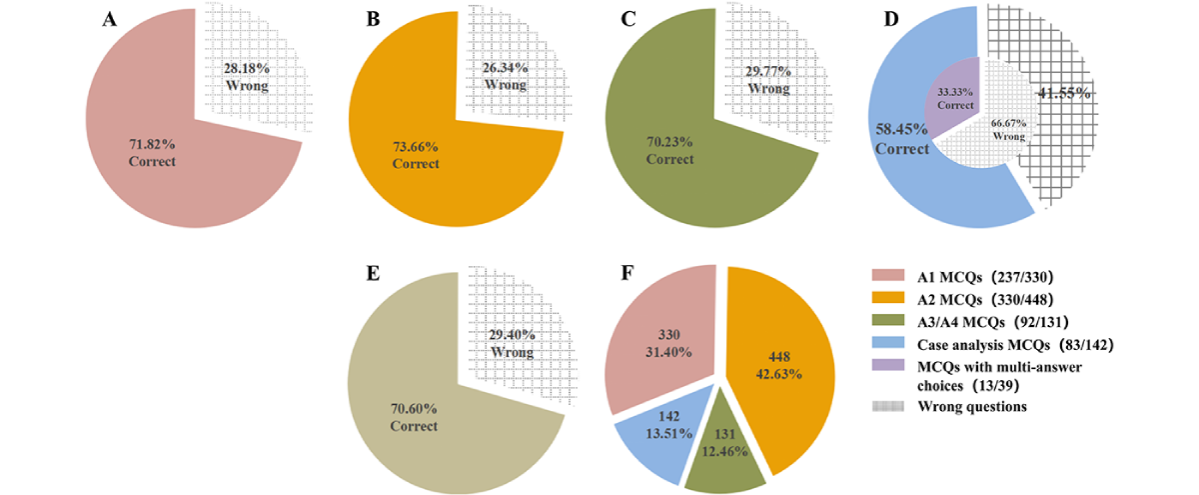
ChatGPT’s performance in answering multiple-choice questions related to orthopedics. (A-D) Accuracy of various types of questions answered by ChatGPT; (E) overall accuracy of questions answered by ChatGPT; and (F) the percentage of each question type in the overall questions. MCQs: multiple-choice questions.

A total of 330 A1 MCQs, 448 A2 MCQs, 131 A3/A4 MCQs, and 142 case analysis MCQs made up to a total of 1051 questions (Figure 3). ChatGPT-4.0 answered the 1051 orthopedics-related MCQs with a satisfactory accuracy rate of 70.60% (742/1051; Figure 3E and 3F). Specifically, the accuracy was 71.8% (237/330) for all A1 MCQs (Figure 3A), 73.7% (330/448) for all A2 MCQs (Figure 3B), and 70.2% (92/131) for all A3/A4 MCQs (Figure 3C), whereas it was only 58.5% (83/142) for all case analysis MCQs (Figure 3D). Among the case analysis MCQs, the accuracy of questions with multiple correct answers was as low as 33% (13/39) (Figure 3D). We found that ChatGPT explained each answer option and enumerated the associated knowledge points (Figure 2A).

### The Accuracy of the 2 Groups of Participants in the Orthopedics Examination

**Figure 4:**
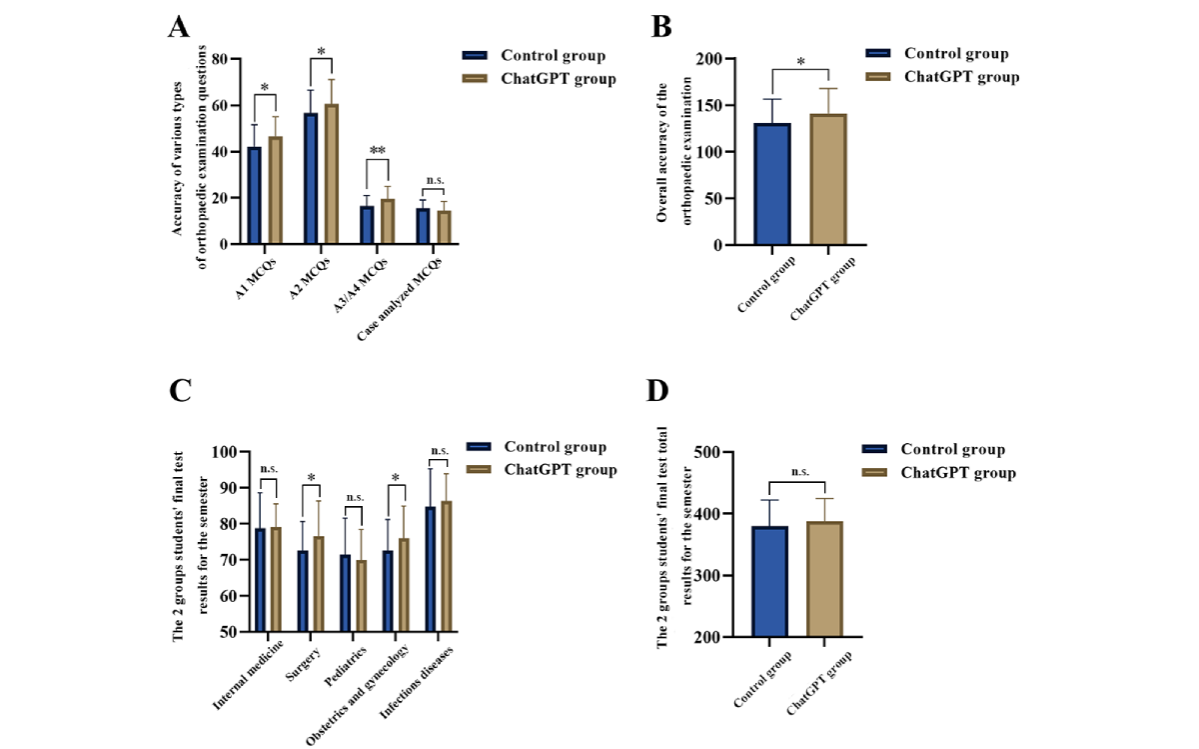
Short-term orthopedics test results and long-term final subjects examination results. (A) Accuracy of various types of orthopedics examination questions; (B) overall accuracy of the orthopedics examination; (C) the 2 groups’ final test results for the semester; and (D) the 2 groups’ final test total results for the semester. ChatGPT group: ChatGPT-4.0–assisted learning group; MCQs: multiple-choice questions; ns: not significant. *P value less than .05; **P value less than .01.

In the orthopedics examination, we were surprised to find out that the participants in the ChatGPT group were typically able to respond to 138.46 (SD 26.97) questions correctly, whereas those in the control group were able to respond to only 130.80 (SD 25.56) questions correctly on average, a significant decline in accuracy (mean 10.40, SD 4.98; *P*=.04; Figure 4B). As shown in Figure 4A, correct answers to A1 (mean 4.40, SD 1.75; *P*=.01), A2 (mean 3.93, SD 1.95; *P*=.047), and A3/4 (mean 3.11, SD 0.96; *P*=.002) among them demonstrated that the ChatGPT group performed significantly better than the control group. Despite the fact that the ChatGPT group showed a lower accuracy in case analysis questions than the control group (mean –1.04, SD 0.72; *P*=.16), the difference was not statistically significant (Figure 4A).

### The Score of the Final Examination for the Semester’s Teaching Task

The statistical findings and the participants’ scores of the final examination for the semester are shown in Figures 4C and 4D. We were pleasantly surprised to find that the ChatGPT-assisted participants scored higher on the final examinations in surgery (mean 4.00, SD 1.72; *P*=.02), internal medicine (mean 0.23, SD 1.58; *P*=.88), obstetrics and gynecology (mean 3.45, SD 1.68; *P*=.04), and infectious diseases (mean 1.60, SD 1.75; *P*=.36) than the control group, although the difference was statistically significant only for surgery and obstetrics and gynecology (Figure 4C). In the pediatric examination, the ChatGPT group scored worse than the control group (mean –1.58, SD 1.79; *P*=.38), but the difference was not statistically significant (Figure 4C). The mean total final examination scores of the ChatGPT group were also higher than those of the control group (mean 7.71, SD 7.53; *P*=.31), but the difference was not statistically significant (Figure 4D).

## Discussion

### Principal Findings

This is the first prospective clinical trial using ChatGPT as an intervention in the instruction of medical undergraduates. We discovered that ChatGPT has a high rate of accuracy when responding to orthopedics-pertinent MCQs, and that it explains each answer option and lists the corresponding knowledge points. After successfully enrolling the final 110 participants in a clinical study, the students using ChatGPT-4.0 were able to have a better comprehension of orthopedics-related knowledge points and performed better on end-of-semester examinations in other disciplines. These findings provide a firm foundation for medical students to use ChatGPT as an auxiliary learning aid in order to improve learning efficacy and promote the use of ChatGPT in medical education.

The purpose of undergraduate medical education is to assist students in developing a strong theoretical foundation in medicine and fundamental clinical abilities. The MCQs test has grown to be one of the most popular tools for objectively evaluating medical theoretical knowledge [32,33]. The aim of our research was to determine whether undergraduate medical students who use ChatGPT as a learning tool in orthopedics can learn more effectively. It is the first prospective randomized controlled trial that used ChatGPT as the main intervention. Through this research, we discovered that using ChatGPT as a learning tool might increase the effectiveness of acquiring information relevant to orthopedics and lead to improved performance on the MCQs test. The short-term follow-up revealed that the students who used ChatGPT as an additional tool for learning also performed better on assessments for the majority of the subjects at the end of the semester, indicating that ChatGPT users might show willing to alter their learning strategies and include ChatGPT as one of their daily learning tools.

We fed ChatGPT-4.0 with the orthopedics-pertinent practice questions and found that it had a respectable accurate answer rate for all of them (A1 [237/330, 71.8%], A2 [330/448, 73.7%], A3/A4 [92/131, 70.2%], and case analysis [83/142, 58.5%]). While doing so, we saw that ChatGPT provided not just knowledge points but also explanations for each answer choice. ChatGPT was also performed at a level of 54.96% accuracy on the Plastic Surgery Inservice Training Examination, which consisted of 242 questions [7]. Because correct rates of 58.5% and 54.96% are not acceptable in clinical work, we believe that ChatGPT is better suited as an auxiliary learning tool for the basic teaching of medical knowledge than for the auxiliary clinical diagnosis and treatment, relying on the powerful and rapid ability to collect and collate data and the ability to answer questions instantly. In addition to textual information such as the patient’s complaint, current history, and prior history, the clinician’s physical examination and imaging must be incorporated into the clinical diagnosis and treatment [34,35]. Although some academics enter the results of physical examinations and imaging examinations into ChatGPT as text for big data analysis [7], the quality of such input content is inconsistent, and systematic errors cannot be ruled out.

The vast and intricate medical knowledge system is the primary source of medical education’s complexity. In addition to cramming for examinations and studying for examinations, medical students must devote significant effort to accumulating and organizing information and making causal links between different domains of knowledge [36,37]. Medical students often reread the medical material many times to ensure a thorough grasp of the material. Moreover, it is also very challenging to realize the transformation of medical knowledge from theory to practice [38,39] because the process of clinical practice requires the interaction between doctors and patients, that is, the immediate output and immediate feedback between them. With the evolution of teaching concepts and learning aids, medical education model has undergone significant transformations over time [40,41]. The traditional education model consists of a 1-way transmission from professors to students [42]. Mass Open Online Course, a nonfixed multidirection input model, has emerged progressively with the advent of the internet [43]. To enhance students’ capacity for self-exploration, education sector constructs problem-based learning and promotes interaction during the learning process [44,45]. Simultaneously, virtual reality–assisted instruction is being developed to enhance medical students’ perceptual comprehension of medical knowledge through the interaction and immediate feedback of programmed audiovisual operations [46]. Through big data analysis and data aggregation, ChatGPT interacts with users via text and provides immediate feedback [5,6]. As LLMs technology continues to evolve and improve, its application prospects in medical education will become even more extensive. Educators should actively embrace this technological innovation [29]. These advanced AI tools can provide personalized learning support, helping students better understand complex medical concepts such as anatomy [47], physiology [48], and biochemistry [48]. Through interactive dialogues with LLMs, students can ask questions, obtain detailed explanations and examples, and deepen their understanding of abstract concepts [49,50]. Moreover, LLMs can generate engaging learning materials, such as clinical case studies, question-and-answer exercises, and knowledge summaries, to enhance student participation and learning motivation [49,51]. This immersive learning experience can stimulate students’ curiosity and encourage them to actively explore knowledge in the biomedical field [51]. Hence, as a supplementary learning aid for medical students, ChatGPT might be able to realize nonprocedural multidirectional interaction and continuously enhance students’ knowledge systems [29] via immediate feedback.

As an auxiliary instrument for medical education, ChatGPT is currently viewed by many academics as a 2-way street [52]. Notably, ChatGPT as a supplementary aid can assist users in collecting the correct target information more efficiently, thereby enhancing work and learning efficiency [5]. However, many academics believe that ChatGPT is merely a plagiarist that lacks initiative and can only collect information, which has become a slang term for slothful people [53-55]. GPTZero (accessed on June 6, 2023), software created by Edward Tian, a student at Princeton University, for statistical analysis of whether text has been generated by AI, has gone a long way toward alleviating the public’s anti-AI sentiment. Instead of emphasizing excessively the “writing shortcuts” provided by ChatGPT’s text-generation function, users should take advantage of its big data rapid retrieval summary and immediate feedback to improve their study and work efficiency. As society, science, and technology advance, it is inevitable that students will take the initiative to adapt and combine their own learning mode with the updates and iteration of learning media and supplementary learning tools [56]. This general trend cannot be reversed. Therefore, society should hold higher standards for teaching methods, teaching content, and student assessment in this irreversible trend.

### Limitations

First, although MCQs are often used to gauge medical students’ fundamental theoretical knowledge in many nations, China may have a distinct custom for framing questions and placing a different priority on knowledge points than other nations. Second, this study focuses more on orthopedic intervention than on multidisciplinary intervention, which might result in some limitations in the research findings. Third, during the preparation stage of this study, while assessing ChatGPT-4.0’s proficiency in answering MCQs pertaining to orthopedics, we made the decision to omit MCQs that included visual elements. This choice might have, to some degree, constrained the breadth of our investigation and consequently fell short of delivering an all-encompassing appraisal of ChatGPT-4.0’s aptitude in tackling a wide spectrum of medical inquiries. This study was a single-center randomized controlled trial; thus, more prospective multicenter and interdisciplinary investigations are required to fully examine ChatGPT’s potential as a teaching tool for medical education.

### Conclusions

ChatGPT has a high rate of accuracy in answering orthopedics-related MCQs, and it explains each answer choice and lists the corresponding knowledge points. Compared with the students assigned to the control group, those in the ChatGPT group had a better understanding of the knowledge points related to orthopedics after a short intervention period and performed better on the end-of-semester examinations in some other disciplines. These findings provided a solid foundation for medical students to use ChatGPT as an auxiliary learning aid to enhance learning efficacy and help promote the use of ChatGPT in medical education.

## Appendix

Multimedia Appendix 1 CONSORT (Consolidated Standards of Reporting Trials) 2010 Checklist.

Multimedia Appendix 2 CONSORT-EHEALTH (Consolidated Standards of Reporting Trials of Electronic and Mobile Health Applications and online TeleHealth; version 1.6.1) checklist.

Multimedia Appendix 3 Multiple-choice questions.

Multimedia Appendix 4 Illustration showing different parts of the training were undertaken by each group.

Multimedia Appendix 5 The reference answers and the responses from ChatGPT-4.0.

## Abbreviations

AI: artificial intelligence
CONSORT: Consolidated Standards of Reporting Trials
CONSORT-EHEALTH: Consolidated Standards of Reporting Trials of Electronic and Mobile Health Applications and online TeleHealth
LLM: large language model
MCQ: multiple-choice question

## Data Availability

The data sets generated and analyzed during this study are available from the corresponding author on reasonable request.
