# Supplementary material for: Integrating ChatGPT in Orthopedic Education for Medical Undergraduates: Randomized Controlled Trial
Source: J Med Internet Res. 2024 Aug 20;26:e57037. doi: 10.2196/57037 (PMC11372336; doi:10.2196/57037)
Supplement: Multimedia Appendix 2 [file jmir_v26i1e57037_app2.pdf]

CONSORT-EHEALTH (V 1.6.1) - Submission/Publication Form

The CONSORT-EHEALTH checklist is intended for authors of randomized trials evaluating web-based and Internet-based applications/interventions, including mobile interventions, electronic games (incl multiplayer games), social media, certain telehealth applications, and other interactive and/or networked electronic applications. Some of the items (e.g. all subitems under item 5 - description of the intervention) may also be applicable for other study designs.

The goal of the CONSORT EHEALTH checklist and guideline is to be
a) a guide for reporting for authors of RCTs,
b) to form a basis for appraisal of an ehealth trial (in terms of validity)

CONSORT-EHEALTH items/subitems are MANDATORY reporting items for studies published in the Journal of Medical Internet Research and other journals / scientific societies endorsing the checklist.

Items numbered 1., 2., 3., 4a., 4b etc are original CONSORT or CONSORT-NPT (non-pharmacologic treatment) items.
Items with Roman numerals (i., ii, iii, iv etc.) are CONSORT-EHEALTH extensions/clarifications.

As the CONSORT-EHEALTH checklist is still considered in a formative stage, we would ask that you also RATE ON A SCALE OF 1-5 how important/useful you feel each item is FOR THE PURPOSE OF THE CHECKLIST and reporting guideline (optional).

Mandatory reporting items are marked with a red *.
In the textboxes, either copy & paste the relevant sections from your manuscript into this form - please include any quotes from your manuscript in QUOTATION MARKS,
or answer directly by providing additional information not in the manuscript, or elaborating on why the item was not relevant for this study.

YOUR ANSWERS WILL BE PUBLISHED AS A SUPPLEMENTARY FILE TO YOUR PUBLICATION IN JMIR AND ARE CONSIDERED PART OF YOUR PUBLICATION (IF ACCEPTED).
Please fill in these questions diligently. Information will not be copyedited, so please use proper spelling and grammar, use correct capitalization, and avoid abbreviations.

DO NOT FORGET TO SAVE AS PDF _AND_ CLICK THE SUBMIT BUTTON SO YOUR ANSWERS ARE IN OUR DATABASE !!!

Citation Suggestion (if you append the pdf as Appendix we suggest to cite this paper in the caption):
Eysenbach G, CONSORT-EHEALTH Group
[illegible]

You're editing your response. Sharing this URL allows others to also edit your response.

FILL OUT A NEW RESPONSE

URL: http://www.jmir.org/2011/4/e126/
doi: 10.2196/jmir.1923 IF: 7.4 Q1 PMID: 22209829 IF: 7.4 Q1

ganwenyitianjing@gmail.com Switch account

Not shared

Resubmit to save

* Indicates required question

Your name *

First Last

Wenyi Gan

Primary Affiliation (short), City, Country *

University of Toronto, Toronto, Canada

Jinan University, Guangzhou, China

Your e-mail address *

abc@gmail.com

garygan2208@163.com

Title of your manuscript *

Provide the (draft) title of your manuscript.

Integrating ChatGPT in Orthopedic Education for Medical Undergraduates: A randomized trial

You're editing your response. Sharing this URL allows others to also edit your response.

FILL OUT A NEW RESPONSE

Name of your App/Software/Intervention *

If there is a short and a long/alternate name, write the short name first and add the long name in brackets.

ChatGPT

Evaluated Version (if any)

e.g. "V1", "Release 2017-03-01", "Version 2.0.27913"

OpenAI's ChatGPT based on the GPT-4 architec

Language(s) *

What language is the intervention/app in? If multiple languages are available, separate by comma (e.g. "English, French")

English,Chinese

URL of your Intervention Website or App

e.g. a direct link to the mobile app on app in appstore (itunes, Google Play), or URL of the website. If the intervention is a DVD or hardware, you can also link to an Amazon page.

https://chat.openai.com/?model=gpt-4

URL of an image/screenshot (optional)

Your answer

You're editing your response. Sharing this URL allows others to also edit your response.

FILL OUT A NEW RESPONSE

Accessibility *

Can an enduser access the intervention presently?

- ○ access is free and open
- ○ access only for special usergroups, not open
- ◉ access is open to everyone, but requires payment/subscription/in-app purchases
- ○ app/intervention no longer accessible
- ○ Other:

Primary Medical Indication/Disease/Condition *

e.g. "Stress", "Diabetes", or define the target group in brackets after the condition, e.g. "Autism (Parents of children with)", "Alzheimers (Informal Caregivers of)"

Orthopaedic learning

Primary Outcomes measured in trial *

comma-separated list of primary outcomes reported in the trial

The accuracy of ChatGPT4.0;Orthopaedic ex

Secondary/other outcomes

Are there any other outcomes the intervention is expected to affect?

The score of the final examination for the semester's teaching task

You're editing your response. Sharing this URL allows others to also edit your response.

FILL OUT A NEW RESPONSE

Recommended "Dose" *

What do the instructions for users say on how often the app should be used?

- (•) Approximately Daily
- ( ) Approximately Weekly
- ( ) Approximately Monthly
- ( ) Approximately Yearly
- ( ) "as needed"
- ( ) Other:

Approx. Percentage of Users (starters) still using the app as recommended after 3 months *

- (•) unknown / not evaluated
- ( ) 0-10%
- ( ) 11-20%
- ( ) 21-30%
- ( ) 31-40%
- ( ) 41-50%
- ( ) 51-60%
- ( ) 61-70%
- ( ) 71%-80%
- ( ) 81-90%
- ( ) 91-100%
- ( ) Other:

You're editing your response. Sharing this URL allows others to also edit your response.

FILL OUT A NEW RESPONSE

Overall, was the app/intervention effective? *

- (selected) yes: all primary outcomes were significantly better in intervention group vs control
- partly: SOME primary outcomes were significantly better in intervention group vs control
- no statistically significant difference between control and intervention
- potentially harmful: control was significantly better than intervention in one or more outcomes
- inconclusive: more research is needed
- Other:

Article Preparation Status/Stage *

At which stage in your article preparation are you currently (at the time you fill in this form)

- not submitted yet - in early draft status
- not submitted yet - in late draft status, just before submission
- submitted to a journal but not reviewed yet
- (selected) submitted to a journal and after receiving initial reviewer comments
- submitted to a journal and accepted, but not published yet
- published
- Other:

You're editing your response. Sharing this URL allows others to also edit your response.

FILL OUT A NEW RESPONSE

Journal *

If you already know where you will submit this paper (or if it is already submitted), please provide the journal name (if it is not JMIR, provide the journal name under "other")

- ○ not submitted yet / unclear where I will submit this
- ◉ Journal of Medical Internet Research (JMIR)
- ○ JMIR mHealth and UHealth
- ○ JMIR Serious Games
- ○ JMIR Mental Health
- ○ JMIR Public Health
- ○ JMIR Formative Research
- ○ Other JMIR sister journal
- ○ Other:

Is this a full powered effectiveness trial or a pilot/feasibility trial? *

- ◉ Pilot/feasibility
- ○ Fully powered

Manuscript tracking number *

If this is a JMIR submission, please provide the manuscript tracking number under "other" (The ms tracking number can be found in the submission acknowledgement email, or when you login as author in JMIR. If the paper is already published in JMIR, then the ms tracking number is the four-digit number at the end of the DOI, to be found at the bottom of each published article in JMIR)

- ○ no ms number (yet) / not (yet) submitted to / published in JMIR
- ◉ Other: 57037

You're editing your response. Sharing this URL allows others to also edit your response.

FILL OUT A NEW RESPONSE

TITLE AND ABSTRACT

1a) TITLE: Identification as a randomized trial in the title

1a) Does your paper address CONSORT item 1a? *

I.e does the title contain the phrase "Randomized Controlled Trial"? (if not, explain the reason under "other")

- (selected) yes
- ( ) Other:

1a-i) Identify the mode of delivery in the title

Identify the mode of delivery. Preferably use “web-based” and/or “mobile” and/or “electronic game” in the title. Avoid ambiguous terms like “online”, “virtual”, “interactive”. Use “Internet-based” only if Intervention includes non-web-based Internet components (e.g. email), use “computer-based” or “electronic” only if offline products are used. Use “virtual” only in the context of “virtual reality” (3-D worlds). Use “online” only in the context of “online support groups”. Complement or substitute product names with broader terms for the class of products (such as “mobile” or “smart phone” instead of “iphone”), especially if the application runs on different platforms.

	1	2	3	4	5	
subitem not at all important	○	○	○	○	◉	essential

Clear selection

Does your paper address subitem 1a-i? *

Copy and paste relevant sections from manuscript title (include quotes in quotation marks "like this" to indicate direct quotes from your manuscript), or elaborate on this item by providing additional information not in the ms, or briefly explain why the item is not applicable/relevant for your study

You're editing your response. Sharing this URL allows others to also edit your response.

FILL OUT A NEW RESPONSE

1a-ii) Non-web-based components or important co-interventions in title

Mention non-web-based components or important co-interventions in title, if any (e.g., “with telephone support”).

	1	2	3	4	5	
subitem not at all important	○	○	○	○	○	essential

Does your paper address subitem 1a-ii?

Copy and paste relevant sections from manuscript title (include quotes in quotation marks "like this" to indicate direct quotes from your manuscript), or elaborate on this item by providing additional information not in the ms, or briefly explain why the item is not applicable/relevant for your study

Your answer

1a-iii) Primary condition or target group in the title

Mention primary condition or target group in the title, if any (e.g., “for children with Type I Diabetes”) Example: A Web-based and Mobile Intervention with Telephone Support for Children with Type I Diabetes: Randomized Controlled Trial

	1	2	3	4	5	
subitem not at all important	○	○	○	○	◉	essential

Clear selection

Does your paper address subitem 1a-iii? *

Copy and paste relevant sections from manuscript title (include quotes in quotation marks "like this" to indicate direct quotes from your manuscript), or elaborate on this item by providing additional information not in the ms, or briefly explain why the item is not applicable/relevant for your study

"Integrating ChatGPT in Orthopedic Education for Medical Undergraduates: A randomized trial". It is important to describe the main subjects of the article.

You're editing your response. Sharing this URL allows others to also edit your response.

FILL OUT A NEW RESPONSE

1b) ABSTRACT: Structured summary of trial design, methods, results, and conclusions

NPT extension: Description of experimental treatment, comparator, care providers, centers, and blinding status.

1b-i) Key features/functionalities/components of the intervention and comparator in the METHODS section of the ABSTRACT

Mention key features/functionalities/components of the intervention and comparator in the abstract. If possible, also mention theories and principles used for designing the site. Keep in mind the needs of systematic reviewers and indexers by including important synonyms. (Note: Only report in the abstract what the main paper is reporting. If this information is missing from the main body of text, consider adding it)

	1	2	3	4	5	
subitem not at all important	○	○	○	○	◉	essential

Clear selection

Does your paper address subitem 1b-i? *

Copy and paste relevant sections from the manuscript abstract (include quotes in quotation marks "like this" to indicate direct quotes from your manuscript), or elaborate on this item by providing additional information not in the ms, or briefly explain why the item is not applicable/relevant for your study

"129 undergraduate medical students participated in a randomised controlled study in which the ChatGPT group utilised ChatGPT as a learning tool while the control group was prohibited from using AI software to support learning."

You're editing your response. Sharing this URL allows others to also edit your response.

FILL OUT A NEW RESPONSE

1b-ii) Level of human involvement in the METHODS section of the ABSTRACT

Clarify the level of human involvement in the abstract, e.g., use phrases like “fully automated” vs. “therapist/nurse/care provider/physician-assisted” (mention number and expertise of providers involved, if any). (Note: Only report in the abstract what the main paper is reporting. If this information is missing from the main body of text, consider adding it)

	1	2	3	4	5	
subitem not at all important	○	○	○	○	○	essential

Does your paper address subitem 1b-ii?

Copy and paste relevant sections from the manuscript abstract (include quotes in quotation marks "like this" to indicate direct quotes from your manuscript), or elaborate on this item by providing additional information not in the ms, or briefly explain why the item is not applicable/relevant for your study

Your answer

1b-iii) Open vs. closed, web-based (self-assessment) vs. face-to-face assessments in the METHODS section of the ABSTRACT

Mention how participants were recruited (online vs. offline), e.g., from an open access website or from a clinic or a closed online user group (closed usergroup trial), and clarify if this was a purely web-based trial, or there were face-to-face components (as part of the intervention or for assessment). Clearly say if outcomes were self-assessed through questionnaires (as common in web-based trials). Note: In traditional offline trials, an open trial (open-label trial) is a type of clinical trial in which both the researchers and participants know which treatment is being administered. To avoid confusion, use “blinded” or “unblinded” to indicated the level of blinding instead of “open”, as “open” in web-based trials usually refers to “open access” (i.e. participants can self-enrol). (Note: Only report in the abstract what the main paper is reporting. If this information is missing from the main body of text, consider adding it)

	1	2	3	4	5	
subitem not at all important	○	○	○	○	○	essential

You're editing your response. Sharing this URL allows others to also edit your response.

FILL OUT A NEW RESPONSE

Does your paper address subitem 1b-iii?

Copy and paste relevant sections from the manuscript abstract (include quotes in quotation marks "like this" to indicate direct quotes from your manuscript), or elaborate on this item by providing additional information not in the ms, or briefly explain why the item is not applicable/relevant for your study

Your answer

1b-iv) RESULTS section in abstract must contain use data

Report number of participants enrolled/assessed in each group, the use/uptake of the intervention (e.g., attrition/adherence metrics, use over time, number of logins etc.), in addition to primary/secondary outcomes. (Note: Only report in the abstract what the main paper is reporting. If this information is missing from the main body of text, consider adding it)

	1	2	3	4	5	
subitem not at all important	○	○	○	○	○	essential

Does your paper address subitem 1b-iv?

Copy and paste relevant sections from the manuscript abstract (include quotes in quotation marks "like this" to indicate direct quotes from your manuscript), or elaborate on this item by providing additional information not in the ms, or briefly explain why the item is not applicable/relevant for your study

Your answer

You're editing your response. Sharing this URL allows others to also edit your response.

FILL OUT A NEW RESPONSE

1b-v) CONCLUSIONS/DISCUSSION in abstract for negative trials

Conclusions/Discussions in abstract for negative trials: Discuss the primary outcome - if the trial is negative (primary outcome not changed), and the intervention was not used, discuss whether negative results are attributable to lack of uptake and discuss reasons. (Note: Only report in the abstract what the main paper is reporting. If this information is missing from the main body of text, consider adding it)

	1	2	3	4	5	
subitem not at all important	○	○	○	○	○	essential

Does your paper address subitem 1b-v?

Copy and paste relevant sections from the manuscript abstract (include quotes in quotation marks "like this" to indicate direct quotes from your manuscript), or elaborate on this item by providing additional information not in the ms, or briefly explain why the item is not applicable/relevant for your study

Your answer

INTRODUCTION

2a) In INTRODUCTION: Scientific background and explanation of rationale

2a-i) Problem and the type of system/solution

Describe the problem and the type of system/solution that is object of the study: intended as stand-alone intervention vs. incorporated in broader health care program? Intended for a particular patient population? Goals of the intervention, e.g., being more cost-effective to other interventions, replace or complement other solutions? (Note: Details about the intervention are provided in “Methods” under 5)

	1	2	3	4	5	
subitem not at all important	○	○	○	◉	○	essential

You're editing your response. Sharing this URL allows others to also edit your response.

FILL OUT A NEW RESPONSE

Does your paper address subitem 2a-i? *

Copy and paste relevant sections from the manuscript (include quotes in quotation marks "like this" to indicate direct quotes from your manuscript), or elaborate on this item by providing additional information not in the ms, or briefly explain why the item is not applicable/relevant for your study

Not applicable.Because the AI industry is growing rapidly,ChatGPT is by far the most powerful.While other intelligent engines exist,they have not yet been studied to verify the full extent of their capabilities.

2a-ii) Scientific background, rationale: What is known about the (type of) system

Scientific background, rationale: What is known about the (type of) system that is the object of the study (be sure to discuss the use of similar systems for other conditions/diagnoses, if appropiate), motivation for the study, i.e. what are the reasons for and what is the context for this specific study, from which stakeholder viewpoint is the study performed, potential impact of findings [2]. Briefly justify the choice of the comparator.

	1	2	3	4	5	
subitem not at all important	○	○	○	○	◉	essential

Clear selection

Does your paper address subitem 2a-ii? *

Copy and paste relevant sections from the manuscript (include quotes in quotation marks "like this" to indicate direct quotes from your manuscript), or elaborate on this item by providing additional information not in the ms, or briefly explain why the item is not applicable/relevant for your study

This step is very important because it represents the research value and social significance of this study.This step is very important because it represents the research value and social significance of this study.It is described in the first and second paragraphs of the "Introduction"section of my article.

You're editing your response. Sharing this URL allows others to also edit your response.

FILL OUT A NEW RESPONSE

Does your paper address CONSORT subitem 2b? *

Copy and paste relevant sections from the manuscript (include quotes in quotation marks "like this" to indicate direct quotes from your manuscript), or elaborate on this item by providing additional information not in the ms, or briefly explain why the item is not applicable/relevant for your study

"Through the results of randomized controlled trials and short-term follow-up,the ultimate objective is to determine whether ChatGPT can be used as an effective learning tool for undergraduate medical students."

METHODS

3a) Description of trial design (such as parallel, factorial) including allocation ratio

Does your paper address CONSORT subitem 3a? *

Copy and paste relevant sections from the manuscript (include quotes in quotation marks "like this" to indicate direct quotes from your manuscript), or elaborate on this item by providing additional information not in the ms, or briefly explain why the item is not applicable/relevant for your study

A parallel-design randomized controlled trial was used for this investigation.First,the accuracy of ChatGPT4.0's responses to orthopedics-related multiple-choice questions (MCQs)was examined.Additionally,for a group experiment,129 third-year medical students from Jinan University's Medical College were recruited.They were divided into two groups at random:the control group and the "ChatGPT4.0-assisted learning group (ChatGPT group). Students using the internet in control group were not permitted to utilize any software or programs associated with Open AI.This study was approved by the First Affiliated Hospital of Jinan University's ethics committee (KY-2023-171),and it was also registered in the Chinese Clinical Trials Registry (Chictr2300071774).“

3b) Important changes to methods after trial commencement (such as eligibility criteria), with reasons

You're editing your response. Sharing this URL allows others to also edit your response.

FILL OUT A NEW RESPONSE

Does your paper address CONSORT subitem 3b? *

Copy and paste relevant sections from the manuscript (include quotes in quotation marks "like this" to indicate direct quotes from your manuscript), or elaborate on this item by providing additional information not in the ms, or briefly explain why the item is not applicable/relevant for your study

There were no important changes to methods after trial commencement.

3b-i) Bug fixes, Downtimes, Content Changes

Bug fixes, Downtimes, Content Changes: ehealth systems are often dynamic systems. A description of changes to methods therefore also includes important changes made on the intervention or comparator during the trial (e.g., major bug fixes or changes in the functionality or content) (5-iii) and other "unexpected events" that may have influenced study design such as staff changes, system failures/downtimes, etc. [2].

	1	2	3	4	5	
subitem not at all important	O	O	O	O	O	essential

Does your paper address subitem 3b-i?

Copy and paste relevant sections from the manuscript (include quotes in quotation marks "like this" to indicate direct quotes from your manuscript), or elaborate on this item by providing additional information not in the ms, or briefly explain why the item is not applicable/relevant for your study

Your answer

4a) Eligibility criteria for participants

You're editing your response. Sharing this URL allows others to also edit your response.

FILL OUT A NEW RESPONSE

Does your paper address CONSORT subitem 4a? *

Copy and paste relevant sections from the manuscript (include quotes in quotation marks "like this" to indicate direct quotes from your manuscript), or elaborate on this item by providing additional information not in the ms, or briefly explain why the item is not applicable/relevant for your study

"Third-year undergraduates from Jinan University's Medical School are the participants in this research.During the evaluation phase,we mostly gathered the results of the orthopedic exercises and orthopedic MCQs tests.We obtained the participants'basic demographic dati as gender,age,and grade point(Table 1),from the educational administration sys fter the participants had agreed and signed the informed consent form.We also obtained the participants'final examination results from the educational administration system after the final exam had concluded.
Inclusion criteria.
Exclusion criteria."

4a-i) Computer / Internet literacy

Computer / Internet literacy is often an implicit “de facto” eligibility criterion - this should be explicitly clarified.

	1	2	3	4	5	
subitem not at all important	○	○	○	○	○	essential

Does your paper address subitem 4a-i?

Copy and paste relevant sections from the manuscript (include quotes in quotation marks "like this" to indicate direct quotes from your manuscript), or elaborate on this item by providing additional information not in the ms, or briefly explain why the item is not applicable/relevant for your study

Your answer

You're editing your response. Sharing this URL allows others to also edit your response.

FILL OUT A NEW RESPONSE

4a-ii) Open vs. closed, web-based vs. face-to-face assessments:

Open vs. closed, web-based vs. face-to-face assessments: Mention how participants were recruited (online vs. offline), e.g., from an open access website or from a clinic, and clarify if this was a purely web-based trial, or there were face-to-face components (as part of the intervention or for assessment), i.e., to what degree got the study team to know the participant. In online-only trials, clarify if participants were quasi-anonymous and whether having multiple identities was possible or whether technical or logistical measures (e.g., cookies, email confirmation, phone calls) were used to detect/prevent these.

	1	2	3	4	5	
subitem not at all important	○	○	○	◉	○	essential

Clear selection

Does your paper address subitem 4a-ii? *

Copy and paste relevant sections from the manuscript (include quotes in quotation marks "like this" to indicate direct quotes from your manuscript), or elaborate on this item by providing additional information not in the ms, or briefly explain why the item is not applicable/relevant for your study

"We began collectively recruiting the Jinan University third-year undergraduate class of medical students on April 1,2023,and we finished the recruitment process on April 7,2023."

4a-iii) Information giving during recruitment

Information given during recruitment. Specify how participants were briefed for recruitment and in the informed consent procedures (e.g., publish the informed consent documentation as appendix, see also item X26), as this information may have an effect on user self-selection, user expectation and may also bias results.

	1	2	3	4	5	
subitem not at all important	○	○	○	○	○	essential

You're editing your response. Sharing this URL allows others to also edit your response.

FILL OUT A NEW RESPONSE

Does your paper address subitem 4a-iii?

Copy and paste relevant sections from the manuscript (include quotes in quotation marks "like this" to indicate direct quotes from your manuscript), or elaborate on this item by providing additional information not in the ms, or briefly explain why the item is not applicable/relevant for your study

Your answer

4b) Settings and locations where the data were collected

Does your paper address CONSORT subitem 4b? *

Copy and paste relevant sections from the manuscript (include quotes in quotation marks "like this" to indicate direct quotes from your manuscript), or elaborate on this item by providing additional information not in the ms, or briefly explain why the item is not applicable/relevant for your study

"The initial study, serving as the primary outcome measure, assessed the impact of different learning aids on participants' performance in multiple-choice practice questions. After completing the concise orthopedics course, the participants were divided into distinct cohorts, each utilizing either the Internet or ChatGPT to identify and rectify any gaps or shortcomings in their practice exercises. Following the culmination of the revision phase, the accuracy rates of the participants from the various groups were meticulously collected and subjected to rigorous analysis.
As for the secondary outcomes, the preparation phase entailed the careful selection and input of orthopedics-related MCQs into ChatGPT4.0. This process was designed to evaluate the knowledge repository of ChatGPT4.0 in terms of its ability to provide accurate responses to text-based MCQs pertaining to orthopedics. In the follow-up study, contingent upon the informed consent of the participants, their final examination scores across different subjects in the current semester were accessed and compiled through the institution's educational system."

You're editing your response. Sharing this URL allows others to also edit your response. FILL OUT A NEW RESPONSE

4b-i) Report if outcomes were (self-)assessed through online questionnaires

Clearly report if outcomes were (self-)assessed through online questionnaires (as common in web-based trials) or otherwise.

	1	2	3	4	5	
subitem not at all important	○	○	○	◉	○	essential

Clear selection

Does your paper address subitem 4b-i? *

Copy and paste relevant sections from the manuscript (include quotes in quotation marks "like this" to indicate direct quotes from your manuscript), or elaborate on this item by providing additional information not in the ms, or briefly explain why the item is not applicable/relevant for your study

The majority of our findings consist of participant-agreed-upon assessment scores.With the participants'assent,baseline participant data and long-term follow-up results were obtained from the school's teaching system.

4b-ii) Report how institutional affiliations are displayed

Report how institutional affiliations are displayed to potential participants [on ehealth media], as affiliations with prestigious hospitals or universities may affect volunteer rates, use, and reactions with regards to an intervention.(Not a required item – describe only if this may bias results)

	1	2	3	4	5	
subitem not at all important	○	○	○	○	○	essential

Does your paper address subitem 4b-ii?

Copy and paste relevant sections from the manuscript (include quotes in quotation marks "like this" to indicate direct quotes from your manuscript), or elaborate on this item by providing additional information not in the ms, or briefly explain why the item is not applicable/relevant for your study

You're editing your response. Sharing this URL allows others to also edit your response.

FILL OUT A NEW RESPONSE

5) The interventions for each group with sufficient details to allow replication, including how and when they were actually administered

5-i) Mention names, credential, affiliations of the developers, sponsors, and owners

Mention names, credential, affiliations of the developers, sponsors, and owners [6] (if authors/evaluators are owners or developer of the software, this needs to be declared in a “Conflict of interest” section or mentioned elsewhere in the manuscript).

	1	2	3	4	5	
subitem not at all important	○	○	○	○	○	essential

Does your paper address subitem 5-i?

Copy and paste relevant sections from the manuscript (include quotes in quotation marks "like this" to indicate direct quotes from your manuscript), or elaborate on this item by providing additional information not in the ms, or briefly explain why the item is not applicable/relevant for your study

Your answer

5-ii) Describe the history/development process

Describe the history/development process of the application and previous formative evaluations (e.g., focus groups, usability testing), as these will have an impact on adoption/use rates and help with interpreting results.

	1	2	3	4	5	
subitem not at all important	○	○	○	○	○	essential

You're editing your response. Sharing this URL allows others to also edit your response.

FILL OUT A NEW RESPONSE

Does your paper address subitem 5-ii?

Copy and paste relevant sections from the manuscript (include quotes in quotation marks "like this" to indicate direct quotes from your manuscript), or elaborate on this item by providing additional information not in the ms, or briefly explain why the item is not applicable/relevant for your study

Your answer

5-iii) Revisions and updating

Revisions and updating. Clearly mention the date and/or version number of the application/intervention (and comparator, if applicable) evaluated, or describe whether the intervention underwent major changes during the evaluation process, or whether the development and/or content was "frozen" during the trial. Describe dynamic components such as news feeds or changing content which may have an impact on the replicability of the intervention (for unexpected events see item 3b).

	1	2	3	4	5	
subitem not at all important	○	○	○	○	○	essential

Does your paper address subitem 5-iii?

Copy and paste relevant sections from the manuscript (include quotes in quotation marks "like this" to indicate direct quotes from your manuscript), or elaborate on this item by providing additional information not in the ms, or briefly explain why the item is not applicable/relevant for your study

Your answer

5-iv) Quality assurance methods

Provide information on quality assurance methods to ensure accuracy and quality of information provided [1], if applicable.

	1	2	3	4	5	
subitem not at all important	○	○	○	○	○	essential

You're editing your response. Sharing this URL allows others to also edit your response.

FILL OUT A NEW RESPONSE

Does your paper address subitem 5-iv?

Copy and paste relevant sections from the manuscript (include quotes in quotation marks "like this" to indicate direct quotes from your manuscript), or elaborate on this item by providing additional information not in the ms, or briefly explain why the item is not applicable/relevant for your study

Your answer

5-v) Ensure replicability by publishing the source code, and/or providing screenshots/screen-capture video, and/or providing flowcharts of the algorithms used

Ensure replicability by publishing the source code, and/or providing screenshots/screen-capture video, and/or providing flowcharts of the algorithms used. Replicability (i.e., other researchers should in principle be able to replicate the study) is a hallmark of scientific reporting.

	1	2	3	4	5	
subitem not at all important	○	○	○	○	○	essential

Does your paper address subitem 5-v?

Copy and paste relevant sections from the manuscript (include quotes in quotation marks "like this" to indicate direct quotes from your manuscript), or elaborate on this item by providing additional information not in the ms, or briefly explain why the item is not applicable/relevant for your study

Your answer

You're editing your response. Sharing this URL allows others to also edit your response.

FILL OUT A NEW RESPONSE

5-vi) Digital preservation

Digital preservation: Provide the URL of the application, but as the intervention is likely to change or disappear over the course of the years; also make sure the intervention is archived (Internet Archive, webcitation.org, and/or publishing the source code or screenshots/videos alongside the article). As pages behind login screens cannot be archived, consider creating demo pages which are accessible without login.

	1	2	3	4	5	
subitem not at all important	○	○	○	○	○	essential

Does your paper address subitem 5-vi?

Copy and paste relevant sections from the manuscript (include quotes in quotation marks "like this" to indicate direct quotes from your manuscript), or elaborate on this item by providing additional information not in the ms, or briefly explain why the item is not applicable/relevant for your study

Your answer

5-vii) Access

Access: Describe how participants accessed the application, in what setting/context, if they had to pay (or were paid) or not, whether they had to be a member of specific group. If known, describe how participants obtained “access to the platform and Internet” [1]. To ensure access for editors/reviewers/readers, consider to provide a “backdoor” login account or demo mode for reviewers/readers to explore the application (also important for archiving purposes, see vi).

	1	2	3	4	5	
subitem not at all important	○	○	○	◉	○	essential

Clear selection

You're editing your response. Sharing this URL allows others to also edit your response.

FILL OUT A NEW RESPONSE

Does your paper address subitem 5-vii? *

Copy and paste relevant sections from the manuscript (include quotes in quotation marks "like this" to indicate direct quotes from your manuscript), or elaborate on this item by providing additional information not in the ms, or briefly explain why the item is not applicable/relevant for your study

"For orthopaedics,there is a severrday review period.The ChatGPT group's pupils are required to use the application ChatGPT4.0 to search up knowledge points to help their learning.Additionally,other Internet search engines and message boards are not allowed to be used by the students in this group to find for information.Students in the Control group are required to use the Internet to support their study by scouring forums and search engines for relevant information.They are not required to utilise any software connected to Open-AI,however."Participants do not need to pay additional fees during the experiment, which is clearly stated in the informed consent"for applying for clinical trials.

5-viii) Mode of delivery, features/functionalities/components of the intervention and comparator, and the theoretical framework

Describe mode of delivery, features/functionalities/components of the intervention and comparator, and the theoretical framework [6] used to design them (instructional strategy [1], behaviour change techniques, persuasive features, etc., see e.g., [7, 8] for terminology). This includes an in-depth description of the content (including where it is coming from and who developed it) [1]," whether [and how] it is tailored to individual circumstances and allows users to track their progress and receive feedback" [6]. This also includes a description of communication delivery channels and – if computer-mediated communication is a component – whether communication was synchronous or asynchronous [6]. It also includes information on presentation strategies [1], including page design principles, average amount of text on pages, presence of hyperlinks to other resources, etc. [1].

	1	2	3	4	5	
subitem not at all important	○	○	○	●	○	essential

Clear selection

You're editing your response. Sharing this URL allows others to also edit your response.

FILL OUT A NEW RESPONSE

Does your paper address subitem 5-viii? *

Copy and paste relevant sections from the manuscript (include quotes in quotation marks "like this" to indicate direct quotes from your manuscript), or elaborate on this item by providing additional information not in the ms, or briefly explain why the item is not applicable/relevant for your study

"For orthopaedics,there is a seven-day review period.The ChatGPT group's pupils are required to use the application ChatGPT4.0 to search up knowledge points to help their learning.Additionally,other Internet search engines and message boards are not allowed to be used by the students in this group to find for information.Students in the Control group are required to use the Internet to support their study by scouring forums and search engines for relevant information.They are not required to utilise any software connected to Open-AI,however."

5-ix) Describe use parameters

Describe use parameters (e.g., intended “doses” and optimal timing for use). Clarify what instructions or recommendations were given to the user, e.g., regarding timing, frequency, heaviness of use, if any, or was the intervention used ad libitum.

	1	2	3	4	5	
subitem not at all important	○	○	○	○	○	essential

Does your paper address subitem 5-ix?

Copy and paste relevant sections from the manuscript (include quotes in quotation marks "like this" to indicate direct quotes from your manuscript), or elaborate on this item by providing additional information not in the ms, or briefly explain why the item is not applicable/relevant for your study

Your answer

You're editing your response. Sharing this URL allows others to also edit your response.

FILL OUT A NEW RESPONSE

5-x) Clarify the level of human involvement

Clarify the level of human involvement (care providers or health professionals, also technical assistance) in the e-intervention or as co-intervention (detail number and expertise of professionals involved, if any, as well as “type of assistance offered, the timing and frequency of the support, how it is initiated, and the medium by which the assistance is delivered”. It may be necessary to distinguish between the level of human involvement required for the trial, and the level of human involvement required for a routine application outside of a RCT setting (discuss under item 21 – generalizability).

	1	2	3	4	5	
subitem not at all important	○	○	○	○	○	essential

Does your paper address subitem 5-x?

Copy and paste relevant sections from the manuscript (include quotes in quotation marks "like this" to indicate direct quotes from your manuscript), or elaborate on this item by providing additional information not in the ms, or briefly explain why the item is not applicable/relevant for your study

Your answer

5-xi) Report any prompts/reminders used

Report any prompts/reminders used: Clarify if there were prompts (letters, emails, phone calls, SMS) to use the application, what triggered them, frequency etc. It may be necessary to distinguish between the level of prompts/reminders required for the trial, and the level of prompts/reminders for a routine application outside of a RCT setting (discuss under item 21 – generalizability).

	1	2	3	4	5	
subitem not at all important	○	○	○	◉	○	essential

Clear selection

You're editing your response. Sharing this URL allows others to also edit your response.

FILL OUT A NEW RESPONSE

Does your paper address subitem 5-xi? *

Copy and paste relevant sections from the manuscript (include quotes in quotation marks "like this" to indicate direct quotes from your manuscript), or elaborate on this item by providing additional information not in the ms, or briefly explain why the item is not applicable/relevant for your study

"For orthopaedics,there is a seven-day review period.The ChatGPT group's pupils are required to use the application ChatGPT4.0 to search up knowledge points to help their learning.Additionally,other Internet search engines and message boards are not allowed to be used by the students in this group to find for information.Students in the Control group are required to use the Internet to support their study by scouring forums and search engines for relevant information.They are not required to utilise any software connected to Open-AI,however."

5-xii) Describe any co-interventions (incl. training/support)

Describe any co-interventions (incl. training/support): Clearly state any interventions that are provided in addition to the targeted eHealth intervention, as ehealth intervention may not be designed as stand-alone intervention. This includes training sessions and support [1]. It may be necessary to distinguish between the level of training required for the trial, and the level of training for a routine application outside of a RCT setting (discuss under item 21 – generalizability.

	1	2	3	4	5	
subitem not at all important	○	○	○	○	◉	essential

Clear selection

Does your paper address subitem 5-xii? *

Copy and paste relevant sections from the manuscript (include quotes in quotation marks "like this" to indicate direct quotes from your manuscript), or elaborate on this item by providing additional information not in the ms, or briefly explain why the item is not applicable/relevant for your study

"For a oneweek orthopaedics course,we first gathered the participants.Following the study,the orthopedics-related multiple-choice exercise was finished,and each participant's accuracy rate was noted."

You're editing your response. Sharing this URL allows others to also edit your response.

FILL OUT A NEW RESPONSE

6a) Completely defined pre-specified primary and secondary outcome measures, including how and when they were assessed

Does your paper address CONSORT subitem 6a? *

Copy and paste relevant sections from the manuscript (include quotes in quotation marks "like this" to indicate direct quotes from your manuscript), or elaborate on this item by providing additional information not in the ms, or briefly explain why the item is not applicable/relevant for your study

"The initial study, serving as the primary outcome measure, assessed the impact of different learning aids on participants' performance in multiple-choice practice questions. After completing the concise orthopedics course, the participants were divided into distinct cohorts, each utilizing either the Internet or ChatGPT to identify and rectify any gaps or shortcomings in their practice exercises. Following the culmination of the revision phase, the accuracy rates of the participants from the various groups were meticulously collected and subjected to rigorous analysis.
As for the secondary outcomes, the preparation phase entailed the careful selection and input of orthopedics-related MCQs into ChatGPT4.0. This process was designed to evaluate the knowledge repository of ChatGPT4.0 in terms of its ability to provide accurate responses to text-based MCQs pertaining to orthopedics. In the follow-up study, contingent upon the informed consent of the participants, their final examination scores across different subjects in the current semester were accessed and compiled through the institution's educational system."

6a-i) Online questionnaires: describe if they were validated for online use and apply CHERRIES items to describe how the questionnaires were designed/deployed

If outcomes were obtained through online questionnaires, describe if they were validated for online use and apply CHERRIES items to describe how the questionnaires were designed/deployed [9].

	1	2	3	4	5	
subitem not at all important	○	○	○	○	○	essential

Does your paper address subitem 6a-i?

Copy and paste relevant sections from manuscript text

You're editing your response. Sharing this URL allows others to also edit your response.

FILL OUT A NEW RESPONSE

6a-ii) Describe whether and how “use” (including intensity of use/dosage) was defined/measured/monitored

Describe whether and how “use” (including intensity of use/dosage) was defined/measured/monitored (logins, logfile analysis, etc.). Use/adoption metrics are important process outcomes that should be reported in any ehealth trial.

	1	2	3	4	5	
subitem not at all important	○	○	○	○	○	essential

Does your paper address subitem 6a-ii?

Copy and paste relevant sections from manuscript text

Your answer

6a-iii) Describe whether, how, and when qualitative feedback from participants was obtained

Describe whether, how, and when qualitative feedback from participants was obtained (e.g., through emails, feedback forms, interviews, focus groups).

	1	2	3	4	5	
subitem not at all important	○	○	○	○	○	essential

Does your paper address subitem 6a-iii?

Copy and paste relevant sections from manuscript text

Your answer

6b) Any changes to trial outcomes after the trial commenced, with reasons

You're editing your response. Sharing this URL allows others to also edit your response.

FILL OUT A NEW RESPONSE

Does your paper address CONSORT subitem 6b? *

Copy and paste relevant sections from the manuscript (include quotes in quotation marks "like this" to indicate direct quotes from your manuscript), or elaborate on this item by providing additional information not in the ms, or briefly explain why the item is not applicable/relevant for your study

No change to trial outcomes after the trial commenced.

7a) How sample size was determined

NPT: When applicable, details of whether and how the clustering by care provides or centers was addressed

7a-i) Describe whether and how expected attrition was taken into account when calculating the sample size

Describe whether and how expected attrition was taken into account when calculating the sample size.

	1	2	3	4	5	
subitem not at all important	○	○	○	○	○	essential

Does your paper address subitem 7a-i?

Copy and paste relevant sections from manuscript title (include quotes in quotation marks "like this" to indicate direct quotes from your manuscript), or elaborate on this item by providing additional information not in the ms, or briefly explain why the item is not applicable/relevant for your study

Your answer

7b) When applicable, explanation of any interim analyses and stopping guidelines

You're editing your response. Sharing this URL allows others to also edit your response.

FILL OUT A NEW RESPONSE

Does your paper address CONSORT subitem 7b? *

Copy and paste relevant sections from the manuscript (include quotes in quotation marks "like this" to indicate direct quotes from your manuscript), or elaborate on this item by providing additional information not in the ms, or briefly explain why the item is not applicable/relevant for your study

"A parallel-design randomized controlled trial was used for this investigation. First, the accuracy of ChatGPT4.0's responses to orthopedics-related MCQs was examined. Additionally, for a group experiment, 129 third-year medical students from Jinan University's Medical College were recruited. They were divided into two groups at random, namely the control group and the ChatGPT4.0-assisted learning group (ChatGPT group). The Internet-using students in the control group were not permitted to use any open-AI-related software or programs, whereas those in the ChatGPT group used only ChatGPT4.0 as the learning tool. In accordance with the Declaration of Helsinki, the participants' written informed consent was obtained before any information about them was acquired. Only after completing the orthopedics course, the orthopedics exercises, the review of the fundamental concepts in orthopedics, using the Internet or ChatGPT, the orthopedic exam, and the final examination for the semester's teaching task did the participants finish the experiment. The detailed process of experimental arrangement is shown in Fig. 1.“

8a) Method used to generate the random allocation sequence

NPT: When applicable, how care providers were allocated to each trial group

Does your paper address CONSORT subitem 8a? *

Copy and paste relevant sections from the manuscript (include quotes in quotation marks "like this" to indicate direct quotes from your manuscript), or elaborate on this item by providing additional information not in the ms, or briefly explain why the item is not applicable/relevant for your study

"After completing the orthopedics-related multiple-choice exercises, the participants were randomly assigned to different groups using the sealed envelope method to minimize systemic bias. To ensure balanced group sizes, a clinician not involved in the program prepared sealed envelopes containing group assignment information. The participants then selected envelopes to determine their group allocation without knowing the group information beforehand. This approach served as a blocking method to achieve balanced group sizes while reducing artificial bias."

You're editing your response. Sharing this URL allows others to also edit your response.

FILL OUT A NEW RESPONSE

Does your paper address CONSORT subitem 8b? *

Copy and paste relevant sections from the manuscript (include quotes in quotation marks "like this" to indicate direct quotes from your manuscript), or elaborate on this item by providing additional information not in the ms, or briefly explain why the item is not applicable/relevant for your study

”Blinding
To eradicate subjective bias in the grading process, the collector was unaware of the classification of the participants when collecting the results from the orthopedics-related multiple-choice exercises and exams. Other information relied on data from the school's pedagogical administration system, and the personnel who collected the data did not know the group information of the experiment participants.
Randomization
After completing the orthopedics-related multiple-choice exercises, the participants were randomly assigned to different groups using the sealed envelope method to minimize systemic bias. To ensure balanced group sizes, a clinician not involved in the program prepared sealed envelopes containing group assignment information. The participants then selected envelopes to determine their group allocation without knowing the group information beforehand. This approach served as a blocking method to achieve balanced group sizes while reducing artificial bias.“

9) Mechanism used to implement the random allocation sequence (such as sequentially numbered containers), describing any steps taken to conceal the sequence until interventions were assigned

You're editing your response. Sharing this URL allows others to also edit your response.

FILL OUT A NEW RESPONSE

Does your paper address CONSORT subitem 9? *

Copy and paste relevant sections from the manuscript (include quotes in quotation marks "like this" to indicate direct quotes from your manuscript), or elaborate on this item by providing additional information not in the ms, or briefly explain why the item is not applicable/relevant for your study

”To eradicate subjective bias in the grading process, the collector was unaware of the classification of the participants when collecting the results from the orthopedics-related multiple-choice exercises and exams. Other information relied on data from the school's pedagogical administration system, and the personnel who collected the data did not know the group information of the experiment participants.After completing the orthopedics-related multiple-choice exercises, the participants were randomly assigned to different groups using the sealed envelope method to minimize systemic bias. To ensure balanced group sizes, a clinician not involved in the program prepared sealed envelopes containing group assignment information. The participants then selected envelopes to determine their group allocation without knowing the group information beforehand. This approach served as a blocking method to achieve balanced group sizes while reducing artificial bias.“

10) Who generated the random allocation sequence, who enrolled participants, and who assigned participants to interventions

Does your paper address CONSORT subitem 10? *

Copy and paste relevant sections from the manuscript (include quotes in quotation marks "like this" to indicate direct quotes from your manuscript), or elaborate on this item by providing additional information not in the ms, or briefly explain why the item is not applicable/relevant for your study

”After completing the orthopedics-related multiple-choice exercises, the participants were randomly assigned to different groups using the sealed envelope method to minimize systemic bias. To ensure balanced group sizes, a clinician not involved in the program prepared sealed envelopes containing group assignment information. The participants then selected envelopes to determine their group allocation without knowing the group information beforehand. This approach served as a blocking method to achieve balanced group sizes while reducing artificial bias.“

11a) If done, who was blinded after assignment to interventions (for example, [illegible]

You're editing your response. Sharing this URL allows others to also edit your response.

FILL OUT A NEW RESPONSE

11a-i) Specify who was blinded, and who wasn't

Specify who was blinded, and who wasn't. Usually, in web-based trials it is not possible to blind the participants [1, 3] (this should be clearly acknowledged), but it may be possible to blind outcome assessors, those doing data analysis or those administering co-interventions (if any).

	1	2	3	4	5	
subitem not at all important	○	○	○	○	◉	essential

Clear selection

Does your paper address subitem 11a-i? *

Copy and paste relevant sections from the manuscript (include quotes in quotation marks "like this" to indicate direct quotes from your manuscript), or elaborate on this item by providing additional information not in the ms, or briefly explain why the item is not applicable/relevant for your study

"To eradicate subjective bias in the grading process, the collector was unaware of the classification of the participants when collecting the results from the orthopedics-related multiple-choice exercises and exams. Other information relied on data from the school's pedagogical administration system, and the personnel who collected the data did not know the group information of the experiment participants."

11a-ii) Discuss e.g., whether participants knew which intervention was the "intervention of interest" and which one was the "comparator"

Informed consent procedures (4a-ii) can create biases and certain expectations - discuss e.g., whether participants knew which intervention was the "intervention of interest" and which one was the "comparator".

	1	2	3	4	5	
subitem not at all important	○	○	○	○	○	essential

You're editing your response. Sharing this URL allows others to also edit your response.

FILL OUT A NEW RESPONSE

Does your paper address subitem 11a-ii?

Copy and paste relevant sections from the manuscript (include quotes in quotation marks "like this" to indicate direct quotes from your manuscript), or elaborate on this item by providing additional information not in the ms, or briefly explain why the item is not applicable/relevant for your study

Your answer

11b) If relevant, description of the similarity of interventions

(this item is usually not relevant for ehealth trials as it refers to similarity of a placebo or sham intervention to a active medication/intervention)

Does your paper address CONSORT subitem 11b? *

Copy and paste relevant sections from the manuscript (include quotes in quotation marks "like this" to indicate direct quotes from your manuscript), or elaborate on this item by providing additional information not in the ms, or briefly explain why the item is not applicable/relevant for your study

"The initial study, serving as the primary outcome measure, assessed the impact of different learning aids on participants' performance in multiple-choice practice questions. After completing the concise orthopedics course, the participants were divided into distinct cohorts, each utilizing either the Internet or ChatGPT to identify and rectify any gaps or shortcomings in their practice exercises. Following the culmination of the revision phase, the accuracy rates of the participants from the various groups were meticulously collected and subjected to rigorous analysis.As for the secondary outcomes, the preparation phase entailed the careful selection and input of orthopedics-related MCQs into ChatGPT4.0. This process was designed to evaluate the knowledge repository of ChatGPT4.0 in terms of its ability to provide accurate responses to text-based MCQs pertaining to orthopedics. In the follow-up study, contingent upon the informed consent of the participants, their final examination scores across different subjects in the current semester were accessed and compiled through the institution's educational system."

12a) Statistical methods used to compare groups for primary and secondary outcomes

NPT: When applicable, details of whether and how the clustering by care providers or centers was addressed

You're editing your response. Sharing this URL allows others to also edit your response. FILL OUT A NEW RESPONSE

Does your paper address CONSORT subitem 12a? *

Copy and paste relevant sections from the manuscript (include quotes in quotation marks "like this" to indicate direct quotes from your manuscript), or elaborate on this item by providing additional information not in the ms, or briefly explain why the item is not applicable/relevant for your study

“For statistical analysis, SPSS version 26.0 software was used. The chi-square test was used to analyze gender differences between different groups. The Kolmogorov–Smirnov test was utilized to determine whether the data exhibited a normal distribution. If the data did not conform to a normal distribution, the Mann–Whitney U test was used for analysis. In addition, the data were processed according to the Levene's test, and it was determined that the variance was homogeneous; accordingly, an independent-samples t test was conducted. When the P value < .05, the difference was considered statistically significant. GraphPad Prism 8 was used to create bar charts. The results of continuous variables were displayed as (mean difference between the experimental group and the control group ± mean standard deviation of the difference, P value), whereas the accuracy rate of ChatGPT was displayed as (correct number/total number).”

12a-i) Imputation techniques to deal with attrition / missing values

Imputation techniques to deal with attrition / missing values: Not all participants will use the intervention/comparator as intended and attrition is typically high in ehealth trials. Specify how participants who did not use the application or dropped out from the trial were treated in the statistical analysis (a complete case analysis is strongly discouraged, and simple imputation techniques such as LOCF may also be problematic [4]).

	1	2	3	4	5	
subitem not at all important	○	○	○	○	●	essential

Clear selection

You're editing your response. Sharing this URL allows others to also edit your response.

FILL OUT A NEW RESPONSE

Does your paper address subitem 12a-i? *

Copy and paste relevant sections from the manuscript (include quotes in quotation marks "like this" to indicate direct quotes from your manuscript), or elaborate on this item by providing additional information not in the ms, or briefly explain why the item is not applicable/relevant for your study

“The exclusion criteria were as follows: failing final exams for six courses in human anatomy, physiology, biochemistry, pathology, pathophysiology, and diagnostics that were completed before; switching majors, stopping classes, or dropping out during the current academic year; refusing to join or leaving in the middle for private reasons; failing to finish the multiple-choice exercises or the orthopedics course; using Open AI-related software or apps in the control group; not completing the orthopedics multiple-choice exam; and missing subjects on the semester's final test.“

12b) Methods for additional analyses, such as subgroup analyses and adjusted analyses

Does your paper address CONSORT subitem 12b? *

Copy and paste relevant sections from the manuscript (include quotes in quotation marks "like this" to indicate direct quotes from your manuscript), or elaborate on this item by providing additional information not in the ms, or briefly explain why the item is not applicable/relevant for your study

”For statistical analysis, SPSS version 26.0 software was used. The chi-square test was used to analyze gender differences between different groups. The Kolmogorov–Smirnov test was utilized to determine whether the data exhibited a normal distribution. If the data did not conform to a normal distribution, the Mann–Whitney U test was used for analysis. In addition, the data were processed according to the Levene's test, and it was determined that the variance was homogeneous; accordingly, an independent-samples t test was conducted. When the P value < .05, the difference was considered statistically significant. GraphPad Prism 8 was used to create bar charts. The results of continuous variables were displayed as (mean difference between the experimental group and the control group ± mean standard deviation of the difference, P value), whereas the accuracy rate of ChatGPT was displayed as (correct number/total number).“

X26) REB/IRB Approval and Ethical Considerations [recommended as subheading under "Methods"] (not a CONSORT item)

You're editing your response. Sharing this URL allows others to also edit your response.

FILL OUT A NEW RESPONSE

X26-i) Comment on ethics committee approval

	1	2	3	4	5	
subitem not at all important	○	○	○	○	○	essential

Does your paper address subitem X26-i?

Copy and paste relevant sections from the manuscript (include quotes in quotation marks "like this" to indicate direct quotes from your manuscript), or elaborate on this item by providing additional information not in the ms, or briefly explain why the item is not applicable/relevant for your study

Your answer

x26-ii) Outline informed consent procedures

Outline informed consent procedures e.g., if consent was obtained offline or online (how? Checkbox, etc.?), and what information was provided (see 4a-ii). See [6] for some items to be included in informed consent documents.

	1	2	3	4	5	
subitem not at all important	○	○	○	○	○	essential

Does your paper address subitem X26-ii?

Copy and paste relevant sections from the manuscript (include quotes in quotation marks "like this" to indicate direct quotes from your manuscript), or elaborate on this item by providing additional information not in the ms, or briefly explain why the item is not applicable/relevant for your study

Your answer

You're editing your response. Sharing this URL allows others to also edit your response.

FILL OUT A NEW RESPONSE

X26-iii) Safety and security procedures

Safety and security procedures, incl. privacy considerations, and any steps taken to reduce the likelihood or detection of harm (e.g., education and training, availability of a hotline)

	1	2	3	4	5	
subitem not at all important	○	○	○	○	○	essential

Does your paper address subitem X26-iii?

Copy and paste relevant sections from the manuscript (include quotes in quotation marks "like this" to indicate direct quotes from your manuscript), or elaborate on this item by providing additional information not in the ms, or briefly explain why the item is not applicable/relevant for your study

Your answer

RESULTS

13a) For each group, the numbers of participants who were randomly assigned, received intended treatment, and were analysed for the primary outcome

NPT: The number of care providers or centers performing the intervention in each group and the number of patients treated by each care provider in each center

You're editing your response. Sharing this URL allows others to also edit your response.

FILL OUT A NEW RESPONSE

Does your paper address CONSORT subitem 13a? *

Copy and paste relevant sections from the manuscript (include quotes in quotation marks "like this" to indicate direct quotes from your manuscript), or elaborate on this item by providing additional information not in the ms, or briefly explain why the item is not applicable/relevant for your study

“During the recruitment phase, four individuals dropped out, seven more during the initial study, and eight during the follow-up, resulting in a total of 110 participants who completed the study, with 56 in the control group and 54 in the ChatGPT group (Fig. 1). As part of our follow-up study, we carried out telephone interviews to assess the extent of ChatGPT usage among the participants in both the control and experimental groups. The interviews revealed that, prior to the final examination, only two individuals from the control group had engaged with large language models. In contrast, every participant in the experimental group had utilized different types of LLMs to varying degrees.”

13b) For each group, losses and exclusions after randomisation, together with reasons

Does your paper address CONSORT subitem 13b? (NOTE: Preferably, this is shown in a CONSORT flow diagram) *

Copy and paste relevant sections from the manuscript (include quotes in quotation marks "like this" to indicate direct quotes from your manuscript), or elaborate on this item by providing additional information not in the ms, or briefly explain why the item is not applicable/relevant for your study

“During the recruitment phase, four individuals dropped out, seven more during the initial study, and eight during the follow-up, resulting in a total of 110 participants who completed the study, with 56 in the control group and 54 in the ChatGPT group (Fig. 1). As part of our follow-up study, we carried out telephone interviews to assess the extent of ChatGPT usage among the participants in both the control and experimental groups. The interviews revealed that, prior to the final examination, only two individuals from the control group had engaged with large language models. In contrast, every participant in the experimental group had utilized different types of LLMs to varying degrees.”

You're editing your response. Sharing this URL allows others to also edit your response.

FILL OUT A NEW RESPONSE

13b-i) Attrition diagram

Strongly recommended: An attrition diagram (e.g., proportion of participants still logging in or using the intervention/comparator in each group plotted over time, similar to a survival curve) or other figures or tables demonstrating usage/dose/engagement.

	1	2	3	4	5	
subitem not at all important	○	○	○	○	○	essential

Does your paper address subitem 13b-i?

Copy and paste relevant sections from the manuscript or cite the figure number if applicable (include quotes in quotation marks "like this" to indicate direct quotes from your manuscript), or elaborate on this item by providing additional information not in the ms, or briefly explain why the item is not applicable/relevant for your study

Your answer

14a) Dates defining the periods of recruitment and follow-up

Does your paper address CONSORT subitem 14a? *

Copy and paste relevant sections from the manuscript (include quotes in quotation marks "like this" to indicate direct quotes from your manuscript), or elaborate on this item by providing additional information not in the ms, or briefly explain why the item is not applicable/relevant for your study

“We began collectively recruiting the Jinan University third-year undergraduate class of medical students on April 1, 2023, and finished the recruitment process on April 7, 2023. ”

You're editing your response. Sharing this URL allows others to also edit your response.

FILL OUT A NEW RESPONSE

14a-i) Indicate if critical “secular events” fell into the study period

Indicate if critical “secular events” fell into the study period, e.g., significant changes in Internet resources available or “changes in computer hardware or Internet delivery resources”

	1	2	3	4	5	
subitem not at all important	○	○	○	○	○	essential

Does your paper address subitem 14a-i?

Copy and paste relevant sections from the manuscript (include quotes in quotation marks "like this" to indicate direct quotes from your manuscript), or elaborate on this item by providing additional information not in the ms, or briefly explain why the item is not applicable/relevant for your study

Your answer

14b) Why the trial ended or was stopped (early)

Does your paper address CONSORT subitem 14b? *

Copy and paste relevant sections from the manuscript (include quotes in quotation marks "like this" to indicate direct quotes from your manuscript), or elaborate on this item by providing additional information not in the ms, or briefly explain why the item is not applicable/relevant for your study

Our experiment process went quite smoothly. However, if any students needed to withdraw mid-way due to personal reasons, we made relevant records.

15) A table showing baseline demographic and clinical characteristics for each group

NPT: When applicable, a description of care providers (case volume, qualification, expertise, etc.) and centers (volume) in each group

You're editing your response. Sharing this URL allows others to also edit your response.

FILL OUT A NEW RESPONSE

Does your paper address CONSORT subitem 15? *

Copy and paste relevant sections from the manuscript (include quotes in quotation marks "like this" to indicate direct quotes from your manuscript), or elaborate on this item by providing additional information not in the ms, or briefly explain why the item is not applicable/relevant for your study

Table 1 showing baseline demographic and clinical characteristics for each group.

15-i) Report demographics associated with digital divide issues

In ehealth trials it is particularly important to report demographics associated with digital divide issues, such as age, education, gender, social-economic status, computer/Internet/ehealth literacy of the participants, if known.

	1	2	3	4	5	
subitem not at all important	○	○	○	○	◉	essential

Clear selection

Does your paper address subitem 15-i? *

Copy and paste relevant sections from the manuscript (include quotes in quotation marks "like this" to indicate direct quotes from your manuscript), or elaborate on this item by providing additional information not in the ms, or briefly explain why the item is not applicable/relevant for your study

"As part of our follow-up study, we carried out telephone interviews to assess the extent of ChatGPT usage among the participants in both the control and experimental groups. The interviews revealed that, prior to the final examination, only two individuals from the control group had engaged with large language models. In contrast, every participant in the experimental group had utilized different types of LLMs to varying degrees.
All of the participants gave their ages and grade point averages as of the school year to the researchers after completing an informed consent form for this study. We analyzed age (−0.02 ± 0.14, P = .89), sex (P = .44), grade point average (0.10 ± 0.11, P = .38), and orthopedic practice accuracy rate (0.12 ± 1.34, P = .93) across the two groups and found no significant differences after omitting those who were lost to follow-up (Table 1)."

You're editing your response. Sharing this URL allows others to also edit your response.

FILL OUT A NEW RESPONSE

16-i) Report multiple “denominators” and provide definitions

Report multiple “denominators” and provide definitions: Report N’s (and effect sizes) “across a range of study participation [and use] thresholds” [1], e.g., N exposed, N consented, N used more than x times, N used more than y weeks, N participants “used” the intervention/comparator at specific pre-defined time points of interest (in absolute and relative numbers per group). Always clearly define “use” of the intervention.

	1	2	3	4	5	
subitem not at all important	○	○	○	○	◉	essential

Clear selection

Does your paper address subitem 16-i? *

Copy and paste relevant sections from the manuscript (include quotes in quotation marks "like this" to indicate direct quotes from your manuscript), or elaborate on this item by providing additional information not in the ms, or briefly explain why the item is not applicable/relevant for your study

"As part of our follow-up study, we carried out telephone interviews to assess the extent of ChatGPT usage among the participants in both the control and experimental groups. The interviews revealed that, prior to the final examination, only two individuals from the control group had engaged with large language models. In contrast, every participant in the experimental group had utilized different types of LLMs to varying degrees.
All of the participants gave their ages and grade point averages as of the school year to the researchers after completing an informed consent form for this study. We analyzed age (−0.02 ± 0.14, P = .89), sex (P = .44), grade point average (0.10 ± 0.11, P = .38), and orthopedic practice accuracy rate (0.12 ± 1.34, P = .93) across the two groups and found no significant differences after omitting those who were lost to follow-up (Table 1)."

16-ii) Primary analysis should be intent-to-treat

Primary analysis should be intent-to-treat, secondary analyses could include comparing only “users”, with the appropriate caveats that this is no longer a randomized sample (see 18-i).

	1	2	3	4	5	
subitem not at all important	○	○	○	○	○	essential

You're editing your response. Sharing this URL allows others to also edit your response. FILL OUT A NEW RESPONSE

Does your paper address subitem 16-ii?

Copy and paste relevant sections from the manuscript (include quotes in quotation marks "like this" to indicate direct quotes from your manuscript), or elaborate on this item by providing additional information not in the ms, or briefly explain why the item is not applicable/relevant for your study

Your answer

17a) For each primary and secondary outcome, results for each group, and the estimated effect size and its precision (such as 95% confidence interval)

Does your paper address CONSORT subitem 17a? *

Copy and paste relevant sections from the manuscript (include quotes in quotation marks "like this" to indicate direct quotes from your manuscript), or elaborate on this item by providing additional information not in the ms, or briefly explain why the item is not applicable/relevant for your study

The presentation in the "Results" section is in accordance with the magazine's requirements.

17a-i) Presentation of process outcomes such as metrics of use and intensity of use

In addition to primary/secondary (clinical) outcomes, the presentation of process outcomes such as metrics of use and intensity of use (dose, exposure) and their operational definitions is critical. This does not only refer to metrics of attrition (13-b) (often a binary variable), but also to more continuous exposure metrics such as “average session length”. These must be accompanied by a technical description how a metric like a “session” is defined (e.g., timeout after idle time) [1] (report under item 6a).

	1	2	3	4	5	
subitem not at all important	○	○	○	○	○	essential

You're editing your response. Sharing this URL allows others to also edit your response.

FILL OUT A NEW RESPONSE

Does your paper address subitem 17a-i?

Copy and paste relevant sections from the manuscript (include quotes in quotation marks "like this" to indicate direct quotes from your manuscript), or elaborate on this item by providing additional information not in the ms, or briefly explain why the item is not applicable/relevant for your study

Your answer

17b) For binary outcomes, presentation of both absolute and relative effect sizes is recommended

Does your paper address CONSORT subitem 17b? *

Copy and paste relevant sections from the manuscript (include quotes in quotation marks "like this" to indicate direct quotes from your manuscript), or elaborate on this item by providing additional information not in the ms, or briefly explain why the item is not applicable/relevant for your study

The presentation in the "Results" section is in accordance with the magazine's requirements.

18) Results of any other analyses performed, including subgroup analyses and adjusted analyses, distinguishing pre-specified from exploratory

Does your paper address CONSORT subitem 18? *

Copy and paste relevant sections from the manuscript (include quotes in quotation marks "like this" to indicate direct quotes from your manuscript), or elaborate on this item by providing additional information not in the ms, or briefly explain why the item is not applicable/relevant for your study

The presentation in the "Results" section is in accordance with the magazine's requirements.

You're editing your response. Sharing this URL allows others to also edit your response.

FILL OUT A NEW RESPONSE

18-i) Subgroup analysis of comparing only users

A subgroup analysis of comparing only users is not uncommon in ehealth trials, but if done, it must be stressed that this is a self-selected sample and no longer an unbiased sample from a randomized trial (see 16-iii).

	1	2	3	4	5	
subitem not at all important	○	○	○	○	○	essential

Does your paper address subitem 18-i?

Copy and paste relevant sections from the manuscript (include quotes in quotation marks "like this" to indicate direct quotes from your manuscript), or elaborate on this item by providing additional information not in the ms, or briefly explain why the item is not applicable/relevant for your study

Your answer

19) All important harms or unintended effects in each group
(for specific guidance see CONSORT for harms)

Does your paper address CONSORT subitem 19? *

Copy and paste relevant sections from the manuscript (include quotes in quotation marks "like this" to indicate direct quotes from your manuscript), or elaborate on this item by providing additional information not in the ms, or briefly explain why the item is not applicable/relevant for your study

No important harm or unintended effect occurs.

You're editing your response. Sharing this URL allows others to also edit your response.

FILL OUT A NEW RESPONSE

19-i) Include privacy breaches, technical problems

Include privacy breaches, technical problems. This does not only include physical "harm" to participants, but also incidents such as perceived or real privacy breaches [1], technical problems, and other unexpected/unintended incidents. "Unintended effects" also includes unintended positive effects [2].

	1	2	3	4	5	
subitem not at all important	○	○	○	○	○	essential

Does your paper address subitem 19-i?

Copy and paste relevant sections from the manuscript (include quotes in quotation marks "like this" to indicate direct quotes from your manuscript), or elaborate on this item by providing additional information not in the ms, or briefly explain why the item is not applicable/relevant for your study

Your answer

19-ii) Include qualitative feedback from participants or observations from staff/researchers

Include qualitative feedback from participants or observations from staff/researchers, if available, on strengths and shortcomings of the application, especially if they point to unintended/unexpected effects or uses. This includes (if available) reasons for why people did or did not use the application as intended by the developers.

	1	2	3	4	5	
subitem not at all important	○	○	○	○	○	essential

Does your paper address subitem 19-ii?

Copy and paste relevant sections from the manuscript (include quotes in quotation marks "like this" to indicate direct quotes from your manuscript), or elaborate on this item by providing additional information not in the ms, or briefly explain why the item is not applicable/relevant for your study

You're editing your response. Sharing this URL allows others to also edit your response.

FILL OUT A NEW RESPONSE

DISCUSSION

22) Interpretation consistent with results, balancing benefits and harms, and considering other relevant evidence

NPT: In addition, take into account the choice of the comparator, lack of or partial blinding, and unequal expertise of care providers or centers in each group

22-i) Restate study questions and summarize the answers suggested by the data, starting with primary outcomes and process outcomes (use)

Restate study questions and summarize the answers suggested by the data, starting with primary outcomes and process outcomes (use).

	1	2	3	4	5	
subitem not at all important	○	○	○	○	◉	essential

Clear selection

Does your paper address subitem 22-i? *

Copy and paste relevant sections from the manuscript (include quotes in quotation marks "like this" to indicate direct quotes from your manuscript), or elaborate on this item by providing additional information not in the ms, or briefly explain why the item is not applicable/relevant for your study

In the "Result" part and "Discussion" part.

22-ii) Highlight unanswered new questions, suggest future research

Highlight unanswered new questions, suggest future research.

	1	2	3	4	5	
subitem not at all important	○	○	○	○	○	essential

You're editing your response. Sharing this URL allows others to also edit your response.

FILL OUT A NEW RESPONSE

Does your paper address subitem 22-ii?

Copy and paste relevant sections from the manuscript (include quotes in quotation marks "like this" to indicate direct quotes from your manuscript), or elaborate on this item by providing additional information not in the ms, or briefly explain why the item is not applicable/relevant for your study

Your answer

20) Trial limitations, addressing sources of potential bias, imprecision, and, if relevant, multiplicity of analyses

20-i) Typical limitations in ehealth trials

Typical limitations in ehealth trials: Participants in ehealth trials are rarely blinded. Ehealth trials often look at a multiplicity of outcomes, increasing risk for a Type I error. Discuss biases due to non-use of the intervention/usability issues, biases through informed consent procedures, unexpected events.

	1	2	3	4	5	
subitem not at all important	○	○	○	○	◉	essential

Clear selection

You're editing your response. Sharing this URL allows others to also edit your response.

FILL OUT A NEW RESPONSE

Does your paper address subitem 20-i? *

Copy and paste relevant sections from the manuscript (include quotes in quotation marks "like this" to indicate direct quotes from your manuscript), or elaborate on this item by providing additional information not in the ms, or briefly explain why the item is not applicable/relevant for your study

"First, although MCQs are often used to gauge medical students' fundamental theoretical knowledge in many nations, China may have a distinct custom for framing questions and placing a different priority on knowledge points than other nations. Second, this study focuses more on orthopedic intervention than multidisciplinary intervention, which might result in some limitations in the research findings. Third, during the preparation stage of this study, while assessing ChatGPT4.0's proficiency in answering MCQs pertaining to orthopedics, we made the decision to omit MCQs that included visual elements. This choice might have, to some degree, constrained the breadth of our investigation and consequently fell short of delivering an all-encompassing appraisal of ChatGPT4.0's aptitude in tackling a wide spectrum of medical inquiries. This study was a single-center randomized controlled trial; thus, more prospective multicenter and interdisciplinary investigations are required to fully examine ChatGPT's potential as a teaching tool for medical education."

21) Generalisability (external validity, applicability) of the trial findings

NPT: External validity of the trial findings according to the intervention, comparators, patients, and care providers or centers involved in the trial

21-i) Generalizability to other populations

Generalizability to other populations: In particular, discuss generalizability to a general Internet population, outside of a RCT setting, and general patient population, including applicability of the study results for other organizations

	1	2	3	4	5	
subitem not at all important	○	○	○	○	○	essential

You're editing your response. Sharing this URL allows others to also edit your response.

FILL OUT A NEW RESPONSE

Does your paper address subitem 21-i?

Copy and paste relevant sections from the manuscript (include quotes in quotation marks "like this" to indicate direct quotes from your manuscript), or elaborate on this item by providing additional information not in the ms, or briefly explain why the item is not applicable/relevant for your study

Your answer

21-ii) Discuss if there were elements in the RCT that would be different in a routine application setting

Discuss if there were elements in the RCT that would be different in a routine application setting (e.g., prompts/reminders, more human involvement, training sessions or other co-interventions) and what impact the omission of these elements could have on use, adoption, or outcomes if the intervention is applied outside of a RCT setting.

	1	2	3	4	5	
subitem not at all important	○	○	○	○	○	essential

Does your paper address subitem 21-ii?

Copy and paste relevant sections from the manuscript (include quotes in quotation marks "like this" to indicate direct quotes from your manuscript), or elaborate on this item by providing additional information not in the ms, or briefly explain why the item is not applicable/relevant for your study

Your answer

OTHER INFORMATION

23) Registration number and name of trial registry

You're editing your response. Sharing this URL allows others to also edit your response.

FILL OUT A NEW RESPONSE

Does your paper address CONSORT subitem 23? *

Copy and paste relevant sections from the manuscript (include quotes in quotation marks "like this" to indicate direct quotes from your manuscript), or elaborate on this item by providing additional information not in the ms, or briefly explain why the item is not applicable/relevant for your study

"This study was approved by the First Affiliated Hospital of Jinan University's ethics committee (KY-2023-171), and it was also registered in the Chinese Clinical Trials Registry (Chictr2300071774). To view details of the registration process, associated accessories, participant compensation and reward structure, and post-experiment privacy protection measures, please refer to the following URL: https://www.chictr.org.cn/hvshowproject.html?id=225740&v=1.0. "

24) Where the full trial protocol can be accessed, if available

Does your paper address CONSORT subitem 24? *

Cite a Multimedia Appendix, other reference, or copy and paste relevant sections from the manuscript (include quotes in quotation marks "like this" to indicate direct quotes from your manuscript), or elaborate on this item by providing additional information not in the ms, or briefly explain why the item is not applicable/relevant for your study

https://www.chictr.org.cn/hvshowproject.html?id=225740&v=1.0

25) Sources of funding and other support (such as supply of drugs), role of funders

Does your paper address CONSORT subitem 25? *

Copy and paste relevant sections from the manuscript (include quotes in quotation marks "like this" to indicate direct quotes from your manuscript), or elaborate on this item by providing additional information not in the ms, or briefly explain why the item is not applicable/relevant for your study

No funding in this RCT. The authors raised their own money.

You're editing your response. Sharing this URL allows others to also edit your response.

FILL OUT A NEW RESPONSE

X27-i) State the relation of the study team towards the system being evaluated

In addition to the usual declaration of interests (financial or otherwise), also state the relation of the study team towards the system being evaluated, i.e., state if the authors/evaluators are distinct from or identical with the developers/sponsors of the intervention.

	1	2	3	4	5	
subitem not at all important	○	○	○	○	○	essential

Does your paper address subitem X27-i?

Copy and paste relevant sections from the manuscript (include quotes in quotation marks "like this" to indicate direct quotes from your manuscript), or elaborate on this item by providing additional information not in the ms, or briefly explain why the item is not applicable/relevant for your study

Your answer

About the CONSORT EHEALTH checklist

As a result of using this checklist, did you make changes in your manuscript? *

- ○ yes, major changes
- ◉ yes, minor changes
- ○ no

What were the most important changes you made as a result of using this checklist?

Your answer

You're editing your response. Sharing this URL allows others to also edit your response.

FILL OUT A NEW RESPONSE

How much time did you spend on going through the checklist INCLUDING making changes in your manuscript *

3 hours spend on going through the checklist.

As a result of using this checklist, do you think your manuscript has improved? *

- (●) yes
- ( ) no
- ( ) Other:

Would you like to become involved in the CONSORT EHEALTH group?

This would involve for example becoming involved in participating in a workshop and writing an "Explanation and Elaboration" document

- (●) yes
- ( ) no
- ( ) Other:

Clear selection

Any other comments or questions on CONSORT EHEALTH

Your answer

You're editing your response. Sharing this URL allows others to also edit your response.

FILL OUT A NEW RESPONSE

STOP - Save this form as PDF before you click submit
To generate a record that you filled in this form, we recommend to generate a PDF of this page (on a Mac, simply select "print" and then select "print as PDF") before you submit it.

When you submit your (revised) paper to JMIR, please upload the PDF as supplementary file.

Don't worry if some text in the textboxes is cut off, as we still have the complete information in our database. Thank you!

Final step: Click submit !
Click submit so we have your answers in our database!

Submit

Never submit passwords through Google Forms.

This content is neither created nor endorsed by Google. Report Abuse - Terms of Service - Privacy Policy

Google Forms

You're editing your response. Sharing this URL allows others to also edit your response.

FILL OUT A NEW RESPONSE

You're editing your response. Sharing this URL allows others to also edit your response.

FILL OUT A NEW RESPONSE
