# Supplementary material for: Integrating ChatGPT in Orthopedic Education for Medical Undergraduates: Randomized Controlled Trial
Source: J Med Internet Res. 2024 Aug 20;26:e57037. doi: 10.2196/57037 (PMC11372336; doi:10.2196/57037)
Supplement: Multimedia Appendix 3 [file jmir_v26i1e57037_app3.docx]

The translation from Chinese to English was performed using ChatGPT-4

First Set of Questions

1. Which of the following options does not conform to the clinical manifestations of bone and joint tuberculosis?

A. Low fever, emaciation, night sweats, anemia

B. Local swelling and pain

C. Accelerated ESR

D. Multiple joint lesions

E. Cold abscess

2. Which of the following is not a cause of primary osteoarthritis?

A. Abnormal cartilage nutrition and metabolism

B. Imbalance of stress

C. Extensive synovial proliferation

D. Abnormal enzymatic degradation of cartilage matrix

E. Accumulative microtrauma

3. The indicator to stop puncture in pyogenic arthritis when local injection of antibiotics is effective is:

A. Significant pain relief

B. Body temperature approaching normal

C. Some functional recovery

D. Disappearance of joint effusion and normal body temperature

E. Basic subsidence of swelling and pain

4. In which situation is surgical treatment for bone and joint tuberculosis not suitable?

A. Formation of sequestrum and large abscess

B. Chronic sinus tract

C. Early total joint tuberculosis, to rescue joint function

D. Spinal tuberculosis with paraplegia

E. Severe systemic toxic symptoms, weakness, severe anemia

5. The main reason why the junction of the middle and lower third of the tibia is prone to fracture is:

A. The shape of the tibia has angles

B. Bearing a large weight

C. Located under the skin with little soft tissue

D. Prone to direct or indirect violence

E. The transition of bone shape

6. Heel pain in patients with ankylosing spondylitis is often due to:

A. Calcaneal spur

B. Bursitis

C. Calcaneal osteitis

D. Osteoporosis

E. Rheumatic myositis

7. The main treatment method for frozen shoulder is:

A. Massage

B. Physical therapy

C. Immobilization

D. Acupuncture

E. Functional exercises

8. Which of the following statements about fracture healing is incorrect?

A. Multiple manual reductions are not conducive to fracture healing

B. Surgery increases the chances of healing more than manual reduction

C. Excessive bone traction can cause delayed healing or nonunion

D. Insufficient internal and external fixation is not conducive to healing

E. Appropriate functional exercises are conducive to healing

9. The most important examination to determine whether a spinal fracture dislocation is accompanied by spinal cord injury is:

A. X-ray examination

B. CT examination

C. MRI examination

D. Neurological examination

E. Neurophysiological examination

10. The most valuable auxiliary examination for the early diagnosis of pyogenic arthritis is:

A. White blood cell count

B. Blood culture

C. Joint cavity puncture and joint fluid examination

D. Arthroscopy

E. X-ray

11. For patients suspected of having a pelvic fracture in the emergency room, the first examination should be:

A. X-ray

B. MRI

C. Radionuclide bone scan

D. Digital rectal examination

E. CT

12. The incorrect statement about cervical traction for cervical spondylosis is:

A. It can relieve muscle spasms

B. It is suitable for all types of cervical spondylosis except the sympathetic type

C. Traction weight is 2-6kg

D. The head is flexed forward 15 degrees during traction

E. It can reposition the synovial folds inside the small joints

13. Mid-humeral fractures are prone to injury of:

A. Median nerve

B. Axillary nerve

C. Ulnar nerve

D. Radial nerve

E. None of the above

14. Which of the following is not an indication for open reduction of fractures?

A. Intra-articular fractures with joint surface displacement exceeding 2mm

B. Manual reduction cannot achieve anatomical reduction

C. Fracture ends with soft tissue entrapment, manual reduction fails

D. Multiple fractures to reduce complications

E. Fracture combined with vascular or nerve injury requiring surgical exploration

15. In the normal weight-bearing line of the lower limb, which three points form a straight line?

A. Anterior superior iliac spine, mid-patella, lower edge of the medial malleolus

B. Anterior superior iliac spine, mid-patella, first web space

C. Greater trochanter, mid-patella, lower edge of the medial malleolus

D. Greater trochanter, mid-patella, first web space

E. Anterior superior iliac spine, mid-patella, mid-ankle joint

16. Which statement about the standards of functional reduction of fractures is incorrect?

A. Rotational displacement at the fracture site must be completely corrected

B. Long bone transverse fractures and metaphyseal fractures should have at least 3/4 alignment

C. Separative displacement at the fracture site must be completely corrected

D. Long bone transverse fractures should have at least 3/4 alignment

E. Angulation displacement in the lower limb must be completely reduced

17. Which of the following is not an early diagnostic reference standard for Kaschin-Beck disease?

A. Finger terminal phalanx bending

B. Bow-shaped finger

C. Suspected knuckle thickening

D. Ankle and knee joint pain

E. Proximal interphalangeal joint pain

18. A breech-delivered infant is found to have left thigh swelling, shortening deformity, and abnormal movement after birth. The first examination to determine the diagnosis should be:

A. Complete blood count

B. Coagulation time

C. X-ray

D. CT

E. MRI

19. Which of the following is not a cause of meniscus injury diagnosis?

A. Joint locking

B. Drawer test (+)

C. McMurray test (+)

D. Grinding test (+)

E. Trauma history

20. Most benign bone tumors of the chest wall are:

A. Bone fibroma

B. Osteoma

C. Chondroma

D. Osteochondroma

E. Lipoma

21. The most diagnostic sign of Colles' fracture is:

A. Local swelling and bone crepitus

B. Typical deformity

C. Local tenderness

D. Abnormal movement

E. Limited hand function

22. Patients should not fulfill which obligation?

A. Self-care obligation

B. Obligation to actively cooperate with treatment

C. Obligation to comply with medical department regulations

D. Exemption from certain social responsibilities and obligations due to illness

E. Obligation to respect medical personnel

23. The characteristic of a spinal tuberculosis abscess is:

A. Often calcified

B. Prone to cough

C. Prone to rupture

D. Acute infection

E. Drains through fascial spaces

24. Which of the following fractures is the least stable?

A. Fissure fracture

B. Transverse fracture

C. Spiral fracture

D. Greenstick fracture

E. Impacted fracture

25. In cases of complete paraplegia due to thoracic spinal cord injury, the muscle strength is graded as:

A. 0

B. 4

C. 2

D. 1

E. 5

26. The most important factor affecting fracture healing is:

A. Type of trauma-induced fracture

B. Blood supply to the fracture site

C. Patient's age

D. Presence of metabolic diseases in the patient

E. Nutritional status

27. Which statement about the diagnosis and treatment of piriformis syndrome is incorrect?

A. Internal rotation of the lower limb aggravates symptoms

B. Localized tenderness in the buttock

C. Surgical release of the piriformis muscle can be performed

D. External rotation of the lower limb aggravates symptoms

E. Localized blocking treatment can be performed

28. The choice of treatment for peripheral nerve injury depends on the cause of the injury. Which option is incorrect?

A. Sciatic nerve injury caused by hip dislocation or pelvic fracture is mostly compression injury, early reduction and observation for 2-3 months, then decide whether surgery is needed based on recovery

B. Early repair for cut injuries

C. Debridement and repair for firearm injuries

D. Local physiotherapy for injection injuries

E. Observation if the epineurium is intact for radial nerve injury caused by humeral fracture

29. Which statement about the clinical healing standards of fractures is incorrect?

A. No axial percussion pain in the affected limb

B. No abnormal movement locally

C. X-ray shows a blurry fracture line

D. No deformation after removing external fixation

E. No local tenderness

30. Which of the following clinical characteristics of primary (postural) scoliosis is incorrect?

A. Mostly occurs in the thoracic or thoracolumbar region, with the spine mostly convex to the right

B. Mostly occurs in children and adolescents, more common in females

C. Accounts for 80% of scoliosis deformities

D. Compensatory secondary curvatures may occur above and below the primary curvature

E. Apart from scoliosis, the spine does not simultaneously have axial rotation

31. The lumbar plexus is composed of fibers from the anterior rami of the 12th thoracic nerve, the anterior rami of the 1st to 3rd lumbar nerves, and part of the anterior ramus of the 4th lumbar nerve. Its branches generally do not include:

A. Lateral femoral cutaneous nerve

B. Sciatic nerve

C. Femoral nerve

D. Iliohypogastric nerve

E. Ilioinguinal nerve

32. Which statement about the sequelae of elbow joint injury is incorrect?

A. The incidence of cubitus varus deformity is

9%-57%, and surgical correction is needed when varus exceeds 20°

B. The incidence of heterotopic ossification is higher in children than in adults

C. Ulnar neuritis can occur after elbow joint injury

D. Surgical treatment should be considered for cubitus valgus over 35°

E. Traumatic heterotopic ossification and progressive heterotopic ossification are not the same disease, with different sites and manifestations

33. Which statement about the clinical healing standards of fractures is incorrect?

A. No axial percussion pain in the affected limb

B. No abnormal movement locally

C. X-ray examination shows continuous callus passing through the fracture line, fracture line disappears or blurs

D. No deformation after removing external fixation

E. No local tenderness

34. If a Class I surgical incision becomes infected postoperatively, the most likely pathogen is:

A. Gram-positive cocci

B. Anaerobic bacteria

C. Fungi

D. Gram-negative bacilli

E. Gram-negative cocci

35. The Drug Administration Law of the People's Republic of China clearly stipulates that prescription drugs shall not be advertised in:

A. Medical journals

B. Pharmaceutical journals

C. Health newspapers

D. Medical newspapers

E. Mass media

36. According to China's Blood Donation Law, which statement about blood collection by medical institutions is correct?

A. Medical institutions are the only non-profit blood collection organizations

B. Medical institutions are the only organizations providing clinical blood

C. To ensure emergency blood use, medical institutions may temporarily collect blood, but must comply with relevant provisions of the Blood Donation Law

D. Medical institutions must file with the health administrative department of the county-level or above people's government when collecting blood from citizens

E. Medical institutions may establish blood collection points to collect blood from citizens after approval by the State Council's health administrative department

37. In cases of severe crush injury with significant limb swelling and venous return obstruction, the primary local treatment should be:

A. Fasciotomy for decompression

B. Limb immobilization

C. Elevation of the affected limb

D. Physiotherapy

E. Promote blood circulation and prevent coagulation

38. Which statement about Kaschin-Beck disease is incorrect?

A. Mainly characterized by cartilage necrosis

B. Related to food poisoning

C. Symmetrical onset

D. Local joint lesions

E. Free bodies can appear in the joint

39. According to the Drug Administration Law, which of the following is not considered a substandard drug?

A. Not labeled with an expiration date or altering the expiration date

B. Indications or functions beyond the prescribed scope

C. Exceeding the expiration date

D. Packaging materials and containers in direct contact with the drug not approved

E. Unauthorized addition of colorants, preservatives, flavors, correctives, and excipients

40. Which statement about compartment syndrome is incorrect?

A. This syndrome is caused by severe ischemia of muscles and nerves in the fascial compartments of the limbs

B. Severe local pain is an urgent signal, and a prompt diagnosis and appropriate treatment should be made

C. Tissue pressure measurement is a diagnostic basis

D. Distal arterial pulse often disappears

E. The affected limb should not be elevated; immediate deep fascia incision is required to relieve intracompartmental high pressure

41. Which statement about congenital hip dislocation is correct?

A. The cause is still unknown

B. A common congenital deformity, more common in females than in males, and more common on the left side than the right

C. Pathological changes involve primary and secondary lesions of the acetabulum, femoral head, neck, and joint capsule

D. The earlier the treatment, the better the outcome

E. All of the above are correct

42. For mid-humeral fractures combined with radial nerve injury, the preferred fixation method is:

A. Plaster fixation

B. Abduction frame fixation

C. Small splint fixation

D. Open reduction and internal fixation

E. No fixation required

43. The most reliable basis for diagnosing adult spinal tuberculosis is:

A. History of low fever and night sweats

B. General weakness and anemia

C. Accelerated ESR

D. Positive tuberculin test

E. X-ray showing blurred edges of adjacent vertebrae and narrowed intervertebral spaces

44. The main reason why suppurative arthritis is easily complicated by pyogenic hip arthritis is:

A. Children's low resistance

B. Epiphyseal fusion

C. Poor blood supply to the femoral neck, low resistance

D. Frequent hip joint activity and heavy load

E. The femoral neck is located within the joint capsule

45. The general X-ray appearance of primary malignant bone tumors is:

A. Bone defect with clear edges and significant periosteal reaction

B. Bone destruction with unclear edges and significant periosteal reaction

C. Bone defect with clear edges and no periosteal reaction

D. Bone destruction with unclear edges and insignificant periosteal reaction

E. Increased bone density with unclear boundaries and significant periosteal reaction

46. The most important measure to prevent postoperative infection is:

A. Enhancing body resistance

B. Extensive use of antibiotics before, during, and after surgery

C. Avoiding shock

D. Early postoperative mobilization

E. Strict adherence to aseptic principles

47. Which preventive measure for lumbar disc herniation is incorrect?

A. Use of a lumbar brace

B. Avoid prolonged sitting

C. Persistent waist bending exercises

D. Strengthening back muscle exercises

E. Regular physiotherapy, massage, and acupuncture

48. Pelvic fractures are prone to associated injuries due to their anatomical position. Which description is incorrect?

A. The pelvic wall is adjacent to multiple venous plexuses, which can bleed after a fracture

B. The cauda equina is located in the sacral canal, and sacral fractures can injure the sacral nerves

C. The sciatic nerve exits through the obturator foramen and can be injured by pubic fractures

D. Ischial ramus fractures can pierce the rectum, causing an open fracture

E. Posterior urethral injuries are mostly caused by pubic fractures

49. Which of the following clinical manifestations should not be considered for acute osteomyelitis?

A. Clear tenderness area in the affected area

B. Elevated white blood cell and neutrophil counts

C. Severe pain at the metaphysis of long bones, unwilling to move the affected limb

D. Varicose veins on the surface of the affected limb, palpable mass

E. Sudden high fever and toxemia symptoms

50. The term "self-discipline" in medical cultivation comes from:

A. "Great Physician Sincerity"

B. "Notes of Middle Way"

C. "Huangdi's Internal Classic"

D. "Theory of Things"

E. "Compendium of Materia Medica"

51. Which of the following measures for treating open joint injuries is incorrect?

A. Prevent joint infection and restore joint function

B. Eliminate factors unfavorable to recovery

C. For first-degree open joint injuries, open the joint capsule and repeatedly rinse with physiological saline

D. For second-degree open joint injuries, try to preserve and repair the joint capsule and ligaments

E. For third-degree open joint injuries, thoroughly debride, leave the wound open, apply sterile dressing, and delay suture for 3-5 days

52. The anteroposterior diameter of the lumbar spinal canal should be less than what measurement to diagnose lumbar spinal stenosis?

A. 10 mm

B. 12 mm

C. 13 mm

D. 15 mm

E. 16 mm

53. What is the clinical treatment method for congenital muscular torticollis?

A. Non-surgical therapy: including hot compress, massage, surgical correction, and head fixation for infants under 3 years old

B. Non-surgical therapy: including hot compress, massage, surgical correction, and head fixation for infants under 5 years old

C. Surgical therapy: cut the sternocleidomastoid muscle for preschool children

D. Surgical therapy: cut the sternocleidomastoid muscle for children over 1 year old

E. Surgical therapy: cut the sternocleidomastoid muscle for children over 12 years old

54. The biceps muscle branch of the musculocutaneous nerve mainly comes from:

A. C5

B. C6

C. C7

D. C8

E. T1

55. A 6-year-old boy with congenital right clubfoot deformity is difficult to correct manually. The preferred surgical treatment is:

A. Muscle balancing surgery

B. Cast correction

C. Achilles tendon lengthening surgery

D. Mckey surgery

E. "Triple joint" fixation after 12 years old

56. The surgical indication for chronic osteomyelitis is:

A. Formation of sequestrum, with ineffective cavity and sinus tract drainage

B. Recurrent fever

C. Extensive periosteal reaction

D. Localized bone sclerosis

E. Significant local swelling

57. The joints fused in the triple joint fixation surgery to correct congenital clubfoot deformity are:

A. Ankle joint, talonavicular joint, calcaneocuboid joint

B. Subtalar joint, talonavicular joint, calcaneocuboid joint

C. Calcaneocuboid joint, tarsometatarsal joint, subtalar joint

D. Talonavicular joint, tarsometatarsal joint, subtalar joint

E. Tarsometatarsal joint, talonavicular joint, calcaneocuboid joint

58. The deformity caused by osteoarthritis is:

A. Buttonhole deformity

B. Square hand

C. Uln

ar deviation of the hand joint

D. Swan neck deformity

E. Clubbing of fingers

59. Radial nerve injury is most commonly seen in:

A. Supracondylar humeral extension fractures

B. Humeral shaft fractures

C. Distal radius fractures

D. Clavicle fractures

E. Proximal third of the ulna fractures

60. Which clinical feature is not included in the characteristics of acute hematogenous osteomyelitis?

A. Common in children under 12 years old

B. Mostly occurs in the metaphysis of long bones

C. The most common pathogen is streptococcus

D. Early diagnosis mainly relies on local stratified puncture

E. X-ray examination generally shows bone destruction and periosteal reaction about 2 weeks after onset

61. The pathological changes in frozen shoulder mainly occur in:

A. The shoulder glenohumeral joint area

B. The shoulder acromioclavicular joint area

C. The deltoid muscle

D. The supraspinatus muscle

E. The infraspinatus muscle

62. Which description of the anatomical features of the portal vein is incorrect?

A. The normal value of portal venous pressure is 13-24 cmH2O

B. The left and right branches of the portal vein enter the left and right halves of the liver, respectively, gradually branching, and then draining into the inferior vena cava

C. The main trunk of the portal vein is formed by the confluence of the superior and inferior mesenteric veins and the splenic vein

D. One end of the portal vein is the capillary network of the stomach, intestines, spleen, and pancreas, and the other end is the hepatic sinusoids in the hepatic lobules

E. The portal vein and the hepatic artery provide equal oxygen supply to the liver

63. The correct procedure for treating a patient with pelvic fracture combined with urethral injury and hemorrhagic shock is:

A. Pelvic fracture - urethral injury - shock

B. Shock - urethral injury - pelvic fracture

C. Shock - pelvic fracture - urethral injury

D. Urethral injury - shock - pelvic fracture

E. Simultaneous treatment of urethral injury, shock, and pelvic fracture

64. The commonly used method for reducing a type I hip dislocation is:

A. Hippocrates method

B. Allis method

C. Knee-chest pull method

D. Kocker method

E. Stimson method

65. Measures to prevent vascular spasm and thrombosis formation after limb replantation surgery do not include:

A. Keeping the limb warm

B. Appropriate use of anticoagulants and antispasmodic drugs

C. Pain management

D. Prohibiting smoking

E. Routine intravenous drip of low molecular weight dextran 500ml for 5-7 weeks

66. Which of the following does not conform to the clinical characteristics of bone metastasis?

A. The incidence of bone metastasis from breast cancer is higher than that from lung cancer

B. Can present as cancerous pain, pathological fractures, spinal cord compression, hypercalcemia, and bone marrow failure

C. Release of soluble mediators activates osteoclasts and osteoblasts, promoting bone destruction

D. So far, there is no effective cure

E. X-ray shows predominantly osteolytic bone destruction

67. The pathological features of acute hematogenous osteomyelitis include:

A. Bone destruction and necrosis

B. Bone destruction and reactive bone proliferation

C. Bone necrosis and reactive bone proliferation

D. Bone destruction

E. Bone destruction/necrosis and reactive bone proliferation

68. The common type of cervical spondylosis causing dizziness and headache is:

A. Nerve root type

B. Spinal cord type

C. Sympathetic nerve type

D. Vertebral artery type

E. Mixed type

69. A patient with symptoms of radial nerve injury two months after humeral fracture surgery is considered to have local scar formation compressing the nerve. For nerve release surgery, the correct application of the tourniquet is:

A. Do not use a tourniquet because of existing ischemia due to nerve compression

B. The nerve has a certain tolerance to ischemia after prolonged compression, so the tourniquet time can be extended by 20-30 minutes

C. Use a tourniquet to obtain a clear view, but reduce the tourniquet time by half due to existing ischemia

D. Use the tourniquet normally, not exceeding 1 hour initially, then release for 10 minutes and reapply for no more than 40 minutes each time

E. None of the above

70. A 42-year-old male with sudden right hip pain and inability to move after being hit on the right knee in a traffic accident should first be considered for the diagnosis of:

A. Hip dislocation

B. Posterior hip dislocation

C. Anterior hip dislocation

D. Hip dislocation combined with knee injury

E. Central hip dislocation

71. In a case where the doctor accidentally injured the artery during bowel cancer surgery, and the patient needs a blood transfusion, the incorrect statement is:

A. Use hospital reserve blood, and if not available, obtain blood from the blood bank

B. In urgent cases, the hospital may temporarily collect blood in compliance with relevant regulations

C. Use voluntary blood donation from the blood bank, with no fees for the patient

D. If the hospital has fulfilled its duty of care during the blood transfusion and no mistakes were made, the infection of the patient due to transfusion is not considered a medical accident

E. The remaining unused portion of the voluntary blood donation from the blood bank cannot be sold to blood product manufacturing companies

72. A 26-year-old female with a machine-cut right index finger with exposed bone and soft tissue loss should not be treated by:

A. Debridement and covering with Vaseline gauze

B. Debridement and ring finger island flap transplantation

C. Debridement and chest wall or upper arm pedicle flap transplantation

D. Debridement and adjacent finger flap transplantation

E. Debridement and shortening and suturing the wound

73. A 10-year-old male with fever, cough, sore throat for 1 week, neck and back pain for 2 days, unable to flex or rotate the head, with no abnormalities in the limbs should have which examination for diagnosis?

A. Cervical CT

B. Cervical MRI

C. Rheumatic blood tests

D. Peripheral blood white cell count and differential

E. Cervical X-ray in lateral and open-mouth views

74. A 31-year-old female with a comminuted fracture of the left olecranon treated surgically 40 days ago now has a limited range of motion to 110° and reduced to less than 90° after 4 months with increased soft tissue density in front of the elbow. The most likely diagnosis is:

A. Heterotopic ossification

B. Traumatic arthritis

C. Post-traumatic hematoma calcification

D. Joint cartilage injury

E. Elbow tuberculosis

75. A 30-year-old male with severe right hip pain and inability to move after a car accident should first be considered for the diagnosis of:

A. Femoral neck fracture

B. Femoral shaft fracture

C. Posterior hip dislocation

D. Anterior hip dislocation

E. Sciatic nerve injury

76. A 3-year-old child with a history of febrile convulsions was admitted for fever and convulsions. On discharge, which statement is incorrect?

A. Actively exercise to strengthen the body and improve resistance, reducing infection and fever chances

B. If convulsions recur, place the child in a lateral position to prevent aspiration of vomit

C. Seek medical help if convulsions last more than 5 minutes to quickly stop the convulsions

D. Wear a mask during flu season to reduce the chances of getting colds and fever

E. Insert a tongue depressor or towel in the child's mouth during convulsions to prevent tongue bites

77. A 38-year-old female industrial worker with right thumb pain, limited flexion and extension, and snapping. The most likely diagnosis is:

A. Rheumatoid arthritis

B. Rheumatoid arthritis

C. Stenosing tenosynovitis

D. Osteoarthritis

E. Osteochondritis

78. A 27-year-old pregnant woman with brain death after a car accident, but with a normal fetal heartbeat, should be managed by:

A. Stopping artificial respiration and waiting for natural cardiac arrest

B. Stopping artificial respiration and taking measures to accelerate cardiac arrest

C. Maintaining artificial respiration and natural cardiac activity

D. Maintaining artificial respiration, supporting cardiac activity, and performing a cesarean section if the fetus can survive

E. Immediate cesarean section to extract the fetus, regardless of survival, and then discontinuing support for the mother

79. A 19-year-old male with a hard, immobile mass in the right proximal thigh discovered by massage. X-ray shows a tumor connected to the lesser trochanter. The diagnosis should be considered as:

A. Giant cell tumor of bone

B. Osteosarcoma

C. Chondrosarcoma

D. Osteomyelitis

E. Osteochondroma

80. A 40-year-old male with pain and swelling of the distal femur and slight knee motion limitation for 21 days with localized swelling and warmth on examination. X-ray shows an eccentric bone absorption lesion. The first diagnosis to consider is:

A. Fibrous dysplasia

B. Giant cell tumor of bone

C. Eosinophilic granuloma

D. Enchondroma

E.

Bone cyst

81. A 10-month-old girl with a cystic mass in the lumbar region growing with age, with a gradually enlarging head and widened cranial sutures. The most likely diagnosis is:

A. Rickets + lumbar tuberculosis

B. Rickets + lymphangioma

C. Lumbar lipoma + hydrocephalus

D. Rickets + myelomeningocele

E. Myelomeningocele + hydrocephalus

82. A 23-year-old male worker with right middle ear cholesteatoma and sigmoid sinus thrombophlebitis needs surgery but lacks a deposit and is accompanied only by colleagues. The best approach is:

A. Admit the patient

B. Admit after receiving a deposit

C. Report to the on-duty senior doctor and hospital management for immediate admission and treatment

D. Observation and intravenous infusion in the emergency room

E. Transfer to another hospital

83. A 24-year-old male with an open tibia and fibula fracture after a car accident should have surgical priorities as:

A. Reduction and internal fixation of tibia and fibula fractures

B. Reduction and internal fixation of tibial fractures

C. Reduction and internal fixation of fibular fractures

D. Thorough debridement to ensure primary wound healing

E. Bone traction and secondary wound healing

84. A 23-year-old female with multiple lacerations on the right hand without major tendon, blood vessel, or bone injuries should not be treated by:

A. Covering the hand and fingers with dressing and bandage

B. Tetanus antitoxin injection

C. Using steroids and dehydration agents to reduce edema

D. Elevating the limb to promote venous return

E. Using antibiotics to prevent infection

85. A 26-year-old male with paraplegia, bilateral leg weakness, and loss of sensation below the xiphoid process with increased reflexes and positive Babinski sign should have an X-ray centered on:

A. T3

B. T5

C. T7

D. T9

E. T11

86. A 1.5-year-old girl with a history of right hip swelling and drainage treatment now walks with a limp, has shortened limbs, and positive Allis and abduction tests, with unclear right femoral head epiphysis and proximal femoral migration on X-ray. The diagnosis is:

A. Congenital hip dislocation

B. Coxa vara

C. Pathological hip dislocation

D. Hip dysplasia

E. Avascular necrosis of the femoral head

87. A 20-year-old male with a history of acute suppurative osteomyelitis and sinus formation now has high fever and pain with sequestrum and a sufficient involucrum on X-ray. The treatment should be:

A. Sinus tract removal and primary suture

B. Bone drilling surgery

C. Sequestrectomy and bone grafting

D. Incision and drainage

E. Sinus tract curettage

88. A 35-year-old male with right leg motion and sensation abnormality after a knife wound to the right buttock should first consider:

A. Sciatic nerve injury

B. Tibial nerve injury

C. Common peroneal nerve injury

D. Gluteus maximus injury

E. Gluteal muscle group injury

89. A 55-year-old female with right iliac and femoral shaft fractures and open tibia and fibula fractures after a car accident should first be aware of which complication?

A. Fat embolism

B. Wound infection

C. Traumatic arthritis

D. Shock

E. Aspiration pneumonia

90. A 30-year-old male driver with recurrent low back pain radiating to the right heel with L5-S1 disc herniation should consider which symptom inconsistent with the diagnosis?

A. Decreased sensation in the right lateral ankle

B. Positive straight leg raise test on the right

C. Significantly weakened right ankle reflex

D. Tenderness between L5-S1 spinous processes radiating to the right lower limb

E. Decreased right toe extensor muscle strength

91. A newborn female with chest asymmetry and syndactyly may also present with:

A. Absence of the long head of the deltoid muscle

B. Absence of the sternal head of the pectoralis major muscle

C. Absence of nipple and areola

D. Bifurcated sternum

E. Macrodactyly

92. A 65-year-old female with wrist pain and swelling after a fall with no abnormal movement but significant fork-like deformity is most likely diagnosed with:

A. Right wrist dislocation

B. Right scaphoid fracture

C. Right wrist Colles' fracture

D. Ulnar styloid fracture

E. Right wrist contusion

93. A 26-year-old female with low back pain, weakness, and fever post-delivery with T9-10 tenderness and decreased sensation below the navel with increased reflexes and accelerated ESR should first consider:

A. Spinal arachnoiditis

B. Spinal tumor

C. Epidural abscess

D. Thoracic tuberculosis

E. Transverse myelitis

94. A 55-year-old female with right knee pain and frequent joint locking for 1 month, with sudden "locking" sensation and severe pain relieved by gentle movement, with positive patellar ballottement and grinding tests and no significant X-ray findings is most likely diagnosed with:

A. Anterior cruciate ligament injury

B. Transient synovitis

C. Loose body in the joint

D. Lateral meniscus injury

E. Rheumatoid arthritis

95. A 4-year-old boy with nocturnal crying and left knee pain not daring to move the leg with normal left knee X-ray should first consider which area?

A. Left knee joint

B. Left hip joint

C. Left tibiotalar joint

D. Left femur

E. Left lower leg

96. A 70-year-old female with a fall and right hip pain with external rotation deformity and percussion pain should be treated by:

A. Skin traction

B. Hip plaster fixation

C. Skeletal traction

D. Intertrochanteric osteotomy

E. Artificial femoral head replacement

97. A 5-year-old boy with early synovial tuberculosis of the hip with low fever, night sweats, emaciation, and right knee pain with semi-flexion deformity and mild limping should first differentiate from:

A. Perthes disease

B. Transient synovitis

C. Rheumatoid arthritis

D. Rheumatic arthritis

E. Early suppurative arthritis

98. A motorcyclist with facial and mandibular open wounds and loss of consciousness after a crash should not use which measure during transport?

A. Lateral or supine position

B. Packing for hemostasis

C. Fixing and bandaging the wound

D. Maintaining airway patency

E. Large doses of analgesics for pain relief

99. A young male with a mediastinal mass undergoing biopsy and rapid pathology progression without payment who refused to pay after receiving the pathology report presents which ethical issue?

A. Infringement of the right to informed consent

B. Infringement of the right to disease recognition

C. Infringement of the right to equal medical care

D. Infringement of privacy

E. Infringement of the right to participation

100. A 41-year-old male with shoulder trauma resulting in clavicle fracture and complete loss of shoulder, elbow, and wrist function with sensory disturbance should be treated by:

A. Early surgical reduction and internal fixation with brachial plexus exploration

B. Manual reduction with figure-8 bandage fixation

C. Manual reduction with plaster fixation

D. Manual reduction with splint fixation

E. Traction reduction

101. Patient, male, 42 years old. Suffered liver and spleen rupture and right femoral fracture due to a car accident, emergency surgery was performed. Twelve hours post-surgery, he gradually developed respiratory distress, clinically diagnosed with Acute Respiratory Distress Syndrome (ARDS). Which of the following examinations is least meaningful?

A. Chest X-ray

B. Arterial blood gas analysis

C. Pulmonary compliance test

D. Pulmonary artery wedge pressure measurement

E. Peak expiratory flow rate (PEFR) measurement

102. Patient, male, 20 years old. Right upper arm contusion resulting in angular deformity in the middle and lower sections. Following the injury, the wrist joint could not actively dorsiflex, and the metacarpophalangeal joints could not actively extend. First consider a fracture of the mid to lower humerus combined with which type of nerve injury?

A. Brachial plexus injury

B. Radial nerve injury

C. Ulnar nerve injury

D. Median nerve injury

E. Musculocutaneous nerve injury

103. Patient, Mr. Song, male, 56 years old. Seeking treatment at a hospital due to a recurrence of erysipelas on his left calf. The doctor prescribed a relatively expensive new antibiotic, but the patient requested to use penicillin, which was effective and cheaper during his last episode. Consequently, the doctor impatiently said, "Do you decide, or do I decide? Do you think I would harm you?" The patient left, perplexed and helpless. Please evaluate the doctor's conduct in terms of medical ethics. The most evident principle violated is:

A. Beneficence

B. Public welfare

C. Justice

D. Respect

E. The value of life

104. Female, 6 years old. Sustained an extension-type supracondylar fracture of the humerus due to trauma. Five hours after manual reduction and external fixation with a plaster cast, she developed finger numbness, active movement restriction, and coldness in the hand. The appropriate immediate treatment is:

A. Immediate removal of the plaster and switch to bone traction treatment

B. Use of vasodilators

C. Cervical plexus block

D. Observe for two days and adopt corresponding treatment measures based on the situation

E. Immediate fasciotomy

105. Boy, 6 years old. Fell from a slide, landing on his right palm, resulting in swelling and pain of the right elbow, with restricted movement. X-ray showed a supracondylar fracture of the right humerus with the distal fracture fragment displaced posteriorly and superiorly. The most likely complication is:

A. Elbow myositis ossificans

B. Triceps tendon injury or rupture

C. Radial or ulnar nerve injury or compression

D. Brachial artery injury or compression

E. Open fracture infection caused by bone fragments piercing the skin

106. Patient sustained a spinal injury, and the lateral X-ray and CT scan showed that the lumbar vertebra 1 was compressed by one-third to one-half. The degree of compression fracture in this patient is:

A. Mild

B. Moderate

C. Severe

D. Extremely severe with nerve damage

E. Extremely severe with nerve damage not classified by this method

107. Patient, male, 27 years old, injured in a traffic accident, presenting with left leg swelling. X-ray showed a transverse fracture of the left tibia in the middle to lower third. The most appropriate treatment plan is:

A. Emergency open reduction and internal fixation with a steel plate

B. Open reduction followed by external fixation with a plaster cast

C. Closed manual reduction with a plaster splint external fixation

D. Skin traction of the affected limb

E. Elevate the affected limb and perform surgery after the swelling subsides

108. Female, 60 years old, unable to walk due to a fall, presenting with left hip pain and an externally rotated left lower limb. Examination showed slight shortening of the left lower limb, positive axial percussion pain, and Pauwels angle of 35°. The best treatment plan is:

A. Tibial tuberosity traction

B. Closed reduction and hip spica cast fixation

C. Orthopedic shoe fixation

D. Closed reduction followed by internal fixation

E. Skin traction

109. Male child, presenting with an emergency after injuring his left elbow. After small splint external fixation, significant swelling of the forearm, cyanosis, pallor, and coldness of the hand developed. X-ray diagnosed a supracondylar fracture of the left humerus. If not treated in time, the most likely consequence is:

A. Infection

B. Ischemic bone necrosis

C. Myositis ossificans

D. Joint stiffness

E. Ischemic contracture

110. For patients under 40, the disease most likely to cause hip osteoarthritis is:

A. Gaucher's disease

B. Increased femoral anteversion angle

C. Congenital hip dislocation

D. Slipped capital femoral epiphysis

E. Congenital hip subluxation

111. Male, 22 years old, suffered a forearm injury from a traffic accident, resulting in skin loss on the palmar side of the forearm with exposed muscles, a wound approximately 8 cm x 6 cm, and concurrent hemopneumothorax. The correct treatment for the forearm wound is:

A. Priority should be given to free flap grafting for repair

B. Only free skin grafting can be used for repair

C. Priority should be given to distant flap repair

D. Only local flap repair can be used

E. Priority should be given to free flap grafting for repair

112. Boy, 6 months old, presenting with congenital bilateral clubfoot. Corrective manipulation can rectify the deformity except for the equinus component. The chosen treatment should be:

A. Muscle balance surgery

B. Kite cast correction

C. Soft tissue release surgery

D. Wait until one year old for surgery

E. McKay surgery

113. Male, 56 years old, injured on the right knee's lateral side in a bicycle accident. Examination revealed the inability to actively dorsiflex the ankle joint. X-ray showed a fibular head fracture. The diagnosis should first consider a fibular head fracture combined with:

A. Sciatic nerve injury

B. Tibial nerve injury

C. Common peroneal nerve injury

D. Anterior tibial muscle tear

E. Long and short peroneal muscle tear

114. Female, 35 years old, experienced left knee joint sprain one year ago, with symptoms resolving after conservative treatment, which the patient did not pay much attention to. In the past three months, the patient developed pain and restricted movement in the left knee, with noticeable swelling for the past month. Aspiration revealed bloody fluid. Physical examination showed diffuse knee joint pain, negative Lachman test, negative anterior drawer test, and positive McMurray sign. The most likely diagnosis is:

A. Left knee ACL injury

B. Left knee synovial chondromatosis

C. Left knee pigmented villonodular synovitis

D. Left knee synovial sarcoma

E. Left knee joint injury syndrome

115. Male, 26 years old. Complaining of constipation, painful defecation, and bleeding for one month. Physical examination in the knee-chest position showed a fresh, shallow, longitudinal ulcer at the 6 o'clock position with smooth edges and an enlarged anal papilla at the dentate line, with a connective tissue external hemorrhoid at the corresponding anal margin. The most likely diagnosis is:

A. Anal fissure

B. Anal sinusitis

C. Anal canal ulcer

D. Connective tissue external hemorrhoid

E. Enlarged anal papilla

116. Male, 24 years old, experienced left knee sprain while playing football, leading to medial swelling, pain, and restricted movement. Symptoms improved after conservative treatment for a month, but occasional joint locking occurred, with significant atrophy of the medial quadriceps head, medial joint space tenderness, positive McMurray sign, negative drawer test, and negative lateral stress test. The most likely diagnosis is:

A. Intra-articular loose bodies

B. Medial meniscus injury

C. Osteomalacia

D. Medial collateral ligament rupture

E. Anterior cruciate ligament rupture

117. Female, 43 years old. Clinically diagnosed with hyperthyroidism, underwent subtotal thyroidectomy under general anesthesia after adequate preoperative preparation. On the second postoperative day, the patient suddenly developed facial and limb spasms. The immediate treatment measure is:

A. Intravenous drip of dexamethasone

B. Oral antithyroid drugs

C. Intramuscular injection of diazepam

D. Oral thyroid tablets

E. Intravenous injection of 10% calcium gluconate

118. Female, 65 years old, suffering from severe left knee pain with walking distance less than 500 meters. Physical examination showed flexion contracture deformity of the left knee joint with limited movement. Weight-bearing X-ray of the knee showed disappearance of the medial joint space, sclerosis, and marginal osteophyte formation.

The most likely diagnosis is:

A. Osteoarthritis

B. Gouty arthritis

C. Suppurative arthritis

D. Tuberculous arthritis

E. Rheumatoid arthritis

119. Female, 29 years old, suffered spinal cord injury from being hit by a heavy object on the lower back. The most accurate physical examination to determine the level of spinal cord injury is:

A. Check tendon reflexes

B. Check limb movement and sensation

C. Check limb temperature

D. Check bulbocavernosus reflex

E. Painful area

120. Male, 54 years old. Suffering from right neck and shoulder pain for two years, accompanied by right-hand numbness for three months. Physical examination revealed loss of cervical lordosis, tenderness between C5 and C6 spinous processes, decreased sensation in the radial side of the right hand, and positive Hoffmann sign. Clinically diagnosed with cervical spondylosis. The most reliable basis for diagnosing cervical spondylosis is:

A. Neck and shoulder pain

B. X-ray showing osteophytes

C. Positive Hoffmann sign on the affected side

D. Loss of cervical lordosis

E. Limited neck movement

121. Female, 40 years old. Complaining of headache and dizziness, aggravated by neck flexion and extension, with sudden falls. Enhanced biceps tendon reflex, cervical X-ray in oblique view showed proliferation of the uncovertebral joint. The most likely diagnosis is:

A. Ménière's disease

B. Positional vertigo

C. Spinal cord tumor

D. Vertebral artery-type cervical spondylosis

E. Adhesive arachnoiditis

122. Male, 52 years old. Suffered abdominal injury due to tunnel collapse during underground work. Physical examination showed pulse 120 beats/min, blood pressure 80/50 mmHg, pale face, significant abdominal tenderness, tenderness at the pubic symphysis, and positive squeezing test. Abdominal puncture revealed about 15 ml of bloody fluid. First consider pelvic fracture combined with:

A. Rectal injury

B. Retroperitoneal hematoma

C. Urethral injury

D. Bladder injury

E. Cauda equina nerve injury

123. Patient, male, 20 years old, worker. Right thigh injured by heavy object at the construction site, unable to stand immediately, with severe local pain. The safest, quickest, and painless examination to preliminarily determine the presence of a fracture is:

A. Examination with a mobile small X-ray machine

B. Check for bone friction sound

C. Check for abnormal movement

D. Check for longitudinal percussion pain

E. Auscultation

124. Patient, male, 25 years old, construction worker. Fell from a height, resulting in back contusion and immediate unconsciousness. Upon regaining consciousness, felt numbness and weakness in all four limbs. Physical examination revealed clear consciousness, central paralysis of all four limbs, and sensory disturbances below C4. The injured site may be:

A. Cervical enlargement

B. Lumbosacral enlargement

C. Thoracic cord

D. Cervical cord above the cervical enlargement

E. Cauda equina nerve

125. Female, 60 years old, experienced recurrent low back pain for 10 years, with worsening pain and intermittent claudication in the past two months, able to walk no more than 100 meters without lower limb radiating pain or numbness, no bladder or bowel dysfunction. Physical examination showed no significant tenderness between the lumbar spinous processes, negative straight leg raising test, normal lower limb sensation, normal knee and Achilles reflexes, and negative Babinski sign on both sides. The diagnosis is:

A. Lumbar disc herniation

B. Lumbar spinal stenosis

C. Lumbar tumor

D. Lumbar tuberculosis

E. Lumbosacral soft tissue strain

126. Patient fell from a height, resulting in neck pain and complete paralysis of both lower limbs. The muscle strength in the lower limbs at this time is:

A. Grade 5

B. Grade 3

C. Grade 2

D. Grade 1

E. Grade 0

127. Patient, male, 24 years old, a porter, experienced sudden low back pain while lifting a heavy object yesterday. Examination revealed tension in the sacrospinal muscles, positive straight leg raising test in the supine position, increased pain when both knees are pressed toward the abdomen in a flexed hip and knee position, and muscle tenderness in the lower back in the prone position. The correct diagnosis is:

A. Lumbar disc herniation

B. Acute lumbar sprain

C. Spondylolisthesis

D. Acute fasciitis

E. Lumbar instability

128. Female, 60 years old, presented with unexplained left upper humeral pain that did not affect sleep. X-ray showed a 2.5 cm round translucent area near the proximal epiphysis of the left humerus, with thin cortical bone. The correct description of this disease is:

A. Treatment involves curettage and bone cement filling

B. The disease is more common in middle-aged people and rare in adolescents

C. Curettage and bone grafting can be performed

D. The disease is prone to metastasis and has a poor prognosis

E. The disease has a higher incidence in females

129. Male, 42 years old, experienced severe upper abdominal pain after a full meal, accompanied by nausea and vomiting for six hours. Vomitus contained stomach contents, with worsening abdominal pain described as knife-like after vomiting, unrelieved by atropine injection. Physical examination showed pulse 124 beats/min, blood pressure 80/50 mmHg, painful expression, abdominal distention, generalized tenderness and rebound tenderness, significant muscle rigidity in the upper abdomen, absent bowel sounds, and hepatic dullness on percussion. Right lower abdominal puncture drew pale red bloody fluid. Laboratory tests revealed WBC 12×10⁹/L with 88% neutrophils, serum amylase 4000 U/L, and blood calcium 1.5 mmol/L. After three hours of observation and treatment, there was no improvement. The most likely diagnosis is:

A. Acute gastric dilatation

B. Gallbladder perforation with diffuse peritonitis

C. Peptic ulcer perforation with diffuse peritonitis

D. Acute hemorrhagic necrotizing pancreatitis

E. Acute strangulated intestinal obstruction

130. Child, male, 4 years old, had fever and purulent cough for two weeks. Body temperature fluctuated between 38-39°C. Chest X-ray showed dense shadow in the right lower lung lobe and right pleural effusion. The most common causative organism is:

A. Streptococcus

B. Anaerobes

C. Pneumococcus

D. Escherichia coli

E. Staphylococcus

131. Male, 30 years old, suffered head trauma resulting in neck stiffness. To determine if there is an atlas fracture, the correct X-ray position to use is:

A. Anteroposterior view

B. Lateral view

C. Oblique view

D. Open-mouth view

E. Tangential view

132. Female, 58 years old, underwent assessment for swallowing difficulties one month after a cerebral infarction, revealing weakened pharyngeal reflex. The likely affected cranial nerve is:

A. IX

B. VI

C. XII

D. V

E. VII

133. Female, 65 years old, presented with joint pain and swelling for two years, with morning stiffness lasting about one hour. Physical examination showed symmetrical swelling and tenderness of the metacarpophalangeal joints. X-ray revealed bone destruction of the second and third metacarpophalangeal joints on both sides. The main drugs for treating this disease do not include:

A. Hydroxychloroquine

B. Leflunomide

C. Sulfasalazine

D. Methotrexate

E. Allopurinol

134. Female, 42 years old, experienced pain and clicking in the right thumb for six months, with inability to extend the thumb actively for one day. Physical examination revealed a pea-sized hard nodule with tenderness on the palmar aspect of the first metacarpophalangeal joint, with a palpable click when the thumb was passively flexed and extended. Based on the patient's symptoms and signs, the most likely diagnosis is:

A. Right flexor pollicis longus tenosynovitis

B. Tumor

C. Rheumatoid nodule

D. Ganglion cyst

E. Osteoarthritis

135. Young male with femoral shaft fracture for one and a half years, presenting with shortening deformity and abnormal movement. X-ray showed both fracture ends sclerosed. The treatment should be:

A. Manual reduction with traction fixation

B. Manual reduction with external fixation

C. Improved nutrition to promote bone healing

D. Strengthened functional exercises

E. Surgical removal of sclerotic bone, bone grafting, and plate fixation with secure external fixation

136. Patient sustained a fibular head fracture, resulting in the inability to dorsiflex the foot. The cause is:

A. Sciatic nerve injury

B. Anterior tibial muscle injury

C. Posterior tibial muscle injury

D. Common peroneal nerve injury

E. Tibial fracture

137. Male, 39 years old, with a fracture of the first lumbar vertebra, retained sensation and movement in both lower limbs but had urinary and fecal incontinence. The most likely diagnosis is:

A. Concurrent damage to the anal sphincter

B. Concurrent damage to the urethral sphincter

C. Spinal cord shock

D. Conus medullaris injury

E. Psychogenic urinary incontinence

138. Female, 66 years old, with right knee joint replacement surgery and compression bandaging. One day postoperatively, she was unable to dorsiflex the right foot, but plantar flexion was normal, with normal dorsalis pedis pulse. The most likely cause is:

A. Common peroneal nerve injury

B. Compartment syndrome

C. Sciatic nerve injury

D. Tibial nerve injury

E. Femoral artery rupture

139. Female, 34 years old, suddenly developed low back pain three days ago, with right leg pain worsened by coughing. Examination showed tenderness in the lumbosacral region radiating to the calf, positive straight leg raising test on the right, decreased sensation in the anterior and lateral aspects of the calf and medial foot, and weakened toe extensor strength. The affected nerve root is:

A. L2

B. L3

C. L4

D. L5

E. S1

140. Child, male, 9 years old, with acute suppurative osteomyelitis for three months, showing a 4 cm long sequestrum in the mid-upper tibia with few involucrum and sinus tracts. Over the past three days, the amount of pus decreased, and he developed fever of 39°C with local redness and swelling. The inappropriate treatment measure is:

A. Intravenous antibiotics in large doses

B. Small amount of blood transfusion

C. Local immobilization

D. Surgical removal of sequestrum

E. Incision and drainage

141. Female, 26 years old, had her right middle finger amputated by a machine. During surgery, the tendons that should be sutured as much as possible are:

A. Flexor digitorum profundus tendon and flexor digitorum superficialis tendon

B. Flexor digitorum profundus tendon and extensor digitorum tendon

C. Flexor digitorum superficialis tendon and extensor digitorum tendon

D. Flexor digitorum profundus tendon

E. Flexor digitorum superficialis tendon

142. Patient, male, hospitalized due to sensory and motor dysfunction in the right upper limb after a motorcycle accident. Physical examination showed skin abrasions on the neck and shoulder, inability to abduct the shoulder joint, inability to flex the elbow joint, weakened wrist flexion and extension strength, but normal grip strength. The most likely diagnosis is:

A. Axillary nerve injury

B. Ulnar nerve injury

C. Brachial plexus injury

D. Humeral surgical neck fracture

E. Mid-humeral fracture

143. Child, male, 6 years old, with left torticollis, palpable mass in the left sternocleidomastoid muscle, and normal cervical X-ray. The appropriate treatment is:

A. Mass excision

B. Plaster orthosis

C. Cutting the sternocleidomastoid muscle above the clavicle + plaster orthosis

D. Physical therapy

E. Total resection of the sternocleidomastoid muscle

144. Young male, with neck pain and limb weakness for four years, presenting with significantly reduced sensation below the second intercostal space. The most meaningful auxiliary examination for diagnosis is:

A. Thoracic spine X-ray

B. Cervical spine X-ray

C. Cervical spine MRI

D. Thoracic spine MRI

E. Comprehensive physical examination

145. Young male diagnosed with lumbar tuberculosis, planned for surgery. Preoperative standard antituberculosis treatment should last at least:

A. 1 week

B. 2 weeks

C. 3 weeks

D. 4 weeks

E. 8 weeks

146. Female, 70 years old, experienced bilateral knee pain for ten years, worsening in the past year, with increased pain after walking long distances. Physical examination showed bilateral knee varus, negative floating patella test, and knee range of motion of 95°-10°-0°. X-ray showed narrowing of the medial joint space with peripheral osteophyte formation. The diagnosis is:

A. Ankylosing spondylitis

B. Rheumatoid arthritis

C. Osteoarthritis

D. Suppurative arthritis

E. Traumatic arthritis

147. Child, male, 6 years old, developed sudden chills, high fever, and severe right knee pain for three days. Physical examination showed body temperature of 39°C, right knee pain, inability to move, refusal of palpation, and no significant swelling. The most likely diagnosis is:

A. Chronic osteomyelitis

B. Suppurative arthritis

C. Acute hematogenous osteomyelitis

D. Rheumatoid arthritis

E. Osteochondritis of the tibial tubercle

148. Male, 38 years old, five days post-appendectomy, complained of wound pain, fever, and fatigue. Physical examination showed a body temperature of 39.0°C, mild wound edema, no exudate, no redness or swelling, and significant tenderness. Clinically considered likely to have incision infection, the immediate treatment measure is:

A. Change antibiotics

B. Remove incision sutures, open the wound for drainage

C. Diagnostic puncture of the incision

D. Physical therapy, daily wound dressing change

E. Physical cooling, continued observation

149. Worker, male, experienced tightness and weakness in both lower limbs for four months, followed by difficulty walking and weakened grip strength. Examination showed increased muscle tone, weakened muscle strength, irregular areas of sensory decrease, and positive Hoffmann sign. The most likely diagnosis is:

A. Transverse myelitis

B. Polyneuritis

C. Cervical spondylotic myelopathy

D. Amyotrophic lateral sclerosis

E. Syringomyelia

150. Child, female, 4 years old, injured her right elbow one hour ago. Physical examination showed significant swelling of the right elbow with subcutaneous ecchymosis. The ulnar side of the hand showed sensory loss, inability to adduct the thumb, and weakness of the other four fingers. X-ray showed a supracondylar fracture of the right humerus. The most likely diagnosis is a supracondylar fracture of the humerus with injury to the:

A. Median nerve

B. Ulnar nerve

C. Radial nerve

D. Recurrent branch of the median nerve

E. Superficial branch of the radial nerve

151. Female, 20 years old, two years post-TB debridement surgery of the 7th and 8th thoracic vertebrae. In the past three months, she developed night sweats, low-grade fever, back pain, and generalized weakness. Physical examination showed significant percussion pain in the 7th and 8th thoracic vertebrae, bilateral lower limb muscle strength grade 3, and positive Babinski sign. The appropriate treatment measure is:

A. Anti-tuberculosis treatment with bed rest on a plaster bed

B. Anti-tuberculosis treatment with debridement surgery if necessary

C. Debridement surgery followed by anti-tuberculosis treatment

D. Standard anti-tuberculosis treatment with early debridement surgery

E. Posterior spinal fusion surgery

152. Female, 48 years old, suffering from left shoulder pain for three months, gradually worsening, unable to comb hair, with restricted abduction and extension of the left shoulder joint, and deltoid muscle atrophy. No numbness in the arm. The most likely diagnosis is:

A. Cervical spondylosis

B. Shoulder tumor

C. Adhesive capsulitis

D. Shoulder tuberculosis

E. Rheumatoid arthritis

153. Male, 78 years old, experienced chronic low back pain, intermittent claudication, difficulty walking, but able to ride a bicycle easily. The most likely diagnosis is:

A. Lumbar spinal stenosis

B. Osteoporosis

C. Lumbar disc herniation

D. Osteoarthritis

E. Piriformis syndrome

154. Female, 39 years old, with right femoral shaft fracture, presenting with shortening deformity and abnormal movement two years post-treatment. X-ray showed both fracture ends sclerosed. The treatment should be:

A. Improved nutrition to promote bone healing

B. Manual reduction with external fixation

C. Manual reduction with traction fixation

D. Strengthened functional exercises

E. Surgical removal of sclerotic bone, bone grafting, and plate fixation with secure external fixation

155. Child, female, 7 years old, developed unexplained pain in the upper left humerus, not affecting sleep. X-ray showed a 2.5 cm round translucent area near the proximal epiphysis of the left humerus, with thin cortical bone. The correct description of this disease is:

A. Treatment involves curettage and bone cement filling

B. The disease is more common in middle-aged people and rare in adolescents

C. Curettage and bone grafting can be performed

D. The disease is prone to metastasis and has a poor prognosis

E. The disease has a higher incidence in females

156. Female, 42 years old, experienced sudden low back pain seven days ago, with left leg pain worsening after half a day and no relief with rest. Examination showed tenderness in the lumbosacral region radiating to the calf, positive straight leg raising test on the right, decreased sensation in the lateral calf and foot, and weakened plantar flexion strength. The affected nerve root is:

A. L2

B. L3

C. L4

D. L5

E. S1

157. Male, 20 years old, suffered tibiofibular fracture, underwent open reduction and internal fixation with plates. At the end of the surgery, the calf was significantly

swollen, tense, and difficult to close the wound. The best method for closing the wound is:

A. Do not suture temporarily, perform delayed suture

B. Try to suture directly

C. Open wound dressing until healing

D. Perform decompressive incisions on both sides and suture the original wound

E. Perform decompressive incisions on both sides, suture the original wound, and cover the decompressive incision area with free skin grafts

158. Child, male, 11 years old, with right knee joint tuberculosis, presenting with local swelling, joint flexion, and holding the knee with both hands, moaning in discomfort. In addition to anti-tuberculosis medication, the following treatment should be performed:

A. Continuous skin traction

B. Plaster fixation

C. Incision and drainage

D. Joint puncture

E. Physical therapy

159. Female, 35 years old, fell backward while walking, landing on her right palm. After the injury, she experienced right shoulder pain and restricted movement. Physical examination showed right shoulder flat shoulder deformity and positive Dugas sign. The clinical diagnosis should first consider:

A. Right shoulder soft tissue injury

B. Anterior dislocation of the right shoulder joint

C. Fracture of the lateral epicondyle of the humerus

D. Fracture of the anatomical neck of the humerus

E. Acromioclavicular joint dislocation

160. Female, 35 years old, fell backward while walking, landing on her right palm. After the injury, she experienced right shoulder pain and restricted movement. Physical examination showed right shoulder flat shoulder deformity and positive Dugas sign. The first auxiliary examination to be performed is:

A. Anteroposterior X-ray

B. Lateral X-ray

C. Anteroposterior and trans-thoracic X-ray

D. CT

E. Shoulder arthroscopy

161. Male, 45 years old, experienced chronic low back pain with intermittent severity, accompanied by bilateral lower limb pain. Ten days ago, he developed increased pain and numbness in the lower back and legs, with difficulty urinating. Physical examination showed limited lumbar movement, paraspinal tenderness radiating to the lower limbs, and decreased sensation in the saddle area. Positive straight leg raising test and positive enhancement test. The cause of difficulty urinating and saddle numbness is:

A. Irritation of the sinuvertebral nerve

B. Compression of the posterior branches of the spinal nerves

C. Compression of the cauda equina nerve

D. Compression of the U nerve root

E. Compression of the S nerve root

162. Male, 45 years old, experienced chronic low back pain with intermittent severity, accompanied by bilateral lower limb pain. Ten days ago, he developed increased pain and numbness in the lower back and legs, with difficulty urinating. Physical examination showed limited lumbar movement, paraspinal tenderness radiating to the lower limbs, and decreased sensation in the saddle area. Positive straight leg raising test and positive enhancement test. The most necessary differential diagnosis is:

A. Lumbar sprain

B. Lumbar spinal stenosis

C. Third lumbar transverse process syndrome

D. Intraspinal tumor

E. Ankylosing spondylitis

163. Male, 45 years old, experienced chronic low back pain with intermittent severity, accompanied by bilateral lower limb pain. Ten days ago, he developed increased pain and numbness in the lower back and legs, with difficulty urinating. Physical examination showed limited lumbar movement, paraspinal tenderness radiating to the lower limbs, and decreased sensation in the saddle area. Positive straight leg raising test and positive enhancement test. The appropriate treatment is:

A. Bed rest on a hardboard bed

B. Traction

C. Epidural prednisone injection

D. Surgery

E. Hormones, non-steroidal anti-inflammatory drugs

164. Female, 15 years old, presented with right forearm laceration caused by glass. Examination revealed difficulty in thumb and finger extension. The first consideration should be:

A. Median nerve injury

B. Deep branch of the radial nerve injury

C. Ulnar nerve injury

D. Musculocutaneous nerve injury

E. Axillary nerve injury

165. Female, 15 years old, presented with right forearm laceration caused by glass. Examination revealed difficulty in thumb opposition. The first consideration should be:

A. Musculocutaneous nerve injury

B. Axillary nerve injury

C. Radial nerve injury

D. Median nerve injury

E. Ulnar nerve injury

166. Female, 15 years old, presented with right forearm laceration caused by glass. Examination revealed sensory reduction in the ulnar one and a half fingers. The first consideration should be:

A. Radial nerve injury

B. Musculocutaneous nerve injury

C. Median nerve injury

D. Ulnar nerve injury

E. Axillary nerve injury

167. Male, 36 years old, suffered a right leg injury in a car accident two hours ago, presenting with a 16 cm long oblique wound contaminated with sand and soil, with exposed tibial ends, minimal bleeding, and extensive soft tissue contusion. The first step before taking X-rays is:

A. Simple external fixation and local dressing

B. Tourniquet hemostasis

C. Debridement

D. Calcaneal tuberosity traction

E. Intravenous infusion and antibiotic administration

168. Male, 36 years old, suffered a right leg injury in a car accident two hours ago, presenting with a 16 cm long oblique wound contaminated with sand and soil, with exposed tibial ends, minimal bleeding, and extensive soft tissue contusion. After thorough debridement, which of the following treatment measures violates the treatment principle?

A. External fixation with a frame

B. Place a silicone tube in the deepest part of the wound, connected to a negative pressure drainage bottle, and remove it after 24-48 hours

C. Use antibiotics to prevent infection

D. Primary wound closure to convert the open wound to a closed wound

E. Tetanus antitoxin injection

169. Male, 15 years old, right wrist laceration caused by a sharp object eight hours ago, treated with disinfection and dressing, antibiotics, and tetanus antitoxin. Examination revealed the inability to abduct the right little finger. The first consideration should be:

A. Interosseous muscle injury

B. Flexor tendon injury

C. Radial nerve injury

D. Ulnar nerve injury

E. Median nerve injury

170. Male, 15 years old, right wrist laceration caused by a sharp object eight hours ago, treated with disinfection and dressing, antibiotics, and tetanus antitoxin. Examination revealed the inability to abduct the right little finger. The appropriate treatment measure is:

A. Observation

B. Secondary suture

C. Delayed suture

D. Primary debridement

E. Secondary debridement

171. Male, 14 years old, presented with supracondylar fracture of the left humerus, treated with manual reduction and external fixation with a plaster splint. The key observation for early detection of compartment syndrome includes:

A. Disappearance of radial pulse

B. Skin temperature and color of the hand and forearm

C. Degree of limb swelling

D. Presence of sensory disturbances

E. Presence of rest pain, increased pain on passive finger extension, and aggravation of pain on finger extension

172. Male, 14 years old, presented with supracondylar fracture of the left humerus, treated with manual reduction and external fixation with a plaster splint. The most severe complication is:

A. Volkmann's contracture

B. Syndactyly

C. Claw hand deformity

D. Wrist drop deformity

E. Mallet finger deformity

173. Male, 75 years old, fell and landed on his right hip, resulting in right hip pain and inability to walk. Examination revealed a 90° external rotation deformity of the right lower limb with limb shortening. The most likely diagnosis is:

A. Femoral neck fracture

B. Intertrochanteric fracture

C. Femoral shaft fracture

D. Hip dislocation

E. Acetabular fracture

174. Male, 75 years old, fell and landed on his right hip, resulting in right hip pain and inability to walk. Examination revealed a 90° external rotation deformity of the right lower limb with limb shortening. The appropriate treatment is:

A. Skin traction

B. Bed rest awaiting natural healing

C. Open reduction and internal fixation

D. Bone traction

E. Total hip replacement

175. Male, 20 years old, right upper arm laceration caused by a knife, resulting in difficulty with thumb opposition and flexion dysfunction of the thumb and index and middle fingers. The diagnosis should consider:

A. Median nerve injury

B. Ulnar nerve injury

C. Radial nerve injury

D. Musculocutaneous nerve injury

E. Axillary nerve injury

176. Male, 20 years old, right upper arm laceration caused by a knife, resulting in difficulty with thumb opposition and flexion dysfunction of the thumb and index and middle fingers. If nerve function recovery is poor, the long-term deformity of the right hand may be:

A. Claw hand deformity

B. Ape hand deformity

C. Spatula deformity

D. Swan-neck deformity

E. Mallet finger deformity

177. Male, 20 years old, right upper arm laceration caused by a knife, resulting in difficulty with thumb opposition and flexion dysfunction of the thumb and index and middle fingers. The patient was only treated with wound debridement and

suture. He returned to the hospital 2.5 years later. The appropriate treatment method is:

A. Nerve exploration and repair

B. Nerve grafting

C. Tendon transfer for functional reconstruction

D. Continued observation

E. Nerve release and decompression

178. Male, 30 years old, working as a porter at a train station for seven years. One day ago, he developed sudden low back pain while moving goods, with restricted movement and radiating pain to the right calf without bladder or bowel dysfunction. Based on the patient's symptoms, the first consideration should be:

A. Lumbar tumor

B. Acute lumbar sprain

C. Lumbar muscle strain

D. Lumbar disc herniation

E. Third lumbar transverse process syndrome

179. Male, 30 years old, working as a porter at a train station for seven years. One day ago, he developed sudden low back pain while moving goods, with restricted movement and radiating pain to the right calf without bladder or bowel dysfunction. Physical examination showed tenderness at L4-L5 spinous processes, positive straight leg raising test, positive enhancement test, decreased sensation in the lateral calf, and weakened dorsiflexion of the toes. If diagnosed with lumbar disc herniation, the nerve root involved is:

A. L2

B. L3

C. L4

D. L5

E. S1

180. Male, 30 years old, working as a porter at a train station for seven years. One day ago, he developed sudden low back pain while moving goods, with restricted movement and radiating pain to the right calf without bladder or bowel dysfunction. The most helpful examination for determining the intervertebral space and differential diagnosis is:

A. Lumbar X-ray

B. Lumbar myelography

C. Electromyography

D. Lumbar MRI

E. Radionuclide scan

181. Male, 30 years old, working as a porter at a train station for seven years. One day ago, he developed sudden low back pain while moving goods, with restricted movement and radiating pain to the right calf without bladder or bowel dysfunction. The most reasonable treatment method is:

A. Immediate surgical removal of the L4-L5 intervertebral disc

B. Bed rest on a hardboard bed for 2-4 weeks, lumbar and back muscle exercises, local physical therapy

C. Use of non-steroidal anti-inflammatory drugs

D. Standard conservative treatment for one course, if symptoms do not significantly improve, then perform lumbar disc removal surgery

E. Use of neurotrophic drugs

182. Male, 41 years old, collided with a car while riding a bicycle and fell, landing on his right hand, resulting in wrist swelling and pain with limited movement. Preliminary diagnosis of scaphoid fracture was made. The physician mainly based the preliminary diagnosis on which physical examination finding:

A. Radial side wrist swelling

B. Limited radial deviation of the wrist joint

C. Limited wrist flexion and extension

D. Wrist pain upon longitudinal percussion of the third metacarpal head

E. Significant tenderness in the anatomical snuffbox

183. Male, 41 years old, collided with a car while riding a bicycle and fell, landing on his right hand, resulting in wrist swelling and pain with limited movement. Preliminary diagnosis of scaphoid fracture was made. If no obvious abnormality is seen on the anteroposterior and lateral wrist X-rays, which of the following examinations is the simplest and most suitable for confirming the diagnosis:

A. Layered X-ray of the wrist joint

B. Scaphoid axial X-ray

C. Wrist joint CT

D. Radionuclide bone scan

E. MRI

184. Male, 41 years old, collided with a car while riding a bicycle and fell, landing on his right hand, resulting in wrist swelling and pain with limited movement. Preliminary diagnosis of scaphoid fracture was made. The appropriate treatment after confirming the diagnosis is:

A. Open reduction and internal fixation with compression screws

B. Open reduction and internal fixation with bone graft

C. No fixation needed, local application of anti-inflammatory drugs

D. Thumb spica cast

E. Wrist splint

185. Female, 40 years old, suffered right hip pain and inability to move for six hours after a sudden brake in a car accident while sitting cross-legged, with the right knee hitting the dashboard. Physical examination revealed shortening, flexion, adduction, and internal rotation deformity of the right hip. The first consideration should be:

A. Anterior hip dislocation

B. Posterior hip dislocation

C. Central hip dislocation

D. Hip dislocation with knee injury

E. Femoral neck fracture

186. Female, 40 years old, suffered right hip pain and inability to move for six hours after a sudden brake in a car accident while sitting cross-legged, with the right knee hitting the dashboard. Physical examination revealed shortening, flexion, adduction, and internal rotation deformity of the right hip. The first examination to be performed is:

A. Lateral hip X-ray

B. Anteroposterior hip X-ray

C. Anteroposterior and lateral bilateral hip X-ray

D. Anteroposterior and lateral hip X-ray

E. MRI

F. CT

187. Female, 40 years old, suffered right hip pain and inability to move for six hours after a sudden brake in a car accident while sitting cross-legged, with the right knee hitting the dashboard. Physical examination revealed shortening, flexion, adduction, and internal rotation deformity of the right hip. If no obvious fracture is seen on X-ray, the appropriate treatment is:

A. Bone traction for 4 weeks

B. Open reduction and plaster splint fixation

C. Simple manual reduction

D. Manual reduction followed by 4 weeks of bed rest

E. Manual reduction followed by 2-3 weeks of bone traction

F. Manual reduction under anesthesia

188. Female, 40 years old, suffered right hip pain and inability to move for six hours after a sudden brake in a car accident while sitting cross-legged, with the right knee hitting the dashboard. Physical examination revealed shortening, flexion, adduction, and internal rotation deformity of the right hip. After reduction and fixation for 4 weeks, the patient can start weight-bearing activities using crutches. The time for full weight-bearing is:

A. 1 month

B. 2 months

C. 3 months

D. 4 months

E. 5 months

F. 6 months

189. Female, 65 years old, admitted with "right knee swelling and pain for one year, worsened for two days." Physical examination showed significant swelling of the right knee without obvious local warmth, limited flexion and extension. Right knee X-ray revealed bone destruction near the joint surface of the distal femur. The patient had a history of breast cancer and underwent radical mastectomy six months ago. The most likely diagnosis is:

A. Giant cell tumor of bone

B. Bone metastasis after breast cancer surgery

C. Osteomyelitis

D. Bone tuberculosis

E. Osteosarcoma

F. Osteochondroma

190. Female, 65 years old, admitted with "right knee swelling and pain for one year, worsened for two days." Physical examination showed significant swelling of the right knee without obvious local warmth, limited flexion and extension. Right knee X-ray revealed bone destruction near the joint surface of the distal femur. The patient had a history of breast cancer and underwent radical mastectomy six months ago. The correct statement about this disease is:

A. It is a localized bone tumor-like lesion, not a true cyst

B. It is common in adolescents and middle-aged people

C. Pathology shows dense fibrous tissue

D. Imaging shows expanded bone with thinning cortical bone and ground-glass appearance

E. Treatment mainly involves curettage and bone grafting, with a low recurrence rate

F. Imaging shows lytic, sclerotic, or mixed types

191. Female, 65 years old, admitted with "right knee swelling and pain for one year, worsened for two days." Physical examination showed significant swelling of the right knee without obvious local warmth, limited flexion and extension. Right knee X-ray revealed bone destruction near the joint surface of the distal femur. The patient had a history of breast cancer and underwent radical mastectomy six months ago. To confirm the diagnosis, the necessary examinations are:

A. Serum alkaline phosphatase

B. Radionuclide bone scan

C. CT scan

D. Local biopsy

E. Breast-related examinations

F. All of the above

192. Female, 65 years old, admitted with "right knee swelling and pain for one year, worsened for two days." Physical examination showed significant swelling of the right knee without obvious local warmth, limited flexion and extension. Right knee X-ray revealed bone destruction near the joint surface of the distal femur. The patient had a history of breast cancer and underwent radical mastectomy six months ago. The main difference between osteosarcoma and this patient's X-ray findings is:

A. Sunburst appearance

B. Onion-skin periosteal reaction

C. Cotton-like tumor bone

D. Eccentric, lytic, and cystic destruction

E. Central, lytic destruction

F. Soap bubble appearance

193. Female, 65 years old, admitted with "right knee swelling and pain for one year, worsened for two days." Physical examination showed significant swelling of the right knee without obvious local warmth, limited flexion and extension. Right knee X-ray revealed bone destruction near the joint surface of the distal femur. The patient had a history of breast cancer and underwent radical mastectomy six months ago. Common sites for bone metast

asis from breast cancer do not include:

A. Thoracic spine

B. Elbow joint

C. Femur

D. Lumbar spine

E. Wrist joint

F. Sternum

194. Female, 65 years old, admitted with "right knee swelling and pain for one year, worsened for two days." Physical examination showed significant swelling of the right knee without obvious local warmth, limited flexion and extension. Right knee X-ray revealed bone destruction near the joint surface of the distal femur. The patient had a history of breast cancer and underwent radical mastectomy six months ago. The common pathways for bone metastasis from breast cancer include:

A. Entering the systemic circulation after lung metastasis

B. Thoracic duct

C. Lymphatic vessels

D. Intercostal veins

E. Vertebral veins

F. Intercostal arteries

195. Female, 65 years old, admitted with "right knee swelling and pain for one year, worsened for two days." Physical examination showed significant swelling of the right knee without obvious local warmth, limited flexion and extension. Right knee X-ray revealed bone destruction near the joint surface of the distal femur. The patient had a history of breast cancer and underwent radical mastectomy six months ago. The most reasonable treatment measures are:

A. Chemotherapy

B. Endocrine therapy

C. Biological therapy

D. Bisphosphonate therapy

E. Surgery

F. Radiotherapy

196. Female, 65 years old, admitted with "right knee swelling and pain for one year, worsened for two days." Physical examination showed significant swelling of the right knee without obvious local warmth, limited flexion and extension. Right knee X-ray revealed bone destruction near the joint surface of the distal femur. The patient had a history of breast cancer and underwent radical mastectomy six months ago. The patient did not seek timely treatment, and two months later, the swelling significantly worsened, with severe night pain and inability to sleep, and significantly restricted right knee movement. The primary treatment measure is:

A. Immediate surgery

B. Oral weak opioids, intramuscular morphine if necessary

C. Non-steroidal anti-inflammatory drugs

D. Nerve block therapy

E. Chemotherapy or radiotherapy

F. None of the above

197. Female, 65 years old, admitted with "right knee swelling and pain for one year, worsened for two days." Physical examination showed significant swelling of the right knee without obvious local warmth, limited flexion and extension. Right knee X-ray revealed bone destruction near the joint surface of the distal femur. The patient had a history of breast cancer and underwent radical mastectomy six months ago. MRI revealed tumor invasion of the posterior tibial nerve and vessels, with no metastatic lesions on radionuclide scan. The treatment goal is:

A. Pain relief

B. Improve quality of life

C. Prevent and treat fractures

D. Control tumor progression

E. Complete resection of the lesion

F. Prolong survival

198. Female, 65 years old, admitted with "right knee swelling and pain for one year, worsened for two days." Physical examination showed significant swelling of the right knee without obvious local warmth, limited flexion and extension. Right knee X-ray revealed bone destruction near the joint surface of the distal femur. The patient had a history of breast cancer and underwent radical mastectomy six months ago. Correct statements about the clinical features of bone metastases are:

A. The incidence of bone metastasis from breast cancer is 65%-75%

B. It can present as cancer pain, pathological fractures, spinal cord compression

C. Metastatic cancer releases soluble mediators, activating osteoclasts and osteoblasts, promoting proliferation and bone destruction at the metastatic site

D. There is currently no effective cure

E. Only osteolytic changes occur

F. About 10% of patients may develop pathological fractures

199. Child, female, 4 years old, presented to the outpatient department. Family reported unsteady walking since birth, but it was not given much attention. Physical examination showed unequal leg lengths, with the left leg about 1 cm shorter than the right, restricted active and passive movement of the left hip joint. Outpatient X-ray revealed left hip dislocation. Correct statements about congenital hip dislocation are:

A. Right side is more common than left, bilateral is less common than unilateral

B. Right side is more common than left, unilateral is more common than bilateral

C. Left side is more common than right, bilateral is less common than unilateral

D. Left side is more common than right, bilateral is more common than unilateral

E. The incidence is equal on both sides

F. More common in males than females

200. Child, female, 4 years old, presented to the outpatient department. Family reported unsteady walking since birth, but it was not given much attention. Physical examination showed unequal leg lengths, with the left leg about 1 cm shorter than the right, restricted active and passive movement of the left hip joint. Outpatient X-ray revealed left hip dislocation. Factors unrelated to the development of congenital hip dislocation include:

A. Genetic factors

B. Trauma

C. Primary acetabular dysplasia

D. Joint capsule and ligament laxity

E. Mechanical factors

F. Infection factors

201. Child, female, 4 years old, presented to the outpatient department. Family reported unsteady walking since birth, but it was not given much attention. Physical examination showed unequal leg lengths, with the left leg about 1 cm shorter than the right, restricted active and passive movement of the left hip joint. Outpatient X-ray revealed left hip dislocation. Simple congenital hip dislocation does not include:

A. Acetabular dysplasia

B. Hip subluxation

C. Hip dislocation

D. Deformity-type hip dislocation

E. Hip instability

F. Hip dislocation with the femoral head located on the iliac wing

202. Child, female, 4 years old, presented to the outpatient department. Family reported unsteady walking since birth, but it was not given much attention. Physical examination showed unequal leg lengths, with the left leg about 1 cm shorter than the right, restricted active and passive movement of the left hip joint. Outpatient X-ray revealed left hip dislocation. Symptoms not indicative of hip dislocation in newborns and infants include:

A. Asymmetrical skin folds on the thighs, buttocks, and popliteal fossa, with shortened and slightly externally rotated affected limb

B. Decreased femoral pulse

C. Limited external rotation with hip flexion to 90 degrees

D. Uncooperative child examination of both lower limbs

E. Lower limb movement disorders

F. Limited hip abduction

203. Child, female, 4 years old, presented to the outpatient department. Family reported unsteady walking since birth, but it was not given much attention. Physical examination showed unequal leg lengths, with the left leg about 1 cm shorter than the right, restricted active and passive movement of the left hip joint. Outpatient X-ray revealed left hip dislocation. Examinations not used for hip dislocation include:

A. Allis sign

B. Ortolani sign

C. Barlow test

D. Drawer test

E. Dupuytren's sign

F. Bilateral hip abduction test

204. Child, female, 4 years old, presented to the outpatient department. Family reported unsteady walking since birth, but it was not given much attention. Physical examination showed unequal leg lengths, with the left leg about 1 cm shorter than the right, restricted active and passive movement of the left hip joint. Outpatient X-ray revealed left hip dislocation. The normal location of the femoral head in the Perkin quadrants is:

A. Inner lower quadrant

B. Outer lower quadrant

C. Outer upper quadrant

D. Inner upper quadrant

E. Midpoint

F. Junction of outer upper and outer lower quadrants

205. Child, female, 4 years old, presented to the outpatient department. Family reported unsteady walking since birth, but it was not given much attention. Physical examination showed unequal leg lengths, with the left leg about 1 cm shorter than the right, restricted active and passive movement of the left hip joint. Outpatient X-ray revealed left hip dislocation. The normal acetabular index for infants is:

A. 10°-15°

B. About 15°

C. 15°-20°

D. 20°-25°

E. 25°-30°

F. 30°-35°

206. Child, female, 4 years old, presented to the outpatient department. Family reported unsteady walking since birth, but it was not given much attention. Physical examination showed unequal leg lengths, with the left leg about 1 cm shorter than the right, restricted active and passive movement of the left hip joint. Outpatient X-ray revealed left hip dislocation. Pelvic anteroposterior X-ray measurements not used for diagnosing hip dislocation include:

A. CE angle

B. Carrying angle

C. Shenton line

D. Sharp angle

E. Acetabular angle

F. Calve line

207. A 4-year-old female child visited our outpatient clinic. The patient's family reported that the patient had unstable walking since infancy, but it was not given much attention. Physical examination showed unequal leg lengths, with the left leg being about 1 cm shorter than the other side. Both active and passive movements of the left hip joint were restricted. Outpatient X-ray indicated dislocation of the left hip joint.

Question: Common surgical methods for hip dislocation do not include:

A. Salter pelvic osteotomy

B. Pemberton periacetabular osteotomy

C. Frog plaster external fixation

D. Chiari pelvic osteotomy

E. Open reduction and capsuloplasty

F. Acetabular roof augmentation

208. A 21-year-old male patient accidentally fell from a height of 3 meters, landing on both feet, resulting in swelling and pain in both heels, and back pain that prevented him from standing. Physical examination showed tenderness and percussion pain at the first lumbar vertebra, swelling and tenderness in both heels with a palpable crepitus, normal sensation in both lower limbs, normal extension of the big toes and toes, and normal flexion and extension of both knees.

Question: The correct method of transportation is:

A. One person lifts the head with one hand, while the other hand holds the legs, placing the patient on a stretcher

B. One person lifts the head, another lifts the feet, placing the patient on a wooden board

C. Two people support the upper limbs, walking the patient onto the wooden board

D. Two people roll the body as a whole onto the wooden board

E. Three people lift the head and both legs separately, placing the patient on a stretcher

F. All of the above

209. A 21-year-old male patient accidentally fell from a height of 3 meters, landing on both feet, resulting in swelling and pain in both heels, and back pain that prevented him from standing. Physical examination showed tenderness and percussion pain at the first lumbar vertebra, swelling and tenderness in both heels with a palpable crepitus, normal sensation in both lower limbs, normal extension of the big toes and toes, and normal flexion and extension of both knees.

Question: If the patient experiences abdominal distension, abdominal pain, and constipation after the injury, it may be due to:

A. Kidney injury

B. Rectal injury

C. Bladder injury

D. Urethral injury

E. Retroperitoneal hematoma stimulation

F. Intestinal perforation

210. A 21-year-old male patient accidentally fell from a height of 3 meters, landing on both feet, resulting in swelling and pain in both heels, and back pain that prevented him from standing. Physical examination showed tenderness and percussion pain at the first lumbar vertebra, swelling and tenderness in both heels with a palpable crepitus, normal sensation in both lower limbs, normal extension of the big toes and toes, and normal flexion and extension of both knees.

Question: To confirm the diagnosis, the necessary examination is:

A. X-ray of both feet

B. X-ray of the lumbar spine

C. CT of the lumbar spine

D. MRI of the lumbar spine

E. Whole-body bone scan

F. Needle biopsy

211. A 21-year-old male patient accidentally fell from a height of 3 meters, landing on both feet, resulting in swelling and pain in both heels, and back pain that prevented him from standing. Physical examination showed tenderness and percussion pain at the first lumbar vertebra, swelling and tenderness in both heels with a palpable crepitus, normal sensation in both lower limbs, normal extension of the big toes and toes, and normal flexion and extension of both knees.

Question: The most likely diagnosis for this patient is:

A. Compression fracture of the first lumbar vertebra with bilateral calcaneal fractures

B. Burst fracture of the first lumbar vertebra

C. Fracture-dislocation of the first lumbar vertebra

D. Fracture of the first lumbar vertebra with kyphotic deformity

E. Fracture of the first lumbar vertebra with spinal canal stenosis

F. Old fracture of the first lumbar vertebra

212. A 21-year-old male patient accidentally fell from a height of 3 meters, landing on both feet, resulting in swelling and pain in both heels, and back pain that prevented him from standing. Physical examination showed tenderness and percussion pain at the first lumbar vertebra, swelling and tenderness in both heels with a palpable crepitus, normal sensation in both lower limbs, normal extension of the big toes and toes, and normal flexion and extension of both knees.

Question: The correct treatment for this patient is:

A. Conservative treatment with 3 months of bed rest

B. Posterior approach with pedicle screw fixation and decompression, followed by bone graft fusion

C. Lateral anterior approach with lumbar vertebral body removal, interbody bone graft fusion, and vertebral screw fixation

D. Posterior approach with laminectomy decompression and pedicle screw fixation

E. Posterior approach with laminectomy decompression

F. All of the above

213. A 21-year-old male patient accidentally fell from a height of 3 meters, landing on both feet, resulting in swelling and pain in both heels, and back pain that prevented him from standing. Physical examination showed tenderness and percussion pain at the first lumbar vertebra, swelling and tenderness in both heels with a palpable crepitus, normal sensation in both lower limbs, normal extension of the big toes and toes, and normal flexion and extension of both knees.

Question: Regarding the indications for spinal surgery in thoracolumbar fractures, the correct statements are:

A. When there is a fracture fragment compressing the spinal cord, such as depressed lamina fracture compressing the spinal cord, laminectomy decompression is needed; if the spinal cord is compressed from the front by a vertebral body fracture, lateral anterior decompression is needed

B. In patients with complete paraplegia where the spinal cord is not believed to be transected but completely injured, or with severe incomplete paraplegia, spinal exploration and treatment are planned

C. In patients with severe fracture-dislocation of the lumbar spine and complete paraplegia, suspected cauda equina transection, planned surgical repair and suture

D. In patients with incomplete paraplegia accompanied by severe nerve root pain, indicating nerve root compression or progressively worsening neurological symptoms

E. In patients without neurological symptoms

F. All of the above are suitable

214. A 21-year-old male patient accidentally fell from a height of 3 meters, landing on both feet, resulting in swelling and pain in both heels, and back pain that prevented him from standing. Physical examination showed tenderness and percussion pain at the first lumbar vertebra, swelling and tenderness in both heels with a palpable crepitus, normal sensation in both lower limbs, normal extension of the big toes and toes, and normal flexion and extension of both knees.

Question: Common complications of thoracolumbar fractures include:

A. Spinal cord injury

B. Cauda equina nerve injury

C. Pressure sores

D. Urinary tract infection

E. Pulmonary infection

F. Lower limb venous thrombosis

215. A 21-year-old male patient accidentally fell from a height of 3 meters, landing on both feet, resulting in swelling and pain in both heels, and back pain that prevented him from standing. Physical examination showed tenderness and percussion pain at the first lumbar vertebra, swelling and tenderness in both heels with a palpable crepitus, normal sensation in both lower limbs, normal extension of the big toes and toes, and normal flexion and extension of both knees.

Question: According to the Frankel classification, the correct descriptions of spinal cord injury classification are:

A. Complete injury: No sensation or motor function preserved in the sacral segments S4-S5

B. Incomplete injury: Sensory function below the neurological level, including the sacral segments, is present but no motor function

C. Incomplete injury: Motor function is preserved below the neurological level, with the majority of key muscles having muscle strength less than grade 3

D. Incomplete injury: Motor function is preserved below the neurological level, with the majority of key muscles having muscle strength greater than or equal to grade 3

E. Normal: Normal sensory and motor function, including bowel and bladder function

F. Complete loss of motor and sensory function below the neurological level can be considered as complete injury

216. A 21-year-old male patient accidentally fell from a height of 3 meters, landing on both feet, resulting in swelling and pain in both heels, and back pain that prevented him from standing. Physical examination showed tenderness and percussion pain at the first lumbar vertebra, swelling and tenderness in both heels with a palpable crepitus, normal sensation in both lower limbs, normal extension of the big toes and toes, and normal flexion and extension of both knees.

Question: Regarding the clinical types of spinal cord injury, the correct statements are:

A. Complete paraplegia: No sensation or motor function preserved in the sacral segments S4-S5

B. Hemicord injury: Unilateral spinal cord injury with loss of proprioception and motor function on the same side, and loss of pain and temperature sensation on the opposite side; mostly incomplete injuries except for sharp injuries

C. Central cord syndrome: Almost exclusively occurs in cervical spinal cord injuries, with incomplete quadriplegia affecting both sensation and motor function, sparing sacral sensation; upper limb paralysis is worse than lower limb paralysis, with hand function most severely affected

D. Anterior cord syndrome: Injury to most of the anterior spinal cord with preservation of the posterior column, retaining proprioception but losing pain, temperature, and motor function

E. Conus medullaris syndrome: Injury to the sacral spinal cord

, where the conus medullaris coexists with the sacral nerve roots; injury to both the conus and nerve roots causes loss or impairment of lower limb sensation and motor function, bladder and bowel dysfunction; isolated conus injury preserves lower limb sensation and motor function but causes sacral and perineal sensory impairment, bladder and bowel dysfunction, loss of anal and bulbocavernosus reflexes

F. Cauda equina syndrome: Injury below the second lumbar vertebra affecting the cauda equina, causing partial or complete loss of lower limb sensation and motor function, bladder and bowel function, with possible asymmetrical symptoms

217. A 21-year-old male patient accidentally fell from a height of 3 meters, landing on both feet, resulting in swelling and pain in both heels, and back pain that prevented him from standing. Physical examination showed tenderness and percussion pain at the first lumbar vertebra, swelling and tenderness in both heels with a palpable crepitus, normal sensation in both lower limbs, normal extension of the big toes and toes, and normal flexion and extension of both knees.

Question: The correct principles for treating spinal cord injury include:

A. Early treatment of spinal cord injury

B. Reduction of spinal fractures and dislocations

C. Treatment of spinal cord injury

D. Prevention and treatment of complications

E. Functional reconstruction and rehabilitation

F. Prioritizing stabilization of vital signs and early decompression of neural structures

This is a multiple-choice question. There is only one correct answer among a, b, c, d, and e. Please choose the correct option.

1. Regarding the clinical features of osteosarcoma, which of the following is incorrect?

A. Originates from osteogenic mesenchymal tissue and is characterized by tumor cells directly forming osteoid or bone tissue

B. Mostly occurs in the most active growing areas of the epiphysis, such as the distal femur, tibia, fibula, and proximal humerus

C. Can occur at any age, but is more common in males aged 10-27

D. Almost all metastases are through lymphatic spread

E. Pain is an early symptom, initially intermittent and later persistent and severe, especially at night

2. For a patient with a fibular neck fracture caused by trauma, who cannot dorsiflex the foot post-injury, the reason is:

A. Sciatic nerve injury

B. Anterior tibial muscle injury

C. Posterior tibial muscle injury

D. Common peroneal nerve injury

E. Posterior tibial nerve injury

3. Which of the following does not conform to the diagnostic criteria for chronic osteomyelitis?

A. Recurrent episodes

B. Shortening of the affected limb compared to the healthy limb

C. Long-term non-healing sinus tract

D. Sequestrum discharge from the sinus tract

E. Thin skin on the affected limb with darkened color

4. Spinal tuberculosis most commonly occurs in the:

A. Transverse process

B. Vertebral body

C. Spinous process

D. Articular process

E. Lamina

5. A fracture occurring at the junction of the cortical and cancellous bone at the metaphysis of long tubular bones, with the heel landing centrally while standing, most likely results in which type of displacement?

A. Angular displacement

B. Lateral displacement

C. Shortening displacement

D. Separation displacement

E. Rotational displacement

6. A medical accident causing mild disability, tissue, and organ damage leading to general functional impairment belongs to:

A. Special grade medical accident

B. First grade medical accident

C. Second grade medical accident

D. Third grade medical accident

E. Fourth grade medical accident

7. After clinical healing of a fracture, the remodeling and shaping of the callus depend on:

A. Stability of external fixation

B. Stress from limb activity and weight-bearing

C. Local blood supply condition

D. Amount of callus

E. Whether treatment cooperation is followed

8. The purpose of proper fixation in fracture first aid management is incorrect in which of the following?

A. To avoid movement of the fracture ends

B. To prevent worsening of soft tissue injury during transportation

C. Pain relief to prevent shock

D. Facilitates transportation

E. Reduces bleeding at the fracture site

9. Regarding the treatment of congenital muscular torticollis, which of the following is incorrect?

A. Non-surgical treatment is suitable for infants under one year old, including hot compresses, massage, manual correction, and orthopedic cap fixation

B. Surgical treatment is suitable for children over one year old, with the ideal surgery age being 1-4 years

C. Surgery often involves the cutting of the sternocleidomastoid muscle

D. Postoperative head placement in an over-corrected position with head, neck, and chest plaster fixation for four weeks

E. Surgical treatment is absolutely contraindicated for those over 12 years old

10. Regarding the surgical indications for finger replantation, which of the following is incorrect?

A. Thumb function is most important and should be replanted at all costs

B. Replantation should be attempted for fingers severed in two or more places

C. For young individuals, replantation should be attempted whenever possible

D. For children, replantation should be sought

E. For the elderly, decisions on replantation should be made cautiously

11. Concerning tendon adhesion release surgery, which of the following statements is incorrect?

A. Generally performed 3-6 months post-surgery

B. Completely expose the previous surgical area and thoroughly remove scar tissue

C. Use sharp dissection to separate adhesions without tendon stripping

D. The more thorough the release, the better

E. Begin finger flexion and extension exercises one week post-surgery

12. Regarding the relationship between spinal trauma and spinal cord injury, which of the following is incorrect?

A. The segment of spinal cord injury does not correspond with the level of vertebral injury

B. Thoracic vertebrae are relatively fixed, and thoracic dislocation often does not cause spinal cord injury

C. Some cases exhibit significant spinal cord injury, but X-ray examination shows no fractures or dislocations

D. Flexion-type fracture dislocations are the most common cause of spinal cord injury

E. Spinal fractures are more likely to cause spinal cord injury in those with spinal canal stenosis

13. Aneurysmal bone cysts of the spine most commonly occur in:

A. Vertebral body

B. Spinal canal

C. Accessory structures

D. Paravertebral region

E. Intervertebral foramen

14. Characteristics of the doctor-patient relationship include:

A. Related interests and unity in value realization

B. Equality of personal rights and asymmetry of medical knowledge

C. Asymmetry in choice and emotional neutrality

D. Sensitivity and inevitability of doctor-patient conflicts

E. All of the above

15. A fibular head fracture is most likely to cause which of the following injuries?

A. Posterior tibial artery injury

B. Deep peroneal nerve injury

C. Common peroneal nerve injury

D. Superficial peroneal nerve injury

E. Anterior tibial artery injury

16. The examination that can help diagnose chronic anterior cruciate ligament (ACL) rupture of the knee is:

A. Squatting walking test

B. Knee X-ray examination

C. Knee varus and valgus stress test

D. Grinding test

E. Drawer test

17. A patient with a C6 fracture dislocation and quadriplegia, with slight quadriceps contraction but no knee joint activity, has muscle strength graded as:

A. 1

B. 2

C. 3

D. 4

E. 5

18. The most basic and important treatment for open fractures is:

A. Early reduction and internal fixation followed by wound closure

B. Early reduction and external fixation followed by wound closure

C. Early thorough debridement and wound closure, with antibiotics to prevent infection

D. Early reduction and fixation, with antibiotics to prevent infection

E. Early thorough debridement and appropriate fixation

19. The occurrence of gout is related to:

A. Amino acid metabolism disorder

B. Protein metabolism disorder

C. Nucleic acid metabolism disorder

D. Lipid metabolism disorder

E. Carbohydrate metabolism disorder

20. The reason that no external callus forms during the healing of femoral neck fractures is:

A. Excessive hematoma after femoral neck fracture

B. Femoral neck lacks periosteum

C. Femoral neck lacks endosteum

D. Poor blood supply to the femoral neck

E. Severe injury to the femoral neck making callus formation difficult

21. The best early treatment for a patient with septic arthritis of the knee is:

A. Early effective antibiotics and supportive therapy

B. Early effective antibiotics and early joint incision and drainage

C. Early effective antibiotics and functional exercises with physiotherapy

D. Early effective antibiotics and joint puncture for fluid aspiration and antibiotic injection

E. Early effective antibiotics and plaster immobilization

22. The diagnostic criteria for frozen shoulder include:

A. More common in males than females

B. More common on the right side than the left

C. Shoulder pain unrelated to movement

D. Limited shoulder abduction, external rotation, and extension

E. No deltoid muscle atrophy

23. The most important diagnostic criterion for cervical spondylotic myelopathy is:

A. Eye pain and abnormal facial sweating

B. Headache and dizziness

C. Numbness in both upper limbs

D. Cold limbs with little or no sweating

E. Numbness and weakness in all four limbs, with positive pathological reflexes

24. The most common site for osteosarcoma is:

A. Distal femur

B. Distal tibia

C. Proximal humerus

D. Upper fibula

E. Spine

25. Which of the following is not a factor causing meniscal injury?

A. Knee semi-flexion

B. Knee adduction or abduction

C. Standing

D. Rotation

E. Gravity compression

26. The best early treatment for acute purulent hip arthritis is:

A. Effective antibiotics and intra-articular antibiotic injection

B. Effective antibiotics and early functional exercises and physiotherapy

C. Effective antibiotics and plaster immobilization

D. Effective antibiotics and joint incision and drainage

E. Effective antibiotics and supportive therapy

27. A positive oblique test indicates:

A. Sacroiliac joint disease

B. Hip joint disease

C. Sacroiliac joint disease

D. Sciatic nerve disease

E. Lumbar spine disease

28. In the emergency treatment of severe hand trauma patients, the first thing to understand is:

A. Hand function

B. Degree of nerve injury

C. Degree of tendon injury

D. Degree of bone and joint injury

E. Blood circulation status

29. The surgical goal for old spinal cord injuries is:

A. Smooth the spinal canal and restore cerebrospinal fluid circulation

B. Improve blood supply to the spinal cord

C. Improve appearance

D. Relieve spinal

cord compression

E. Restore spinal stability and relieve spinal cord compression

30. The most important aspect of preventing bone and joint tuberculosis is:

A. Prevention and control of respiratory and gastrointestinal tuberculosis

B. Prevention of bone and joint injuries

C. Regular orthopedic examinations

D. Taking anti-tuberculosis drugs

E. Strict isolation of patients with bone and joint tuberculosis

31. The cause of osteoarthritis is:

A. Genetic factors

B. Sex hormones

C. Mechanical factors

D. Obesity

E. All of the above

32. An important manifestation of hip joint tuberculosis is:

A. Low fever

B. Emaciation

C. Pain in the affected limb

D. Deformity of the affected limb

E. Shortening of the affected limb

33. The pathological changes in chondromalacia patellae first occur in:

A. Deep layers of cartilage

B. Surface layers of cartilage

C. Margins of cartilage

D. Synovium

E. Meniscus

34. The main signs of lumbar disc herniation do not include:

A. Restricted lumbar movement and limping, changes in lumbar lordosis

B. Local tenderness, percussion pain, and radiating pain

C. Positive straight leg raise, neck flexion, and supine bridge tests

D. Changes in lower limb sensation, muscle strength, and reflexes

E. Lower limb edema, itching, pigmentation, and ulcers

35. When pregnancy threatens the life of the mother, allowing abortion or induction conforms to:

A. Principle of life value

B. Principle of optimization

C. Principle of non-maleficence

D. Principle of respect

E. Principle of justice

36. A stable type of femoral neck fracture is:

A. Subcapital fracture

B. Transcervical fracture

C. Basal fracture

D. Adduction fracture

E. Abduction fracture

37. Which of the following statements about bone metastases is incorrect?

A. Bone metastases are 35-40 times more common than primary malignant bone tumors

B. Both carcinoma and sarcoma can metastasize, but carcinoma metastasis to bone is more common

C. Bone metastases on imaging can appear osteoblastic, osteolytic, or mixed

D. Pathological fractures and spinal cord compression are surgical indications for bone metastases

E. The main purpose of surgery for bone metastases is to prolong the patient's life

38. The most common type of cervical disc herniation is:

A. Vertebral artery type

B. Sympathetic type

C. Nerve root type

D. Spinal cord type

E. Mixed type

39. The usual traction weight for a closed femoral shaft fracture with tibial tuberosity traction is:

A. 1/2 to 1/3 of body weight

B. 1/3 to 1/4 of body weight

C. 1/4 to 1/5 of body weight

D. 1/5 to 1/6 of body weight

E. 1/7 to 1/8 of body weight

40. The most important early diagnostic method for differentiating ankylosing spondylitis from other diseases is:

A. Anteroposterior and lateral radiographs of the lumbar spine

B. Erythrocyte sedimentation rate (ESR)

C. Blood HLA-B27 test

D. Rheumatoid factor

E. Anteroposterior and lateral radiographs of the pelvis

41. Which clinical manifestation can be diagnosed as a fracture?

A. Local swelling

B. Significant tenderness

C. Inability to move the lower limb independently

D. Bone crepitus

E. Significant limping

42. The correct position for transporting a suspected spinal fracture patient is:

A. Several people lifting flat

B. Flat back

C. Hugging

D. One person lifting the head, one person lifting the feet

E. Sitting in a wheelchair

43. Acute hematogenous osteomyelitis commonly occurs in:

A. Infants

B. Young adults

C. Children

D. Elderly

E. Middle-aged adults

44. The most typical clinical manifestation of meniscal injury is:

A. Pain

B. Swelling

C. Limping

D. Joint locking

E. Rash

45. One reason why children are prone to radial head subluxation is:

A. Shallow olecranon fossa

B. Loose joint capsule and annular ligament

C. Deep olecranon fossa

D. Large radial head

E. Contracted joint capsule and annular ligament

46. The first local treatment for simple synovial tuberculosis of the knee, in addition to general treatment, is:

A. Skin traction

B. Plaster immobilization

C. Aspiration and injection of anti-tuberculosis drugs

D. Knee arthrodesis

E. Debridement of the knee joint lesion

47. Which of the following descriptions of the main functions of the meniscus is incorrect?

A. Absorbing shock and cushioning

B. Helping to evenly distribute synovial fluid

C. Providing neural feedback and proprioception

D. Secreting synovial fluid to nourish joint cartilage

E. Participating in maintaining joint stability

48. If pelvic fracture with retroperitoneal hemorrhagic shock does not improve after active resuscitation, immediate action should be:

A. DSA embolization of the common iliac artery

B. DSA embolization of the internal iliac artery

C. DSA embolization of the external iliac artery

D. Internal DSA embolization of the external iliac artery

E. DSA embolization of the internal and external iliac arteries and veins

49. The best time for early debridement and suturing after hand trauma is:

A. 6-8 hours

B. 8-12 hours

C. 12-16 hours

D. 16-24 hours

E. Within 48 hours

50. Which of the following tests, if positive, can confirm congenital hip dislocation in a newborn?

A. Allis sign

B. Ortolani test

C. Barlow test

D. Telescoping sign

E. Trendelenburg sign

51. The appropriate ratio of artery to vein during replantation of a severed limb (finger) is:

A. 1:1

B. 1:2

C. 1:3

D. 2:1

E. 3:2

52. The treatment principle for bone cysts is:

A. Resection of the lesion segment and bone grafting

B. Thorough removal of the cyst wall and bone grafting

C. Curettage of the cyst wall and drainage of the cyst cavity fluid

D. Observation without surgery

E. Amputation

53. During the transportation of a patient with a spinal fracture, the position to be fixed is:

A. Semi-sitting position

B. Lateral position

C. Prone position

D. Supine hyperextension position

E. Flexed lateral position

54. The most distinguishing auxiliary examination between lumbar disc herniation and intramedullary tumors is:

A. X-ray examination

B. CT scan

C. MRI scan

D. Electromyography

E. Ultrasound

55. Which of the following is not part of emergency care for limb amputation?

A. Rapid transportation

B. Bandaging

C. Preservation of the amputated limb

D. Debridement

E. Hemostasis

56. Regarding septic arthritis, which statement is incorrect?

A. It commonly occurs in the hip joint

B. It can heal spontaneously

C. The causative agent is Staphylococcus aureus

D. The infection route is mostly hematogenous

E. It can lead to pathological dislocation

57. In the case of urgent patient rescue without timely medical record writing, the relevant medical staff should make up the records truthfully within how long after the rescue ends?

A. 2 hours

B. 4 hours

C. 6 hours

D. 8 hours

E. 12 hours

58. For a patient with severe destruction of the proximal interphalangeal joint of the right index finger, where future recovery of function is difficult, the joint should be fixed in:

A. Protective position

B. Resting position

C. Extended position

D. Extreme flexed position

E. Functional position

59. Flatfoot (also known as pes planus) refers to:

A. Pain on the anterior medial side of the ankle joint, worsened by prolonged standing or walking, relieved by rest

B. Swelling on the lateral side of the joint, pain at the navicular tuberosity, both heels in an outward position

C. Loss of the longitudinal arch in the footprint examination, widened midfoot

D. Low or absent medial arch, loose foot soft tissue, heel valgus

E. Loss of the arch, increased angle between the long axes of the calcaneus and talus, formation of bony bridges

60. Acute hematogenous osteomyelitis commonly occurs in:

A. Ulna and radius

B. Distal tibia and proximal humerus

C. Proximal tibia and distal femur

D. Iliac bone

E. Vertebral bone

61. The most common site of spinal fractures with spinal cord injury is:

A. Cervical spine

B. Thoracic spine

C. Lumbar spine

D. Sacral spine

E. Pelvic region

62. The most common and severe late complication of intracapsular femoral neck fractures is:

A. Shortening of the lower limb

B. Hip varus deformity

C. Avascular necrosis

of the femoral head

D. Hip subluxation

E. Traumatic arthritis

63. Which of the following is not a preventive measure for lumbar disc herniation?

A. Avoid prolonged sitting

B. Use of lumbar support

C. Regularly bending exercises

D. Strengthening back muscles

E. Regular physiotherapy, massage, and acupuncture

64. Incorrect treatment for frozen shoulder is:

A. Physiotherapy

B. Corticosteroid injection

C. Massage

D. Restriction of shoulder joint movement

E. Conservative treatment

65. Ischemic bone necrosis is most likely to occur in:

A. Lunate bone fracture

B. Scaphoid bone fracture

C. Trapezium bone fracture

D. Capitate bone fracture

E. Ulnar styloid process fracture

66. The clinical manifestations of primary osteoarthritis do not include:

A. Slow onset with joint pain as the main initial symptom

B. Some patients experience resting pain

C. Joint redness and swelling in the early stages

D. Joint pain sometimes related to weather changes

E. Some patients have various noises during joint movement

67. Regarding the clinical features of cervical spondylosis, which statement is incorrect?

A. It can have sympathetic symptoms such as tachycardia

B. The nerve root type presents as hand numbness and weakness

C. The sympathetic type is the most common

D. Severe cases can lead to paraplegia

E. Osteophytes compressing the esophagus can cause swallowing difficulties

Patient Liu, due to fever for three days, went to the county hospital for consultation. Dr. Zhang from the outpatient department examined Liu and found Koplik spots on the buccal mucosa, tentatively diagnosing measles. Because the county hospital did not have isolation treatment facilities, Dr. Zhang instructed Liu's parents to take Liu to the municipal infectious disease hospital. According to the Infectious Disease Prevention Law, Dr. Zhang should:

A. Request a consultation with a superior doctor and transfer the patient after a confirmed diagnosis

B. Request a consultation with a superior doctor, confirm the diagnosis, and provide isolation treatment

C. Report to hospital leadership, confirm the diagnosis, and isolate the patient locally

D. Report to the local disease control institution and transfer the patient with a copy of the medical records

E. Report to the local disease control institution and let the disease control institution handle the transfer

69. A male patient, 25 years old, a construction worker, sustained a left iliac bone fracture and pubic symphysis separation due to trauma. Eight hours post-injury, he had no urine, clear consciousness, and the most likely condition is:

A. Hemorrhagic anemia

B. Urethral injury

C. Unaccustomed to urinating in bed

D. Urinary tract stones

E. Bladder rupture

70. A female patient, 30 years old, was injured by a falling wall six hours ago, causing severe swelling and shining of the injured foot, pale toenails, absence of dorsalis pedis pulse, toes in semi-flexed position, and severe pain upon traction. The most likely diagnosis is:

A. Tibial nerve injury

B. Compartment syndrome

C. Common peroneal nerve injury

D. Arterial injury

E. Tendon injury

71. A male patient, 30 years old, fell from the second floor during construction work, immediately experiencing severe back pain, sensory and motor deficits in both lower limbs, and urinary and fecal incontinence. Clinical and X-ray examinations diagnosed thoracolumbar flexion-type compression fractures with spinal cord injury. The most valuable examination to determine the displacement of the fracture fragments into the spinal canal is:

A. MRI

B. CT scan

C. ECT

D. Myelography

E. X-ray tomography

72. A male patient, 28 years old, sustained a compressive fracture of L1 vertebra with paraplegia. One week post-fracture, he developed a fever, most likely due to:

A. Heat from hematoma absorption post-fracture

B. Urinary tract or respiratory infection causing fever

C. Central regulation dysfunction due to spinal cord injury

D. Autonomic nervous dysfunction

E. Low heat production due to muscle paralysis

73. A male patient, 56 years old, underwent liver transplantation for liver cancer one year ago. He fell and sustained a right hip fracture, with X-ray showing femoral neck fracture and osteolytic changes around the femoral neck. The appropriate treatment is:

A. Hip replacement surgery

B. Closed reduction and internal fixation

C. Open reduction and internal fixation

D. Amputation

E. Hip resurfacing

74. In an emergency room, a patient sustained multiple injuries from a car accident. The first attending doctor issued an urgent consultation request to the entire hospital. The emergency consultation doctor should arrive within how long after the consultation request is issued?

A. 10 minutes

B. 30 minutes

C. 12 minutes

D. 8 minutes

E. 5 minutes

75. A male patient, 18 years old, had pain and swelling in the proximal left upper arm for one month. X-ray showed an expansile cystic radiolucent area with clear borders and internal bony septa dividing the cyst into a honeycomb appearance. The most likely diagnosis is:

A. Bone cyst

B. Fibrous dysplasia

C. Aneurysmal bone cyst

D. Enchondroma

E. Osteosarcoma

76. A female patient, 16 years old, had left hip pain for two months, worsening over the past week and affecting sleep. On admission, her temperature was normal, and X-ray showed local destruction of the left iliac bone with moth-eaten appearance. Biopsy indicated a small round cell malignant bone tumor. The correct clinical treatment is:

A. Chemotherapy

B. Radiotherapy

C. Bone resection and chemotherapy

D. Bone resection and combined radiotherapy and chemotherapy

E. Segmental resection and replantation

77. A male patient, 15 years old, had high fever and swelling of the right knee for six days. Knee puncture extracted purulent fluid. The most appropriate treatment is:

A. Large doses of antibiotics

B. Antibiotics and immobilization of the affected limb

C. Systemic antibiotics, joint puncture, washing, antibiotic injection, and external fixation

D. Antibiotics and incision and drainage

E. Antibiotics and functional exercises

78. A male patient, 30 years old, with L2 vertebral fracture dislocation and cauda equina nerve injury. Examination showed quadriceps contraction with active knee extension but unable to lift off the bed. Quadriceps muscle strength is:

A. Grade 1

B. Grade 2

C. Grade 3

D. Grade 4

E. Grade 5

79. A female patient, 24 years old, fell and had shoulder pain for two hours. Physical examination showed the affected shoulder sagging, limited limb movement, and the head tilted to the affected side. Dugas sign was negative. The most likely diagnosis is:

A. Shoulder dislocation

B. Brachial plexus injury

C. Clavicle fracture

D. Surgical neck fracture of the humerus

E. Radial head subluxation

80. A boy, 7 years old, had sudden chills and high fever (T39.8℃), restlessness, and severe pain below the right knee, with the knee in a semi-flexed position, refusing movement. Physical examination revealed warmth and tenderness in the proximal right lower leg. The most likely diagnosis is:

A. Rheumatic knee arthritis

B. Suppurative knee arthritis

C. Suppurative osteomyelitis

D. Traumatic knee arthritis

E. Distal femoral osteosarcoma

81. Patient Chen, male, diagnosed with "erysipelas of the lower leg" for one week, received intravenous penicillin at a hospital. Today, he suddenly developed pillow-sized red patches and itching on the skin, diagnosed as "drug-induced dermatitis". The most critical treatment measure is:

A. Immediately mark the allergenic drug on the patient's medical record

B. Immediately administer oral or intravenous corticosteroids

C. Immediately administer oral or intramuscular antihistamines

D. Immediately discontinue penicillin

E. Immediately administer anti-allergic auxiliary drugs

82. A male patient, 22 years old, with tightness in both lower limbs, followed by weakness in holding objects and difficulty walking for three months. Physical examination showed irregular sensory reduction areas, increased muscle tone, weak muscle strength, and positive Hoffmann sign. The most likely diagnosis is:

A. Amyotrophic lateral sclerosis

B. Transverse myelitis

C. Cervical spondylotic myelopathy

D. Primary neuritis

E. Syringomyelia

83. Boy, 11 years old. Left hip pain for 3 months radiating to the knee. Physical examination: emaciation, flexion adduction deformity of the left hip joint, limited abduction and external rotation. X-ray shows narrowed joint space. The most likely diagnosis is:

A. Rheumatoid arthritis

B. Avascular necrosis of the femoral head

C. Suppurative hip arthritis

D. Transient synovitis

E. Hip tuberculosis

84. Male, with a history of long-term steroid use for skin disease, has had bilateral hip pain and limited movement for the past 2 years. The preliminary diagnosis is:

A. Bilateral rheumatoid arthritis

B. Bilateral traumatic synovitis

C. Bilateral degenerative osteoarthritis

D. Bilateral avascular necrosis of the femoral head

E. Bilateral neoplastic lesions of the hip joint

85. Dr. Hu, a physician engaged in maternal and child healthcare in the county, issued false medical certificates in violation of the "Maternal and Child Healthcare Regulations" and the circumstances were serious. The county health bureau should:

A. Issue a public criticism

B. Give a warning

C. Revoke his practice license

D. Impose a fine

E. Demote and reduce salary

86. A 3-year-old child experienced pain and refused to use the right hand after being pulled by the mother an hour ago. Physical examination: no deformity of the right upper limb, mild tenderness in the right elbow, no obvious swelling, X-ray (-). The most likely diagnosis is:

A. Elbow joint injury

B. Non-displaced supracondylar fracture of the humerus

C. Non-displaced intracondylar fracture of the humerus

D. Subluxation of the radial head

E. Muscle and tendon injury of the elbow

87. A 6-year-old boy with right clubfoot deformity, difficult to correct manually, should be treated with:

A. Muscle balancing surgery

B. McKay surgery

C. Cast correction

D. Triple arthrodesis after age 12

E. Soft tissue release surgery

88. An 8-year-old girl with a transverse fracture of the right femur, treated with single hip spica cast for 12 days, still has overlapping displacement and slight angulation on X-ray. The family requests transfer for further treatment. The most inappropriate management is:

A. Remove the cast, replace with a new cast (due to limb deflation making the original cast loose)

B. Maintain current treatment with continued cast fixation

C. Remove the cast and apply skin traction

D. Remove the cast and apply a small splint to the thigh with skin traction

E. Open reduction and internal fixation to correct overlap and angulation deformity

89. Male, 34 years old, sudden onset of low back pain 3 days ago, followed by right leg pain, aggravated by coughing. Physical examination: sacral tenderness radiating to the calf, right straight leg raise positive at 40 degrees, decreased sensation in the anterior calf and medial foot, weakness of the extensor hallucis longus muscle. The affected nerve root is:

A. L2

B. L3

C. L5

D. S1

E. L4

90. A 33-year-old male was admitted with right forearm injury and diagnosed with a Colles' fracture. After manual reduction and cast immobilization, he had limited wrist and finger flexion-extension 6 weeks later. The main cause is:

A. Possible flexor tendon injury of the fingers during fracture

B. Possible joint injury of the fingers during fracture

C. Insufficient active and passive finger exercises during cast immobilization causing joint stiffness

D. Cast-induced compression causing ischemic contracture of the hand

E. Possible median nerve injury during fracture

91. A person who passed the qualification exam and registered as a practicing physician opened a dental clinic. Due to his familiarity with obstetrics and gynecology, he also treated some gynecological and obstetric patients. This activity is considered:

A. A legal act as long as it serves patient needs

B. A legal act

C. Legal as long as no errors occur

D. Illegal as it exceeds the scope of practice

E. Allowed under physician practice regulations

92. An 8-year-old boy with a supracondylar fracture of the humerus, diagnosed locally. After two failed attempts at manual reduction, he presented with severe swelling, obvious tenderness, and weak radial pulse in the right elbow 48 hours post-injury. The diagnosis should include:

A. Extensive soft tissue contusion

B. Major venous injury

C. Brachial artery injury

D. Muscle rupture

E. Median, ulnar, and radial nerve injury

93. Male, 40 years old, wrist injury one hour ago. Physical examination: swelling of the right wrist, "dinner fork" deformity, tenderness, and limited movement. The most likely diagnosis is:

A. Right scaphoid fracture

B. Smith fracture

C. Radial nerve injury

D. Colles fracture

E. Right wrist dislocation

94. Elderly patient with a distal radius (Colles) fracture post-trauma. Good fracture alignment and impaction are noted. The treatment of choice is:

A. Traction treatment

B. Symptomatic treatment

C. Surgical treatment

D. Swelling reduction treatment

E. Manual reduction and external fixation

95. A 26-year-old male with low-grade fever and neck pain for 4 months, no history of trauma. Physical examination: limited neck rotation. X-ray lateral view shows anterior dislocation of the atlas and widening of the retropharyngeal space. The initial diagnosis is:

A. Peri-atlantal soft tissue infection

B. Atlantoaxial tuberculosis

C. Spontaneous atlantoaxial dislocation

D. Retropharyngeal abscess

E. Atlantoaxial deformity

96. A 40-year-old female with right elbow pain for half a month, weak grip, localized tenderness. The most helpful diagnostic test is:

A. Tinel's sign

B. Finkelstein test

C. Dugas' sign

D. Hoffmann's sign

E. Mills test

97. A 60-year-old female with a comminuted distal radius fracture diagnosed by X-ray 4 weeks ago. After closed reduction and cast immobilization, she had limited finger flexion-extension upon cast removal. The main cause is:

A. Combined median and ulnar nerve injury during fracture

B. Combined flexor and extensor tendon injury during fracture

C. Cast compression causing ischemic contracture of the hand

D. Insufficient active and passive finger exercises during cast immobilization causing joint stiffness

E. Combined joint injury of the hand during fracture

98. A 50-year-old sailor with a transverse fracture of the right tibia and fibula at the distal third. After alignment and splint fixation, X-ray 3 months later shows poor callus formation and non-union. The cause is:

A. Splint fixation

B. Poor blood supply to the fracture site

C. Older age

D. Impact of surrounding soft tissue injury

E. Inadequate fracture alignment

99. A 15-year-old male with left knee pain for 2 months, worsening for 1 week, with local swelling, no fever. X-ray shows osteolytic changes with scattered calcification and periosteal reaction at the distal femur, confirmed as chondrosarcoma by biopsy. The treatment is:

A. Traditional Chinese medicine

B. En bloc resection of the lesion

C. En bloc resection with bone grafting

D. Segmental resection with vascularized bone graft

E. Chemotherapy and immunotherapy

100. A 28-year-old pregnant woman in the late stage of pregnancy presented with progressive back pain, lower limb weakness, and loss of appetite. Physical examination: mild kyphosis with tenderness at T7, X-ray shows narrowed T6-7 intervertebral space and paraspinal soft tissue swelling, ESR 60 mm/h. The most likely diagnosis is:

A. Thoracic metastasis

B. Thoracic tuberculosis

C. Thoracic hemangioma

D. Pyogenic spondylitis

E. Thoracic disc herniation

101. During a hearing in the US Congress on the commercialization of genetic testing, a phone recording was released: An interviewer pretending to be a customer sought help from an unnamed genetic testing company. The "customer" asked, "Can I secretly take my fiancé's saliva and have you test it?" The company's response was shocking: "Of course we can conduct this test without your fiancé's permission." Regarding this case, the correct statement is:

A. The company does not need to respect privacy rights when handling other people's genetic data and can disclose personal information to third parties.

B. The "customer" was not wrong in secretly testing their fiancé's genes for a harmonious marriage.

C. The "customer" has the autonomy to test their fiancé's genes, but it's best to discuss it with him.

D. When using someone else's genetic data, the person's informed consent must be ensured.

E. All of the above statements are correct.

102. Male, 26 years old, left shoulder joint dislocation, fixed after manual reduction. The shoulder joint should be fixed in:

A. Adduction and internal rotation, elbow flexed 90°

B. Abduction, external rotation, elbow extended

C. Abduction and external rotation, elbow flexed 90°

D. Abduction and internal rotation, elbow flexed 90°

E. Adduction and internal rotation, elbow extended

103. 8-year-old boy, left femur distal pain and swelling for 1 week, body temperature 39.5°C, local skin temperature high, deep tenderness. Ineffective after anti-infective treatment, local puncture extracted a small amount of purulent fluid. For further treatment, the first consideration should be:

A. Combined use of high-dose antibiotics

B. Local immobilization to aid inflammation absorption

C. Systemic supportive therapy

D. Local decompression and drainage

E. Lesion clearance surgery

104. Male, 33 years old, open fracture of the left femoral shaft caused by a car accident. The first complication to be noted at admission is:

A. Nerve injury

B. Wound infection

C. Hypovolemic shock

D. Traumatic ossification

E. Ischemic necrosis

105. Male, 12 years old, weakness in the left lower limb, sometimes falls for 7 years. Physical examination: slight contraction of the left quadriceps, unable to cause knee extension activity in the right lateral position. The muscle strength of this quadriceps is:

A. Grade 0

B. Grade 1

C. Grade 2

D. Grade 3

E. Grade 4

106. Female, 45 years old, with right thumb stenosing tenosynovitis for 2 weeks. The best treatment at this time is:

A. Immobilization

B. Physiotherapy

C. Local injection of prednisolone

D. Tenosynovectomy

E. Local antibiotic injection

107. Boy, 11 years old, left hip pain for 2 months, radiating to the knee. Physical examination: emaciation, flexion adduction deformity of the left hip joint, restricted abduction and external rotation. X-ray showed narrowed joint space. The most important treatment measure to save joint function is:

A. Timely lesion clearance surgery

B. Local puncture to drain pus

C. Continuous skin traction on the affected limb to prevent joint deformity

D. Systemic drug therapy

E. Active supportive therapy

108. An 8-year-old child with left tibia and fibula midshaft fractures for 3 months. X-ray showed oblique fractures of the tibia and fibula midshaft, 60% alignment, 10° forward angulation, 1 cm overlap, and healing. The appropriate treatment is:

A. Open reduction and plate fixation

B. Open reduction and intramedullary pin fixation

C. Manual reduction and plaster fixation

D. Manual reduction and continuous traction

E. No treatment needed

109. Female, 26 years old, had appendectomy 3 years ago. Recently, she had abdominal pain and bloating for 2 days, no defecation or flatus, and an abdominal X-ray in the upright position showed isolated dilated bowel loops, with a fixed position that did not change over time. The most appropriate treatment is:

A. Infusion to replenish blood volume

B. Gastrointestinal decompression and antibiotic treatment

C. Correcting water and electrolyte disturbances

D. Rectal tube for gas release

E. Active preoperative preparation and exploratory laparotomy

110. Mr. Li wanted to apply for a leave of absence from his unit to prepare for the postgraduate entrance exam, so he asked his middle school classmate, Dr. Wang, a practicing physician at the county hospital, to write him a sick leave note. Dr. Wang issued a diagnosis certificate for "viral myocarditis, 1 month rest." For Dr. Wang's actions, the county health bureau can impose:

A. Revocation of his medical practice license

B. Warning or suspension of practice activities for 3 to 6 months, and requiring training and continuing education

C. Warning or suspension of practice activities for 6 months to 1 year

D. Transfer from the physician position

E. Administrative or disciplinary action

111. Male, 35 years old, gradual swelling on the right radial side, restricted movement. Physical examination: swelling at the distal end of the right radius, tenderness, normal skin temperature. X-ray showed eccentric expansion of the distal right radius, decreased bone density, and soap bubble appearance. The diagnosis is considered:

A. Osteosarcoma

B. Osteomyelitis

C. Osteochondroma

D. Chondrosarcoma

E. Giant cell tumor of bone

112. Male, 30 years old, right fibula shaft fracture with partial displacement from a football injury. The recommended treatment is:

A. Open reduction and internal fixation with plates and screws

B. Open reduction and intramedullary pin fixation

C. External fixation with plaster

D. Skin traction treatment

E. Bone traction treatment

113. Male, 25 years old, fell and caused right tibial fracture 2 hours ago. After manual reduction and plaster splint fixation, the initial pain was not significant but gradually increased. At this point, the treatment measure is:

A. Administration of analgesics

B. Removal and re-wrapping of the plaster splint

C. Intravenous antibiotics

D. Use of vasodilators

E. Immediate fasciotomy for decompression

114. Male, 55 years old, fell and caused right ankle fracture. After manual reduction, plaster cast fixation was applied. After the fracture healed, the cast was removed. Six months later, the right ankle was still swollen, with burning pain, low local skin temperature, and hair loss. The most likely diagnosis is:

A. Traumatic ossification

B. Deep vein thrombosis

C. Acute bone atrophy

D. Ischemic necrosis of the lateral ankle

E. Traumatic arthritis of the ankle

115. Male, 30 years old, fell from a high place 3 hours ago, causing a left femoral upper segment fracture with bone ends exposed. X-ray showed a small oblique fracture. The best treatment at this time is:

A. Debridement and suturing, splint fixation

B. Debridement, fracture reduction, intramedullary nail fixation

C. Debridement and suturing, fracture deferred for secondary treatment

D. Debridement and suturing, plaster fixation

E. Debridement and suturing, manual reduction, and splint fixation

116. Male, 70 years old, left lower limb pain after walking for 2 years, worsened for 1 month. He has a 10-year history of rheumatoid arthritis. Physical examination: normal lumbar spine curvature, normal flexion activity, positive spinal hyperextension test, decreased sensation on the left lateral calf, left long extensor muscle strength grade 4. X-ray showed slight narrowing of the L4-L5 intervertebral space, positive straight leg raising test at 35°. The most likely diagnosis is:

A. Lumbar muscle strain

B. Lumbar spine osteophyte

C. Lumbar spondylolysis

D. L4-L5 disc herniation

E. Obliterative arteritis

117. A 6-month-old boy occasionally found a left inguinal mass, 2 cm × 3 cm, slightly movable, no tenderness, normal bilateral testes. Possible diagnosis:

A. Left indirect inguinal hernia

B. Left spermatic cord hydrocele

C. Lymphangioma

D. Lymphadenitis

E. Malignant lymphoma

118. Male, 60 years old, recently noticed gradual enlargement of the left scrotum without pain, unrelated to body position, unable to palpate the left testis and epididymis, positive transillumination test. The diagnosis should be:

A. Hydrocele of the testis

B. Hydrocele of the spermatic cord

C. Communicating hydrocele

D. Spermatic cyst

E. Strangulated inguinal hernia

119. Male, 35 years old, swelling and pain in the proximal phalanx of the right middle finger for 1 month. Physical examination: swelling of the proximal phalanx of the right middle finger, normal skin color, slight tenderness, no joint movement restriction. The most likely diagnosis is:

A. Bone cyst

B. Enchondroma

C. Giant cell tumor of bone

D. Osteochondroma

E. Bone tuberculosis

120. A construction worker accidentally fell from a high place, resulting in C5-6 vertebral fracture and dislocation with spinal cord injury. The patient is most likely to:

A. Suffocate and die

immediately

B. Become quadriplegic

C. Have elbow flexion motion, loss of elbow extension and hand function, and leg paralysis

D. Have spastic paralysis of the lower limbs

E. Have flaccid paralysis of the lower limbs

121. Female, 20 years old, fell from a height, causing lower abdominal pain. Pelvic separation and compression test positive, perineal bruising. The primary consideration for diagnosis is:

A. Pubic fracture

B. Hip dislocation

C. Coccyx fracture

D. Sacral fracture

E. Spinal cord injury

122. Male, 28 years old, low-grade fever and night sweats for 2 years, progressive weakness and paralysis in both lower limbs for 2 months, pharyngeal abscess on physical examination. The diagnosis should be considered as:

A. Cervical spine tuberculosis

B. Thoracic spine tuberculosis

C. Lumbar spine tuberculosis

D. Knee joint tuberculosis

E. Hip joint tuberculosis

123. Female, 42 years old, swelling and pain in the proximal interphalangeal joints of both hands with morning stiffness for over 6 months, morning stiffness lasting more than 2 hours and improving with activity. X-ray showed significant osteoporosis. Laboratory tests showed elevated erythrocyte sedimentation rate (ESR), negative rheumatoid factor. The most likely diagnosis is:

A. Osteoarthritis

B. Gout

C. Rheumatic arthritis

D. Rheumatoid arthritis

E. Ankylosing spondylitis

124. A 6-year-old child diagnosed with greenstick fracture of the clavicle on X-ray. The correct treatment is:

A. Sling suspension of the affected limb for 4 weeks

B. Manual reduction and figure-eight bandage fixation

C. Surgical internal fixation

D. No treatment needed

E. Bed rest for 2 weeks

125. Male, 30 years old, with low back pain for over a year. Physical examination: stiff lumbar back. No abnormalities in the nervous system. Laboratory tests: elevated ESR, positive HLA-B27. X-ray showed narrowing of the sacroiliac joint space, lumbar spine appearing "bamboo-like." The inappropriate treatment method is:

A. Immunosuppressants

B. Antibiotics

C. Appropriate traction to prevent spinal and joint deformities

D. Oral nonsteroidal anti-inflammatory drugs

E. Oral sulfasalazine enteric-coated tablets

126. Male, 18 years old, left upper arm pain and swelling for 1 month, X-ray showed an expansive cystic radiolucent area in the upper segment of the left humerus, clear boundaries, with bony septa dividing the cyst into a honeycomb appearance. The most likely diagnosis is:

A. Bone cyst

B. Fibrous dysplasia

C. Aneurysmal bone cyst

D. Enchondroma

E. Osteosarcoma

127. Male, 25 years old, right thigh injured by a mining accident, unable to stand immediately, local pain. The fastest, most convenient, and safest method to determine if there is a fracture is:

A. Portable X-ray imaging

B. Check for abnormal movement

C. Check for bone crepitus

D. Auscultate for diminished bone conduction

E. Check for longitudinal percussion pain

128. Male, 22 years old, underwent radical orchiectomy of the left testis, pathology indicated pure seminoma. Two weeks postoperatively, serum HCG returned to normal, but AFP remained high, abdominal CT suspected enlarged retroperitoneal lymph nodes, and no abnormalities were found in chest CT. The next step in treatment should be:

A. Retroperitoneal radiotherapy

B. PVB chemotherapy

C. Retroperitoneal and mediastinal radiotherapy

D. Retroperitoneal lymph node dissection

E. Radiotherapy followed by chemotherapy

129. Male, 41 years old, shoulder injury resulting in clavicle fracture. Examination revealed loss of abduction, elbow flexion, and extension function, as well as wrist and hand function, with sensory impairment. The recommended treatment is:

A. Early surgical open reduction and internal fixation, with exploration of the brachial plexus

B. Manual reduction and figure-eight bandage fixation

C. Manual reduction and external plaster fixation

D. Manual reduction and splint fixation

E. Traction reduction

130. Female, 26 years old, frequent urination, urgency, pain, and terminal hematuria for 3 months. Recently experiencing nocturia 6-8 times per night. Urinalysis: full field of red blood cells, white blood cells, and pus cells. Routine bacterial culture negative, urinary tract X-ray showed no obvious abnormalities. Treated as cystitis for 6 months without improvement. Highly suspected:

A. Urethritis

B. Urinary tract tumor

C. Chronic pyelonephritis

D. Urinary tuberculosis

E. Interstitial cystitis

131. Male, 70 years old, knee pain when going up and down stairs for 2 years. Examination: Heberden's nodes on the dorsal aspect of the distal interphalangeal joints of both hands, crepitus during knee movement. Laboratory tests: normal ESR, RF 15 IU/ml (normal <20 IU/ml). The most likely diagnosis is:

A. Gouty arthritis

B. Rheumatoid arthritis

C. Meniscal injury

D. Rheumatic arthritis

E. Osteoarthritis

132. Male, 46 years old, gastroscopy revealed a 1.0 cm × 1.0 cm superficial ulcer on the lesser curvature of the stomach, biopsy diagnosed as gastric adenocarcinoma. Surgical pathology showed lesions involving the mucosa and submucosa, with metastasis to two lymph nodes on the lesser curvature. According to histopathological staging, it belongs to:

A. Early gastric cancer

B. Intermediate gastric cancer

C. Advanced gastric cancer

D. Progressive gastric cancer

E. Progressive gastric cancer type I

133. Male, 38 years old, injured in a car accident for half an hour. Examination: cyanosis, agitation, dyspnea, irregular 4 cm wound at the 5th intercostal space on the left chest with audible air movement. The pathophysiological change of this case is:

A. Mediastinal flutter causing circulatory failure

B. Acute pulmonary edema leading to decreased gas exchange

C. Mediastinal shift to the healthy side, compensatory expansion of the injured lung

D. Increased intrapleural pressure causing respiratory failure

E. Increased pressure in the healthy pleural cavity during inhalation causing mediastinal swing to the injured side

134. Elderly male, fell downstairs and injured his right hip, resulting in shortened right lower limb. To confirm the diagnosis, the first test needed is:

A. X-ray

B. CT scan

C. MRI scan

D. Bone scan

E. Joint arthrography

135. Female, 42 years old, morning stiffness in both interphalangeal joints, unable to make a fist for 2 years; swelling, pain, and tenderness in both wrist joints for 9 months; swelling and pain in both knee joints for 7 months. Negative rheumatoid factor, synovial biopsy of the wrist joint showed inflammatory hyperplasia. X-ray of both hands showed subchondral bone cysts, ground glass changes in the surrounding bone tissue, and narrowed joint spaces. The diagnosis is considered:

A. Possible rheumatoid arthritis

B. Rheumatic arthritis

C. Typical rheumatoid arthritis

D. Primary osteoarthritis

E. Secondary osteoarthritis

136. Female, 16 years old, noticed shoulder tilt at age 13, gradually worsening. Examination: spinal deviation from the midline, uneven shoulder and scapular heights, asymmetrical back during forward bending. Standing full-length X-ray of the spine: S-shaped spine, grade II rotation. The most likely diagnosis is:

A. Osteochondrodystrophic scoliosis

B. Neuromuscular scoliosis

C. Non-structural scoliosis

D. Congenital scoliosis

E. Specific scoliosis

137. Elderly person fell with palm landing, resulting in distal radius fracture. The treatment measure is:

A. Closed reduction and external fixation with a brace

B. Open reduction and internal fixation

C. Suspension traction

D. Bone traction

E. Manual reduction and plaster fixation

138. Male, 48 years old, history of prostate cancer, multiple pain sites in the body over the past few months, especially the left thigh. X-ray indicated "osteolytic destruction of the left femur, left 3rd, 5th, and 7th ribs, right 2nd and 7th ribs." Biopsy found numerous abnormal plasma cells in the lesions. The diagnosis is likely:

A. Multiple bone metastases of prostate cancer

B. Non-ossifying fibroma

C. Multiple myeloma

D. Chondrosarcoma with systemic bone metastasis

E. Osteosarcoma with systemic bone metastasis

139. Male, 30 years old, injured right lower leg in a car accident, X-ray showed oblique fracture at the junction of the middle and lower third of the right tibia. The most likely complication is:

A. Delayed union or nonunion of the fracture

B. Vascular injury

C. Nerve injury

D. Fat embolism

E. Compartment syndrome

140. Female, 40 years old, dizziness, headache, tinnitus, and blurred vision for 3 months. Examination: positive head compression test, increased dizziness with cervical extension or lateral bending, hyperactive tendon reflexes. History of sudden falls followed by immediate awakening. The most likely diagnosis is:

A. Cervical myelopathy

B. Vertebral artery cervical s

pondylosis

C. Cervical radiculopathy

D. Hypertension

E. Ménière's syndrome

141. Boy, 3 years old, right arm pain and limited movement after being pulled by an adult. Examination: refusal to use the right hand, slightly flexed elbow, forearm pronated. The most likely diagnosis is:

A. Wrist dislocation

B. Epiphyseal cartilage injury of the forearm

C. Elbow dislocation

D. Subluxation of the radial head

E. Epiphyseal injury of the humerus

142. Male, 21 years old, left tibia upper segment pain, swelling, tenderness, and limited movement for over 4 months. Examination: superficial vein dilation on the upper left tibia, significant swelling and tenderness, palpation felt like a ping-pong ball. X-ray showed cortical destruction and fracture. Pathological biopsy reported grade III giant cell tumor of bone. The current recommended treatment is:

A. Amputation

B. Curettage and bone grafting

C. Curettage and bone cement filling

D. Curettage, inactivation, and bone grafting

E. Curettage, inactivation, and bone cement filling

143. Male, 55 years old, right neck and shoulder pain for 1 year, right hand numbness for 3 months. Examination: loss of cervical lordosis, tenderness between C5-6 spinous processes, right neck and shoulder muscle tension, decreased sensation in the radial side of the right palm, hyperactive biceps reflex, diagnosed as cervical spondylosis. The most reliable diagnostic basis is:

A. Neck and shoulder pain

B. X-ray showing bone spurs

C. Positive Hoffmann sign

D. Finger numbness

E. Limited neck movement

144. Male, 29 years old, swelling and pain in the proximal phalanx of the left second finger for 1 month. Examination: swelling of the proximal phalanx of the left second finger, slight tenderness, normal joint movement. The most appropriate treatment is:

A. Curettage and bone cement filling

B. Curettage and bone grafting

C. Amputation

D. Chemotherapy

E. Chemotherapy and amputation

145. Male, 16 years old, right proximal lower leg pain for 2 months without obvious cause. Examination: local swelling, slightly elevated skin temperature, visible varicose veins on the surface, knee joint movement not affected. Blood test showed ALT 68 U/L, ALP 228 U/L. X-ray showed worm-eaten destruction of the proximal tibia. The most appropriate treatment is:

A. Curettage and bone grafting with internal fixation

B. Chemotherapy, tumor resection, joint replacement, chemotherapy

C. Chemotherapy, curettage, bone grafting with internal fixation, chemotherapy

D. Chemotherapy, amputation, chemotherapy

E. Radiotherapy, chemotherapy, amputation, radiotherapy, chemotherapy

146. Female, 22 years old, left lower leg pain for 1 month, worsening in the past week. Examination: body temperature 37.6°C, no significant weight loss in the past month, no obvious abnormality in the left lower leg appearance, normal knee and ankle joint movement. X-ray showed a 6 cm ground-glass-like shadow in the left lower leg. The best treatment is:

A. Chemotherapy

B. Chemotherapy and lesion resection

C. Curettage and bone grafting

D. Chemotherapy and amputation

E. Lesion resection and reimplantation after inactivation

147. Female, 52 years old, progressively worsening right shoulder pain for 1 year, worse in winter and spring, better in summer and autumn, significant movement restriction, especially in abduction, elevation, and rotation, no redness, swelling, or heat in the joint. The primary diagnosis to consider is:

A. Shoulder dislocation

B. Shoulder fracture

C. Shoulder tumor

D. Periarthritis of the shoulder

E. Shoulder rupture

148. Male, 36 years old, injured in a car accident 1 hour ago. Examination: left 5th rib fracture, left pleural effusion. The correct site for closed thoracic drainage is:

A. 2nd intercostal space along the midclavicular line

B. 8th intercostal space along the anterior axillary line

C. 10th intercostal space along the posterior axillary line

D. 6th-8th intercostal space between the midaxillary line and posterior axillary line

E. Incision along the lower edge of the rib

149. Male, 42 years old, left lower limb numbness, coldness, intermittent claudication for 2 years, worsening with persistent rest pain for 3 months. History of smoking 1 pack per day. Examination: left lower limb pale, cold skin, visible purpura, erythema, significant atrophy of the left calf muscles compared to the right, disappearance of dorsalis pedis and posterior tibial artery pulses, positive Buerger test. The most likely diagnosis is:

A. Thromboangiitis obliterans

B. Raynaud's disease

C. Takayasu's arteritis

D. Polyarteritis nodosa

E. Atherosclerotic occlusive disease

150. Female, 51 years old, recurrent low back and leg pain for over 6 months, intermittent claudication of both calves when walking. Examination: numbness and pain in the dorsal lateral aspect of both calves and feet, relieved by squatting for a while, no pain when lying down. Slightly diminished pain sensation in both feet, lateral calves, and saddle area, normal Achilles tendon reflexes, palpable dorsalis pedis artery pulses. The most likely diagnosis is:

A. Central lumbar spinal stenosis

B. Nerve root canal stenosis

C. Disc herniation

D. Peripheral neuritis

E. Thromboangiitis obliterans

151. Female, 21 years old, cervical high fracture dislocation with severe respiratory distress. The first emergency measure should be:

A. Bed rest and immobilization

B. Oxygen inhalation

C. Tracheotomy

D. Surgical open reduction

E. MRI to determine the location and extent of the injury

152. Female, 35 years old, fell with wrist flexion, landing on the back of the hand, causing wrist swelling and pain. The most likely diagnosis is:

A. Colles fracture

B. Smith fracture

C. Barton fracture

D. Distal radioulnar joint dislocation

E. Scaphoid fracture

153. Female, 78 years old, fell causing right femoral neck fracture, Garden type IV. History of hypertension and coronary heart disease for 20 years. The reasonable treatment plan is:

A. Total hip replacement

B. Three-wing nail internal fixation

C. Conservative treatment

D. Compression screw internal fixation

E. Hemiarthroplasty

154. Male, 18 years old, scoliosis for 2 years, X-ray showed S-shaped spine, thoracic curve Cobb angle 43°, bending position 39°, apex at T7; lumbar curve Cobb angle 40°, bending position 32°, apex at L2. According to Lenke classification, it belongs to:

A. Main thoracic curve

B. Double thoracic curve

C. Thoracolumbar/lumbar curve

D. Double lumbar curve

E. Double main curve

155. Female child, 9 years old, sudden severe pain in the left hip 8 days ago, restricted movement of the left lower limb, accompanied by chills, high fever, general discomfort, and loss of appetite. Acute severe appearance, anemia, body temperature 38.7°C, pulse 100 beats/min, swelling at the proximal left thigh, elevated skin temperature, but no obvious abnormality in appearance, deep tenderness below the midpoint of the inguinal ligament. The most likely diagnosis is:

A. Acute rheumatic arthritis of the left hip

B. Acute suppurative arthritis of the left hip

C. Tuberculosis of the left hip

D. Inflammation of the soft tissues of the left thigh

E. Malignant tumor of the proximal left femur

156. Young male patient, initially experienced low back pain and stiffness, gradually developed kyphosis. X-ray showed blurred sacroiliac joints and disappearance of joint space. The most likely diagnosis is:

A. Chronic lumbar sprain

B. Lumbar muscle strain

C. Lumbar tuberculosis

D. Lumbar disc herniation

E. Ankylosing spondylitis

157. Male child, 12 years old, general discomfort, fever, left knee pain after a fall. Ineffective treatment with penicillin for 3 days. Examination showed swelling at the distal left femur, localized deep tenderness, joint effusion, restricted movement, slight redness, body temperature 39.6°C, WBC 18×10^9/L, N 90%, ESR 80 mm/h. The diagnosis is:

A. Rheumatic knee arthritis

B. Suppurative knee arthritis

C. Traumatic knee arthritis

D. Suppurative osteomyelitis of the distal femur

E. Osteosarcoma of the distal femur

158. Male child, 3 years old, intermittent vomiting with bile since birth. Examination: flat, soft abdomen, no gastrointestinal pattern, no tenderness, no palpable mass. Barium enema showed the ileocecal region located in the right upper abdomen. The diagnosis is considered:

A. Chronic intussusception

B. Congenital malrotation of the intestine

C. Congenital megacolon

D. Fecal impaction

E. Rectal stricture

159. Female, 18 years old, cough for 4 months, low-grade fever, left knee injury 1 month ago. Examination: limping, left quadriceps atrophy, left knee swelling, positive patellar floating test. The first examination to be performed is:

A. CT of the left knee

B. Blood routine

C. ESR

D. Left knee puncture for biochemical and bacterial culture

E. Chest and left knee X-rays

160. Female, 18 years old, cough for 4 months, low-grade fever, left knee injury 1 month ago. Examination: limping, left quadriceps atrophy, left knee swelling, positive patellar floating test. Chest X-ray showed scattered calcified foci in the left upper lung, ESR 90 mm/h. The preliminary diagnosis is:

A. Rheumatoid arthritis

B. Suppurative arthritis

C. Tuberculous arthritis

D. Traumatic arthritis

E. Rheumatic arthritis

161. Female, 18 years old, cough for 4 months, low-grade fever, left knee injury 1 month ago. Examination: limping, left quadriceps atrophy, left knee swelling, positive patellar floating test. The most valuable auxiliary examination to confirm the diagnosis is:

A. CT of the left knee

B. Blood routine

C. Blood culture

D. Left knee puncture for biochemical and bacterial culture

E. Tuberculin test

162. Male, 26 years old, humeral shaft fracture, presents with wrist drop and finger drop. The damaged nerve is:

A. Ulnar nerve

B. Brachial plexus

C. Median nerve

D. Radial nerve

E. Median and ulnar nerves

163. Male, 26 years old, humeral shaft fracture, presents with wrist drop and finger drop. The treatment method with the least pain and the most reliability is:

A. Immediate open reduction, internal fixation, and exploration and repair of the injured nerve

B. Bone traction treatment

C. Manual reduction and splint fixation

D. Manual reduction and plaster cast fixation

E. Manual reduction and hanging plaster fixation, observe for 2 months, if no recovery, then surgical treatment

164. Male, 20 years old, knee injury while playing football, persistent pain after 8 weeks. Examination: knee swelling, positive patellar floating test, positive anterior drawer test, negative side stress test. The best non-invasive diagnostic method is:

A. Knee CT

B. Knee X-ray

C. Knee exploratory surgery

D. Knee MRI

E. Knee puncture and fluid examination

165. Male, 20 years old, knee injury while playing football, persistent pain after 8 weeks. Examination: knee swelling, positive patellar floating test, positive anterior drawer test, negative side stress test. The best diagnostic and treatment method is:

A. Knee arthroscopy

B. Open exploration and repair

C. Knee CT

D. Knee puncture and fluid examination

E. Knee X-ray

166. Male, 20 years old, knee injury while playing football, persistent pain after 8 weeks. Examination: knee swelling, positive patellar floating test, positive anterior drawer test, negative side stress test. Which of the following statements about the cruciate ligament is correct:

A. The cruciate ligament is made of fibrocartilage

B. The anterior cruciate ligament is more prone to injury compared to the posterior cruciate ligament

C. Anterior cruciate ligament injuries are typically caused by lateral stress in extension

D. Isolated posterior cruciate ligament injuries do not require treatment

E. Isolated anterior cruciate ligament injuries do not require treatment

167. Male, 20 years old, knee injury while playing football, persistent pain after 8 weeks. Examination: knee swelling, positive patellar floating test, positive anterior drawer test, negative side stress test. Which physical examination finding is least likely:

A. Localized joint line tenderness

B. Positive pivot shift test

C. Positive drawer test

D. Positive patellar floating test

E. Positive varus and valgus stress test

168. Male, 6 years old, intermittent claudication and right hip pain for 4 months. If considering osteochondritis of the femoral head, the easiest sign to find is:

A. Positive "4" test of the right hip

B. Clicking during right hip flexion and extension

C. Unequal length of both lower limbs

D. Restricted right hip flexion and extension

E. Periarticular tenderness of the hip joint

169. Male, 6 years old, intermittent claudication and right hip pain for 4 months. Osteochondritis of the femoral head needs to be differentiated from which disease:

A. Acute suppurative hip arthritis

B. Suppurative osteomyelitis of the proximal femur

C. Congenital hip dislocation

D. Tuberculosis of the hip joint

E. Gluteal muscle contracture

170. Male, 6 years old, intermittent claudication and right hip pain for 4 months. The most characteristic X-ray finding of stage III osteochondritis of the femoral head is:

A. Flattened femoral head, increased density

B. Flattened femoral head, fragmented into small pieces

C. Narrowing of the joint space, destruction of the acetabulum

D. Joint swelling, widened joint space, worm-eaten destruction of the proximal femur

E. Enlarged femoral head, density similar to adjacent bone

171. Male, 6 years old, intermittent claudication and right hip pain for 4 months. Currently, surgical treatment is recommended. The purpose of the surgery is:

A. Remove necrotic bone tissue

B. Remove necrotic cartilage

C. Open joint decompression, increase acetabular coverage of the femoral head

D. Increase femoral anteversion angle

E. Decrease femoral anteversion angle

172. Male, 67 years old, left lower limb pain after walking for 2 years, worsening for 1 month. Examination: normal lumbar spine curvature, normal flexion activity, positive spinal hyperextension test, decreased sensation on the left lateral calf, left long extensor muscle strength grade 4. X-ray showed slight narrowing of the L4-L5 intervertebral space, positive straight leg raising test at 40°. He has a history of rheumatoid arthritis for over 10 years, taking prednisone. The most likely diagnosis is:

A. Acute lumbar sprain

B. Lumbar spinal stenosis

C. Lumbar spondylolysis

D. L4-L5 disc herniation

E. Thromboangiitis obliterans

173. Male, 67 years old, left lower limb pain after walking for 2 years, worsening for 1 month. Examination: normal lumbar spine curvature, normal flexion activity, positive spinal hyperextension test, decreased sensation on the left lateral calf, left long extensor muscle strength grade 4. X-ray showed slight narrowing of the L4-L5 intervertebral space, positive straight leg raising test at 40°. He has a history of rheumatoid arthritis for over 10 years, taking prednisone. Which examination is unnecessary:

A. MRI

B. CT

C. Evoked potential

D. Blood routine

E. Bone scan

174. Male, 67 years old, left lower limb pain after walking for 2 years, worsening for 1 month. Examination: normal lumbar spine curvature, normal flexion activity, positive spinal hyperextension test, decreased sensation on the left lateral calf, left long extensor muscle strength grade 4. X-ray showed slight narrowing of the L4-L5 intervertebral space, positive straight leg raising test at 40°. He has a history of rheumatoid arthritis for over 10 years, taking prednisone. If considering surgical treatment, during and after surgery, the patient should not:

A. Stop corticosteroids

B. Use antibiotics

C. Use sedatives

D. Continue corticosteroids

E. Use analgesics

175. Male, 30 years old, car accident caused a comminuted fracture of the right acetabulum. Examination: blood pressure 80/50 mmHg, pale face, right foot unable to dorsiflex. The most likely complication is:

A. Urinary system injury

B. Colorectal injury

C. Hypovolemic shock

D. Genital injury

E. Nerve injury

176. Male, 30 years old, car accident caused a comminuted fracture of the right acetabulum. Examination: blood pressure 80/50 mmHg, pale face, right foot unable to dorsiflex. The most appropriate treatment is:

A. Pelvic X-ray

B. Pelvic CT

C. Open an IV line, fluid replacement, and anti-shock treatment

D. Detailed physical examination

E. Lumbar MRI

177. Male, 30 years old, car accident caused a comminuted fracture of the right acetabulum. Examination: blood pressure 80/50 mmHg, pale face, right foot unable to dorsiflex. Which sign is least likely to appear:

A. Positive local palpation test

B. Positive local right lower limb axial percussion test

C. Positive pelvic separation and compression test

D. Positive right Thomas test

E. Positive right "4" test

178. Male, 20 years old, bike accident, landed on the right shoulder, X-ray showed a midshaft fracture of the right clavicle with significant displacement. During the consultation, the patient kept his head tilted to the right and supported his right elbow with his left hand. The main reason is:

A. Fracture with right neck and shoulder soft tissue sprain, adopting a forced position

B. Fracture with right brachial plexus injury, weakened right

upper limb muscle strength

C. Fracture with right subclavian vascular injury, reducing upper limb ischemia in this position

D. This position temporarily immobilizes and reduces bleeding from the fracture site

E. This position relaxes muscles and reduces gravity traction on the upper limb, reducing fracture pain

179. Male, 20 years old, bike accident, landed on the right shoulder, X-ray showed a midshaft fracture of the right clavicle with significant displacement. Following the principles of fracture reduction, the direction of traction for closed reduction should be:

A. Posterior, upward, and outward

B. Upward and outward

C. Posterior and downward

D. Anterior and downward

E. Anterior and upward

180. Male, 20 years old, bike accident, landed on the right shoulder, X-ray showed a midshaft fracture of the right clavicle with significant displacement. Surgery is necessary in the following situation:

A. The patient is young and concerned about aesthetics

B. Significant overlap and shortening of the fracture ends

C. After reduction, there is still 2/3 lateral displacement of the fracture ends

D. After manual reduction, the fracture ends cannot touch due to obstruction

E. Numbness in the affected limb after manual reduction and plaster fixation

181. Female, 72 years old, fell while descending stairs, injured right hip, examination showed shortened right lower limb, 50° external rotation deformity, slight right hip swelling, and percussion pain. The most likely diagnosis is:

A. Posterior hip dislocation

B. Anterior hip dislocation

C. Femoral neck fracture

D. Intertrochanteric fracture

E. Right hip soft tissue injury

F. Femoral shaft fracture

G. Acetabular fracture

H. Ischial fracture

182. Female, 72 years old, fell while descending stairs, injured right hip, examination showed shortened right lower limb, 50° external rotation deformity, slight right hip swelling, and percussion pain. To confirm the diagnosis in the emergency department, the examination needed is:

A. Plain X-ray

B. CT scan

C. MRI

D. Bone scan

E. Vascular ultrasound

F. Joint arthrography

G. Ultrasound

H. Enhanced CT

183. Female, 72 years old, fell while descending stairs, injured right hip, examination showed shortened right lower limb, 50° external rotation deformity, slight right hip swelling, and percussion pain. X-ray and CT confirmed a femoral neck fracture. The possible complication is:

A. Fat embolism

B. Sciatic nerve injury

C. Coxa vara deformity

D. Avascular necrosis of the femoral head

E. Traumatic ossification around the hip joint

F. Deep vein thrombosis

G. Rectal injury

H. Urethral injury

184. Male, 11 years old, no obvious cause of right hip pain, limited movement for six months, worsened for one week, afternoon low-grade fever, night sweats, fatigue. Examination: right hip tenderness, no swelling, right hip joint stiffness, limited flexion and extension, X-ray showed no significant destruction of right hip bone, slightly narrowed joint space, history of primary pulmonary tuberculosis. The primary consideration is:

A. Acute suppurative arthritis of the right hip

B. Ankylosing spondylitis involving the hip

C. Transient synovitis of the right hip

D. Tuberculosis of the right hip

E. Osteoarthritis of the right hip

F. Traumatic arthritis of the right hip

185. Male, 11 years old, no obvious cause of right hip pain, limited movement for six months, worsened for one week, afternoon low-grade fever, night sweats, fatigue. Examination: right hip tenderness, no swelling, right hip joint stiffness, limited flexion and extension, X-ray showed no significant destruction of right hip bone, slightly narrowed joint space, history of primary pulmonary tuberculosis. To further confirm the diagnosis, the most valuable examination is:

A. Blood routine and ESR

B. CT scan

C. ECT

D. Hip arthroscopy and synovial biopsy

E. Joint puncture and smear examination

F. MRI

186. Male, 11 years old, no obvious cause of right hip pain, limited movement for six months, worsened for one week, afternoon low-grade fever, night sweats, fatigue. Examination: right hip tenderness, no swelling, right hip joint stiffness, limited flexion and extension, X-ray showed no significant destruction of right hip bone, slightly narrowed joint space, history of primary pulmonary tuberculosis. The early X-ray manifestation of hip tuberculosis is:

A. Mainly bone hyperplasia

B. Coexistence of bone hyperplasia and destruction

C. Mainly bone osteoporosis

D. Localized decalcification

E. Disappearance of joint space

F. Hip joint fusion

187. Male, 11 years old, no obvious cause of right hip pain, limited movement for six months, worsened for one week, afternoon low-grade fever, night sweats, fatigue. Examination: right hip tenderness, no swelling, right hip joint stiffness, limited flexion and extension, X-ray showed no significant destruction of right hip bone, slightly narrowed joint space, history of primary pulmonary tuberculosis. Hip tuberculosis often presents as:

A. Single joint pain

B. Acute onset, rapid progression

C. Multiple joint pain

D. Mainly spinal facet joint pain

E. Common in the elderly

F. Self-healing

188. Male, 11 years old, no obvious cause of right hip pain, limited movement for six months, worsened for one week, afternoon low-grade fever, night sweats, fatigue. Examination: right hip tenderness, no swelling, right hip joint stiffness, limited flexion and extension, X-ray showed no significant destruction of right hip bone, slightly narrowed joint space, history of primary pulmonary tuberculosis. The following is not a common physical examination method for diagnosing hip tuberculosis:

A. Hip extension test

B. "4" test

C. Thomas test

D. Pick-up test

E. Hip rolling test

F. Straight leg raising test

189. Male, 11 years old, no obvious cause of right hip pain, limited movement for six months, worsened for one week, afternoon low-grade fever, night sweats, fatigue. Examination: right hip tenderness, no swelling, right hip joint stiffness, limited flexion and extension, X-ray showed no significant destruction of right hip bone, slightly narrowed joint space, history of primary pulmonary tuberculosis. The most common type of hip tuberculosis is:

A. Simple synovial tuberculosis

B. Mixed type tuberculosis

C. Simple bone tuberculosis

D. Total joint tuberculosis

E. General systemic symptoms

F. None of the above

190. Male, 11 years old, no obvious cause of right hip pain, limited movement for six months, worsened for one week, afternoon low-grade fever, night sweats, fatigue. Examination: right hip tenderness, no swelling, right hip joint stiffness, limited flexion and extension, X-ray showed no significant destruction of right hip bone, slightly narrowed joint space, history of primary pulmonary tuberculosis. Late-stage pathological changes of hip tuberculosis exclude:

A. Cold abscess

B. Pathological dislocation

C. Narrowed joint space

D. Joint space remains

E. Joint stiffness

F. Possible sinus tract formation

191. Male, 11 years old, no obvious cause of right hip pain, limited movement for six months, worsened for one week, afternoon low-grade fever, night sweats, fatigue. Examination: right hip tenderness, no swelling, right hip joint stiffness, limited flexion and extension, X-ray showed no significant destruction of right hip bone, slightly narrowed joint space, history of primary pulmonary tuberculosis. The differential point between hip tuberculosis and transient synovitis is:

A. The latter is transient, without afternoon low-grade fever and night sweats

B. The former is more common in adults

C. The latter is more common in adults

D. X-ray of the latter shows bone destruction

E. Difficult to distinguish

F. The latter is persistent

192. Male, 11 years old, no obvious cause of right hip pain, limited movement for six months, worsened for one week, afternoon low-grade fever, night sweats, fatigue. Examination: right hip tenderness, no swelling, right hip joint stiffness, limited flexion and extension, X-ray showed no significant destruction of right hip bone, slightly narrowed joint space, history of primary pulmonary tuberculosis. The common site for simple bone tuberculosis in the hip joint is:

A. Ligamentum teres femoris

B. Edge of the acetabulum

C. Edge of the femoral head

D. Edge of the femoral head or iliac part of the acetabulum

E. Center of the femoral head

F. None of the above

193. Male child, 4 years old, fell from a 50 cm high bed while playing, left elbow pain and refusal to touch. Examination: the child refused to use the left hand and move the elbow, no obvious deformity. The first examination is:

A. X-ray

B. Electrophysiological testing

C. CT scan

D. Ultrasound

E. MRI

F. ECG

194. Male child, 4 years old, fell from a 50 cm high bed while playing, left elbow pain and refusal to touch. Examination: the child refused to use the left hand and move the elbow, no obvious deformity. If deformity is observed on examination, the first examination is:

A. X-ray of the left humerus in AP and lateral views

B. X-ray of the left elbow joint in AP and lateral views

C. X-ray of the left radius and ulna in AP and

lateral views

D. CT scan of the humerus

E. MRI of the elbow joint

F. CT scan of the elbow joint

195. Male child, 4 years old, fell from a 50 cm high bed while playing, left elbow pain and refusal to touch. Examination: the child refused to use the left hand and move the elbow, no obvious deformity. If X-ray is negative, the most likely diagnosis is:

A. Supracondylar fracture of the humerus

B. Shoulder dislocation

C. Elbow dislocation

D. Subluxation of the radial head

E. Soft tissue injury of the elbow

F. Olecranon fracture of the ulna

196. Male child, 4 years old, fell from a 50 cm high bed while playing, left elbow pain and refusal to touch. Examination: the child refused to use the left hand and move the elbow, no obvious deformity. The most common age for this condition is:

A. Under 5 years

B. 3-8 years

C. 5-10 years

D. 10-12 years

E. 12-15 years

F. 15-18 years

197. Male child, 4 years old, fell from a 50 cm high bed while playing, left elbow pain and refusal to touch. Examination: the child refused to use the left hand and move the elbow, no obvious deformity. The most likely positive sign is:

A. Abnormal elbow triangle relationship

B. Swelling of the elbow joint

C. Positive Dugas sign

D. Positive radial head tenderness

E. Elbow valgus

F. Elbow varus

198. Male child, 4 years old, fell from a 50 cm high bed while playing, left elbow pain and refusal to touch. Examination: the child refused to use the left hand and move the elbow, no obvious deformity. The further management of this condition is:

A. Shoulder CT

B. Shoulder X-ray

C. Elbow CT

D. Manual reduction without fixation

E. Manual reduction with sling fixation

F. Elbow MRI

199. Male child, 4 years old, fell from a 50 cm high bed while playing, left elbow pain and refusal to touch. Examination: the child refused to use the left hand and move the elbow, no obvious deformity. The contributing factor for this condition is:

A. Anterior inclination angle between the humeral shaft axis and humeral condyle axis

B. Rotational external force of the forearm

C. Weak annular ligament at the radial neck

D. Simultaneous action of abduction and external rotation forces on the humeral head

E. Carrying angle between the humeral longitudinal axis and ulnar longitudinal axis

F. Ligament rupture

200. Male child, 4 years old, fell from a 50 cm high bed while playing, left elbow pain and refusal to touch. Examination: the child refused to use the left hand and move the elbow, no obvious deformity. The best treatment plan for recurrence 3 months after reduction due to being pulled by a family member is:

A. Plaster fixation

B. Splint fixation

C. Sling suspension

D. Manual reduction and sling suspension

E. Surgical open reduction

F. Observation

201. Female child, 5 years old, limping in the right lower limb for 3 years. She started walking at age 2 and was found to have unstable gait and waddling gait, no history of trauma. Examination: body temperature 36.5°C, no redness, swelling, or tenderness in the right lower limb, normal muscle strength, normal skin sensation, normal hip flexion and extension, slightly restricted abduction, normal movement in other joints. The following conditions cannot be ruled out:

A. Right lower limb nerve paralysis

B. Suppurative arthritis of the right hip

C. Tuberculosis of the right knee joint

D. Poliomyelitis sequelae

E. Transient synovitis of the right hip

F. Congenital hip dislocation

202. Female child, 5 years old, limping in the right lower limb for 3 years. She started walking at age 2 and was found to have unstable gait and waddling gait, no history of trauma. Examination: body temperature 36.5°C, no redness, swelling, or tenderness in the right lower limb, normal muscle strength, normal skin sensation, normal hip flexion and extension, slightly restricted abduction, normal movement in other joints. Allis sign (+), Trendelenburg test (+), X-ray showed: disruption of Shenton's line on the right, acetabular index 50°, femoral head located in the upper outer quadrant of Perkin's square, underdeveloped, smaller but normal density, anteversion angle 80°, no acetabular destruction, neck-shaft angle 140°. The following additional targeted examinations and specialized physical tests should be performed:

A. X-ray of both hips in AP view

B. X-ray of the affected hip in AP, lateral, and oblique views

C. Routine CT scan to determine femoral neck anteversion angle

D. Hip arthrography to identify factors hindering reduction

E. McMurray test

F. Pelvic separation and compression test

203. Female child, 5 years old, limping in the right lower limb for 3 years. She started walking at age 2 and was found to have unstable gait and waddling gait, no history of trauma. Examination: body temperature 36.5°C, no redness, swelling, or tenderness in the right lower limb, normal muscle strength, normal skin sensation, normal hip flexion and extension, slightly restricted abduction, normal movement in other joints. Allis sign (+), Trendelenburg test (+), X-ray showed: disruption of Shenton's line on the right, acetabular index 50°, femoral head located in the upper outer quadrant of Perkin's square, underdeveloped, smaller but normal density, anteversion angle 80°, no acetabular destruction, neck-shaft angle 140°. The diagnosis is:

A. Unequal leg length

B. Congenital coxa vara (right)

C. Avascular necrosis of the right femoral head

D. Congenital multiple joint contracture

E. Congenital dislocation of the right hip

204. Female child, 5 years old, limping in the right lower limb for 3 years. She started walking at age 2 and was found to have unstable gait and waddling gait, no history of trauma. Examination: body temperature 36.5°C, no redness, swelling, or tenderness in the right lower limb, normal muscle strength, normal skin sensation, normal hip flexion and extension, slightly restricted abduction, normal movement in other joints. Regarding the pathological changes of congenital dislocation of the hip (CDH), the following are correct:

A. The acetabulum is deeper than normal

B. The cartilage of the acetabular notch turns outwards and upwards

C. Fibrofatty tissue and thickened round ligament in the acetabular fossa

D. The femoral head becomes conical in infancy

E. Significant shortening of the femoral neck

F. Bilateral dislocation causes posterior pelvic tilt, leading to decreased lumbar lordosis

205. Female child, 5 years old, limping in the right lower limb for 3 years. She started walking at age 2 and was found to have unstable gait and waddling gait, no history of trauma. Examination: body temperature 36.5°C, no redness, swelling, or tenderness in the right lower limb, normal muscle strength, normal skin sensation, normal hip flexion and extension, slightly restricted abduction, normal movement in other joints. Regarding the symptoms and signs of CDH, the following are correct:

A. Ortolani test is often used for children over 3 years old

B. Allis sign is considered a specific sign of CDH

C. Telescoping test is often positive

D. Positive Trendelenburg test indicates gluteus medius dysfunction on the side of the raised leg

E. Unilateral hip dislocation causes a waddling gait

F. Asymmetrical gluteal and thigh folds, empty femoral triangle, widened perineum

206. Female child, 5 years old, limping in the right lower limb for 3 years. She started walking at age 2 and was found to have unstable gait and waddling gait, no history of trauma. Examination: body temperature 36.5°C, no redness, swelling, or tenderness in the right lower limb, normal muscle strength, normal skin sensation, normal hip flexion and extension, slightly restricted abduction, normal movement in other joints. Regarding the X-ray manifestations and measurements of CDH, the following are correct:

A. The y-line connects the centers of the y-shaped cartilages of both acetabula

B. The normal femoral head should be in the upper inner quadrant of Perkin's square

C. The acetabular index for children over 2 years old is normally below 20

D. Shenton's line is an arc connecting the medial edge of the femoral neck and the lower edge of the obturator foramen

E. The normal CE angle is 20-40°

F. The femoral neck anteversion angle is the angle formed between the frontal plane of the femur and the femoral neck

207. The patient is a 5-year-old girl admitted to the hospital due to a 3-year history of limping on her right lower limb. She learned to walk at the age of 2, and at that time, her right lower limb was found to be unstable with a stepping gait, with no history of trauma. Physical examination: temperature 36.5℃, no redness, swelling, or tenderness in the right lower limb, normal muscle strength in the lower limbs, normal skin sensation, normal flexion and extension of the right hip joint, slightly limited abduction, and normal movement of other joints.

Question: How should this patient be treated?

A. Immediate open reduction and acetabuloplasty

B. Manual reduction and frog-leg cast immobilization

C. Conservative treatment for 9 months first, and if ineffective, consider simple open reduction

D. Manual reduction and immobilization with a special Rosen brace

E. First release the iliopsoas and adductor muscles, then traction the femoral head to the acetabular level, followed by open reduction and acetabuloplasty (Penberton surgery)

F. Femoral rotational osteotomy is also required to correct the femoral neck anteversion angle

208. A 29-year-old man accidentally twisted his left knee while playing football, immediately felt pain, and experienced some improvement after resting in bed. However, 2 weeks later, he still had significant pain and his left knee often "locked" while walking. He came to the hospital for consultation, and physical examination found a positive McMurray test and a negative drawer test.

Question: What is the most likely diagnosis for this patient?

A. Left patellar chondromalacia

B. Loose body in the left knee joint

C. Left knee meniscus injury

D. Left knee collateral ligament injury

E. Left knee cruciate ligament injury

F. Left patellar fracture

209. A 29-year-old man accidentally twisted his left knee while playing football, immediately felt pain, and experienced some improvement after resting in bed. However, 2 weeks later, he still had significant pain and his left knee often "locked" while walking. He came to the hospital for consultation, and physical examination found a positive McMurray test and a negative drawer test.

Question: What common examination methods can assist in the above diagnosis?

A. Anteroposterior and lateral X-ray of the knee joint

B. Knee joint MRI

C. Knee joint arthrography

D. Knee arthroscopy

E. Knee joint bone scan

F. Knee joint ultrasound

210. A 29-year-old man accidentally twisted his left knee while playing football, immediately felt pain, and experienced some improvement after resting in bed. However, 2 weeks later, he still had significant pain and his left knee often "locked" while walking. He came to the hospital for consultation, and physical examination found a positive McMurray test and a negative drawer test.

Question: What are the suitable treatment methods for this patient?

A. Arthroscopic exploration

B. Open knee surgery

C. Continued rest and pain medication

D. Long leg cast immobilization in extension position for 4-6 weeks

E. Coordinated physiotherapy to strengthen knee joint movement

F. Joint fusion

1. Regarding the X-ray features of knee joint tuberculosis, which of the following is incorrect:

A. Swelling of the suprapatellar bursa and soft tissues

B. Narrowing or disappearance of the joint space

C. Anterior dislocation of the tibia

D. Bone appears fuzzy like frosted glass

E. Marginal bone erosion and destruction

2. The theoretical lifespan of an artificial hip joint prosthesis is approximately:

A. 10-15 years

B. 15-20 years

C. 20-25 years

D. 25-30 years

E. 30-35 years

3. Regarding the treatment of chronic musculoskeletal injuries, the least commonly used method clinically is:

A. Restricting injurious actions and correcting poor posture

B. Local injection of corticosteroids

C. Physical therapy and massage

D. Taking anti-inflammatory and analgesic drugs

E. Surgical treatment

4. During the healing process of fractures, which of the following does not belong to the hematoma organization evolution stage:

A. Formation of a hematoma at the fracture ends and surrounding soft tissue

B. A few millimeters of bone necrosis at the fracture ends

C. The hematoma and necrotic soft tissue cause a local aseptic inflammatory reaction

D. Osteoblasts from the inner and outer bone membrane form bone-like tissue that calcifies into new bone

E. New capillaries and phagocytes, fibroblasts invade from the periphery

5. Which of the following is not a complication or sequelae of tibia and fibula fractures:

A. Charcot joint

B. Claw toe deformity

C. Sudeck’s atrophy

D. Refracture

E. Nonunion and delayed healing

6. After aspirating pus from under the periosteum or in the bone marrow in acute osteomyelitis, the most critical treatment measure is:

A. Multiple pus aspirations and antibiotic injections

B. Bacterial culture and drug sensitivity test of the pus, adjust medication based on results

C. Combined use of large doses of antibiotics

D. Local drainage

E. Local immobilization to prevent pathological fractures

7. In lumbar disc herniation, radiating pain in the lower extremities is most commonly found in:

A. Sciatic nerve distribution area

B. Obturator nerve distribution area

C. Pudendal nerve distribution area

D. Femoral nerve distribution area

E. Lateral femoral cutaneous nerve distribution area

8. Regarding the criteria for fracture healing, which of the following is incorrect:

A. No local tenderness or percussion pain

B. No abnormal movement at the fracture site

C. X-ray shows the fracture line is blurred, with continuous callus passing through the fracture line

D. After removing the external fixation, the upper limb can hold a 10 kg object for 10 minutes

E. Continuous observation for 2 weeks shows no deformation at the fracture site

9. Which type of fracture requires anatomical reduction:

A. Femoral shaft fracture

B. Humeral shaft fracture

C. Metacarpal fracture

D. Fibula fracture

E. Talocrural joint fracture

10. The third stage of fracture healing is:

A. Hematoma organization evolution stage

B. Primary callus formation stage

C. Callus remodeling stage

D. Intramembranous ossification absorption stage

E. Cartilage ossification absorption stage

11. Which of the following options does not match the clinical features of stenosing tenosynovitis:

A. The lesion is not limited to the flexor tendon sheath

B. Local steroid injection is effective

C. The lesion is most commonly located on the palmar side of the thumb metacarpophalangeal joint

D. Morning stiffness and pain, relieved after activity

E. Local tenderness

12. Which of the following descriptions of muscle strength levels is incorrect:

A. Level 1: Complete inability to contract, complete paralysis

B. Level 2: Muscle contraction can cause joint movement, but cannot overcome gravity

C. Level 3: Can move the joint, but cannot resist resistance

D. Level 4: Can resist some resistance, but muscle strength is weak

E. Level 5: Normal muscle strength

13. The correct principle of fracture treatment is:

A. Reduction, immobilization, and functional exercise

B. Generally requires anatomical reduction

C. Adhere to a combination of immobilization and activity

D. Emphasize both bone and soft tissue

E. Consider both local and systemic treatment

14. After nerve injury, the early manifestation of autonomic nerve dysfunction is:

A. Pale skin

B. Increased skin temperature

C. Sweaty skin

D. Deepened skin creases

E. Decreased skin temperature

15. Interspinous ligament injuries most commonly occur between:

A. L2-L3

B. L3-L5

C. L4-L5

D. T12-L1

E. L5-S1

16. Ischemic contracture of the forearm is most commonly seen in:

A. Supracondylar fracture of the humerus

B. Radial fracture

C. Ulnar fracture

D. Combined radial and ulnar fractures

E. Colles fracture

17. The most common fracture in the elbow of children is:

A. Lateral condyle fracture of the humerus

B. Supracondylar fracture of the humerus

C. Medial epicondyle fracture of the humerus

D. Radial neck fracture

E. Olecranon fracture of the ulna

18. Which of the following fractures is not suitable for external fixation with a small splint:

A. Closed tibial shaft fracture

B. Closed radial and ulnar fractures in children

C. Open tibial fracture with a small wound that has healed after treatment

D. Severe open humeral shaft fracture after emergency debridement and suture

E. Old humeral shaft fracture that is still suitable for manual reduction

19. Carpal tunnel syndrome is caused by compression of which structure within the carpal tunnel:

A. Ulnar nerve

B. Ulnar artery

C. Radial nerve

D. Median nerve

E. Radial artery

20. The most important sign of greenstick fractures is:

A. Local swelling and bruising

B. Abnormal movement

C. Local tenderness and indirect tenderness

D. Bone crepitus or bone rubbing sound

E. Loss of function

21. Which of the following is a spinal X-ray manifestation of ankylosing spondylitis:

A. The vertebral body appears frosted glass-like

B. The small joints of the vertebrae and the costovertebral joints are fuzzy with unclear edges

C. Narrowing of the intervertebral disc, calcification of the annulus fibrosus of the intervertebral space

D. “Bamboo spine” changes

E. All of the above

22. Which herniated disc can weaken or abolish the Achilles tendon reflex:

A. L3-L4

B. L2-L3

C. L4-L5

D. L5-S1

E. S1-S2

23. The sequence of physical examination for the musculoskeletal system is:

A. Touch, look, move, measure, special examination

B. Move, touch, look, special examination

C. Look, touch, move, measure, special examination

D. Measure, move, look, touch, special examination

E. Special examination, look, touch, move, measure

24. Regarding the examination for congenital hip dislocation, which test is not suitable for a 3-year-old child:

A. Ortolani test

B. One-leg standing test

C. "Pump handle" sign

D. Hip flexion, abduction, and external rotation test

E. Galeazzi sign

25. Open joint dislocation means:

A. The joint cavity communicates with the external environment

B. Dislocation with skin laceration

C. Dislocation with joint capsule rupture

D. Dislocation with ligament rupture

E. Dislocation with joint surface fracture

26. Regarding the goals of scoliosis treatment, which of the following is incorrect:

A. Correct deformity

B. Minimize the fusion range as much as possible

C. Fuse as many segments as possible to prevent complications

D. Achieve stability

E. Maintain balance

27. The goal of surgical treatment for chronic osteomyelitis does not include:

A. Eliminate sinus tract

B. Remove large areas of sequestrum

C. Eliminate dead space

D. Close the wound

E. Identify the causative pathogen

28. The initial medical accident technical appraisal is organized by which department:

A. Municipal Health Bureau

B. Provincial Health Department

C. Medical department of each hospital

D. Accident appraisal committee of each hospital

E. County (city) local medical association with direct jurisdiction

29. The Declaration of Helsinki is an international document on human experimentation, which mainly stipulates:

A. Compare experiments with the best existing diagnostic and treatment methods

B. Ethical requirements for biomedical research involving human subjects

C. Ensure that each subject receives the best diagnostic and treatment methods

D. The experiment memo records some matters that need special explanation

E. The medical goal is subordinate to the benefits obtained in diagnosing and treating the test subjects

30. The advantages of prospective cohort studies do not include:

A. Can be used to study the relationship between one exposure and multiple diseases

B. Can directly calculate the incidence rate

C. Requires a small sample size and low cost

D. Mainly used to test causal hypotheses

E. The time sequence relationship between disease and cause is clear, facilitating the determination of causality

31. In lumbar tuberculosis with left iliac fossa abscess, a positive Thomas sign indicates:

A. Restricted hip joint movement

B. Radiating pain in the left lower limb

C. Stimulation of the left psoas muscle

D. Stimulation of the right psoas muscle

E. Stimulation of the gluteus maximus

32. Which of the following is not a sign of lumbar disc herniation:

A. Tenderness at the tip of the transverse process of the third lumbar vertebra

B. Tenderness and spasm of the sacrospinal muscles

C. Restricted lumbar movement

D. Lumbar scoliosis

E. Positive straight leg raise test and enhancement test

33. The characteristics of gouty arthritis pain, which is incorrect:

A. The lesion does not involve the hands, knees, wrists, and elbow joints

B. Severe joint pain often occurs suddenly after drinking alcohol, overexertion, or eating a high-purine diet

C. The lesion is self-limiting and subsides on its own after 1-2 weeks, but often recurs

D. Joint deformity can occur in the late stage

E. Most commonly involves the first metatarsophalangeal joint and thumb joint

34. Regarding femoral neck fractures, which statement is incorrect:

A. Subcapital fractures are prone to femoral head necrosis

B. Basal fractures are easy to heal

C. The higher the Garden classification of femoral neck fractures, the lower the nonunion rate

D. Children with femoral neck fractures are prone to avascular necrosis of the femoral head

E. Femoral neck fractures can still develop avascular necrosis of the femoral head several years after healing

35. Regarding debridement, which of the following is incorrect:

A. Thoroughly remove foreign bodies and blood clots in the wound

B. Perform primary suturing after thorough debridement of open wounds

C. Administer intravenous antibiotics to prevent infection

D. When nerve repair is difficult, it can be left for secondary treatment

E. Blood vessels that do not affect the blood supply to the affected limb can be ligated

36. In the case of elevated body temperature in open fractures, consider the possibility of:

A. Pain stimulation

B. Infection

C. Shock

D. Blood loss

E. Loss of tissue fluid

37. Regarding popliteal cysts, which of the following is incorrect:

A. Also known as Baker’s cyst

B. Should be treated surgically immediately after diagnosis

C. Can be treated with local aspiration, drainage, and steroid injection

D. Mostly occurs in the semimembranosus tendon bursa and the bursa between the medial head of the gastrocnemius and the semimembranosus

E. Often communicates with the joint cavity

38. The most valuable examination for diagnosing adult spinal tuberculosis is:

A. Bacterial culture

B. Erythrocyte sedimentation rate

C. X-ray film

D. Tuberculin test

E. Routine blood and urine tests and blood biochemical tests

39. Primary functional scoliosis:

A. Has significant structural changes

B. No primary curvature

C. Has compensatory secondary curvature

D. Functional scoliosis cannot develop into structural scoliosis

E. None of the above is correct

40. Regarding the treatment principles of patellar chondromalacia, which is inappropriate:

A. Non-steroidal anti-inflammatory drugs

B. Physical therapy

C. Quadriceps exercises

D. Intra-articular steroid injection

E. Early surgical treatment if found

41. According to the Prescription Management Regulations, the correct statement about the prescription rights of advanced trainee physicians is:

A. Advanced trainee physicians do not have prescription rights in the medical institutions where they are training

B. Advanced trainee physicians have the same prescription rights in the training institutions as in their original units

C. The prescription rights are granted based on the advanced trainee physician's actual performance in their specialty in the training institution

D. The prescription rights are granted after passing a unified examination organized by the health administration department of the training institution

E. The prescriptions issued by advanced trainee physicians are valid only after being reviewed and signed by the clinical department head of the training institution

42. Septic arthritis most commonly occurs in:

A. Shoulder and elbow joints

B. Elbow and wrist joints

C. Wrist and hip joints

D. Hip and knee joints

E. Elbow and ankle joints

43. The most common degenerative intervertebral spaces in lumbar disc herniation are arranged in the following order:

A. L3-4; L5-S1; L4-5

B. L3-4; L4-5; L5-S1

C. L4-5; L5-S1; L4-4

D. L5-S1; L3-4; L4-5

E. L4-5; L3-4; L5-S1

44. The first choice for treating an adult with a fresh closed femoral shaft fracture is:

A. Open reduction and internal fixation

B. Manual reduction and splint fixation

C. Manual reduction and hip spica cast fixation

D. Skin traction and small splint fixation

E. Bed rest

45. Bone and joint tuberculosis are most commonly secondary to:

A. Intestinal tuberculosis

B. Pulmonary tuberculosis

C. Renal tuberculosis

D. Pleural tuberculosis

E. Lymph node tuberculosis

46. The standard X-ray position for developmental dysplasia of the hip is:

A. Anteroposterior view of both hips

B. Lateral view of both hips

C. Single hip abduction view

D. Both hips adduction view

E. Single hip adduction view

47. When no urine can be obtained after an emergency catheterization following a pelvic fracture, the first consideration should be to exclude:

A. Bladder rupture

B. Anterior urethral injury

C. Posterior urethral injury

D. Hemorrhagic shock

E. Anuria due to renal failure

48. Regarding the characteristics of tibial tubercle osteochondrosis, which of the following is incorrect:

A. Mostly seen in boys under 10 years old

B. The main treatment is to reduce physical activity

C. Can self-heal

D. Symptoms worsen after exercise

E. Local steroid injection treatment is generally not performed

49. "Wherever the art of medicine is loved, there is also a love of humanity" is from:

A. "In Memory of Norman Bethune"

B. "Collections of Galen"

C. "Hippocratic Oath"

D. "Guangji Medical Journal"

E. "Prayer of Maimonides"

50. Regarding the diagnosis of subluxation of the radial head, which of the following is incorrect:

A. History of traction

B. Elbow flexion, forearm pronation

C. Local swelling and deformity

D. Local tenderness

E. Negative X-ray examination

51. Muscular atrophy due to poliomyelitis-induced limb paralysis is:

A. Nutritional atrophy

B. Neurogenic atrophy

C. Disuse atrophy

D. Compressive atrophy

E. Physiological atrophy

52. The clinical characteristics of Brown-Séquard syndrome do not include:

A. Complete upper motor neuron paralysis on the same side below the level of injury

B. Spastic paralysis on the same side below the level of injury

C. Hyperreflexia and pathological reflexes on the same side below the level of injury

D. Loss of pain and temperature sensation on the contralateral side below the level of injury

E. No lower motor neuron paralysis on the contralateral side below the level of injury

53. The bone tumor with the poorest prognosis is:

A. Undifferentiated reticulosarcoma

B. Osteosarcoma

C. Primary chondrosarcoma

D. Grade I giant cell tumor of bone

E. Fibrosarcoma of bone

54. The main cause of early paraplegia in spinal tuberculosis is:

A. Compression of the spinal cord by pus, granulation tissue, and sequestrum

B. Thrombosis of the anterior spinal artery

C. Meningitis

D. Compression due to spinal deformity

E. Compression due to pathological dislocation

55. To evaluate nerve repair after peripheral nerve injury, the least significant test is:

A. Electrical stimulation test

B. Nerve trunk percussion test

C. Electromyography

D. CT scan

E. Nerve conduction velocity test

56. Which of the following principles of debridement is incorrect:

A. Remove foreign bodies from the wound

B. Excise non-viable tissue

C. Achieve thorough hemostasis

D. Suture the wound as appropriate

E. Drainage must be placed

57. The most common site for bone and joint tuberculosis is:

A. Knee joint

B. Spine

C. Elbow joint

D. Ankle joint

E. Hip joint

58. Which type of displacement after a child's fracture is most likely to cause permanent functional loss:

A. Rotational displacement

B. Overlapping of fracture ends

C. Lateral displacement

D. Angulation

E. Separation of fracture ends

59. Regarding the range of motion of the wrist joint, which of the following is correct:

A. Dorsiflexion 70°

B. Palmar flexion 80°

C. Radial deviation 25°

D. Ulnar deviation 50°

E. Neutral position 0°

60. Clinical treatment methods for bone metastases do not include

A. Radionuclide therapy

B. Hyperbaric oxygen therapy

C. Radiotherapy and chemotherapy

D. Application of bone resorption inhibitors such as bisphosphonates and calcitonin, and endocrine therapy

E. Surgical treatment

61. The main clinical significance of X-ray examination in the treatment of fractures is

A. Clear diagnosis

B. Understanding the mechanism of injury

C. Understanding bone density

D. Understanding the condition of the injury

E. Judging the prognosis of the fracture

62. The treatment plan for enchondroma is

A. Curettage of the lesion and autologous bone grafting

B. Tumor segment resection

C. Artificial joint replacement surgery if necessary

D. Amputation

E. Combination of radiotherapy, chemotherapy, and surgery

63. Regarding the principles and procedures of limb (finger) replantation surgery, the incorrect one is

A. First, anastomose arteries and veins to quickly restore blood circulation

B. Perform thorough debridement surgery, and thoroughly remove all poorly circulated muscles

C. When ischemia time is long, heparin saline can be used to perfuse the vascular bed of the severed limb (finger)

D. The number of vascular anastomoses should be as many as possible, with an arterial to venous ratio of 1:2

E. Shorten and fix the bones to restore their support function

64. Regarding the clinical treatment of Ewing's sarcoma, the incorrect one is

A. Not sensitive to radiotherapy

B. Mainly adopts comprehensive treatment

C. Surgery is the main treatment method

D. Due to early metastasis, the long-term efficacy of radiotherapy or chemotherapy alone is poor

E. Depending on the patient's condition and surgical indications, surgery can be limb-sparing or amputation

65. The simplest and most effective method for hemostasis of hand trauma bleeding is

A. Clamping the blood vessel

B. Suture ligation to stop bleeding

C. Using a pneumatic tourniquet for hemostasis

D. Ligation of major blood vessels is necessary

E. Local dressing and pressure hemostasis

66. Regarding the treatment methods of acute suppurative arthritis, the incorrect one is

A. Combined application of adequate sensitive antibiotics

B. Local immobilization and supportive treatment

C. Daily aspiration, irrigation, and drug injection for superficial joints

D. Open drainage and closed irrigation for deep joints

E. Immediate open reduction for acute pathological dislocation

67. The indications for anterior spinal fusion surgery for scoliosis do not include

A. Severe scoliosis with poor flexibility

B. Accompanied by kyphotic deformity

C. Severe rotational deformity or unsuitability for posterior correction

D. Age <10 years, open triradiate cartilage, Risser sign <0°

E. Rotational deformity accompanied by kyphoscoliosis

68. The clinical manifestations of scoliosis do not include

A. Asymmetry of the back when bending forward

B. Skin folds on one side of the waist

C. Deviation of the spine from the midline

D. Unequal height of the shoulders and scapulae

E. Inward concavity of the lateral part of the costal cartilage and ribs

69. Carpal tunnel syndrome is caused by compression of which structure within the carpal tunnel

A. Ulnar nerve

B. Ulnar artery

C. Radial nerve

D. Median nerve

E. Radial artery

70. Regarding the clinical characteristics of cubital tunnel syndrome, the incorrect one is

A. It is a chronic injury to the ulnar nerve

B. The most common cause is cubitus valgus

C. Numbness of the ulnar side of the back of the hand, hypothenar, little finger, and ulnar side of the ring finger is often the first symptom

D. Surgery is required

E. Atrophied small muscles of the hand can recover quickly after surgery, while hand sensation can slowly return to normal

71. Patient Sun, male, 28 years old. He visited the urology outpatient department of a hospital because of pus discharge from the urethra. After examination, he was diagnosed with gonorrhea. The doctor informed the patient that it was a sexually transmitted disease and prescribed spectinomycin injections. The doctor also explained the "Law on the Prevention and Control of Infectious Diseases" and required the patient to report the gonorrhea case to the disease control agency and stated that his wife also needed to be checked at the hospital. Upon hearing this, the patient immediately begged the doctor not to report his condition or inform his wife, fearing it would ruin his family. The doctor's best choice is:

A. Report to the disease control agency and inform the patient's wife

B. Neither report to the disease control agency nor inform the patient's wife

C. Report to the disease control agency and have the patient persuade his wife to get checked

D. Do not report to the disease control agency, only inform the patient's wife

E. Only report to the disease control agency, do not inform the patient's wife

72. Male, 26 years old, suffered a fracture of the left humeral shaft due to trauma, with the upper arm shorter by 4 cm compared to the opposite side and mild angular deformity. Auscultation revealed significantly reduced bone conduction sound. The type of fracture displacement is:

A. Lateral displacement

B. Mixed displacement

C. Shortening displacement

D. Angular displacement

E. Separation displacement

73. Female, 34 years old, injured her left chest in a car accident 2 hours ago, complaining of difficulty breathing. Physical examination: cyanosis of the lips, no paradoxical breathing movement on the left anterior chest, obvious swelling and tenderness, decreased breath sounds in the left lung, X-ray showed fractures of the posterior ends of the left 8th and 9th ribs. Which of the following treatments is not appropriate?

A. Clear respiratory secretions

B. Sedation and pain relief

C. Use of antibiotics

D. Traction fixation

E. Chest band fixation

74. Female patient, 41 years old. Two years after undergoing debridement surgery for tuberculosis of the 7th and 8th thoracic vertebrae, she developed symptoms of night sweats, low-grade fever, back pain, and general fatigue over the past three months. Physical examination: obvious percussion pain at the 7th and 8th thoracic vertebrae, muscle strength in both lower limbs at grade 3, positive Babinski reflex, erythrocyte sedimentation rate (ESR) of 35 mm/h. The most appropriate treatment is:

A. Non-surgical treatment, bed rest with plaster bed

B. Mainly non-surgical treatment, with debridement surgery if necessary

C. Emergency debridement surgery

D. Standard anti-tuberculosis treatment, followed by early debridement surgery

E. Posterior spinal fusion

75. Female patient, 30 years old. Three hours after cervical spine trauma, physical examination revealed complete paralysis of all four limbs with loss of sensation, movement, and reflexes, but normal sensation above the acromion. The likely site of injury is:

A. Between C2 and C3 cervical vertebrae

B. Between C3 and C4 cervical vertebrae

C. Between C4 and C5 cervical vertebrae

D. Between C5 and C6 cervical vertebrae

E. Between C6 and C7 cervical vertebrae

76. Female, 16 years old, with a mass below the right knee that has gradually enlarged over the past 5 years without pain and with normal gait. X-ray showed a mass on the upper inner side of the right tibia, connected to the tibia by trabecular bone at the base, with decreased density at the top and clear boundaries. The most likely diagnosis is:

A. Osteosarcoma

B. Giant cell tumor of bone

C. Osteoma

D. Osteochondroma

E. Metastatic bone cancer

77. A patient developed neck pain after a neck injury, with normal sensory and motor function in all four limbs. X-ray showed no cervical fractures or dislocations. No treatment was given, but one month after the injury, the patient developed numbness and weakness in the right upper limb, suggesting delayed spinal cord injury. The most likely cause is:

A. Vascular compression

B. Vascular embolism

C. Tissue proliferation

D. Local instability

E. Ligament calcification

78. Male patient, 25 years old, with a closed fracture of the left femoral shaft caused by trauma, with no obvious displacement on X-ray, showing a transverse fracture. The first choice of treatment is:

A. Manual reduction, small splint fixation with weight traction

B. Manual reduction, plaster fixation

C. External fixator fixation

D. Open reduction and internal fixation

E. Bed rest with strict immobilization

79. Male patient, young adult. Knocked down by a car, right hip impacted the ground, could stand up but after walking 10 meters, could not move due to pain. The right hip is in a flexed, shortened, and externally rotated position. The most appropriate treatment is:

A. Bed rest

B. Small splint fixation

C. Plaster fixation

D. Pain relief

E. Surgical treatment

80. Male patient, 20 years old, developed a waddling gait after a right thigh amputation and prosthesis fitting, requiring resistance exercises for which muscle to counteract hip external rotation deformity:

A. Iliopsoas

B. Adductor muscles

C. Sartorius

D. Gluteus maximus

E. Gluteus medius and minimus

81. How should a 4-year-old child with a femoral shaft fracture be treated?

A. Posterior plaster splint

B. Skeletal traction

C. Horizontal skin traction

D. Suspension skin traction

E. Elastic nail fixation

82. Male, 20 years old, sustained a left knee injury while playing soccer, with pain and swelling on the medial side of the joint and restricted movement. After one month of conservative treatment, symptoms reduced but occasional joint locking and buckling occurred. The medial head of the quadriceps muscle is significantly atrophied, with tenderness in the medial joint space, positive McMurray test, negative drawer test, and negative valgus stress test. The most likely diagnosis is:

A. Medial collateral ligament rupture

B. Medial meniscus injury

C. Anterior cruciate ligament rupture

D. Intra-articular loose body

E. Osteomalacia

83. Male patient, 24 years old. Open tibia and fibula fractures with soft tissue injury for 4 hours caused by a car accident. The key point of emergency surgery is:

A. Reduction and internal fixation of tibia and fibula fractures

B. Reduction and internal fixation of tibia fracture

C. Reduction and internal fixation of fibula fracture

D. Thorough debridement to ensure primary wound healing

E. Skeletal traction, waiting for secondary wound healing

84. Male, 70 years old. Left lower extremity pain for 2 years, worsened for 1 month. History of rheumatoid arthritis for more than 10 years. Physical examination: normal lumbar lordosis, normal flexion, positive lumbar extension test, reduced sensation on the lateral side of the left calf, muscle strength of left extensor digitorum longus at grade 4. X-ray: mild narrowing of L4-5 intervertebral space, positive straight leg raise test at 35° on the left. The most likely diagnosis is:

A. Lumbar muscle strain

B. Lumbar spondylosis

C. Spondylolysis

D. L4-5 disc herniation

E. Thromboangiitis obliterans

85. Male patient, 56 years old. Right knee pain for half a year, worsened in the last 5 days, especially when climbing stairs. X-ray showed degenerative changes in the knee joint. Diagnosed with chondromalacia patellae. The initial period for non-surgical treatment with knee immobilization should be:

A. 1-2 weeks

B. 3-4 weeks

C. 4-5 weeks

D. 6-7 weeks

E. 8-9 weeks

86. A researcher investigating the causes of neonatal jaundice selected 100 infants with the condition and 100 healthy newborns from the same hospital. The researcher then reviewed the prenatal care and delivery records of the mothers to identify various exposure factors. This study is:

A. Case-control study

B. Ecological comparison study

C. Cross-sectional study

D. Cohort study

E. Clinical trial

87. Female patient, 40 years old. Tibial plateau or femoral condyle fracture with an uneven joint surface, differing by 0.8 cm. After treatment, the fracture displacement did not change. The most likely late complication is:

A. Myositis ossificans

B. Poor bone healing

C. Traumatic osteoarthritis of the knee

D. Knee joint stiffness

E. Genu valgum deformity

88. Male child, 7 years old. Clinically diagnosed with right torticollis. Physical examination revealed a mass in the right sternocleidomastoid muscle. X-ray showed normal cervical vertebrae. The recommended treatment is:

A. Mass excision

B. Plaster orthosis

C. Complete resection of the sternocleidomastoid muscle

D. Physical therapy

E. Sectioning of the sternocleidomastoid muscle above the clavicle and plaster orthosis

89. Male, 25 years old, right knee sprain 2 weeks ago, feeling unstable with a sensation of "giving way" while walking. Which test can diagnose cruciate ligament injury?

A. Positive drawer test

B. Positive valgus/varus stress test

C. Positive hyperextension/hyperflexion test

D. Positive patellar ballottement test

E. Positive McMurray test

90. Male, right thumb cut injury with a skin defect of about 1 cm x 2 cm, exposing the tendon. The treatment should be:

A. Free skin graft

B. Z-plasty

C. Direct skin suture

D. Shorten the phalanx and suture the skin

E. Pedicled flap graft

91. Female patient, 41 years old, hit on the right knee with a blunt object. X-ray showed fibular head fracture. The patient cannot actively dorsiflex the ankle, suggesting:

A. Common peroneal nerve injury

B. Sciatic nerve injury

C. Tibial nerve injury

D. Anterior tibialis muscle rupture

E. Gastrocnemius muscle tear

92. Female child, 8 years old, right arm pain for 2 months, often with fever around 38°C, high white blood cell count, erythrocyte sedimentation rate of 32 mm/h. X-ray showed cortical bone destruction of the right humeral shaft with a moth-eaten appearance and onion skin changes. The most likely diagnosis is:

A. Acute pyogenic osteomyelitis

B. Chronic pyogenic osteomyelitis

C. Tuberculosis of the bone shaft

D. Ewing's sarcoma

E. Reticulum cell sarcoma

93. Female patient, 68 years old, sustained a pelvic fracture with extensive soft tissue degloving injury and shock caused by an earthquake. After rescue, fluid resuscitation, and emergency debridement and pelvic external fixation surgery were performed. The most indicative parameter for monitoring the effectiveness of shock treatment post-surgery is:

A. Mental state

B. Skin temperature

C. Pulse rate

D. Blood pressure

E. Urine output

94. A patient developed neck stiffness after a head injury. To determine if there is a fracture of the atlas arch, which X-ray view is diagnostic?

A. Anteroposterior view

B. Lateral view

C. Oblique view

D. Open-mouth view

E. Tangential view

95. Male patient, 56 years old. Right knee pain for half a year, worsened in the last 5 days, especially when climbing stairs. X-ray showed degenerative changes in the knee joint. Diagnosed with chondromalacia patellae. The initial period for non-surgical treatment with knee immobilization should be:

A. 1-2 weeks

B. 3-4 weeks

C. 4-5 weeks

D. 6-7 weeks

E. 8-9 weeks

96. Female, 20 years old, 3 hours after cervical spine injury with C4 and C5 fractures and spinal cord injury, presenting with flaccid paralysis of all four limbs and high fever of 40°C persisting for several days. The appropriate cooling method is:

A. Hibernation therapy

B. Oral aspirin

C. Antibiotics

D. Physical cooling

E. None of the above

97. Female patient, 68 years old, with osteoarthritis of the left knee, flexion contracture of 30°, and varus deformity of 10°, underwent total knee arthroplasty. Postoperatively, she experienced numbness in the dorsal foot and weakness in ankle dorsiflexion. The likely complication is:

A. Prosthesis dislocation

B. Tight bandaging

C. Anterior tibial artery injury

D. Common peroneal nerve palsy

E. Tibial nerve injury

98. Male, 18 years old, intermittent pain and swelling in the middle of the left thigh with fever for 3 years, no episodes in the last 8 months. X-ray diagnosed as chronic osteomyelitis of the femur. Physical examination: body temperature 36.6°C, no redness and swelling locally, mild deep tenderness. X-ray showed thickened cortex at the junction of the upper and middle third of the femur with a dead space of 30 mm x 25 mm x 25 mm and a sequestrum of 20 mm x 15 mm x 20 mm. The most appropriate treatment is:

A. Local hot compresses, oral antibiotics

B. Sequestrectomy, open dressing change

C. Sequestrectomy and saucerization

D. Sequestrectomy, muscle flap filling, and wound closure

E. Sequestrectomy, closed irrigation-suction therapy

99. Female, 36 years old, underwent surgery for uterine fibroids at a county hospital. Postoperatively, dissatisfied with the surgical outcome, the patient sued the hospital. The court found that the hospital had committed a presumed fault under the Tort Liability Law and ruled against the hospital. This presumed fault is:

A. Failure to provide medical explanation

B. Failure to fulfill diagnostic and treatment obligations corresponding to the current medical level

C. Falsification of medical records

D. Disclosure of patient privacy

E. Difficulty in diagnosis and treatment due to limited medical level at that time

100. A patient with a forearm fracture experienced severe pain, finger numbness, swelling, and restriction of movement 5 hours after manual reduction and small splint fixation. The main cause is:

A. Nerve injury

B. Nerve compression and venous compression

C. Arterial compression and venous compression

D. Venous compression

E. Arterial compression

101. An elderly male, 65 years old, with an old femoral neck fracture and avascular necrosis of the femoral head. The best treatment method is:

A. Hip joint fusion

B. Intertrochanteric rotational osteotomy

C. Femoral head drilling and bone grafting

D. Total hip arthroplasty

E. Medication therapy

102. Patient Li, suffering from severe right lower abdominal pain, was diagnosed with acute appendicitis after examination and tests. During the operation, the surgeon found that the wound healing was poor with yellowish fluid leakage. The surgeon explained that it was due to non-absorption of catgut, which is rare clinically. After nearly a month of continued treatment, the patient fully recovered. According to the "Regulations on the Handling of Medical Accidents," the objective result of Li's delayed recovery by nearly a month should be classified as:

A. Grade II medical accident

B. Grade III medical accident

C. Grade IV medical accident

D. Medical incident due to the patient's special constitution

E. Adverse outcome caused by force majeure

103. A 5-year-old boy suddenly developed chills, a temperature of 39°C, and severe pain in the right knee, avoiding movement, without obvious swelling. The first consideration should be:

A. Chronic osteomyelitis

B. Suppurative arthritis

C. Rheumatoid arthritis

D. Acute hematogenous osteomyelitis

E. Tibial tubercle osteochondritis

104. A 54-year-old male patient with an open fracture of the middle and lower third of the tibia and fibula, three months post-treatment. X-ray shows slight displacement of the tibial fracture, and the fracture line is clear. The most important factor affecting fracture healing is:

A. Age

B. Insufficient blood supply to the fracture site

C. Wound infection

D. Severe contusion of surrounding soft tissues

E. Poor fracture reduction

105. A 36-year-old male with the tip of the right middle finger cut off by a machine 4 hours ago, with the bone exposed. The least suitable treatment method is:

A. Shortening the bone and suturing the skin

B. Free skin grafting

C. Pedicled flap grafting

D. Bilateral advancement flap formation

E. Temporarily bandaging and observing if the wound is severely contaminated

106. A 22-year-old male with lower back pain radiating to the buttocks for 10 months. Physical examination reveals stiff lower back with no neurological abnormalities. Laboratory tests show decreased hemoglobin and increased erythrocyte sedimentation rate. HLA-B27 positive. X-ray shows "bamboo spine" changes and narrowed sacroiliac joint space. The primary diagnosis should consider:

A. Rheumatoid arthritis

B. Spinal tuberculosis

C. Ankylosing spondylitis

D. Lumbar disc herniation

E. Rheumatoid arthritis

107. A 24-year-old male fell from a height of 8 meters, landing on his buttocks, and was admitted 12 hours later with numbness below three fingers below the navel, loss of voluntary movement in both lower limbs except for slight movement in the right big toe, urinary retention, and T10 vertebral compression fracture with bone fragments protruding into the spinal canal. The key early treatment is:

A. Aggressive antibiotic therapy

B. Catheterization with a retained catheter

C. Proper nursing care to prevent bedsores

D. Early surgery to relieve compression

E. Realignment with plaster vest application

108. An elderly female patient was admitted with "recurrent chest tightness and pain for over a year, worsened for 4 hours." ECG shows ST elevation in leads V1-V4, and TNI levels are elevated, diagnosed as "acute ST-segment elevation myocardial infarction." After clinical pathway assessment, she was enrolled in the "acute ST-segment elevation myocardial infarction interventional treatment" pathway but discharged without interventional treatment due to economic reasons. The basis for the patient's exit from the clinical pathway is:

A. Serious complications requiring a change in the original treatment plan during the clinical pathway

B. Patient request for discharge during the clinical pathway

C. Patient request to change treatment method during the clinical pathway

D. Patient request for transfer during the clinical pathway

E. Incorrect diagnosis leading to the patient's entry into the clinical pathway

109. A 16-year-old female with acute hematogenous osteomyelitis of the left femur treated with antibiotics for over 2 months, generally stable condition, with two sinuses on the inner thigh that intermittently heal. Suddenly developed fever, rapid pulse, and significantly elevated white blood cell count with 89% neutrophils. X-ray shows thickened femur with uneven density, new bone encasing dead bone and cavities. The appropriate treatment is:

A. Removal of dead bone, converting the cavity into a saucer shape

B. Removal of dead bone, curettage of the sinus, and wound opening

C. Thorough removal of dead bone, excision of the sinus, and wound closure

D. Removal of dead bone and muscle flap filling of the cavity

E. Adequate antibiotic treatment and local incision and drainage

110. A 30-year-old male with a left lower limb injury near the groin with profuse bleeding at the scene. After immediate hemostasis measures, the first examination should be:

A. Palpation of the dorsalis pedis and posterior tibial arteries

B. Check for limb sensation

C. Check knee or Achilles tendon reflex

D. Check for active movement of the foot

E. Immediate X-ray examination

111. A 7-year-old female suddenly slipped while being held by her mother, causing radial head dislocation, ulnar fracture, and radial nerve deep branch injury. Physical examination should reveal:

A. Inability to abduct the thumb

B. Loss of skin sensation in the thenar eminence

C. Difficulty extending the metacarpophalangeal joints

D. Wrist extension difficulty

E. Inability to adduct the thumb

112. A 9-year-old male with right hip pain and limp, low fever, night sweats, and poor appetite for 3 weeks. Physical examination: 37.7°C, limited right hip movement, positive Thomas sign. X-ray: slightly narrowed right hip joint space with marginal bone destruction. The primary diagnosis should be:

A. Avascular necrosis of the femoral head

B. Acute suppurative arthritis

C. Acute osteomyelitis

D. Osteoarthritis

E. Hip tuberculosis

113. An 8-year-old boy with a history of high fever and convulsions at 8 months old, now presents with left lower limb limp, muscle atrophy, and typical walking posture. Clinical examination reveals weakened gluteal muscles. The appropriate surgery is:

A. Gluteal muscle release

B. Sacrospinalis to gluteal muscle transfer

C. Iliopsoas tendon release

D. Adductor muscle release

E. Obturator nerve anterior branch release

114. A newborn with a firm mass in the right neck, head tilted to the right, and chin turned to the left, diagnosed as congenital muscular torticollis. The optimal time for surgery is:

A. 1 year old

B. 3 years old

C. 8 years old

D. 6 years old

E. 6 months old

115. A 45-year-old female diagnosed with mixed cervical spondylosis for 3 years, now presenting with severe left shoulder pain and significant movement restriction. The cause of shoulder pain is:

A. Shoulder tumor

B. Frozen shoulder

C. Cervical spondylosis

D. Shoulder tuberculosis

E. Rheumatoid arthritis

116. A 24-year-old male with a left chest gunshot wound, X-ray shows the disappearance of the left costophrenic angle with the bullet lodged in the abdominal cavity. The primary diagnosis is:

A. Left lung injury

B. Left diaphragm injury

C. Left chest injury

D. Left thoracoabdominal injury

E. Left abdominal injury

117. A 56-year-old male with neck and shoulder pain radiating to the right hand, decreased pain sensation in the right thumb, and weakness in the biceps. The primary diagnosis is:

A. Cervical spondylosis

B. Frozen shoulder

C. Rotator cuff syndrome

D. Brachial plexus neuritis

E. Neck strain

118. An 18-month-old female with sudden episodic crying and limb movement, pale complexion, and refusal to eat. After 4 hours, she passes red jelly-like stool. Physical examination during a crying episode reveals a sausage-shaped mass in the right lower abdomen. The most likely diagnosis is:

A. Congenital hypertrophic pyloric stenosis (CHPS)

B. Intestinal spasm

C. Acute necrotizing enterocolitis

D. Cecal volvulus

E. Intussusception

119. A 20-year-old male with a right thigh mass for 2 months. During physical examination, the incorrect principle is:

A. Examine the healthy side first

B. Examine the distal part before the affected part

C. Perform passive movements before active movements

D. Compare both sides when exposed

E. Gentle movements to minimize patient discomfort

120. A 56-year-old male with sudden hematemesis of about 800 ml after a 20-year history of heavy drinking. Physical examination: P110/min, BP 90/70 mmHg, liver and spleen not palpable. Laboratory tests: RBC 3.0×10¹²/L, Hb 70g/L, PLT 56×10⁹/L. The most likely diagnosis is:

A. Hemorrhagic gastr

itis

B. Bleeding peptic ulcer

C. Esophagogastric variceal bleeding

D. Gastric cancer bleeding

E. Biliary bleeding

121. A 38-year-old male with left-sided back pain for 1 year, worsened after activity, relieved by rest. Recent urinalysis shows 5 RBC/HP. For further diagnosis and treatment of upper urinary tract stones, the preferred test is:

A. Ultrasound

B. Kidney-ureter-bladder (KUB) X-ray

C. Intravenous pyelogram (IVP)

D. KUB + IVP

E. CT

122. A 20-year-old male with right shoulder pain for 3 hours after a fall. Physical examination: swelling and tenderness at the right acromioclavicular joint, no abnormality on X-ray. The correct management is:

A. Sling

B. Figure-eight bandage

C. No treatment

D. Bed rest for 2 weeks

E. Small splint

123. A 28-year-old male with a fracture of the left forearm radius and ulna. Examination: inability to oppose the thumb. The most likely nerve injury is:

A. Ulnar nerve

B. Radial nerve

C. Median nerve

D. Axillary nerve

E. Musculocutaneous nerve

124. A 53-year-old female with acute diffuse peritonitis. After 2.5 hours of observation, her condition worsens with abdominal distension and persistent pain. The immediate treatment does not include:

A. Fasting and gastrointestinal decompression

B. High-dose sensitive antibiotics

C. Immediate exploratory laparotomy

D. Antispasmodics and pain relief

E. Fluid resuscitation and correction of acidosis

125. A 31-year-old female with lateral knee trauma. Physical examination: inability to dorsiflex the ankle. X-ray: fracture of the left fibular head. The correct diagnosis is:

A. Left fibular head fracture with tibial nerve injury

B. Left fibular head fracture with sciatic nerve injury

C. Left fibular head fracture with common peroneal nerve injury

D. Left fibular head fracture with anterior tibial muscle injury

E. Left fibular head fracture with fibular longus and brevis muscle tear

126. A 44-year-old female with pain and snapping at the base of the right thumb for a year, worsening for 2 weeks. Physical examination: palpable nodule with tenderness, snapping on flexion and extension of the thumb. The most likely diagnosis is:

A. Ganglion cyst

B. Dermoid cyst

C. Neuroma

D. Stenosing tenosynovitis

E. Synovial tumor

127. A 30-year-old female with lateral elbow pain for six months. Physical examination: positive Mills test on the right. No abnormalities on X-ray. The key to treatment and preventing recurrence is:

A. Functional exercise

B. Early surgery

C. Limiting wrist joint activity

D. Medication therapy

E. Conservative treatment

128. A male with a fracture of the right humeral shaft, with shortening of 4 cm and slight angulation, decreased bone conduction sound. The most likely displacement of the fracture is:

A. Shortening displacement

B. Mixed displacement

C. Lateral displacement

D. Angular displacement

E. Separation displacement

129. A 72-year-old female with knee pain for over 10 years, worsened for 3 months. Physical examination: no swelling, joint space tenderness, crepitus on movement. X-ray: narrowed joint space, osteophytes around the joint. The most likely diagnosis is:

A. Rheumatoid arthritis

B. Osteoarthritis

C. Osteoporosis

D. Ankylosing spondylitis

E. Gout

130. A 5-year-old male with lumbar tuberculosis for 1 month. The test that is likely positive is:

A. McMurray test

B. Thomas sign

C. Gaenslen test

D. Picking up test

E. Dugas sign

131. An 18-year-old female with low fever, night sweats, and lumbar pain for 2 months. A mass in the right iliac fossa, confirmed to be fluid-filled by ultrasound, with limited right hip flexion. The primary diagnosis is:

A. Metastatic spinal tumor

B. Pelvic tumor

C. Lumbar tuberculosis

D. Knee tuberculosis

E. Hip tuberculosis

132. A 38-year-old male with recurrent left lumbar pain radiating to the left heel for 3 years. MRI shows L5-S1 disc protrusion compressing the nerve root. The sign that is not expected is:

A. Decreased sensation on the lateral side of the left foot and ankle

B. Positive straight leg raising test on the left side

C. Weakness in the left calf triceps

D. Tenderness between L5-S1 with radiation to the left lower limb

E. Decreased knee reflex on the left side

133. A patient stabbed in the left groin 1 hour ago with profuse bleeding. The first examination should be:

A. Check for active foot movement

B. Check knee or Achilles tendon reflex

C. Check vital signs and palpate the dorsalis pedis and posterior tibial arteries

D. Check for limb sensation loss

E. Immediate X-ray examination

134. A 20-year-old male with a middle third clavicle fracture from a fall, presenting with the head tilted to the right and supporting the right elbow with the left hand. The main reason for this posture is:

A. Adopting a forced position

B. Clavicle fracture with brachial plexus injury and decreased right upper limb strength

C. Clavicle fracture with subclavian vessel injury, reducing upper limb ischemia

D. Temporary immobilization to reduce fracture end bleeding

E. Muscle relaxation and reduced traction pain on the fracture ends

135. An 8-year-old male with a cystic mass in the scrotum, increasing when standing and positive transillumination test, disappearing or shrinking when lying down. The possible diagnosis is:

A. Testicular hydrocele

B. Spermatic cord hydrocele

C. Communicating hydrocele

D. Orchitis

E. Complete inguinal hernia

136. A 35-year-old female with headache and unsteady gait for 2 months. Physical examination reveals bilateral optic disc edema and right cerebellar dysfunction. Blood tests show erythrocytosis, renal ultrasound reveals a cystic mass in the right kidney, and cranial CT shows a cystic lesion in the right cerebellar hemisphere. The primary diagnosis is:

A. Hemangioblastoma

B. Astrocytoma

C. Brain abscess

D. Epithelial cyst

E. Metastatic tumor

137. A 58-year-old male with bilateral hip pain and restricted movement for 2 years after long-term corticosteroid use for a skin condition. The primary diagnosis is:

A. Bilateral rheumatoid arthritis

B. Bilateral traumatic synovitis

C. Bilateral degenerative osteoarthritis

D. Bilateral avascular necrosis of the femoral head

E. Bilateral hip dislocation

138. A 36-year-old female with the right thumb amputated by a machine, received debridement after 24 hours. The next step should be:

A. The severed time is too long, replantation should not be attempted due to toxic reactions

B. Fix the fracture and repair the flexor and extensor tendons

C. Fix the fracture and anastomose the arteries and veins

D. Fix the fracture and repair the nerves

E. Anastomose the arteries and veins, then fix the fracture

139. A 32-year-old female with back pain for 5 weeks, worse with activity, accompanied by weight loss, fatigue, and night sweats. Physical examination reveals tenderness and percussion pain in the 7th and 8th thoracic vertebrae. To clarify the diagnosis, which examination is not needed:

A. Complete blood count and erythrocyte sedimentation rate

B. Thoracic spine X-rays

C. Bone scan

D. Tuberculin test

E. Chest X-ray

140. A 51-year-old male with a mid-third tibia and fibula fracture treated with long leg plaster cast immobilization. After 4 months, the cast was removed and knee joint dysfunction was observed. The most likely cause is:

A. Muscle atrophy

B. Joint stiffness

C. Joint ankylosis

D. Poor fracture reduction

E. Malunion

141. An 18-year-old male with a right knee and lower leg injury from a car accident, presenting with foot drop, inversion, adduction dysfunction, and loss of plantar sensation. The most likely injured nerve is:

A. Sural nerve

B. Femoral nerve

C. Common peroneal nerve

D. Tibial nerve

E. Sciatic nerve

142. A male patient with a duodenal ulcer and pyloric obstruction for 2 years, vomiting large amounts of food every night. Physical examination shows emaciation, loose skin, and dry skin, with serum sodium at 128 mmol/L. The patient's condition suggests:

A. Isotonic dehydration

B. Hypotonic dehydration

C. Hypertonic dehydration

D. Dilutional hyponatremia

E. Mixed dehydration

143. A 9-year-old male with a hard mass on the left lower leg discovered during a bath, slightly tender, with slightly elevated skin temperature. The disease's malignant features do not include:

A. Local pain and swelling

B. X-ray showing enlarged tumor and undefined margins

C. Increased thickness and altered shape of the cartilage cap

D. Solitary disease with a 10% chance of malignancy

E. Calcification of the cartilage cap

144. A 20-year-old male with a scaphoid fracture of the left wrist, treated with closed reduction and cast immobilization for 6 months. X-ray shows nonunion with sclerotic fracture ends. The possible cause is:

A. Poor local blood supply

B. Soft tissue interposition at the fracture ends

C. Severe soft tissue injury

D. Inappropriate treatment

E. Advanced patient age

145. A 4-year-old male with a transverse fracture of the mid-femoral shaft and overlapping fracture ends. The appropriate treatment method is:

A. Vertical suspension skin traction

B. Closed reduction and small splint fixation

C. Closed reduction and small splint fixation with skin traction

D. Open reduction and internal fixation

E. Closed reduction and skeletal traction

146. A 17-year-old male with wrist laceration and secondary wound healing, presenting with claw deformity, significant hand muscle atrophy, loss of sensation in the fingers, and loss of thumb opposition function. The most likely diagnosis is:

A. Ulnar nerve injury

B. Median nerve injury

C. Radial nerve injury

D. Combined ulnar and median nerve injury

E. Combined radial and median nerve injury

147. A 28-year-old male with a wrist laceration 3 months ago, presenting with claw deformity, significant hand muscle atrophy, sensory loss in the ulnar one and a half fingers, and loss of thumb opposition function. The most likely diagnosis is:

A. Median nerve injury

B. Ulnar nerve injury

C. Radial nerve injury

D. Combined ulnar and median nerve injury

E. Combined radial and median nerve injury

148. A 30-year-old male with left knee pain for 5 months, worsening gradually, with difficulty climbing stairs. Physical examination: localized tenderness below the left patella, positive patellar grind test, quadriceps atrophy. X-ray shows no abnormalities, but bone scan shows localized increased uptake in the patella. The most likely diagnosis is:

A. Meniscus injury

B. Patellar ligament injury

C. Cruciate ligament injury

D. Chondromalacia patellae

E. Knee osteoarthritis

149. A 34-year-old male with right chest trauma from a car accident, presenting with difficulty breathing, cyanosis, and paradoxical chest wall movement. The incorrect statement about inspiratory pathophysiology is:

A. Soft chest wall collapse

B. Mediastinum shift to the healthy side

C. Diaphragm on the injured side ascends

D. Venous return obstruction

E. Carbon dioxide retention

150. A middle-aged male with tibial plateau comminuted fracture post-surgery for 10 months, experiencing knee pain during walking. The most likely cause is:

A. Joint stiffness

B. Synovitis

C. Traumatic arthritis

D. Ischemic bone necrosis

E. Acute bone atrophy

151. A 45-year-old female with suspected radiculopathy cervical spondylosis. The least appropriate treatment is:

A. Consider surgery for recurrent episodes

B. Local physical therapy

C. Local pain point block

D. Cervical traction

E. Manual therapy

152. A 34-year-old male with tibia and fibula fracture, treated with closed reduction and cast immobilization. Examination reveals abnormal movement at the fracture site, with X-ray showing medullary canal closure and 3 mm gap at the fracture ends, angulated posteriorly by 15°. The appropriate treatment is:

A. Closed reduction with small splint fixation

B. Closed reduction with cast immobilization

C. Open reduction with internal and external fixation

D. Open reduction with external fixation

E. Open reduction with autologous bone graft and internal fixation

153. A 72-year-old female with left hip pain after a fall, initially able to walk, but pain worsened the next day. Physical examination: mild external rotation of the left lower limb. The most likely diagnosis is:

A. Lumbar strain

B. Traumatic disc herniation

C. Greater trochanter fracture

D. Femoral neck fracture

E. Soft tissue contusion

154. A 55-year-old female with right femoral shaft fracture post-car accident, suddenly developing difficulty breathing and cyanosis. The most likely diagnosis is:

A. Pulmonary infection

B. Fat embolism syndrome

C. Shock

D. Myocardial infarction

E. Pneumothorax

155. A 30-year-old male with a gunshot wound to the upper arm, presenting with wrist drop, inability to extend the fingers, numbness on the dorsum of the thumb, index, and middle fingers, with normal elbow flexion and extension. X-ray shows a bullet-shaped metal foreign body in the humeral shaft without fracture. The most likely injured nerve is:

A. Radial nerve

B. Median nerve

C. Ulnar nerve

D. Brachial plexus

E. Lumbosacral nerve

156. A young male with cervical spine injury and incomplete quadriplegia post-car accident, presenting with elbow flexion but no elbow extension. The injury level is likely:

A. C2-3

B. C3-4

C. C4-5

D. C5-6

E. C6-7

157. A 7-year-old female with 12 days of fever and right knee pain, with swelling and tenderness of the upper tibia, elevated WBC count, and purulent fluid from tibial metaphysis puncture. The correct treatment is:

A. High-dose intravenous penicillin

B. Lesion removal and bone grafting

C. Lesion irrigation and abscess curettage

D. Window decompression surgery

E. Continuous intra-articular antibiotic irrigation

158. An 83-year-old male with subcapital femoral neck fracture (Garden III) after a fall. The most appropriate treatment is:

A. Skin traction

B. Hip spica cast

C. Skeletal traction

D. Intertrochanteric osteotomy

E. Hemiarthroplasty

159. A 10-year-old male with left arm pain for 1 week post-fall. X-ray shows a clear radiolucent lesion in the proximal humeral metaphysis with cortical thinning and no periosteal reaction. The primary diagnosis is:

A. Osteochondroma

B. Osteosarcoma

C. Osteomyelitis

D. Bone cyst

E. Giant cell tumor

160. An 18-year-old female with fatigue and a left chest wall mass for 6 months, with recent enlargement, pain, and low-grade fever. Physical examination reveals a soft mass with reddish skin overlying it. Chest X-ray is normal. The primary diagnosis is:

A. Tuberculous chest wall infection

B. Subcutaneous abscess

C. Rib tumor

D. Rib osteomyelitis

E. Accessory breast

161. A 20-year-old female with right lower leg pain and fever for 1 week. X-ray shows tibial destruction with periosteal "onion skin" reaction. Clinical diagnosis is Ewing's sarcoma, but differentiation is needed with:

A. Tuberculous osteomyelitis

B. Pyogenic osteomyelitis

C. Bone cyst

D. Fibrosarcoma

E. Fibrous dysplasia

162. A 20-year-old female with right lower leg pain and fever for 1 week. X-ray shows tibial destruction with periosteal "onion skin" reaction. The most valuable diagnostic test is:

A. Plain X-ray

B. Bone scan

C. CT

D. WBC count and differential

E. Biopsy and pathology

163. A 20-year-old female with right lower leg pain and fever for 1 week. X-ray shows tibial destruction with periosteal "onion skin" reaction. The appropriate treatment is:

A. Amputation with chemotherapy

B. Radiotherapy

C. Amputation with radiotherapy and chemotherapy

D. Amputation with radiotherapy

E. Segmental resection and inactivation replantation

164. A critically ill patient experienced rapid breathing and reduced right breath sounds after a successful right internal jugular vein catheterization. The primary diagnosis is:

A. Pneumothorax

B. Hemothorax

C. Cardiac tamponade

D. Brachial plexus injury

E. Air embolism

165. A critically ill patient experienced rapid breathing and reduced right breath sounds after a successful right internal jugular vein catheterization. The further necessary examination is:

A. Complete blood count

B. Cranial CT

C. Chest X-ray

D. Psychological comfort

E. Chest CT

166. A 70-year-old female with lower back pain for over 3 months, without lower limb radiation pain. Physical examination: L3-L4 spinous process tenderness, negative straight leg raising test. X-ray shows biconcave vertebral bodies with fish tail deformity. The likely abnormal examination finding is:

A. Elevated ESR

B. Complete blood count

C. Ultrasound bone density measurement

D. HLA-B27

E. HLA-DR4

167. Female, 70 years old, with no obvious cause of lower back pain for more than 3 months, not accompanied by radiating pain in the lower limbs. Physical examination: tenderness in the spinous processes of L3 and L4, straight leg raising test (-), normal muscle strength and sensation in the lower limbs. X-ray shows biconcave, fish-tail deformities in the L3 and L4 vertebral bodies.

Q: The possible diagnosis is

A. Postmenopausal osteoporosis

B. Lumbar disc herniation

C. Lumbar compression fracture

D. Lumbar spinal stenosis

E. Lumbar tumor

168. Female, 70 years old, with no obvious cause of lower back pain for more than 3 months, not accompanied by radiating pain in the lower limbs. Physical examination: tenderness in the spinous processes of L3 and L4, straight leg raising test (-), normal muscle strength and sensation in the lower limbs. X-ray shows biconcave, fish-tail deformities in the L3 and L4 vertebral bodies.

Q: Which treatment measure is the most effective

A. Bed rest

B. Local heat application + physiotherapy

C. NSAIDs

D. NSAIDs + Estrogen

E. NSAIDs + Estrogen + Calcium supplements

169. Male, 50 years old, has been working as a diver for 22 years, with right hip pain for the past year. The pain is temporarily relieved by taking NSAIDs, but then worsens and becomes persistent, gradually leading to limping and difficulty walking.

Q: The most likely diagnosis is

A. Osteoarthritis

B. Ankylosing spondylitis

C. Rheumatoid arthritis

D. Avascular necrosis of the femoral head

E. Hip synovitis

170. Male, 50 years old, has been working as a diver for 22 years, with right hip pain for the past year. The pain is temporarily relieved by taking NSAIDs, but then worsens and becomes persistent, gradually leading to limping and difficulty walking.

Q: The most significant imaging examination for early diagnosis is

A. Plain X-ray

B. CR

C. CT

D. 3D CT reconstruction

E. MRI

171. Male, 50 years old, has been working as a diver for 22 years, with right hip pain for the past year. The pain is temporarily relieved by taking NSAIDs, but then worsens and becomes persistent, gradually leading to limping and difficulty walking.

Q: If imaging examination shows no obvious abnormalities, the best surgical procedure is

A. Core decompression of the femoral head

B. Vascular bundle and cancellous bone grafting

C. Trans-trochanteric rotational osteotomy

D. Hip fusion

E. Total hip arthroplasty

172. Male, 50 years old, has been working as a diver for 22 years, with right hip pain for the past year. The pain is temporarily relieved by taking NSAIDs, but then worsens and becomes persistent, gradually leading to limping and difficulty walking.

Q: If imaging shows cystic changes in the femoral head, the best surgical procedure is

A. Core decompression of the femoral head

B. Vascular bundle and cancellous bone grafting

C. Trans-trochanteric rotational osteotomy

D. Hip fusion

E. Total hip arthroplasty

173. Male, 29 years old, suffered right lower leg swelling, pain, bleeding, and restricted movement due to a car accident 3 hours ago. Physical examination: vital signs stable, a 13 cm laceration on the right lower leg with exposed tibial fracture ends, severe soft tissue contusion but no significant defect, minimal bleeding, dorsalis pedis artery palpable.

Q: For such a trauma patient, the first physical examination to be performed is

A. Measurement

B. Palpation

C. Motion examination

D. Inspection

E. Auscultation

174. Male, 29 years old, suffered right lower leg swelling, pain, bleeding, and restricted movement due to a car accident 3 hours ago. Physical examination: vital signs stable, a 13 cm laceration on the right lower leg with exposed tibial fracture ends, severe soft tissue contusion but no significant defect, minimal bleeding, dorsalis pedis artery palpable.

Q: Before performing auxiliary examinations, the initial treatment should be

A. Simple external fixation and local dressing

B. Hemostasis with pneumatic tourniquet

C. Emergency transfer to the operating room

D. Plaster fixation

E. Calcaneal traction

175. Male, 29 years old, suffered right lower leg swelling, pain, bleeding, and restricted movement due to a car accident 3 hours ago. Physical examination: vital signs stable, a 13 cm laceration on the right lower leg with exposed tibial fracture ends, severe soft tissue contusion but no significant defect, minimal bleeding, dorsalis pedis artery palpable.

Q: After the physical examination, the most necessary examination is

A. Anteroposterior and lateral X-rays centered on the middle upper third of the right lower leg, including the knee joint

B. CT

C. Routine blood test

D. Electrophysiological examination

E. Full-length anteroposterior and lateral X-rays of the right lower leg

176. Male, 29 years old, suffered right lower leg swelling, pain, bleeding, and restricted movement due to a car accident 3 hours ago. Physical examination: vital signs stable, a 13 cm laceration on the right lower leg with exposed tibial fracture ends, severe soft tissue contusion but no significant defect, minimal bleeding, dorsalis pedis artery palpable.

Q: According to Gustilo classification, this fracture should be classified as

A. Type I

B. Type II

C. Type IIIa

D. Type IIIb

E. Type IIIc

177. Male, 29 years old, suffered right lower leg swelling, pain, bleeding, and restricted movement due to a car accident 3 hours ago. Physical examination: vital signs stable, a 13 cm laceration on the right lower leg with exposed tibial fracture ends, severe soft tissue contusion but no significant defect, minimal bleeding, dorsalis pedis artery palpable.

Q: The best treatment method is

A. Debridement, fracture reduction, internal fixation with a plate

B. Debridement, fracture reduction, internal fixation with an intramedullary nail

C. Debridement, fracture reduction, external fixation

D. Debridement, fracture reduction, splint fixation

E. Debridement, plaster cast fixation

178. Female, 2 years old. Walks with a waddling gait, both lower limbs are of equal length, Allis sign negative, Ortolani test positive. Pelvic X-ray: femoral head epiphysis significantly smaller, laterally displaced, with subluxation, increased acetabular index.

Q: The first choice of treatment based on the child's condition is

A. Skin traction, reduction, plaster fixation

B. Skeletal traction, reduction, plaster fixation

C. Skin traction, open reduction

D. Skeletal traction, open reduction, pelvic osteotomy

E. Open reduction, pelvic osteotomy

179. Female, 2 years old. Walks with a waddling gait, both lower limbs are of equal length, Allis sign negative, Ortolani test positive. Pelvic X-ray: femoral head epiphysis significantly smaller, laterally displaced, with subluxation, increased acetabular index.

Q: After reduction, if the X-ray shows symmetrical alignment of both femoral heads and acetabula, the duration of plaster fixation should be

A. 3 months

B. 6 months

C. 9 months

D. 1 year

E. 3 months of plaster fixation followed by 3 months of brace fixation

180. Female, 2 years old. Walks with a waddling gait, both lower limbs are of equal length, Allis sign negative, Ortolani test positive. Pelvic X-ray: femoral head epiphysis significantly smaller, laterally displaced, with subluxation, increased acetabular index.

Q: The most severe complication after reduction is

A. Redislocation after reduction

B. Subluxation after reduction

C. Avascular necrosis of the femoral head

D. Acetabular dysplasia

E. Joint dysfunction

181. Male, 60 years old, suffered an open fracture of the middle distal third of the tibia and fibula due to a car accident, underwent open reduction and internal fixation. After 3 months, X-ray shows slight displacement, with the fracture line still visible.

Q: Among the following factors, what is the main reason for the patient's nonunion

A. Poor fracture reduction

B. Severe surrounding soft tissue contusion

C. Advanced age

D. Poor blood supply to the fracture segment

E. Early postoperative activity

182. Male, 60 years old, suffered an open fracture of the middle distal third of the tibia and fibula due to a car accident, underwent open reduction and internal fixation. After 3 months, X-ray shows slight displacement, with the fracture line still visible.

Q: Which of the following is not a complication or sequela of tibia and fibula fractures

A. Nonunion or delayed union

B. Charcot joint

C. Compartment syndrome

D. Refracture

E. Claw toe deformity

183. Female, 25 years old. Gradually developing a lump with aching pain in the outer upper left knee for six months. X-ray: a lesion on the lateral side of the distal left femur, with expansion of the margin, soap bubble-like changes in the center, and no significant periosteal reaction.

Q: The initial diagnosis should consider

A. Fibrous dysplasia

B. Myeloma

C. Osteosarcoma

D. Giant cell tumor of bone

E. Bone cyst

184. Female, 25 years old. Gradually developing a lump with aching pain in the outer upper left knee for six months. X-ray: a lesion on the lateral side of the distal left femur, with expansion of the margin, soap bubble-like changes in the center, and no significant periosteal reaction.

Q: To confirm the diagnosis and grade the tumor, the most valuable examination is

A. Alkaline phosphatase test

B. Local biopsy

C. CT scan

D. Radionuclide bone scan

E. Ultrasound

185. Female, 25 years old. Gradually developing a lump with aching pain in the outer upper left knee for six months. X-ray: a lesion on the lateral side of the distal left femur, with expansion of the margin, soap bubble-like changes in the center, and no significant periosteal reaction.

Q: Based on the patient's condition, the appropriate treatment is

A. Curettage of the lesion and bone grafting

B. Segmental resection and tibial flip bone grafting, knee fusion

C. Segmental resection and allogeneic hemiarthroplasty

D. Segmental resection and vascularized bone grafting

E. Amputation above the knee and fitting with a prosthesis

186. Male, 10 years old. Pain and swelling in the lower right thigh, increased skin temperature, with high fever of 39.5°C. Clinically suspected of acute pyogenic osteomyelitis.

Q: The most valuable auxiliary examination is

A. X-ray

B. Blood culture

C. Local puncture

D. Blood routine test

187. Male, 10 years old. Pain and swelling in the lower right thigh, increased skin temperature, with high fever of 39.5°C. Clinically suspected of acute pyogenic osteomyelitis.

Q: If the diagnosis is confirmed, the most critical treatment measure is

A. Bed rest

B. Symptomatic treatment

C. Limb immobilization

D. Intravenous fluids and nutritional support

E. Use of sensitive antibiotics + drilling and drainage

188. Young male patient, with a lump on his left ankle for over a year, no tenderness, not affecting walking or work. Left ankle X-ray: thinning of the cortical bone at the lower end of the left tibia, no periosteal reaction.

Q: The most likely diagnosis for this patient is

A. Giant cell tumor of bone

B. Benign bone tumor

C. Osteosarcoma

D. Bone tuberculosis

E. Osteochondroma

F. Fibrosarcoma

189. Young male patient, with a lump on his left ankle for over a year, no tenderness, not affecting walking or work. Left ankle X-ray: thinning of the cortical bone at the lower end of the left tibia, no periosteal reaction.

Q: To confirm the diagnosis, additional examinations needed are

A. Elevated serum alkaline phosphatase

B. Radionuclide bone scan

C. MRI, CT scan

D. Local biopsy

E. CT scan

F. AFP test

190. Young male patient, with a lump on his left ankle for over a year, no tenderness, not affecting walking or work. Left ankle X-ray: thinning of the cortical bone at the lower end of the left tibia, no periosteal reaction.

Q: Regarding the epidemiological characteristics of bone tumors, which statement is incorrect

A. Primary benign tumors are more common than malignant tumors

B. Osteosarcoma occurs mostly in children and adolescents

C. More common in females than males

D. Bone tumors commonly occur at the epiphyseal-metaphyseal junction of long bones

E. Osteosarcoma is the most common primary malignant bone tumor

F. Benign bone tumors such as osteochondroma and chondroma are more common

191. Young male patient, with a lump on his left ankle for over a year, no tenderness, not affecting walking or work. Left ankle X-ray: thinning of the cortical bone at the lower end of the left tibia, no periosteal reaction.

Q: Regarding the examination and diagnosis of bone tumors, which statement is incorrect

A. X-ray examination is important for diagnosing bone tumors

B. Routine chest X-ray is unnecessary for malignant bone tumors

C. Onion skin phenomenon is commonly seen in Ewing's sarcoma

D. Codman's triangle is commonly seen in osteosarcoma

E. Soap bubble appearance is common in giant cell tumor of bone

F. Biopsy can help confirm the diagnosis

192. Young male patient, with a lump on his left ankle for over a year, no tenderness, not affecting walking or work. Left ankle X-ray: thinning of the cortical bone at the lower end of the left tibia, no periosteal reaction.

Q: Regarding the clinical manifestations of bone tumors, which statement is correct

A. Common in children

B. Commonly located at the epiphyseal-metaphyseal junction of long bones

C. Slow onset

D. Pain is a characteristic symptom, worse at night

E. The tumor continues to grow after skeletal maturity

F. Pathological fractures can occur

193. Young male patient, with a lump on his left ankle for over a year, no tenderness, not affecting walking or work. Left ankle X-ray: thinning of the cortical bone at the lower end of the left tibia, no periosteal reaction.

Q: Regarding the staging of bone tumors, which statement is incorrect

A. Treatment plans are determined according to surgical staging

B. Bone tumors confined within the cortical or periosteal range are defined as T1

C. Juxtacortical tumors not invading the cortex are defined as T2

D. For stage I tumors, thorough resection is required to avoid recurrence and metastasis

E. For stage II tumors, adjuvant chemotherapy is often needed after thorough surgical resection to reduce surgical risk

F. M stands for metastasis, M0: no metastasis, M1: metastasis present

194. Young male patient, with a lump on his left ankle for over a year, no tenderness, not affecting walking or work. Left ankle X-ray: thinning of the cortical bone at the lower end of the left tibia, no periosteal reaction.

Q: Regarding the imaging features of osteochondroma, which statement is correct

A. The thickness of the cartilage cap is usually greater than 1 cm

B. The base of the tumor does not communicate with the cortical and medullary cavity of the parent bone

C. If the thickness of the cartilage cap gradually increases, malignancy should be suspected

D. Multiple osteochondromas do not cause widening of the epiphyseal-metaphyseal junction and deformation of the bone shaft

E. CT scan is the preferred examination method

F. It appears as a round or oval bone mass protruding from the epiphyseal-metaphyseal junction

195. Young male patient, with a lump on his left ankle for over a year, no tenderness, not affecting walking or work. Left ankle X-ray: thinning of the cortical bone at the lower end of the left tibia, no periosteal reaction.

Q: The characteristic X-ray appearance of enchondroma is

A. Codman triangle

B. Onion skin periosteal reaction

C. Sunburst periosteal reaction

D. Oval radiolucent area in the medullary cavity with septa or stippled calcifications

E. Increased density of tumor bone

F. Soap bubble appearance

196. Young male patient, with a lump on his left ankle for over a year, no tenderness, not affecting walking or work. Left ankle X-ray: thinning of the cortical bone at the lower end of the left tibia, no periosteal reaction.

Q: Regarding the statement about chondroma, which description is incorrect

A. Also known as enchondroma, it is the second most common benign bone tumor

B. Commonly occurs in the short tubular bones of the hands and feet

C. Clinical manifestations are mostly pain and swelling, sometimes presenting with pathological fractures

D. Its X-ray appearance is lytic destruction, cortical thinning without expansion

E. Treatment is mainly curettage, with a low recurrence rate

F. Generally asymptomatic, with occasional dull pain

197. Male, 48 years old, gradually developed posterior neck discomfort, numbness in both hands, clumsiness, difficulty using chopsticks, and a feeling of stepping on cotton while walking, prone to falls, for the past year. Physical examination: decreased pain sensation on the lateral forearms, increased muscle tone in all four limbs, positive Hoffmann's sign, ankle clonus, and Babinski's sign. Auxiliary examination: X-ray shows loss of normal cervical lordosis at C5-6, mild local kyphosis, narrowed intervertebral space, and sclerotic changes; sagittal diameter of the spinal canal is 15 mm.

Q: The initial clinical diagnosis is

A. Cervical spondylotic radiculopathy

B. Amyotrophic lateral sclerosis

C. Cervical spondylotic myelopathy

D. Syringomyelia

E. Thoracic outlet syndrome

F. Vertebral artery type cervical spondylosis

198. Male, 48 years old, gradually developed posterior neck discomfort, numbness in both hands, clumsiness, difficulty using chopsticks, and a feeling of stepping on cotton while walking, prone to falls, for the past year. Physical examination: decreased pain sensation on the lateral forearms, increased muscle tone in all four limbs, positive Hoffmann's sign, ankle clonus, and Babinski's sign. Auxiliary examination: X-ray shows loss of normal cervical lordosis at C5-6, mild local kyphosis, narrowed intervertebral space, and sclerotic changes; sagittal diameter of the spinal canal is 15 mm.

Q: The first choice of further examination is

A. Cervical CT

B. Cervical MRI

C. Myelography

D. Evoked potentials

E. Upper limb electromyography

F. Lower limb electromyography

199. Male, 48 years old, gradually developed posterior neck discomfort, numbness in both hands, clumsiness, difficulty using chopsticks, and a feeling of stepping on cotton while walking, prone to falls, for the past year. Physical examination: decreased pain sensation on the lateral forearms, increased muscle tone in all four limbs, positive Hoffmann's sign, ankle clonus, and Babinski's sign. Auxiliary examination: X-ray shows loss of normal cervical lordosis at C5-6, mild local kyphosis, narrowed intervertebral space, and sclerotic changes; sagittal diameter of the spinal canal is 15 mm.

Q: The patient also experiences paroxysmal dizziness, blurred vision, tinnitus, and two episodes of sudden collapse lasting about half a minute, related to head movement. Given this, what additional examination should be conducted

A. Cranial CT

B. Electroencephalography

C. Vertebral artery angiography

D. Radionuclide scanning

E. Evoked potentials

F. Brain MRI

200. Male, 48 years old, gradually developed posterior neck discomfort, numbness in both hands, clumsiness, difficulty using chopsticks, and a feeling of stepping on cotton while walking, prone to falls, for the past year. Physical examination: decreased pain sensation on the lateral forearms, increased muscle tone in all four limbs, positive Hoffmann's sign, ankle clonus, and Babinski's sign. Auxiliary examination: X-ray shows loss of normal cervical lordosis at C5-6, mild local kyphosis, narrowed intervertebral space, and sclerotic changes; sagittal diameter of the spinal canal is 15 mm.

Q: The first treatment to consider is

A. Cervical traction

B. Massage

C. Surgery

D. Cervical brace fixation

E. NSAIDs and neurotrophic drugs

F. Small needle knife therapy

201. Male, 48 years old, gradually developed posterior neck discomfort, numbness in both hands, clumsiness, difficulty using chopsticks, and a feeling of stepping on cotton while walking, prone to falls, for the past year. Physical examination: decreased pain sensation on the lateral forearms, increased muscle tone in all four limbs, positive Hoffmann's sign, ankle clonus, and Babinski's sign. Auxiliary examination: X-ray shows loss of normal cervical lordosis at C5-6, mild local kyphosis, narrowed intervertebral space, and sclerotic changes; sagittal diameter of the spinal canal is 15 mm.

Q: If surgical treatment is needed, the preferred method is

A. Anterior cervical discectomy and fusion (ACDF)

B. Posterior cervical laminoplasty

C. Posterior midline myelotomy and syrinx drainage

D. Posterior cervical foraminotomy

E. Thoracic outlet decompression

F. Posterior cervical laminoplasty

202. Male, 48 years old, gradually developed posterior neck discomfort, numbness in both hands, clumsiness, difficulty using chopsticks, and a feeling of stepping on cotton while walking, prone to falls, for the past year. Physical examination: decreased pain sensation on the lateral forearms, increased muscle tone in all four limbs, positive Hoffmann's sign, ankle clonus, and Babinski's sign. Auxiliary examination: X-ray shows loss of normal cervical lordosis at C5-6, mild local kyphosis, narrowed intervertebral space, and sclerotic changes; sagittal diameter of the spinal canal is 15 mm.

Q: The typical clinical manifestation of cervical spinal stenosis is

A. Weakness in all four limbs

B. Dizziness and headache

C. Bilateral upper limb pain

D. Nausea and vomiting

E. Sensation of a tight band around the body

F. Palpitations

203. Male, 48 years old, gradually developed posterior neck discomfort, numbness in both hands, clumsiness, difficulty using chopsticks, and a feeling of stepping on cotton while walking, prone to falls, for the past year. Physical examination: decreased pain sensation on the lateral forearms, increased muscle tone in all four limbs, positive Hoffmann's sign, ankle clonus, and Babinski's sign. Auxiliary examination: X-ray shows loss of normal cervical lordosis at C5-6, mild local kyphosis, narrowed intervertebral space, and sclerotic changes; sagittal diameter of the spinal canal is 15 mm.

Q: Cervical spondylosis does not include the following types

A. Myelopathic type

B. Radiculopathic type

C. Sympathetic type

D. Vertebral artery type

E. Disc herniation type

F. Mixed type

204. Male, 48 years old, gradually developed posterior neck discomfort, numbness in both hands, clumsiness, difficulty using chopsticks, and a feeling of stepping on cotton while walking, prone to falls, for the past year. Physical examination: decreased pain sensation on the lateral forearms, increased muscle tone in all four limbs, positive Hoffmann's sign, ankle clonus, and Babinski's sign. Auxiliary examination: X-ray shows loss of normal cervical lordosis at C5-6, mild local kyphosis, narrowed intervertebral space, and sclerotic changes; sagittal diameter of the spinal canal is 15 mm.

Q: Regarding cervical spondylosis, which statement is incorrect

A. It can cause sympathetic symptoms such as tachycardia

B. The radiculopathic type presents with hand numbness and weakness

C. The sympathetic type is the most common form of cervical spondylosis

D. Severe cases can lead to paralysis

E. Osteophytes compressing the esophagus can cause dysphagia

F. Severe cases require surgical treatment

205. Male, 48 years old, gradually developed posterior neck discomfort, numbness in both hands, clumsiness, difficulty using chopsticks, and a feeling of stepping on cotton while walking, prone to falls, for the past year. Physical examination: decreased pain sensation on the lateral forearms, increased muscle tone in all four limbs, positive Hoffmann's sign, ankle clonus, and Babinski's sign. Auxiliary examination: X-ray shows loss of normal cervical lordosis at C5-6, mild local kyphosis, narrowed intervertebral space, and sclerotic changes; sagittal diameter of the spinal canal is 15 mm.

Q: The surgical indications for cervical spondylosis are

A. Neck pain with hand numbness

B. Headache, dizziness, vertigo

C. Severe neck and shoulder pain, decreased hand grip strength, X-ray showing osteophytes, narrowed intervertebral space

D. Repeated severe symptoms, long-term ineffective conservative treatment, spinal cord compression or paralysis

E. Neck and shoulder pain, decreased hand muscle strength, headache, dizziness, tinnitus

F. Palpitations, chest tightness

206. Female, 45 years old, suffered a left femoral neck fracture due to trauma. X-ray shows a complete fracture of the femoral neck with rotation and partial displacement of the femoral head, and the fracture is located in the middle part of the femoral neck.

Q: According to the Garden classification, this femoral neck fracture is

A. Type I

B. Type II

C. Type III

D. Type IV

E. Type V

207. Female, 45 years old, suffered a left femoral neck fracture due to trauma. X-ray shows a complete fracture of the femoral neck with rotation and partial displacement of the femoral head, and the fracture is located in the middle part of the femoral neck.

Q: The local hospital performed reduction and internal fixation for the fracture. Postoperative X-ray shows an angle of 148° between the inner edge of the femoral shaft and the medial pressure trabeculae of the femoral head on the anteroposterior view, and an angle of 190° between the axis of the femoral head and the axis of the femoral neck on the lateral view. According to the Garden alignment index, the reduction grade is

A. Grade I

B. Grade II

C. Grade III

D. Grade IV

E. Not applicable

208. Female, 45 years old, suffered a left femoral neck fracture due to trauma. X-ray shows a complete fracture of the femoral neck with rotation and partial displacement of the femoral head, and the fracture is located in the middle part of the femoral neck.

Q: Ten months after surgery, the patient feels pain in the left hip that worsens with walking and improves with rest. X-ray shows the internal fixation in place, a clear fracture line, and the left femoral head flattened with uneven density. The patient may have the following condition

A. Nonunion of the fracture

B. Left hip osteoarthritis

C. Malunion of the fracture

D. Avascular necrosis of the femoral head

E. Hip dislocation

F. Hip infection

209. Female, 45 years old, suffered a left femoral neck fracture due to trauma. X-ray shows a complete fracture of the femoral neck with rotation and partial displacement of the femoral head, and the fracture is located in the middle part of the femoral neck.

Q: At this point, the treatment options include

A. Replace the internal fixation

B. Remove the internal fixation and perform functional exercises

C. Take bone metabolism-regulating drugs

D. Continue observation and rest

E. Bone grafting

F. Total hip arthroplasty

210. Female, 63 years old, with left knee pain for 8 years, worsening over the past year, aggravated by climbing stairs, relieved by rest, sometimes experiencing "giving way" and "locking" of the knee joint, with poor response to conservative treatment. Physical examination: left knee varus deformity, tenderness in the medial joint space of the left knee, and normal range of motion. X-ray of the left knee shows osteophyte formation, narrowed medial joint space, and subchondral sclerosis.

Q: The initial diagnosis to consider includes

A. Rheumatoid arthritis

B. Osteoarthritis

C. Left knee meniscus injury

D. Left knee medial collateral ligament injury

E. Left knee loose body

211. Female, 63 years old, with left knee pain for 8 years, worsening over the past year, aggravated by climbing stairs, relieved by rest, sometimes experiencing "giving way" and "locking" of the knee joint, with poor response to conservative treatment. Physical examination: left knee varus deformity, tenderness in the medial joint space of the left knee, and normal range of motion. X-ray of the left knee shows osteophyte formation, narrowed medial joint space, and subchondral sclerosis.

Q: The possible pathological changes in the left knee of the patient are

A. Destruction of joint cartilage

B. Significant synovial proliferation

C. Osteoclastic bone destruction

D. Laxity of the lateral ligaments

E. Presence of lymphocytes in the subchondral bone marrow

F. Synovial mononuclear cell infiltration

212. Female, 63 years old, with left knee pain for 8 years, worsening over the past year, aggravated by climbing stairs, relieved by rest, sometimes experiencing "giving way" and "locking" of the knee joint, with poor response to conservative treatment. Physical examination: left knee varus deformity, tenderness in the medial joint space of the left knee, and normal range of motion. X-ray of the left knee shows osteophyte formation, narrowed medial joint space, and subchondral sclerosis.

Q: The most reasonable and effective treatment for this patient is

A. Physical therapy, strengthening the muscles around the knee joint

B. Intra-articular injection of hyaluronic acid to lubricate the joint cartilage

C. Proximal tibial wedge osteotomy to correct the varus deformity

D. Total knee arthroplasty

E. Arthroscopic synovectomy

F. Arthroscopic removal of loose bodies

1. The Achilles tendon reflex is used to check:

A. L2 nerve root

B. C6 nerve root

C. L4 nerve root

D. L5 nerve root

E. S1 nerve root

2. Cervical myelopathy is not related to which of the following diseases:

A. Tightness and numbness in the lower limbs, difficulty walking

B. Numbness in the upper limbs, weakness in hand muscles, instability in holding objects

C. Urinary and fecal dysfunction

D. Positive head compression and traction test

E. Irregular trunk and lower limb sensory disturbance, hyperreflexia, increased muscle tone

3. The abduction angle of the acetabulum in total hip arthroplasty should be:

A. 40°-50°

B. Greater than 60°

C. Less than 40°

D. 40°-60°

E. 30°-40°

4. Sewage, waste, and feces contaminated by pathogens of which class of infectious diseases must be thoroughly disinfected under the guidance and supervision of health and epidemic prevention institutions? If disinfection is refused, local authorities can take mandatory measures:

A. Class A

B. Class B

C. Class C

D. Class A and B

E. Class B and C

5. The test for hip flexion deformity, also known as the Thomas test, shows:

A. Flexion of the affected hip while lying flat

B. Straightening of the lumbar spine when flexing the hip

C. Flexion of the affected hip when the healthy side hip is flexed until the lumbar spine is straight

D. Flexion of the affected hip when the affected side hip is flexed until the lumbar spine is straight

E. Flexion of the hip while lying prone

6. The main manifestations of rheumatoid arthritis are:

A. Migratory swelling and pain of large joints

B. Swelling and pain of all joints with fever and rash

C. Symmetric swelling and pain of small joints with morning stiffness

D. Sudden unilateral knee pain with local heat and limited movement

E. Lumbosacral pain with morning stiffness

7. Regarding the characteristics of acromioclavicular joint dislocation, which of the following is incorrect:

A. Commonly seen in sports injuries of young people

B. Often caused by direct violence

C. Severe cases involve rupture of both the acromioclavicular and coracoclavicular ligaments

D. Negative X-ray findings can rule out acromioclavicular joint dislocation

E. Surgical treatment is needed if both the acromioclavicular and coracoclavicular ligaments are ruptured

8. The best examination for diagnosing knee ligament injury is:

A. CT

B. MRI

C. DSA

D. X-ray

E. Ultrasound

9. The most typical manifestation of anterior cruciate ligament injury of the knee is:

A. Pain

B. Joint locking

C. Swelling

D. Joint deformity

E. Knee instability

10. The functional position of the wrist joint is dorsiflexion at:

A. 10°-20°

B. 20°-25°

C. 0°-5°

D. 5°-10°

E. 30°-40°

11. The most common, simple, and safe method for reducing posterior dislocation of the hip joint is:

A. Bigelow method

B. Stimson method

C. Allis method

D. Bone traction reduction

E. Open reduction

12. The most common age for bone and joint tuberculosis is:

A. Newborns

B. Infants and young children

C. Children and adolescents

D. Middle-aged

E. Elderly

13. The best treatment for pelvic fracture with complete urethral rupture is:

A. Catheterization with a rubber catheter

B. Catheterization with a metal catheter

C. Bladder puncture

D. Bladder fistula

E. Urethral anastomosis

14. The most appropriate early treatment for acute suppurative hip arthritis is:

A. Adequate and effective antibiotics plus supportive therapy

B. Adequate and effective antibiotics plus intra-articular antibiotic injection

C. Adequate and effective antibiotics plus joint incision and drainage

D. Adequate and effective antibiotics plus plaster fixation

E. Adequate and effective antibiotics plus early functional exercises and physiotherapy

15. When medical institutions perform surgery, special examinations, or special treatments, if the patient's opinion cannot be obtained and there are no family members or related persons present, the appropriate action is:

A. The attending physician proposes a medical treatment plan and implements it after approval from the medical institution's responsible person or authorized personnel

B. The attending physician proposes a medical treatment plan and implements it after obtaining public recognition

C. The attending physician proposes a medical treatment plan and implements it after confirming its effectiveness with a third party

D. The attending physician proposes a medical treatment plan and implements it after approval from the county-level or higher health administrative department

E. The attending physician proposes a medical treatment plan and implements it after obtaining approval from peers

16. Which of the following is a drawback of cohort studies:

A. Low evidence strength

B. Difficulty establishing causality

C. Prone to loss to follow-up bias

D. Prone to recall bias

E. Prone to misclassification bias

17. Advantages of small splint fixation for fractures include all except:

A. Effectively preventing fracture end displacement

B. Facilitating timely exercises to prevent joint stiffness

C. Not hindering longitudinal muscle contraction, promoting fracture healing

D. Easy to obtain materials, simple operation, fewer complications

E. Frequent adjustments needed

18. Regarding the principles of fracture treatment, which of the following is correct:

A. Reduction, fixation, and internal and external medication

B. Reduction, fixation, and functional exercises

C. Reduction, fixation

D. Reduction, fixation, and physical therapy

E. Fixation, functional exercises, and internal and external treatment

19. The greatest advantage of open reduction over closed reduction is:

A. Achieving anatomical reduction

B. Reducing the risk of infection

C. Shortening the immobilization time

D. Shortening fracture healing time

E. Reducing trauma at the fracture site

20. Which of the following is not a right enjoyed by physicians in their professional activities:

A. Conducting medical examinations, disease investigations, medical treatments, issuing relevant medical certificates, and selecting reasonable medical, preventive, and health care plans within the scope of registered practice

B. Obeying the dispatch of health administrative departments in the event of natural disasters, infectious disease outbreaks, or major emergencies

C. Obtaining medical equipment and basic conditions commensurate with their professional activities as approved by the State Council's health administrative department

D. Engaging in medical research, academic exchanges, and participating in academic organizations

E. Participating in professional training and receiving continuing medical education

21. The most valuable method for early diagnosis of suppurative arthritis is:

A. Clinical physical examination

B. CT examination

C. Joint puncture fluid examination

D. X-ray examination

E. Blood test

22. The type of tuberculosis most likely to cause paraplegia is:

A. Cervical spine

B. Thoracic spine

C. Lumbar spine

D. Sacrococcygeal

E. Spinal appendages

23. The typical displacement of Colles' fracture of the distal radius is:

A. Distal end towards the ulnar and dorsal side

B. Proximal end towards the radial and dorsal side

C. Distal end towards the radial and dorsal side

D. Proximal end towards the radial side

E. Distal end towards the radial and palmar side

24. The preferred treatment for osteochondroma is:

A. Early detection, early surgery

B. Tumor segment resection

C. If the tumor is large and grows rapidly, the resection range should include the normal bone tissue around the tumor base

D. Curettage of the lesion and autologous bone grafting

E. Resection followed by artificial joint replacement

25. For open wounds of the face presented after 12 hours, local treatment should be:

A. Primary suture after debridement

B. No suture after debridement

C. Delayed suture after debridement

D. Treat as infected wounds with dressing changes, no debridement

E. None of the above

26. The main reason for the formation of large sequestra in acute hematogenous osteomyelitis is:

A. Rupture of periosteal blood vessels

B. Embolism of the nutrient artery of the bone

C. Direct destruction of bone tissue by abscess

D. Pathological fracture

E. Poor body resistance

27. The correct method for transporting a patient with a spinal fracture is:

A. Carry on the back

B. Transport by bicycle

C. Transport by car

D. Carry on a stretcher

E. Carry on a hard board

28. Which of the following is a late complication of fractures:

A. Traumatic arthritis

B. Shock

C. Compartment syndrome

D. Spinal cord injury

E. Fat embolism

29. Which of the following is not a predisposing cause of secondary osteoarthritis of the knee:

A. Meniscus tear

B. Rickets sequelae - bowlegs

C. Degenerative changes in knee cartilage

D. Long-term improper use of steroids

E. Knee instability

30. Which of the following is not a factor causing displacement of fracture ends:

A. The size and direction of the force

B. Muscle traction

C. The age of the injured person

D. Improper handling and treatment

E. The weight of the distal limb segment

31. Which of the following actions is inappropriate during debridement of severe open fractures:

A. Do not use a tourniquet

B. Wash the wound surface with a large amount of saline

C. Thoroughly remove non-viable tissue

D. Thoroughly remove bone fragments

E. Close the wound with rotational flaps

32. In the emergency response to public health incidents, if relevant units and individuals do not cooperate with the investigation, sampling, technical analysis, and inspection by relevant professionals, the responsible persons should be given:

A. A warning

B. Revocation of relevant licenses

C. Disciplinary action of demotion or dismissal

D. Administrative or disciplinary action

E. Criminal responsibility

33. A patient has limited thumb abduction after a glass cut on the palmar side of the wrist, likely due to:

A. Flexor digitorum profundus injury

B. Flexor digitorum superficialis injury

C. Ulnar nerve injury

D. Median nerve injury

E. Radial nerve injury

34. The displacement most likely to cause non-union of fractures is:

A. Angulation displacement

B. Lateral displacement

C. Separation displacement

D. Rotational displacement

E. Impacted displacement

35. The knee joint triad refers to injuries of:

A. Medial collateral ligament, medial meniscus, and posterior cruciate ligament

B. Lateral collateral ligament, medial meniscus, and posterior cruciate ligament

C. Medial collateral ligament, medial meniscus, and anterior cruciate ligament

D. Medial collateral ligament, lateral meniscus, and anterior cruciate ligament

E. Lateral collateral ligament, medial meniscus, and anterior cruciate ligament

36. The typical presentation of cervical spinal stenosis is:

A. Weakness in all four limbs

B. Dizziness and headache

C. Nausea and vomiting

D. Pain in both upper limbs

E. Band-like sensation

37. Regarding popliteal cysts, which statement is incorrect:

A. Local puncture, aspiration, and prednisone injection can be performed

B. Immediate surgical treatment is required upon diagnosis

C. Also known as Baker's cyst

D. Often occurs in the bursae between the semimembranosus tendon and the medial head of the gastrocnemius

E. Often communicates with the joint cavity

38. Regarding joint range of motion examinations, which statement is incorrect:

A. The elbow joint's extended position is 0° in the neutral position

B. The knee joint's extended position is 0° in the neutral position

C. The foot's lateral edge perpendicular to the lower leg is 90° in ankle extension

D. The wrist joint's extended position is 0° in the neutral position

E. All finger joints fully extended are 0° in the neutral position

39. Incorrect principle of on-site treatment for hand injuries:

A. Reduce wound contamination

B. Hemostasis

C. Wound dressing

D. Medication application

E. Local immobilization

40. The pathological changes of congenital hip dislocation mainly occur in:

A. Pelvis

B. Spine

C. Nerves and blood vessels of the hip

D. Acetabulum, femoral head, femoral neck, and joint capsule

E. Adductor muscles, iliopsoas, gluteal muscles, and tensor fasciae latae

41. Which of the following conditions results in faster fracture healing:

A. Fracture of the middle and lower third of the tibia

B. Fracture in children

C. Excessive traction

D. Soft tissue entrapment

E. Repeated manual reduction

42. Regarding spastic cerebral palsy, which deformity is the least common:

A. Foot inversion

B. Achilles tendon contracture

C. Knee flexion contracture

D. Hip flexion contracture

E. Hip abduction contracture

43. Incorrect statement about patient rights:

A. Patients have the right to equal medical treatment

B. Patients have the right to informed consent about their condition

C. Patients have the right to privacy protection

D. Patients have the right to criticize, supervise, and adjust the work of medical staff

E. Medical autonomy rights

44. Cold abscesses formed after vertebral destruction by spinal tuberculosis will not appear in:

A. Paravertebral abscess

B. Psoas abscess

C. Iliac fossa abscess

D. Deep inguinal abscess

E. Femoral triangle abscess

45. The hand deformity commonly known as "ape hand" occurs after injury to which nerve:

A. Radial nerve

B. Median nerve

C. Ulnar nerve

D. Radial nerve

E. Common peroneal nerve

46. The primary reason why abscesses in children with suppurative osteomyelitis are less likely to enter the joint cavity is:

A. Pus is easily localized and absorbed

B. Joint capsule protects the joint cavity

C. Children's joints have strong resistance to suppurative inflammation

D. Abscesses easily rupture through soft tissue

E. The epiphyseal plate acts as a barrier

47. The incorrect cause of traumatic fractures is:

A. Direct violence

B. Indirect violence

C. Accumulative wear and tear

D. Muscle contraction pulling force

E. Osteomyelitis (chronic) trauma

48. "Disappearance of the joint space, fibrous and bony ankylosis, subluxation" belongs to which X-ray stage of rheumatoid arthritis:

A. Stage I

B. Stage II

C. Stage III

D. Stage IV

E. Stage V

49. Which of the following fracture types is considered stable:

A. Oblique fracture of the radial shaft

B. Transverse fracture of the femoral shaft

C. Extension-type supracondylar fracture of the humerus

D. Greenstick fracture of both the radius and ulna

E. Adduction-type femoral neck fracture

50. Incorrect pathological change after limb dismemberment is:

A. Tissue does not die immediately even though blood circulation is interrupted

B. Irreversible pathological changes occur after 6-7 hours of ischemia at room temperature, gradually leading to death

C. Toxic substances return through veins, causing hemoglobinuria

D. If replantation surgery is performed more than 8 hours after dismemberment, systemic toxicity can occur

E. The closer the dismemberment level is to the trunk, the smaller the systemic reaction after replantation

51. Incorrect statement about the range of motion of the glenohumeral joint:

A. Abduction 90°

B. Adduction 45°

C. Flexion 90°

D. Extension 45°

E. Internal rotation 135°

52. Which of the following is not an X-ray sign of fractures:

A. Increased density band due to impaction

B. Depression and bulge of bone cortex

C. Interruption and twisting of bone trabeculae

D. Epiphyseal separation

E. Sclerotic edge forming a low-density shadow

53. When the knee joint is in a semi-flexed position and an external force is applied to the anterior aspect of the upper tibia, the injury that may occur is:

A. Medial meniscus injury

B. Lateral meniscus injury

C. Anterior cruciate ligament injury

D. Posterior cruciate ligament injury

E. Medial collateral ligament injury

54. Which of the following is not a structural scoliosis:

A. Idiopathic scoliosis

B. Congenital scoliosis

C. Neuromuscular scoliosis

D. Scoliosis associated with neurofibromatosis

E. Scoliosis caused by nerve root irritation

55. Regarding the clinical characteristics of osteosarcoma, which statement is appropriate:

A. Osteosarcoma commonly occurs in the distal femur, proximal tibia, and proximal humerus, where the growth of the epiphyseal plate is fastest

B. Osteosarcoma also frequently occurs in the spine and pelvis

C. Once diagnosed by pathological examination, amputation should be performed immediately

D. Most osteosarcomas are multifocal

E. 80%-90% of all cases occur around the knee joint

56. Incorrect description about bone tumors:

A. Bone tumors refer to those occurring within the bone or originating from various bone tissues, whether primary or metastatic

B. Benign tumors commonly include giant cell tumor of bone and osteochondroma, while malignant tumors include osteosarcoma and chondrosarcoma

C. Bone or joint pain, bone mass, and functional impairment are the three main clinical symptoms

D. X-ray examination is crucial for diagnosing bone tumors

E. Amputation remains the only treatment option

57. Complications of total knee arthroplasty do not include:

A. Deep vein thrombosis

B. Infection

C. Peroneal nerve palsy

D. Aseptic loosening of the prosthesis

E. Osteoarthritis

58. The indications for artificial hip replacement do not include

A. Old femoral neck fractures or avascular necrosis of the femoral head, damaged acetabulum, accompanied by pain and severe functional impairment

B. Hip joint ankylosis after inflammation or tuberculosis, hip joint pain or chronic hip dislocation, failed joint formation

C. Degenerative osteoarthritis with damaged acetabulum, deformed femoral head or rheumatoid arthritis, intolerable pain, limited range of motion

D. Thorough debridement of suppurative hip arthritis

E. The age range previously thought to be most suitable, 60-75 years, has been expanded to include both elderly and young patients

59. Regarding the treatment measures for acute mastitis, which is not advisable

A. Clean the local breast area daily, empty the breast milk

B. Systemic use of antibiotics

C. Apply 50% magnesium sulfate solution during the cellulitis stage

D. Stop breastfeeding

E. Incision and drainage after abscess formation

60. The possible complications of clavicle fractures are

A. Injury to C2, C3 nerve roots

B. Injury to the sympathetic nerves

C. Injury to the accessory nerve

D. Injury to the phrenic nerve

E. Injury to the brachial plexus

61. The relationship between nerve injury and deformity, which is incorrect

A. Radial nerve injury -- wrist drop and finger drop

B. Median nerve injury -- ape hand

C. Ulnar nerve injury -- claw hand

D. Radial nerve injury -- ape hand

E. Common peroneal nerve injury -- foot drop

62. Regarding the description of Froment's sign, which is incorrect

A. The proximal interphalangeal joint of the index finger flexes when pinching with the thumb

B. The distal interphalangeal joint of the index finger hyperextends when pinching with the thumb

C. The metacarpophalangeal joint of the thumb flexes when pinching with the index finger

D. Indicates motor dysfunction of the ulnar nerve

E. Caused by paralysis of the interosseous muscles and the adductor pollicis

63. The joints most commonly affected by osteoarthritis are

A. Knee joint, hip joint, distal interphalangeal joints

B. Knee joint, hip joint, proximal interphalangeal joints

C. Knee joint, wrist joint, proximal interphalangeal joints

D. Knee joint, wrist joint, elbow joint

E. Wrist joint, elbow joint, metacarpophalangeal joints

64. Among the treatment methods for chronic musculoskeletal injuries, the least commonly used clinically is

A. Restricting harmful movements, correcting poor posture

B. Local injection of corticosteroids

C. Physiotherapy, massage

D. Taking anti-inflammatory and pain-relieving medications

E. Surgical treatment

65. The most common clinical symptom of lumbar disc herniation is

A. Pain when extending the waist

B. Limited movement of the waist

C. Pain when bending the waist

D. Lower back and leg pain

E. Increased lower back pain after prolonged sitting

66. The shortcomings of relying solely on anti-tuberculosis drugs in the treatment of bone and joint tuberculosis do not include

A. Long-term use can lead to drug resistance in tuberculosis bacteria

B. Drug treatment alone is only effective for early synovial or bone tuberculosis, not for late-stage joint tuberculosis with large sequestra or abscesses

C. Drug treatment alone has a long course but a low recurrence rate

D. Early bone or synovial tuberculosis treated with drugs alone may develop into full joint tuberculosis

E. To improve the cure rate, local treatment and lesion removal surgery are also needed

67. Male, 35 years old, severe pain in the right hip after a car accident. Examination: right lower limb shortened, internally rotated, adducted, and elastically fixed, right foot unable to dorsiflex, the most likely complication is

A. Femoral nerve injury

B. Acute bone atrophy

C. Avascular necrosis of the femoral head

D. Deep vein thrombosis of the lower limb

E. Lymphedema of the lower limb

68. A patient with a right knee flexion contracture of 30 degrees, can extend the knee, and can flex the knee up to 100 degrees. The standard description is

A. 100°-0°

B. 100°-30°-0°

C. 30°-0°

D. 100°-0°-30°

E. 100°-30°

69. A 4-year-old child cried and had right elbow pain and reluctance to move when lifted by the hand up the stairs by the mother. Examination: slight swelling of the right elbow, no obvious deformity, X-ray showed no significant abnormalities. The correct treatment method is

A. Simple manual reduction

B. Manual reduction under general anesthesia

C. Open reduction and internal fixation

D. Manual reduction followed by external fixation of the affected elbow for 2 weeks

E. Surgery to release compressed nerves

70. A 2-year-old girl with a small lump on the right side of the manubrium since birth, central protrusion with occasional discharge of colorless mucus from the protruding part, and twice local redness and swelling. The diagnosis is

A. Rib deformity

B. Ectopic tracheal cartilage

C. Third branchial fistula

D. Dermoid cyst

E. Sebaceous gland tumor

71. A 43-year-old male with an open comminuted fracture of the upper third of the tibia and fibula after a leg injury from a car accident, underwent thorough debridement and removal of free bone fragments. After prolonged traction and fixation of the injured limb, the fracture still did not heal after 6 months. The most likely reason is

A. Poor blood circulation at the fracture site

B. Inadequate fixation of the injured limb

C. Excessive removal of bone fragments during thorough debridement

D. Insufficient functional exercise

E. Failure to perform timely open reduction and internal fixation surgery

72. A 60-year-old male with a neck mass for 2 years and hoarseness for 4 months, underwent total thyroidectomy 2 months ago. Pathology reported thyroid cancer. He accidentally fell on his buttocks and experienced back pain. Recently, back pain worsened, unable to get out of bed. X-ray showed compression and flattening of the L3 vertebral body, uneven density, disappearance of the right pedicle shadow, and normal intervertebral space. The diagnosis is likely

A. Lumbar disc herniation

B. Lumbar tuberculosis

C. Lumbar osteomyelitis

D. Vertebral echinococcosis

E. Metastatic bone tumor

73. A 43-year-old male gave a red envelope to the chief surgeon before surgery, but the doctor repeatedly refused. The patient insisted, saying he would lose confidence in the surgery if the doctor did not accept it. The doctor's action should be

A. Accept the red envelope reluctantly for personal use

B. Temporarily accept the red envelope and turn it in, returning it to the patient after the surgery

C. Accept the red envelope as a fund for department activities

D. Refuse the surgery if the patient insists on giving the red envelope

E. Accept the red envelope secretly if the patient insists

74. An 18-year-old female with elbow injury and swelling, limited movement, was admitted to the emergency department. Examination: significant swelling of the elbow, subcutaneous bruising, loss of sensation on the ulnar side of the hand, inability to adduct the thumb, and weakness in pinching the other four fingers together. X-ray showed supracondylar fracture of the humerus. The clinical diagnosis is humeral supracondylar fracture with

A. Median nerve injury

B. Ulnar nerve injury

C. Radial nerve injury

D. Recurrent branch of the median nerve injury

E. Superficial branch of the radial nerve injury

75. A 5-year-old child has been crying at night for 2 months, complaining of right knee pain, refusing to move the right leg, and being unable to walk or stand. Hospital examination and X-ray showed no significant abnormalities in the right knee. The problem is most likely in

A. Right ankle joint

B. Right knee joint

C. Right tibia

D. Right hip joint

E. Right femur

76. A 25-year-old male with a cut on the left palm for 2 hours. Examination: index finger can flex and extend actively, but the distal interphalangeal joint does not flex when the middle phalanx of the index finger is fixed. The most likely diagnosis is

A. Extensor tendon rupture of the index finger

B. Distal interphalangeal joint injury of the index finger

C. Flexor digitorum superficialis tendon rupture of the index finger

D. Flexor digitorum profundus tendon rupture of the index finger

E. Both flexor digitorum superficialis and profundus tendons rupture of the index finger

77. A young female patient with a left tibial midshaft fracture caused by a wheel injury, significant swelling of the affected limb. X-ray showed a midshaft fracture of the left tibia. The most meaningful sign to observe early for compartment syndrome is

A. Whether the dorsalis pedis artery pulse disappears

B. Temperature and color of the skin on the calf and foot

C. Degree of swelling of the affected limb

D. Presence of sensory impairment

E. Resting pain in the affected limb, inability to passively extend the toes, and increased pain on toe extension

78. A 45-year-old female patient with a foreign body sensation in the left throat for a week. She felt it after dining at a restaurant. Endoscopy at multiple hospitals did not find the foreign body. Specialized examination revealed some debris in the left tonsil crypt and significant tenderness in the upper left neck. A fine wire foreign body was removed from the left tonsil crypt. The patient was grateful but complained about previous hospitals. The best explanation for the patient is

A. The previous hospitals lacked technical capability

B. The doctor has a unique method

C. The patient did not cooperate well

D. The foreign body was hidden in the tonsil crypt with no part exposed, making it difficult to find with routine examination; it is a rare special case, and this timing made it easier to find

E. You are lucky to come to our hospital

79. A 32-year-old male with right lower leg pain, bleeding, and abnormal mobility after trauma, diagnosed with an open fracture of the lower third of the leg. The wound is 1 cm long, caused by the fractured end piercing the skin. X-ray showed a short oblique fracture. The correct and simplest treatment is

A. Debridement followed by plaster fixation as for a closed fracture

B. Debridement followed by interlocking intramedullary nailing

C. Debridement followed by small splint fixation

D. Debridement followed by external fixation

E. Debridement followed by plate fixation

80. Mr. Jin, a 78-year-old male, was treated for pneumonia at a nearby clinic without improvement until he became comatose and was taken to a large hospital. Diagnosed with lobar pneumonia and secondary toxic encephalopathy, he was treated in the emergency room due to a lack of beds in the internal medicine department. After using expensive antibiotics and serum albumin, his temperature normalized and he moved from deep to light coma. However, the week's treatment cost was 8000 yuan. His two retired daughters could not afford further treatment and requested to discontinue treatment. The most ethical course of action for the doctor is

A. To save health resources, avoid further rescue for such low-quality life patients

B. Persuade the family to try relatively cheaper, effective drugs for a few more days before deciding

C. Respect the family's decision and stop all treatment

D. Provide free treatment to save the patient

E. Continue treatment regardless of the family's wishes

81. Mr. Yang, a 24-year-old migrant worker, needed urgent hospitalization with only 3000 yuan on hand, while the deposit required was 8000 yuan. Without family or friends in the city and his employer struggling, he could not gather the amount. The emergency doctor consulted the hospital leadership. The ethical response is

A. Prioritize the hospital's economic benefit and admit the patient only after the full deposit is gathered

B. Prioritize social benefit and admit similar patients for free

C. Provide emergency treatment in the emergency room and refer the patient to another hospital

D. Admit the patient with the 3000 yuan deposit, requiring automatic discharge when the deposit runs out, and have the patient sign an agreement

E. Admit the patient with the 3000 yuan deposit, providing optimal treatment, and let the patient find a way to pay later if the deposit is insufficient

82. For patients over 50 with significant pain and movement disorders due to hip osteoarthritis, the first choice should be

A. Hip fusion surgery

B. Osteotomy

C. Artificial joint replacement

D. Joint debridement

E. Drilling decompression

83. An elderly patient with an insidious onset, slow progression, long-term lower back pain, intermittent claudication, difficulty walking but ease in cycling, is most likely diagnosed with

A. Lumbar spinal stenosis

B. Osteoporosis

C. Cerebral infarction

D. Lumbar disc herniation

E. Piriformis syndrome

84. A male patient, injured in a car accident, admitted with right upper arm angular deformity, exposed humeral ends, and wrist drop. The clinical diagnosis is

A. Open fracture of the right humeral shaft

B. Open fracture of the right humeral shaft with ulnar nerve injury

C. Open fracture of the right humeral shaft with radial nerve injury

D. Open fracture of the right humeral shaft with musculocutaneous nerve injury

E. Open fracture of the right humeral shaft with median nerve injury

85. A miner's right upper limb was crushed under a rock for 2 hours in a mine collapse, resulting in wrist drop, finger drop, and sensory loss in the thenar area. Electrophysiological examination was normal. The type of radial nerve injury is

A. Nerve rupture

B. Nerve disuse

C. Nerve irritation

D. Nerve contusion

E. Axonotmesis

86. A 28-year-old male with 10 years of back pain, initially radiating from the lumbosacral region to both buttocks. Diagnosed with lumbar disc herniation at the county hospital. Later, back pain increased, kyphotic deformity appeared, and bilateral hip movement was partially restricted, with shortness of breath and difficulty climbing stairs. Examination showed significant spinal movement restriction and reduced lung capacity. Thomas test positive, bedside test positive, RF(-), HLA-B27(+), ASO 200U, ESR 54 mm/h. The initial diagnosis should first consider

A. Rheumatoid arthritis

B. DISH disease

C. Ankylosing spondylitis

D. Spinal tuberculosis

E. Hypertrophic spondylitis

87. A 30-year-old male with a midshaft humeral fracture of the right upper arm, presenting with wrist drop and inability to extend the fingers. The cause is

A. Extensor pollicis longus injury

B. Ulnar nerve injury

C. Extensor digitorum communis injury

D. Radial nerve injury

E. Radial wrist extensor longus injury

88. A 16-year-old male who underwent shoulder joint disarticulation and chemotherapy for right humeral osteosarcoma 10 months ago, now presenting with right chest pain and cough. Chest X-ray indicated metastatic tumor in the right lung. The main pathway of metastasis is

A. Arterial spread

B. Lymphatic spread

C. Venous spread

D. Nerve trunk spread

E. Direct spread

89. A 13-year-old male with sudden high fever for 4 days, accompanied by pain and swelling in the upper left thigh. Examination: temperature 39.5℃, pulse 120/min, fair general condition, significant swelling in the upper left thigh, severe lateral pressure pain without fluctuation, significantly elevated white blood cell count, 83% neutrophils, normal X-ray. The initial consideration should be

A. Acute suppurative osteomyelitis

B. Ewing's sarcoma

C. Rheumatic fever

D. Giant cell tumor of bone

E. Tuberculosis of bone with secondary infection

90. A 19-year-old male with right upper arm wrist drop and finger drop after a 2-hour crush injury, with sensory loss in the thenar area and normal electrophysiological examination. The type of radial nerve injury is

A. Nerve rupture

B. Nerve disuse

C. Nerve irritation

D. Nerve contusion

E. Axonotmesis

91. A 49-year-old female with more than 5 years of neck and shoulder pain, four limbs numbness, and weakness, and unsteady gait for half a year. Examination: decreased sensation below the ulnar side of both hands, increased muscle tone in both lower limbs, muscle strength 3-4. X-ray showed significant degenerative changes in the cervical spine. The most likely diagnosis is

A. Cervical spondylosis

B. Cervical spondylotic radiculopathy

C. Cervical spondylotic myelopathy

D. Cervical spondylotic sympathetic syndrome

E. Intradural tumor

92. A 25-year-old male worker experienced sudden severe lower back pain while lifting a heavy object, followed by pain radiating to the right lower limb, with coughing pain extending to the left heel. Examination: muscle spasm in the lower back, limited movement, and lateral bending, significant tenderness and percussion pain at L4-L5. The most likely clinical diagnosis is

A. Lumbar fracture

B. Lumbar dislocation

C. Subarachnoid hemorrhage

D. Lumbar disc herniation

E. Lumbar sprain

93. A 52-year-old male with chronic bronchitis and emphysema for 6 years, experienced sudden chest pain and shortness of breath after coughing, taken to the emergency department. Examination: blood pressure 100/70 mmHg, respiration 28/min, restless, cyanosis of lips and fingers, trachea deviated to the left, right chest fullness, tympanic percussion, significantly reduced breath sounds. Diagnosed with right pneumothorax. Without proper treatment, he was sent for fluoroscopy, resulting in Cheyne-Stokes respiration and death en route to the emergency room. The correct choice for medical personnel should have been

A. Confirm the diagnosis and collect objective evidence

B. Consult relevant specialists to decide on treatment

C. Quickly judge and determine appropriate goals, even diagnosing and treating simultaneously for timely intervention

D. Make a decisive diagnosis and perform a diagnostic puncture

E. Observe first and then treat

94. The diagnosis to consider first for a 47-year-old female with exertional palpitations, shortness of breath, facial pigmentation, and a diastolic rumbling murmur at the apex that changes with body position is

A. Mitral stenosis

B. Mitral regurgitation

C. Left atrial myxoma

D. Aortic regurgitation

E. Total anomalous pulmonary venous return

95. A 29-year-old female with a left forearm fracture, manually reduced and splinted for 5 hours, experiencing severe pain, finger numbness, swelling, and immobility. The most likely cause is

A. Nerve injury

B. Nerve and vein compression

C. Artery and vein compression

D. Vein compression

E. Artery injury

96. A 56-year-old male with neck and shoulder pain for 1 month radiating to the right hand, decreased sensation in the right thumb, and weakened biceps muscle strength. The preliminary diagnosis is

A. Cervical spondylosis

B. Frozen shoulder

C. Rotator cuff syndrome

D. Brachial plexus neuritis

E. Neck strain

97. A 25-year-old female with a femoral shaft fracture from a car accident, requiring emergency surgery, with the primary indication being

A. Significant angulation of the fracture

B. Significant displacement of the fracture

C. Overlapping and angulated fracture

D. Comminuted fracture

E. Open fracture

98. Doctors conducting clinical research without patient consent, but informing the attending physician to obtain extra blood or bone marrow samples, is ethically analyzed as

A. Not informing to avoid unnecessary psychological burden on the patient, which is justifiable

B. Not informing because most patients would not consent, ensuring research continuity, which is justifiable

C. The good intentions of clinical research justify the lack of informed consent

D. Any human experiment without informed consent is ethically indefensible, regardless of intention

E. None of the above

99. A patient with positional vertigo due to cervical spondylosis most likely has

A. Cervical radiculopathy

B. Cervical myelopathy

C. Cervical sympathetic syndrome

D. Vertebral artery syndrome

E. Esophageal syndrome

100. A 22-year-old male with a replantation of a severed right thumb, presenting with pallor and decreased temperature of the replanted thumb 36 hours post-surgery. The incorrect clinical management is

A. Brachial plexus block

B. Use of antispasmodic drugs

C. Hyperbaric oxygen therapy

D. Immediate surgical exploration

E. Loosening dressings to relieve compression

101. The patient, a 78-year-old male, was admitted to the hospital due to an intertrochanteric fracture of the right femur and multiple rib fractures caused by a car accident. After admission, only chest band fixation and skin traction of the right lower limb were given. The complication that should be most vigilant and prevented in this patient is

A. Avascular necrosis of the femoral head

B. Fat embolism

C. Hypostatic pneumonia

D. Major vascular and nerve injury

E. Traumatic myositis ossificans

102. A 24-year-old male fell from an 8-meter height, landing on his arm. He was admitted to the hospital 12 hours after the injury, with significantly reduced or absent sensation below three fingers from the navel, only slight movement of the right big toe, and loss of voluntary movement in both lower limbs, along with urinary retention. X-ray shows T10 vertebral compression with bone fragments protruding into the spinal canal. The key early treatment is

A. Active and vigorous antibiotic treatment

B. Catheterization with indwelling catheter

C. Good nursing care, frequent turning to prevent bedsores

D. Early surgery to relieve compression

E. Reduction by high-low table method followed by plaster vest

103. The patient, a 18-year-old female cyclist, has had pain below the patella without obvious inducement for 4 months, worsening after activity and relieving with rest. The condition has gradually worsened, with knee weakness and difficulty climbing stairs. The most likely diagnosis is

A. Osgood-Schlatter disease

B. Chondromalacia patellae

C. Cruciate ligament injury

D. Meniscus injury

E. Collateral ligament injury

104. The patient, Mr. Chen, a 49-year-old male with advanced gastric cancer, is in extreme pain but believes it is gastritis and hopes for treatment. Whenever he feels discomfort or has demands, he rings the bell hoping the medical staff can solve it. The staff often avoids him, causing great disappointment. Once, when the pain recurred and he requested pain relief, he was refused. The best approach in medical ethics for dealing with such a patient is

A. All patient requests should be met

B. Meet some of the patient's requests to alleviate pain

C. Explain the truth and inform the patient that treatment is impossible

D. Continue to hide the condition and provide passive treatment

E. Inform the patient of the truth with family consent when appropriate, meet the patient's needs as much as possible, and alleviate pain

Correct answer: E

105. A 60-year-old male porter has had intermittent claudication for 1 year, worsening for 1 month, with no history of smoking, alcohol, or trauma. Physical examination is most likely to find

A. Positive straight leg raising test

B. Significantly weakened dorsalis pedis pulse

C. Cyanosis of lips

D. Obvious tenderness in the lumbar and hip area

E. No obvious positive signs in the lumbar and leg area

Correct answer: E

106. The patient, a 41-year-old female, was hit on the right knee with a blunt object. X-ray shows a fracture of the fibular head, and the ankle joint cannot be actively dorsiflexed, considering

A. Common peroneal nerve injury

B. Sciatic nerve injury

C. Tibial nerve injury

D. Anterior tibial muscle rupture

E. Gastrocnemius tear

107. A 68-year-old female patient with left knee osteoarthritis and 30° flexion contracture and 10° varus deformity underwent total knee arthroplasty. Postoperatively, the patient's foot dorsum was numb, and ankle dorsiflexion was weak. The possible complication is

A. Prosthesis dislocation

B. Dressing too tight

C. Anterior tibial artery injury

D. Common peroneal nerve paralysis

E. Tibial nerve injury

108. The patient, a 20-year-old male, sustained a fracture of the left scaphoid bone, treated with manual reduction and plaster cast immobilization for 6 months. X-ray recheck shows nonunion with sclerosis at the fracture ends, possibly due to

A. Poor blood supply to the fracture site

B. Soft tissue interposition at the fracture ends

C. Severe soft tissue injury

D. Improper treatment method

E. Advanced patient age

109. A 36-year-old male had the distal segment of the right middle finger amputated by a machine, exposing the phalanx. The least suitable treatment method is

A. Shorten the phalanx and suture the skin

B. Free grafting of medium-thickness skin

C. Pedicled flap grafting

D. Bilateral advancement flap formation

E. Severe wound contamination, debridement, temporary dressing, and observation

110. A 20-year-old badminton player has had pain on the lateral side of the right elbow for half a year, worsening after exercise. Examination reveals tenderness on the lateral side of the right elbow, but normal elbow joint function. The most likely diagnosis is

A. Radial nerve injury

B. Old elbow dislocation

C. Old supracondylar fracture of the humerus

D. Chronic suppurative osteomyelitis of the humerus

E. Lateral epicondylitis of the humerus

Correct answer: E

111. A 5-year-old male child has had lumbar tuberculosis for 1 month. The test most likely to be positive is

A. McMurray test

B. Thomas sign

C. Gaenslen sign

D. Picking-up test

E. Dugas sign

112. A 40-year-old female patient sustained a tibial plateau or femoral condyle fracture with a 0.8 cm uneven articular surface. Despite treatment, the fracture displacement remained unchanged. The most likely late complication is

A. Myositis ossificans

B. Malunion

C. Traumatic arthritis of the knee joint

D. Knee joint stiffness

E. Knee joint valgus deformity

113. A male patient sustained a right humeral shaft fracture due to trauma, with the right upper arm 4 cm shorter than the left and a mild angular deformity. Bone conduction sound was weakened on auscultation. The most likely fracture displacement is

A. Shortening displacement

B. Mixed displacement

C. Lateral displacement

D. Angular displacement

E. Separation displacement

114. A 28-year-old female had a 2-year-old scar contracture deformity of the right palm after a burn. After scar excision, blood vessels, tendons, nerves, and joint capsules were exposed. The first choice for wound repair is

A. Flap grafting

B. Split-thickness skin grafting

C. Forearm flap repair

D. Abdominal flap repair

E. Dorsal foot flap repair

115. A 20-year-old male had right shoulder pain after a fall from a bicycle 3 hours ago. Examination reveals swelling and tenderness at the right acromioclavicular joint, with no elastic fixation palpable. X-ray shows no abnormalities. The correct treatment is

A. Sling suspension

B. Figure-eight bandage fixation

C. No treatment

D. Bed rest for 2 weeks

E. Small shoulder splint fixation

116. A 70-year-old male has had left lower limb pain for 2 years, worsening for 1 month, with a 10-year history of rheumatoid arthritis. Examination reveals normal lumbar lordosis and flexion activities, positive spinal extension test, decreased sensation on the left lateral calf, and grade 4 strength in the left long extensor muscle. X-ray shows mild narrowing of the L4-5 intervertebral space, and positive straight leg raising test at 35°. The most likely diagnosis is

A. Lumbar muscle strain

B. Lumbar spondylosis

C. Lumbar spondylolysis

D. L4-5 disc herniation

E. Buerger's disease

117. A 68-year-old female patient with left knee osteoarthritis and 30° flexion contracture and 10° varus deformity underwent total knee arthroplasty. Postoperatively, the patient's foot dorsum was numb, and ankle dorsiflexion was weak. The possible complication is

A. Prosthesis dislocation

B. Dressing too tight

C. Anterior tibial artery injury

D. Common peroneal nerve paralysis

E. Tibial nerve injury

118. A 28-year-old male had low back pain with radiating pain to the left lower limb after lifting a heavy object 1 week ago. Symptoms worsened with coughing and sneezing, making it difficult to get out of bed. No previous similar episodes. Examination reveals loss of lumbar lordosis, significant activity limitation, positive straight leg raising test at 30°, decreased sensation on the left lateral foot, and weakened Achilles reflex on the left. X-ray shows loss of lumbar lordosis. Urinalysis is normal. The initial diagnosis is

A. Acute lumbar facet joint dysfunction

B. Acute lumbar sprain

C. L4-5 disc herniation

D. L5-S1 disc herniation

E. Lumbar spondylolysis

119. A 25-year-old male sustained a closed fracture of the left femoral shaft with no significant displacement. X-ray shows a transverse fracture of the left femoral shaft. The first choice of treatment is

A. Manual reduction with small splint fixation and additional weight traction

B. Manual reduction with plaster fixation

C. External fixation frame

D. Open reduction with internal fixation

E. Bed rest with strict immobilization

120. An 18-year-old female has had low-grade fever, night sweats, and back pain for 2 months. A mass appeared in the right iliac fossa, which was fluid-filled on ultrasound. The right hip joint had restricted flexion. The diagnosis is considered to be

A. Vertebral metastasis

B. Pelvic tumor

C. Lumbar tuberculosis

D. Knee joint tuberculosis

E. Hip joint tuberculosis

121. A 25-year-old male sustained an open comminuted fracture of the upper third of the right tibia and fibula from a wheel crush injury. After thorough debridement to remove all free bone fragments, traction was applied but the fracture remained unhealed 3 months later. The most likely reason is

A. Insufficient blood supply to the fracture site

B. Inadequate limb fixation

C. Insufficient functional exercise

D. Failure to perform timely open reduction and fixation

E. Excessive removal of bone fragments during debridement

Correct answer: E

122. A 30-year-old male was hit on the lower left thigh with an iron rod, resulting in deformity. X-ray shows a transverse fracture of the left femoral shaft with the distal fragment significantly tilted backward. The main factor causing this fracture displacement is

A. Muscle pulling force

B. Nature of the violence

C. Magnitude of the violence

D. Weight of the distal limb segment

E. Improper handling

123. A 41-year-old male has had right knee medial pain and swelling for half a year, worsening for 5 days, especially when climbing stairs. X-ray at an external hospital shows a translucent area of 4 cm × 6 cm on the medial side of the upper right tibia, with a "soap bubble" appearance in the middle and bone expansion. Recently, swelling and pain have worsened, especially at night. X-ray at admission shows an expanded lesion in the upper tibia with a "cloudy" appearance and invasion into soft tissue. The incorrect treatment option is

A. Autologous bone and joint transplantation

B. Curettage, bone grafting, and bone cement filling

C. Allogeneic bone and artificial joint composite transplantation

D. Amputation

E. Allogeneic hemiarthroplasty and total joint replacement

124. A 25-year-old male had a right knee sprain 2 weeks ago, with knee instability, giving way, and a sense of "misalignment" while walking. The medial head of the quadriceps muscle is visibly atrophied, with tenderness in the medial joint space, positive McMurray test, negative drawer test, and negative valgus stress test. The most likely diagnosis is

A. Medial collateral ligament rupture

B. Medial meniscus injury

C. Anterior cruciate ligament rupture

D. Intra-articular loose body

E. Osteomalacia

125. A 38-year-old male has had dull pain in the left lower back after activity for the past year, relieved with rest. Recently, the same symptoms recurred, and a urine test showed 5 red blood cells per HP. To confirm if there is an upper urinary tract stone and provide a basis for treatment, the examination of choice is

A. Ultrasound

B. Plain film of the urinary system (KUB)

C. Excretory urography (IVP)

D. KUB + IVP

E. CT

126. An 8-year-old boy had pain in his left upper arm 2 hours after trauma. Based on the AP and oblique views of the left humerus, the most likely diagnosis is

Click to see 1

A. Osteosarcoma

B. Ewing's sarcoma

C. Bone cyst

D. Eosinophilic granuloma

E. Non-ossifying fibroma

127. An 18-year-old male had right knee and lower leg injuries from a car accident, leading to immediate dysfunction of foot plantar flexion, inversion, and adduction, with loss of foot sole sensation. The most likely injured nerve is

A. Sural nerve

B. Femoral nerve

C. Common peroneal nerve

D. Tibial nerve

E. Sciatic nerve

128. A 56-year-old male has had right knee pain for half a year, worsening for 5 days, especially when climbing stairs. X-ray shows degenerative changes in the knee joint, diagnosed as chondromalacia patellae. The first non-surgical treatment should be immobilization of the knee joint for

A. 1-2 weeks

B. 3-4 weeks

C. 4-5 weeks

D. 6-7 weeks

E. 8-9 weeks

129. A male porter had sudden severe low back pain during unloading, unable to move his waist. After 3 days of bed rest, bilateral buttock pain started. Neurological examination is normal. The most likely clinical diagnosis is

A. Lumbar disc herniation

B. Lumbar myofascial inflammation

C. Lumbar muscle strain

D. Acute lumbar sprain

E. Lumbar fracture

130. A 45-year-old female patient had a head injury from a car accident, with immediate coma. She was admitted 1 hour later in a moderate coma, with right pupil dilation, loss of light reflex, and positive pathological signs in the left upper and lower limbs. The first measure to take is

A. Administer hemostatic drugs

B. 20% mannitol 250 ml IV infusion

C. Administer antibiotics to prevent infection

D. Dexamethasone 20 mg IV infusion

E. Administer respiratory stimulants

131. A 20-year-old male had his right thigh amputated due to a car accident. To reduce residual limb swelling, minimize bedrest complications, and start early weight-bearing training, the most important treatment is

A. Elastic bandage wrapping

B. ADL training

C. Enhanced residual limb skin care

D. Residual limb muscle strength training

E. Fitting a temporary prosthesis

Correct answer: E

132. A 21-year-old male had swelling and pain in the upper left calf for half a year, worsening for the past month, affecting sleep. Examination reveals severe swelling and tenderness in the upper left tibia, with distended superficial veins. A 6 cm × 7 cm hard, fixed mass with unclear boundaries is palpable. X-ray shows "moth-eaten" osteolytic destruction in the upper left tibia, with significant periosteal reaction and visible "Codman triangle." The first-line chemotherapy regimen should be

A. Adriamycin + Vincristine, Cyclophosphamide + Adriamycin

B. Vincristine + Cyclophosphamide

C. Melphalan + Prednisone

D. Adriamycin + Cisplatin + Methotrexate + Ifosfamide

E. Cyclophosphamide + Adriamycin + Vincristine

133. An 18-year-old male has had intermittent pain and swelling in the middle of the left thigh with fever for 3 years, with no episodes in the past 8 months. X-ray shows chronic osteomyelitis of the femur. Examination reveals no redness or swelling, mild deep tenderness, and an 8 cm thickened femur with a 30 mm × 25 mm × 25 mm dead space and a 20 mm × 15 mm × 20 mm sequestrum. The best treatment is

A. Local hot compresses and oral antibiotics

B. Sequestrectomy with open wound dressing

C. Sequestrectomy with saucerization

D. Sequestrectomy with pedicled muscle flap filling and wound closure

E. Sequestrectomy with closed irrigation-suction therapy

134. A 20-year-old male injured his left knee while playing soccer, resulting in medial joint pain and swelling with limited movement. After 1 month of conservative treatment, symptoms improved but there were occasional joint locking and giving way. Examination reveals atrophy of the medial head of the quadriceps muscle, tenderness in the medial joint space, positive McMurray test, negative drawer test, and negative valgus stress test. The most likely diagnosis is

A. Medial collateral ligament rupture

B. Medial meniscus injury

C. Anterior cruciate ligament rupture

D. Intra-articular loose body

E. Osteomalacia

135. A 9-year-old girl had sudden right hip pain with high fever 5 days ago. Examination reveals a temperature of 39.5°C, pulse of 110 bpm, white blood cell count of 22 × 10⁹/L, 98% neutrophils, and erythrocyte sedimentation rate of 30 mm/h. The right hip joint is swollen and immobile. The primary consideration is

A. Acute suppurative arthritis

B. Acute rheumatic arthritis

C. Rheumatoid arthritis

D. Periarticular soft tissue inflammation

E. Tuberculous arthritis

136. A 65-year-old male has an old femoral neck fracture and avascular necrosis of the femoral head. The best treatment is

A. Hip fusion surgery

B. Trochanteric rotational osteotomy

C. Femoral head drilling and bone grafting

D. Total hip arthroplasty

E. Drug therapy

137. A 40-year-old female is scheduled for knee replacement after tumor resection of the upper tibia. The best choice of prosthesis is

A. Condylar constrained knee prosthesis

B. Hinged artificial knee prosthesis

C. Rotating hinge knee prosthesis

D. Ball-and-socket knee prosthesis

E. Posterior stabilized knee prosthesis

138. A 4-year-old boy had a transverse fracture of the right femoral shaft with overlapping displacement. The appropriate treatment method is

A. Vertical suspension skin traction

B. Manual reduction with small splint fixation

C. Manual reduction with small splint fixation plus skin traction

D. Open reduction with internal fixation

E. Manual reduction with skeletal traction

139. A male was injured when a cart overturned, crushing his lower abdomen. Examination reveals tenderness at the pubic symphysis, a positive compression test, a full bladder, and blood on the tip of the catheter after attempted insertion. This suggests

A. Insufficient depth of catheter insertion

B. Incorrect catheter insertion method

C. Catheter obstruction

D. Pelvic fracture with urethral rupture

E. Pelvic fracture with bladder injury

140. A 26-year-old male experienced sudden vomiting of blood and abdominal pain after drinking two bottles of chilled beer. The most likely diagnosis is

A. Gastric mucosal prolapse

B. Gastric ulcer

C. Duodenal ulcer

D. Acute erosive hemorrhagic gastritis

E. Mallory-Weiss syndrome

141. A 30-year-old female, 30 weeks pregnant, was admitted with upper abdominal colic, nausea, vomiting, and lower abdominal pain. Lab tests show increased white blood cells and bilirubin; blood potassium is 3.1 mmol/L, amylase is 4854 U/L. Ultrasound shows multiple gallstones and cholecystitis. Fetal ultrasound indicates normal development. The incorrect treatment is

A. Immediate surgical treatment upon diagnosis

B. Cesarean section

C. Conservative treatment

D. Termination of pregnancy followed by conservative treatment

E. Termination of pregnancy followed by surgery

142. A 30-year-old male had a blunt abdominal injury six months ago and developed an upper abdominal mass two months ago, with recent vomiting but no pain or fever. Examination reveals a 9 cm × 10 cm cystic mass in the upper abdomen without tenderness. The clinical diagnosis is a pancreatic pseudocyst. The best treatment is

A. Aspiration of the cyst contents

B. Surgical removal of the cyst

C. External drainage of the cyst

D. Internal drainage of the cyst

E. Observation without treatment

143. A 45-year-old male developed right neck and shoulder pain, with numbness and weakness in the right upper limb after a week of catching a cold. The right brachial plexus stretch test is positive, and the right middle finger has reduced pain sensation. The compressed nerve root is

A. C4

B. C5

C. C6

D. C7

E. C8

144. A 6-year-old child was diagnosed with a greenstick fracture of the clavicle. The correct treatment is

A. Suspend the affected limb with a sling for 3 weeks

B. Manual reduction with figure-eight bandage fixation

C. Surgical reduction with internal fixation

D. No treatment

E. Bed rest for 2 weeks

145. A 20-year-old male fell headfirst into a ditch while riding a bicycle at night, resulting in incomplete quadriplegia. X-ray shows odontoid process fracture with subluxation. The first treatment measure should be

A. Plaster collar fixation

B. Skull traction fixation

C. Sling traction

D. Occipital-cervical fixation surgery

E. Open reduction with internal fixation

146. A 33-year-old male suddenly developed headache, ptosis of the right eyelid, right pupil dilation, loss of direct and consensual light reflex in the right eye, with a normal consensual light reflex in the left eye. The likely site of injury is

A. Left optic nerve and oculomotor nerve

B. Left oculomotor nerve

C. Right oculomotor nerve

D. Specific projection system

E. Non-specific projection system

147. A 36-year-old female had the ulnar side of the distal phalanx of the right thumb cut by a machine, with a 1.6 cm × 1.2 cm soft tissue defect and exposed phalanx. The wound was contaminated for 12 hours. The treatment should be

A. Debridement followed by primary medium-thickness skin grafting

B. Debridement followed by primary shortening and suturing of the distal phalanx

C. Debridement followed by primary thumb island flap grafting

D. Debridement followed by temporary coverage with artificial skin or Vaseline gauze, with re-debridement and thumb island flap grafting after 72 hours

E. Debridement followed by free flap grafting

148. A 50-year-old female had periumbilical pain for 12 hours, spreading to the entire abdomen for 2 hours, with tenderness, rebound tenderness, and muscle rigidity, especially in the right lower abdomen. Abdominal X-ray shows no abnormalities. Diagnostic abdominal paracentesis yields thin, purulent fluid with a slight odor. The most likely diagnosis is

A. Perforated acute appendicitis

B. Strangulated intestinal obstruction

C. Tuberculous peritonitis

D. Severe acute pancreatitis

E. Intra-abdominal hemorrhage

149. A 30-year-old male had right elbow pain and limited movement 2 hours after a car accident. Examination reveals stable vital signs, clear consciousness, stable breathing, negative chest compression test, soft abdomen without significant tenderness, rebound tenderness, or muscle rigidity. Specialized examination reveals swelling, deformity, and tenderness of the right elbow joint, with the disappearance of the elbow triangle. The right hand and forearm have normal sensation and movement, and the radial pulse is normal. A palpable displaced olecranon with bone crepitus is noted. The most likely diagnosis is

A. Extension-type supracondylar fracture of the humerus

B. Flexion-type supracondylar fracture of the humerus

C. Terrible triad of the elbow

D. Radial head subluxation

E. Olecranon fracture

150. A 68-year-old male with benign prostatic hyperplasia underwent transurethral resection of the prostate and developed urinary incontinence 1 month postoperatively, with urine leaking spontaneously from the urethral meatus without bladder distension. The most likely type of urinary incontinence is

A. Overflow incontinence

B. True incontinence

C. Urge incontinence

D. Psychogenic incontinence

E. Stress incontinence

151. A 48-year-old female has had left shoulder pain for 3 months, gradually worsening, with inability to comb her hair, limited abduction, and extension of the left shoulder joint. Examination reveals deltoid muscle atrophy without numbness in the arm. The most likely diagnosis is

A. Cervical spondylosis

B. Shoulder tumor

C. Adhesive capsulitis

D. Shoulder joint tuberculosis

E. Rheumatoid arthritis

152. A 20-year-old male sustained a mid-shaft fracture of the right clavicle with significant displacement. During the consultation, he consistently maintained his head tilted to the right, with his left hand supporting his right elbow. The primary reason for this position is

A. Forced posture due to concurrent right neck and shoulder soft tissue sprain

B. Right brachial plexus injury with decreased muscle strength in the right upper limb

C. Concurrent right subclavian vessel injury, with this posture reducing upper limb ischemia

D. Temporary immobilization to reduce bleeding at the fracture site

E. Muscle relaxation and reduced upper limb weight traction to alleviate fracture pain

Correct answer: E

153. A 20-year-old male sustained a mid-shaft fracture of the right clavicle with significant displacement. Consider surgery for open reduction and internal fixation if

A. The patient is young and has high aesthetic demands

B. The fracture ends are significantly overlapping and shortened

C. There is still 2/3 lateral displacement of the fracture ends after reduction

D. The fracture ends cannot be contacted after manual reduction due to obstruction

E. Numbness occurs in the affected limb after manual reduction and plaster fixation

154. A 20-year-old male sustained a mid-shaft fracture of the right clavicle with significant displacement. During manual closed reduction, the right upper limb should be tractioned

A. Upward and outward

B. Upward and backward

C. Downward and backward

D. Downward and forward

E. Upward and forward

155. A 60-year-old male porter has had intermittent claudication for 1 year, worsening for 1 month, with no history of smoking, alcohol, or trauma. The correct statement is

A. For lumbar disc herniation on the medial side of the nerve root, spinal curvature towards the healthy side can relieve pain

B. For lumbar disc herniation on the lateral side of the nerve root, spinal curvature towards the healthy side can relieve pain

C. For lumbar disc herniation outside the intervertebral foramen, spinal curvature towards the affected side can relieve pain

D. For lumbar disc herniation outside the intervertebral foramen, spinal curvature towards the healthy side can relieve pain

E. For lumbar disc herniation on the medial side of the nerve root, spinal curvature towards either side cannot relieve pain

156. A 60-year-old male porter has had intermittent claudication for 1 year, worsening for 1 month, with no history of smoking, alcohol, or trauma. The most prevalent type of scoliosis is

A. Idiopathic scoliosis

B. Congenital scoliosis

C. Neuromuscular scoliosis

D. Neurofibromatosis

E. Mesenchymal tissue disorders

157. A 42-year-old female accountant has had neck discomfort and soreness for 2 months, progressing to neck and shoulder pain radiating to the upper limb over the past 3 weeks. Turning the neck to a certain angle causes shooting pain. The most likely diagnosis is

A. Cervical radiculopathy

B. Adhesive capsulitis

C. Atlantoaxial subluxation

D. Cervical disc herniation

E. Cervical spinal stenosis

158. A 42-year-old female accountant has had neck discomfort and soreness for 2 months, progressing to neck and shoulder pain radiating to the upper limb over the past 3 weeks. Turning the neck to a certain angle causes shooting pain. The most significant examination finding is

A. Neck tenderness

B. Positive upper limb traction test

C. Limited cervical spine movement

D. Abnormal upper limb sensation

E. Decreased upper limb muscle strength

159. A 42-year-old female accountant has had neck discomfort and soreness for 2 months, progressing to neck and shoulder pain radiating to the upper limb over the past 3 weeks. Turning the neck to a certain angle causes shooting pain. The most significant imaging examination is

A. Open-mouth view

B. Anteroposterior cervical spine view

C. Three-view cervical spine series

D. Cervical spine extension view

E. Cervical spine flexion view

160. A 42-year-old female accountant has had neck discomfort and soreness for 2 months, progressing to neck and shoulder pain radiating to the upper limb over the past 3 weeks. Turning the neck to a certain angle causes shooting pain. The first-choice treatment is

A. Oral non-steroidal anti-inflammatory drugs

B. Pain point injection

C. Manipulative therapy

D. Cervical traction

E. Physical therapy

161. A 35-year-old female fell backward while walking, landing on her right hand. She experienced right shoulder pain and limited movement. Examination reveals a squared shoulder deformity and a positive Dugas sign. The initial clinical diagnosis should consider

A. Right shoulder soft tissue injury

B. Anterior dislocation of the right shoulder joint

C. Surgical neck fracture of the humerus

D. Anatomical neck fracture of the humerus

E. Acromioclavicular joint dislocation

162. A 35-year-old female fell backward while walking, landing on her right hand. She experienced right shoulder pain and limited movement. Examination reveals a squared shoulder deformity and a positive Dugas sign. The first auxiliary examination to perform is

A. Anteroposterior X-ray

B. Anteroposterior and lateral X-ray

C. Anteroposterior and transthoracic X-ray

D. CT

E. Shoulder arthroscopy

163. A 50-year-old female has had left shoulder pain radiating to the left upper arm for over a month, worsening with limited shoulder joint movement, and night pain affecting sleep for 7 days. A history of left shoulder trauma was noted. Examination reveals atrophy of shoulder muscles, limited shoulder joint movement, especially abduction and rotation, with localized tenderness in the anterior shoulder and no obvious deformity. The most likely diagnosis is

A. Left shoulder soft tissue contusion

B. Left shoulder rheumatoid arthritis

C. Left shoulder muscle strain

D. Left shoulder adhesive capsulitis

E. Cervical spondylosis

164. A 50-year-old female has had left shoulder pain radiating to the left upper arm for over a month, worsening with limited shoulder joint movement, and night pain affecting sleep for 7 days. A history of left shoulder trauma was noted. Examination reveals atrophy of shoulder muscles, limited shoulder joint movement, especially abduction and rotation, with localized tenderness in the anterior shoulder and no obvious deformity. The most helpful examination for diagnosis is

A. X-ray

B. Electromyography

C. CT and MRI

D. Arthroscopy

E. CT

165. A 50-year-old female has had left shoulder pain radiating to the left upper arm for over a month, worsening with limited shoulder joint movement, and night pain affecting sleep for 7 days. A history of left shoulder trauma was noted. Examination reveals atrophy of shoulder muscles, limited shoulder joint movement, especially abduction and rotation, with localized tenderness in the anterior shoulder and no obvious deformity. The current appropriate treatment is

A. Immobilize the affected side

B. Traditional Chinese medicine

C. Oral pain medication

D. Local injection of prednisolone acetate

E. Active shoulder joint exercises with adjunctive physical therapy and massage

166. While seated in a car, the patient experienced acute right hip pain and hip joint movement restriction in a flexed, adducted, and internally rotated position after a sudden stop caused the right knee to hit the front. The diagnosis should be

A. Femoral neck fracture

B. Intertrochanteric fracture

C. Subtrochanteric fracture

D. Posterior dislocation of the hip joint

E. Anterior dislocation of the hip joint

167. While seated in a car, the patient experienced acute right hip pain and hip joint movement restriction in a flexed, adducted, and internally rotated position after a sudden stop caused the right knee to hit the front. The possible associated injury is

A. Sciatic nerve injury

B. Femoral nerve injury

C. Obturator nerve injury

D. Tibial nerve injury

E. Common peroneal nerve injury

168. While seated in a car, the patient experienced acute right hip pain and hip joint movement restriction in a flexed, adducted, and internally rotated position after a sudden stop caused the right knee to hit the front. The treatment method to choose is

A. Hippocrates method

B. Kocher method

C. Allis method

D. Skeletal traction

E. Skin traction

169. A 25-year-old obese male was in a traffic accident, resulting in a left tibia and fibula open comminuted fracture. The most important step in emergency treatment is

A. Temporarily bandage the wound, immobilize, and transport quickly

B. Transport quickly

C. Attempt reduction

D. Properly immobilize

E. Correct use of a tourniquet

170. A 25-year-old obese male was in a traffic accident, resulting in a left tibia and fibula open comminuted fracture. The most suitable fixation method for this type of fracture is

A. Plaster splint

B. Windowed plaster cast with traction pin

C. External fixation frame

D. Plate and screw internal fixation

E. Intramedullary nail internal fixation

171. A 60-year-old male fell, injuring his right hip. Examination reveals right lower limb shortening, 50° external rotation deformity, and mild swelling of the right hip with percussion pain. The first examination to perform is

A. X-ray

B. CT

C. MRI

D. Nuclear bone scan

E. Arthrography

172. A 60-year-old male fell, injuring his right hip. Examination reveals right lower limb shortening, 50° external rotation deformity, and mild swelling of the right hip with percussion pain. Examination reveals a Garden type I fracture of the right femoral neck. The best treatment plan is

A. Emergency open reduction and internal fixation with three-blade nails

B. Right lower limb skin traction

C. Right total hip replacement

D. Right femoral head replacement

E. Reduction and plaster fixation

173. A 60-year-old male fell, injuring his right hip. Examination reveals right lower limb shortening, 50° external rotation deformity, and mild swelling of the right hip with percussion pain. One year later, the patient experiences pain with hip movement. X-ray shows increased density and unclear trabecular pattern of the right femoral head. This suggests

A. Hypertrophic changes of the femoral head

B. Traumatic arthritis

C. Avascular necrosis of the femoral head

D. Degenerative arthritis

E. Degenerative changes of the femoral head

174. A 60-year-old male fell, injuring his right hip. Examination reveals right lower limb shortening, 50° external rotation deformity, and mild swelling of the right hip with percussion pain. If total hip arthroplasty is performed, the acetabular anteversion angle should be

A. 10°-15°

B. Greater than 60°

C. Less than 40°

D. 40°-60°

E. 40°-50°

175. A 20-year-old female has had right calf pain for 2 months, progressively worsening with fever for 1 week. Examination reveals a temperature of 38.0°C, swelling and tenderness in the middle of the right tibia, with increased local skin temperature. X-ray shows osteolytic destruction in the middle of the right tibia with "onion skin" periosteal reaction. The clinical diagnosis is Ewing's sarcoma, which needs to be differentiated from

A. Bone tuberculosis

B. Suppurative osteomyelitis

C. Bone cyst

D. Fibrosarcoma

E. Fibrous dysplasia

176. A 20-year-old female has had right calf pain for 2 months, progressively worsening with fever for 1 week. Examination reveals a temperature of 38.0°C, swelling and tenderness in the middle of the right tibia, with increased local skin temperature. X-ray shows osteolytic destruction in the middle of the right tibia with "onion skin" periosteal reaction. The most valuable auxiliary examination for diagnosis and differentiation is

A. X-ray

B. Nuclear bone scan

C. CT

D. White blood cell count and differential

E. Tissue biopsy and pathological examination

Correct answer: E

177. A 20-year-old female has had right calf pain for 2 months, progressively worsening with fever for 1 week. Examination reveals a temperature of 38.0°C, swelling and tenderness in the middle of the right tibia, with increased local skin temperature. X-ray shows osteolytic destruction in the middle of the right tibia with "onion skin" periosteal reaction. The appropriate treatment is

A. Chemotherapy

B. Radiotherapy

C. Surgery + chemotherapy

D. Surgery + radiotherapy

E. Surgery + chemotherapy + radiotherapy

Correct answer: E

178. A 45-year-old male had both mid-thighs crushed by a car 6 hours before being brought to the hospital. Examination shows severe swelling, bruising, and deformity of both mid-thighs, with bone crepitus and abnormal mobility. Sensation and movement below the knees are normal, and blood circulation to the feet is good. X-rays show severe comminuted fractures of both femoral shafts. The most likely complication to be vigilant about is

A. Shock

B. Vascular injury

C. Nerve injury

D. Compartment syndrome

E. Ischemic bone necrosis

179. A 45-year-old male had both mid-thighs crushed by a car 6 hours before being brought to the hospital. Examination shows severe swelling, bruising, and deformity of both mid-thighs, with bone crepitus and abnormal mobility. Sensation and movement below the knees are normal, and blood circulation to the feet is good. X-rays show severe comminuted fractures of both femoral shafts. After 1 week of skeletal traction, X-rays show poor alignment of both fractures. The best treatment is

A. Adjust traction angle and weight

B. Switch to small splint external fixation

C. Small splint external fixation + skeletal traction

D. Manual reduction + bilateral hip spica cast

E. Open reduction and internal fixation

Correct answer: E

180. A 45-year-old male had both mid-thighs crushed by a car 6 hours before being brought to the hospital. Examination shows severe swelling, bruising, and deformity of both mid-thighs, with bone crepitus and abnormal mobility. Sensation and movement below the knees are normal, and blood circulation to the feet is good. X-rays show severe comminuted fractures of both femoral shafts. After 1 year of systematic physical therapy and functional exercise, the right knee function has mostly recovered, but the left knee has not improved. X-rays show no abnormalities. The most likely cause is

A. Traumatic arthritis

B. Joint stiffness

C. Joint ankylosis

D. Internal fixation affecting joint movement

E. Severe soft tissue injury

181. A 45-year-old male had both mid-thighs crushed by a car 6 hours before being brought to the hospital. Examination shows severe swelling, bruising, and deformity of both mid-thighs, with bone crepitus and abnormal mobility. Sensation and movement below the knees are normal, and blood circulation to the feet is good. X-rays show severe comminuted fractures of both femoral shafts. The most appropriate treatment is

A. Continue physical therapy + functional exercise

B. Combine with Chinese herbal medicine treatment

C. Surgical release of periarticular adhesions

D. Left knee joint fusion

E. Total knee arthroplasty

182. A 4-year-old boy fell from a bed approximately 50 cm high, refusing to let anyone touch his left elbow. Examination shows he is unwilling to use his left hand or move his elbow, with no obvious deformity. The first choice of examination is

A. X-ray

B. Nerve electrophysiology

C. CT scan

D. Ultrasound

E. MRI

F. ECG

183. A 4-year-old boy fell from a bed approximately 50 cm high, refusing to let anyone touch his left elbow. Examination shows he is unwilling to use his left hand or move his elbow, with no obvious deformity. If there is deformity in the elbow on examination, the first choice of examination is

A. AP and lateral X-rays of the left humerus

B. AP and lateral X-rays of the left elbow joint

C. AP and lateral X-rays of the left radius and ulna

D. CT scan of the humerus

E. MRI of the elbow joint

F. CT scan of the elbow joint

184. A 4-year-old boy fell from a bed approximately 50 cm high, refusing to let anyone touch his left elbow. Examination shows he is unwilling to use his left hand or move his elbow, with no obvious deformity. If the X-ray is negative, the most likely diagnosis is

A. Supracondylar fracture of the humerus

B. Shoulder dislocation

C. Elbow dislocation

D. Radial head subluxation

E. Elbow soft tissue injury

F. Olecranon fracture

185. A 4-year-old boy fell from a bed approximately 50 cm high, refusing to let anyone touch his left elbow. Examination shows he is unwilling to use his left hand or move his elbow, with no obvious deformity. The common age of onset for this condition is

A. Under 5 years

B. 3-8 years

C. 5-10 years

D. 10-12 years

E. 12-15 years

F. 15-18 years

186. A 4-year-old boy fell from a bed approximately 50 cm high, refusing to let anyone touch his left elbow. Examination shows he is unwilling to use his left hand or move his elbow, with no obvious deformity. The most likely positive finding is

A. Abnormal relationship of the elbow triangle

B. Elbow joint swelling

C. Positive Dugas sign

D. Radial head tenderness

E. Elbow varus

F. Elbow valgus

187. A 4-year-old boy fell from a bed approximately 50 cm high, refusing to let anyone touch his left elbow. Examination shows he is unwilling to use his left hand or move his elbow, with no obvious deformity. The further treatment for this condition is

A. CT scan of the shoulder joint

B. X-ray of the shoulder joint

C. CT scan of the elbow joint

D. Manual reduction without fixation

E. Manual reduction with sling fixation

F. MRI of the elbow joint

188. A 4-year-old boy fell from a bed approximately 50 cm high, refusing to let anyone touch his left elbow. Examination shows he is unwilling to use his left hand or move his elbow, with no obvious deformity. The contributing factor for this condition is

A. Anterior inclination angle between the humeral shaft axis and the humeral condyle axis

B. Rotational external force on the forearm

C. Weakness of the annular ligament around the radial neck

D. Simultaneous abduction and external rotation force on the humeral head

E. Carrying angle between the humeral longitudinal axis and the extended line of the ulna's long axis

F. Ligament rupture

189. A 4-year-old boy fell from a bed approximately 50 cm high, refusing to let anyone touch his left elbow. Examination shows he is unwilling to use his left hand or move his elbow, with no obvious deformity. If the condition recurs after a family member pulls the left hand 3 months after treatment, the best treatment plan is

A. Plaster fixation

B. Splint fixation

C. Sling suspension

D. Manual reduction with sling suspension

E. Surgical open reduction

F. Observation

190. A 36-year-old male had left knee pain for 1 year, worsening with activity and relieved by rest, with no significant night pain. One month ago, pain worsened, and the left knee became swollen and restricted in movement. No history of trauma, other bone diseases, bone tumors, or family history. Examination reveals swelling below the left knee, increased skin temperature, a palpable elastic bone shell-like mass (like a ping pong ball) with tenderness and clear boundaries, and limited active but normal passive knee movement. X-ray results are as follows:

Click to see 1

The most likely diagnosis is

A. Giant cell tumor of bone

B. Ewing's sarcoma

C. Osteoid osteoma

D. Chondrosarcoma

E. Osteosarcoma

F. Osteochondroma

191. A 36-year-old male had left knee pain for 1 year, worsening with activity and relieved by rest, with no significant night pain. One month ago, pain worsened, and the left knee became swollen and restricted in movement. No history of trauma, other bone diseases, bone tumors, or family history. Examination reveals swelling below the left knee, increased skin temperature, a palpable elastic bone shell-like mass (like a ping pong ball) with tenderness and clear boundaries, and limited active but normal passive knee movement. X-ray results are as follows:

Click to see 1

Regarding giant cell tumor of bone, the correct statements are

A. It is a malignant tumor

B. It commonly occurs in people aged 20-40

C. It can metastasize to the lungs

D. Some patients present with pathological fractures

E. It often involves joint dysfunction

F. It commonly occurs in the epiphysis of long bones and vertebral bodies

Correct answers: CDEF

192. A 36-year-old male had left knee pain for 1 year, worsening with activity and relieved by rest, with no significant night pain. One month ago, pain worsened, and the left knee became swollen and restricted in movement. No history of trauma, other bone diseases, bone tumors, or family history. Examination reveals swelling below the left knee, increased skin temperature, a palpable elastic bone shell-like mass (like a ping pong ball) with tenderness and clear boundaries, and limited active but normal passive knee movement. X-ray results are as follows:

Click to see 1

Further auxiliary examinations should include

A. ECG

B. Blood tests

C. Blood biochemistry

D. MRI

E. CT

F. Pathological examination

Correct answers: BCDEF

193. A 36-year-old male had left knee pain for 1 year, worsening with activity and relieved by rest, with no significant night pain. One month ago, pain worsened, and the left knee became swollen and restricted in movement. No history of trauma, other bone diseases, bone tumors, or family history. Examination reveals swelling below the left knee, increased skin temperature, a palpable elastic bone shell-like mass (like a ping pong ball) with tenderness and clear boundaries, and limited active but normal passive knee movement. X-ray results are as follows: Click to see 1

Regarding the treatment of this patient, the correct statements are

A. The goal is to completely remove the tumor

B. Prevent recurrence

C. Preserve limb function

D. Radiotherapy should be the primary treatment

E. Chemotherapy should be the primary treatment

F. Surgical treatment should be the primary approach

Correct answers: BCF

194. A 20-year-old male injured his right knee while playing basketball, causing pain, swelling, and restricted movement. Examination reveals slight swelling, positive patellar tap, and significant pain with active and passive knee movement. For a definitive diagnosis, the following tests can be performed

A. Thomas sign

B. Drawer test and Lachman test

C. "4" sign test

D. Single-leg stand test

E. Straight leg raise test

F. Femoral nerve stretch test

195. A 20-year-old male injured his right knee while playing basketball, causing pain, swelling, and restricted movement. Examination reveals slight swelling, positive patellar tap, and significant pain with active and passive knee movement. A positive valgus stress test indicates

A. Medial collateral ligament injury

B. Anterior cruciate ligament injury

C. Posterior cruciate ligament injury

D. Meniscus injury

E. Lateral collateral ligament injury

F. Discoid meniscus

196. A 20-year-old male injured his right knee while playing basketball, causing pain, swelling, and restricted movement. Examination reveals slight swelling, positive patellar tap, and significant pain with active and passive knee movement. X-ray shows no obvious fracture. The examination that can help clarify the diagnosis is

A. X-ray

B. MRI

C. Ultrasound

D. CT

E. Nerve electrophysiology

F. ECG

197. A 20-year-old male injured his right knee while playing basketball, causing pain, swelling, and restricted movement. Examination reveals slight swelling, positive patellar tap, and significant pain with active and passive knee movement. A positive drawer test and Lachman test suggest the most likely diagnosis is

A. Right knee meniscus injury

B. Right knee anterior cruciate ligament injury

C. Right knee injury with combined ligament damage

D. Right knee posterior cruciate ligament injury

E. Right knee injury with multiple ligament damage

F. Discoid meniscus of the right knee

198. A 20-year-old male injured his right knee while playing basketball, causing pain, swelling, and restricted movement. Examination reveals slight swelling, positive patellar tap, and significant pain with active and passive knee movement. O'Donoghue's triad in knee injuries does not include

A. Anterior cruciate ligament

B. Medial meniscus

C. Medial collateral ligament

D. Lateral collateral ligament

E. Tibial plateau

F. Distal femur

Correct answers: EF

199. A 20-year-old male injured his right knee while playing basketball, causing pain, swelling, and restricted movement. Examination reveals slight swelling, positive patellar tap, and significant pain with active and passive knee movement. Common types of knee injuries do not include

A. Flexion, abduction, and external rotation injuries

B. Anteroposterior displacement injuries

C. Hyperextension injuries

D. Flexion, adduction, and external rotation injuries

E. Excessive abduction

F. Flexion, abduction, and internal rotation injuries

Correct answers: EF

200. A 20-year-old male injured his right knee while playing basketball, causing pain, swelling, and restricted movement. Examination reveals slight swelling, positive patellar tap, and significant pain with active and passive knee movement. The functions of the posterior cruciate ligament do not include

A. Limiting posterior displacement of the tibia

B. Limiting knee hyperflexion and external rotation

C. Limiting knee hyperextension and internal rotation

D. Limiting abduction and adduction

E. Limiting knee hyperextension

F. Limiting knee hyperflexion

Correct answer: F

201. A 20-year-old male injured his right knee while playing basketball, causing pain, swelling, and restricted movement. Examination reveals slight swelling, positive patellar tap, and significant pain with active and passive knee movement. If the patient has a medial collateral ligament injury, the most suitable treatment is

A. Plaster splint external fixation

B. Arthroscopic exploration and repair

C. Oral pain medication and bed rest

D. Local block

E. Splint external fixation

F. Acupuncture and physical therapy

202. An 11-year-old male has had right hip pain and limited movement for half a year, worsening in the past week, with afternoon low-grade fever, night sweats, and fatigue. Examination reveals right hip tenderness without swelling, hip joint stiffness, and limited flexion and extension. X-ray shows no significant bone destruction, with slightly narrowed joint space. The patient has a history of primary pulmonary tuberculosis. The primary consideration is

A. Right hip suppurative arthritis

B. Ankylosing spondylitis with hip involvement

C. Transient synovitis of the hip

D. Right hip tuberculosis

E. Right hip osteoarthritis

F. Traumatic arthritis of the right hip

203. An 11-year-old male has had right hip pain and limited movement for half a year, worsening in the past week, with afternoon low-grade fever, night sweats, and fatigue. Examination reveals right hip tenderness without swelling, hip joint stiffness, and limited flexion and extension. X-ray shows no significant bone destruction, with slightly narrowed joint space. The patient has a history of primary pulmonary tuberculosis. For further diagnosis, the most valuable examination is

A. Blood tests and erythrocyte sedimentation rate

B. CT scan

C. ECT

D. Synovial biopsy under arthroscopy

E. Joint aspiration and smear examination

F. MRI

204. An 11-year-old male has had right hip pain and limited movement for half a year, worsening in the past week, with afternoon low-grade fever, night sweats, and fatigue. Examination reveals right hip tenderness without swelling, hip joint stiffness, and limited flexion and extension. X-ray shows no significant bone destruction, with slightly narrowed joint space. The patient has a history of primary pulmonary tuberculosis. The early X-ray findings of hip tuberculosis primarily show

A. Predominant bone proliferation

B. Coexistence of bone proliferation and destruction

C. Predominant bone resorption

D. Localized decalcification

E. Disappearance of joint space

F. Hip joint fusion

205. An 11-year-old male has had right hip pain and limited movement for half a year, worsening in the past week, with afternoon low-grade fever, night sweats, and fatigue. Examination reveals right hip tenderness without swelling, hip joint stiffness, and limited flexion and extension. X-ray shows no significant bone destruction, with slightly narrowed joint space. The patient has a history of primary pulmonary tuberculosis. Hip tuberculosis often presents as

A. Single joint pain onset

B. Acute onset with rapid progression

C. Multiple joint pain

D. Predominant pain in small joints of the spine

E. More common in the elderly

F. Self-healing

206. An 11-year-old male has had right hip pain and limited movement for half a year, worsening in the past week, with afternoon low-grade fever, night sweats, and fatigue. Examination reveals right hip tenderness without swelling, hip joint stiffness, and limited flexion and extension. X-ray shows no significant bone destruction, with slightly narrowed joint space. The patient has a history of primary pulmonary tuberculosis. The physical examination method not commonly used to assess hip tuberculosis is

A. Hip hyperextension test

B. "4" sign test

C. Thomas test

D. Picking-up test

E. Hip rolling test

F. Straight leg raise test

207. An 11-year-old male has had right hip pain and limited movement for half a year, worsening in the past week, with afternoon low-grade fever, night sweats, and fatigue. Examination reveals right hip tenderness without swelling, hip joint stiffness, and limited flexion and extension. X-ray shows no significant bone destruction, with slightly narrowed joint space. The patient has a history of primary pulmonary tuberculosis. The most common early form of hip tuberculosis is

A. Simple synovial tuberculosis

B. Mixed tuberculosis

C. Simple osseous tuberculosis

D. Total joint tuberculosis

E. Systemic toxic symptoms

F. None of the above

208. An 11-year-old male has had right hip pain and limited movement for half a year, worsening in the past week, with afternoon low-grade fever, night sweats, and fatigue. Examination reveals right hip tenderness without swelling, hip joint stiffness, and limited flexion and extension. X-ray shows no significant bone destruction, with slightly narrowed joint space. The patient has a history of primary pulmonary tuberculosis. The pathological changes in late-stage hip tuberculosis do not include

A. Cold abscess

B. Pathological dislocation

C. Narrowing of the joint space

D. Normal joint space

E. Joint stiffness

F. Possible sinus formation

209. An 11-year-old male has had right hip pain and limited movement for half a year, worsening in the past week, with afternoon low-grade fever, night sweats, and fatigue. Examination reveals right hip tenderness without swelling, hip joint stiffness, and limited flexion and extension. X-ray shows no significant bone destruction, with slightly narrowed joint space. The patient has a history of primary pulmonary tuberculosis. The distinguishing feature between hip tuberculosis and transient synovitis of the hip is

A. The latter is transient without afternoon fever or night sweats

B. The former is more common in adults

C. The latter is more common in adults

D. The latter's X-ray shows bone destruction

E. Difficult to distinguish

F. The latter is persistent

210. An 11-year-old male has had right hip pain and limited movement for half a year, worsening in the past week, with afternoon low-grade fever, night sweats, and fatigue. Examination reveals right hip tenderness without swelling, hip joint stiffness, and limited flexion and extension. X-ray shows no significant bone destruction, with slightly narrowed joint space. The patient has a history of primary pulmonary tuberculosis. The common sites for simple osseous tuberculosis in the hip are

A. Ligamentum teres of the femur

B. Edge of the acetabulum

C. Edge of the femoral head

D. Edge of the femoral head or the iliac part of the acetabulum

E. Center of the femoral head

F. None of the above

1. The typical displacement of the distal end in Colles fracture is

A. Displacement of the distal end towards the ulnar and dorsal side

B. Displacement of the proximal end towards the radial and dorsal side

C. Displacement of the distal end towards the radial and dorsal side

D. Displacement of the proximal end towards the radial side

E. Displacement of the distal end towards the radial and palmar side

2. Which of the following is not a situation where "medical institutions are presumed to be at fault"?

A. Violating laws, administrative regulations, rules, and other relevant medical norms

B. Concealing or refusing to provide medical records related to the dispute

C. Forging or altering medical records

D. Medical staff not yet obtaining a practicing physician's certificate

E. Deliberately destroying medical records

3. Which of the following limb measurement methods is incorrect?

A. Length must be measured with both limbs placed symmetrically

B. Circumference must be measured at the middle of the limb

C. The length of the upper limb is from the acromion to the radial styloid process (or the tip of the middle finger)

D. The length of the lower limb is from the anterior superior iliac spine to the tip of the medial malleolus

E. The axis of the lower limb is the line between the anterior superior iliac spine and the first toe web when the knee is extended, passing through the center of the patella

4. The position with the highest pressure on the lumbar intervertebral discs is

A. Standing position

B. Sitting position

C. Semi-recumbent position

D. Supine position

E. Hyperextended position

5. The following statements about medical personnel leaking medical secrets and causing adverse consequences do not include

A. It may cause discrimination against patients by some people in society

B. It may cause patients to distrust and fear medical personnel

C. It may cause doctor-patient conflicts and family disputes

D. It may cause a heavy psychological burden on patients, even leading to serious consequences such as suicide

E. It may lead to medical errors and accidents

Correct answer: E

6. The most common long-term complication of supracondylar humerus fractures is

A. Median nerve injury

B. Malunion forming cubitus varus deformity

C. Ischemic contracture of forearm muscles

D. Myositis ossificans

E. Joint stiffness

7. Fractures at the junction of the cortical and cancellous bone at the metaphysis of long bones, with the center of the heel landing upright, are most likely to cause which type of displacement?

A. Angular displacement

B. Lateral displacement

C. Shortening displacement

D. Separation displacement

E. Rotational displacement

8. Which of the following can become the object of health legal relations?

A. Medical institution

B. Patient

C. Health administrative agency

D. Health administrative actions

E. Health supervision agency

9. The Charnley type artificial hip joint small head design is intended to

A. Save materials

B. Reduce friction between the head and socket

C. Increase joint stability

D. Extend the life of the head

E. Increase the range of motion of the artificial hip joint

10. The routine X-ray position for developmental hip dysplasia is

A. Bilateral hip anteroposterior view

B. Bilateral hip lateral view

C. Single hip abduction position

D. Bilateral hip adduction position

E. Single hip adduction position

11. The most common site for osteosarcoma is

A. Distal femur

B. Distal tibia

C. Proximal humerus

D. Upper end of the fibula

E. Spine

12. Which intervertebral disc herniation can cause changes in the Achilles tendon reflex?

A. L2~L3

B. L3~L4

C. L4~L5

D. L5~S1

E. S1~S2

13. Which of the following does not constitute a public health emergency?

A. A hospital in Shanghai receives 10 measles patients in one day

B. 30 workers in a hardware factory in Tianjin show symptoms of mild to severe lead poisoning

C. Zhejiang reports 2 H1N1 avian flu patients, one of whom has died

D. Beijing reports 1 case of atypical pneumonia, who has been cured and discharged

E. A hospital in a city discovers 7 cases of unknown etiology with similar symptoms in one day

14. After determining the auxiliary examination items, medical personnel must

A. No need to persuade or explain as long as the purpose of the examination is clear

B. Inform the patient (or family) to obtain informed consent, and respect the examinee

C. Doctors can make decisions as long as it is beneficial for treatment

D. Explain the dangers of the examination to the patient

E. No need to explain the financial burden of the examination items to the patient due to treatment needs

15. Factors affecting the therapeutic effect of scoliosis do not include

A. Age of onset

B. Time of detection

C. Degree of curvature

D. Medication status

E. Selection and wearing of orthoses

16. The surgical neck of the humerus is located

A. At the junction of the greater and lesser tuberosities of the humerus

B. At the junction of the greater and lesser tuberosities transitioning to the humeral shaft

C. Around the annular groove of the humeral head

D. At the junction of the humeral head and humeral shaft

E. At the metaphysis of the upper end of the humerus

17. The fracture that presents with a "silver fork" deformity after injury is

A. Smith fracture

B. Colles fracture

C. Ulnar nerve injury

D. Radial nerve injury

E. Median nerve injury

18. The indicator for determining the maturity of spinal development is

A. Cobb angle

B. Risser index

C. Barthel index

D. Kater index

E. FIM

19. The energy for biphasic external defibrillation in adults should generally not exceed

A. 100J

B. 200J

C. 300J

D. 400J

E. 500J

20. Regarding the clinical healing criteria for fractures, which of the following is incorrect?

A. No longitudinal percussion pain in the affected limb

B. No abnormal movement locally

C. Disappearance of the fracture line

D. No deformation after removal of external fixation

E. The injured upper limb can hold 1 kg in front horizontally for 1 minute

21. To measure the elevation of the greater trochanter, which method can be used to determine this?

A. Nelaton line

B. Codman triangle

C. Pauwels angle

D. Chamberlain line

E. Schmorl's node

22. The common type of cervical spondylosis that causes dizziness and headache is

A. Nerve root type

B. Spinal cord type

C. Sympathetic nerve type

D. Vertebral artery type

E. Mixed type

23. According to the "Regulations on the Implementation Rules of Medical Institution Management": The legal right of patients to understand the disease situation, diagnostic and treatment measures, costs, and prognosis is

A. Right to choose

B. Right to decide

C. Right to privacy

D. Right to informed consent

E. Right to personality

24. In the early stages after limb immobilization or fixation, which of the following is most valuable for preventing muscle atrophy and restoring function?

A. Isokinetic exercise

B. Isometric exercise

C. Resistance exercise

D. Isotonic exercise

E. Stretching or extension exercises

25. During the transportation of patients with spinal fractures, the position that should be fixed is

A. Semi-recumbent position

B. Lateral position

C. Prone position

D. Supine hyperextended position

E. Flexed lateral position

26. Which of the following is not included in the basic ethical principles of genetic diagnosis and treatment?

A. Principle of benefiting scientific research

B. Principle of informed consent

C. Principle of benefiting the patient

D. Principle of confidentiality

E. Principle of respecting the patient

27. The incorrect statement about nerve injuries is

A. When there is a conduction function disorder, the nerve fibers have not undergone degenerative changes

B. Conduction function disorders can mostly recover on their own without sequelae

C. Complete nerve rupture must be surgically repaired

D. Axonal interruption of the nerve cannot mostly recover on its own

E. Axonal interruption of the nerve requires neurolysis

28. The functional position of the elbow joint is

A. 0° position

B. 30° position

C. 60° position

D. 90° position

E. 120° position

29. Which of the following is not an indication for open reduction of fractures?

A. Intra-articular fractures with joint surface displacement exceeding 2mm

B. Manual reduction fails to achieve anatomical alignment

C. Soft tissue interposition between fracture ends, manual reduction fails

D. Multiple fractures, to reduce complications

E. Fractures with vascular and nerve injury requiring surgical exploration

30. The main treatment method for scoliosis with a Cobb angle of 20° to 45° is

A. Medication

B. Exercise therapy

C. Corrective gymnastics

D. Wearing a scoliosis brace

E. Surgery

31. In the clinical manifestations of congenital hip dislocation before the age of 1, which of the following will not appear?

A. Asymmetrical skin folds

B. Positive Allis sign

C. Shortening of the affected limb in unilateral dislocation

D. Positive Ortolani sign

E. Positive Trendelenburg test

Correct answer: E

32. Which of the following fractures requires anatomical reduction?

A. Femoral shaft fracture

B. Humeral shaft fracture

C. Metacarpal fracture

D. Fibular fracture

E. Ankle fracture

Correct answer: E

33. The treatment principle for congenital muscular torticollis in infants is

A. Non-surgical treatment, including massage, traction, and posture correction

B. Surgical treatment, including sternocleidomastoid muscle release

C. Hormone therapy

D. No treatment needed, just observation

E. Local injection of sclerosing agent

34. In cases of pelvic fractures with an inability to urinate in the emergency department, the first consideration should be to exclude

A. Bladder rupture

B. Anterior urethral injury

C. Posterior urethral injury

D. Hemorrhagic shock

E. Renal failure with anuria

35. Which of the following is incorrect about Garden classification of femoral neck fractures?

A. Type I, incomplete fracture

B. Type II, complete fracture without displacement

C. Type III, complete fracture with partial displacement

D. Type IV, complete fracture with full displacement

E. Type V, fracture dislocation

Correct answer: E

36. Once compartment syndrome is diagnosed, the first step in treatment is

A. Use of dehydration and swelling reduction drugs

B. Immediate removal of external fixation

C. Continue observation for 1 hour, if no improvement, then treat

D. Immediate open reduction to relieve vascular compression

E. Immediate surgical decompression of the deep fascia

Correct answer: E

37. Which of the following is incorrect in the postoperative management of hand injuries?

A. Elevate the affected limb to prevent swelling

B. Administer tetanus antitoxin

C. Use gauze to separate fingers during dressing and expose fingertips

D. Use a plaster splint to fix the hand in an extended position after surgery

E. Use antibiotics to prevent infection

38. The fundamental approach and method for medical ethics cultivation is

A. Learning medical ethics theory

B. Cultivating in medical and health care practice

C. Targeted approach

D. Persistence

E. Pursuing self-discipline

39. Which of the following is a closed fracture?

A. Pubic fracture with bladder or urethral rupture

B. Coccyx fracture causing rectal rupture

C. Lumbar compression fracture with retroperitoneal hematoma

D. Knife wound causing ulna fracture

E. Gunshot wound causing femoral shaft fracture

40. Which of the following is incorrect about the characteristics of central spinal cord injury syndrome?

A. The injury is mostly caused by hyperflexion of the cervical spine

B. Cervical spine injury causing obstruction of the radicular artery and anterior spinal artery can worsen the injury

C. The severity of paralysis varies between the upper and lower limbs, with the upper limbs being more affected, or one upper limb being paralyzed, or both lower limbs being paralyzed

D. Hand function impairment is often significant, with atrophy of the intrinsic muscles of the hand

E. There may be sensory and deep sensation disturbances below the injury level

41. Which of the following statements about lumbar spondylolisthesis is correct?

A. All lumbar spondylolisthesis should be actively treated surgically

B. Degenerative lumbar spondylolisthesis often exceeds Grade II

C. The key to surgical treatment of lumbar spondylolisthesis is reduction and fixation of the slippage

D. Isthmic spondylolisthesis often has no clear history of trauma

E. Lumbar spine X-rays in both anteroposterior and lateral views can clearly show the condition of the isthmus

42. Which of the following does not match the clinical characteristics of bone metastases?

A. The incidence of bone metastases from breast cancer is higher than that from lung cancer

B. It can present with cancer pain, pathological fractures, spinal cord compression, hypercalcemia, and bone marrow failure

C. It releases soluble mediators, activating osteoclasts and osteoblasts, promoting bone destruction

D. There is currently no effective cure

E. X-ray shows mostly osteolytic bone destruction

Correct answer: E

43. A positive oblique pull test indicates

A. Sacroiliac joint disease

B. Hip joint disease

C. Sacroiliac joint disease

D. Sciatic nerve disease

E. Lumbar disease

44. The common complication of extension-type supracondylar humerus fractures is

A. Ischemic necrosis of the lower end of the humerus

B. Vascular nerve injury

C. Local infection of the fracture

D. Fat embolism

E. Traumatic shock

45. Spinal cord concussion due to spinal trauma is caused by

A. Spinal cord nerve cells encountering shock, causing temporary functional inhibition and conduction disorder

B. Bone fragments piercing the spinal cord

C. Spinal cord compression by hematoma and other factors

D. Destruction of spinal cord nerve cells after trauma

E. Disruption of ascending and descending neural tracts in the spinal cord

46. Rheumatoid arthritis most commonly affects

A. Hip joint

B. Elbow joint

C. Knee joint

D. Shoulder joint

E. Small joints of the limbs

Correct answer: E

47. The pathological change in stenosing tenosynovitis is

A. Tenosynovitis

B. Bursitis

C. Tendonitis

D. Tenosynovitis and bursitis

E. Tendonitis and tenosynovitis

Correct answer: E

48. Frozen shoulder is a self-limiting disease, and the general recovery time is about

A. One month

B. Three months

C. One year

D. Two years

E. Three years

49. After peripheral nerve injury, which of the following examinations is least significant for evaluating nerve repair?

A. Electrical stimulation test

B. Nerve trunk percussion test

C. Electromyography

D. CT scan

E. Nerve conduction velocity test

50. The following description of the main functions of the meniscus does not include

A. Absorbing shocks, providing cushioning

B. Helping to evenly distribute joint fluid

C. Providing nerve feedback and proprioceptive function

D. Secreting synovial fluid to nourish articular cartilage

E. Participating in maintaining joint stability

51. Which of the following knee joint diseases and corresponding tests is incorrect?

A. Positive patellar tap test: knee joint effusion

B. Positive anterior drawer test: posterior cruciate ligament rupture

C. Positive posterior drawer test: posterior cruciate ligament rupture

D. Positive McMurray test: meniscal injury

E. Positive patellar grind test: chondromalacia patellae

52. Medical ethics principles do not include

A. Principle of justice

B. Principle of beneficence

C. Principle of non-maleficence

D. Principle of life value

E. Principle of respect

53. The displacement deformity of the distal radius in Smith fracture is

A. Displacement towards the ulnar and dorsal side

B. Displacement towards the radial and dorsal side

C. Displacement towards the ulnar and palmar side

D. Displacement towards the radial and palmar side

E. Displacement only towards the palmar side

54. The earliest site of acute hematogenous osteomyelitis is

A. Metaphysis of short tubular bones

B. Flat bones

C. Epiphysis of joints

D. Metaphysis of long tubular bones

E. Diaphysis of long tubular bones

55. Which of the following is incorrect about the ethical significance of hospice care?

A. Hospice care is an important development of medical humanism

B. It reflects the unity of sanctity of life, life quality theory, and life value theory

C. Hospice care has become an important means of controlling the dying process like euthanasia

D. It respects and practices the principles of non-maleficence and beneficence in medicine

56. Which of the following situations does not require delayed suture removal?

A. Severe anemia, emaciation, cachexia

B. Severe electrolyte imbalance not corrected

C. Elderly patients and infants

D. Uncontrolled coughing with chest or abdominal incisions should delay suture removal

E. Suture reaction at the wound site

Correct answer: E

57. After the reduction of radial and ulnar shaft fractures, the forearm should be fixed in

A. Pronation

B. Supination

C. Neutral position

D. Radial deviation

E. Ulnar deviation

58. The ethical principle reflected in "Saving lives en masse" in the "Huangdi Neijing · Suwen" is:

A. Principle of autonomy

B. Principle of non-maleficence

C. Principle of beneficence

D. Principle of justice

E. Principle of humanitarianism

Correct answer: E

59. The joint most prone to dislocation is

A. Shoulder joint

B. Knee joint

C. Elbow joint

D. Ankle joint

E. Hip joint

60. Which of the following is not a complication of total knee arthroplasty?

A. Deep vein thrombosis

B. Infection

C. Common peroneal nerve palsy

D. Aseptic loosening of the prosthesis

E. Osteoarthritis

Correct answer: E

61. A 9-year-old boy with swelling and pain in the right hip, limping, accompanied by low-grade fever, night sweats, and loss of appetite for 3 weeks. Physical examination: Temperature 37.7°C, limited movement in the right hip, positive Thomas sign. X-ray: Slight narrowing of the right hip joint space, marginal bone destruction. The primary diagnosis should be considered as

A. Avascular necrosis of the femoral head

B. Acute suppurative arthritis

C. Acute osteomyelitis

D. Osteoarthritis

E. Hip joint tuberculosis

Correct answer: E

62. A 7-year-old boy clinically diagnosed with right-sided torticollis. Physical examination: A mass palpable in the right sternocleidomastoid muscle. X-ray: Normal cervical spine. The appropriate treatment method is

A. Mass excision

B. Plaster correction

C. Total sternocleidomastoid muscle excision

D. Physical therapy

E. Sternocleidomastoid muscle release above the clavicle + plaster correction

Correct answer: E

63. A 36-year-old male with a right-sided Colles fracture fixed with a plaster cast for 13 weeks. Follow-up X-ray shows discontinuity of the bone cortex, clear fracture gap, and increased bone density at the fracture ends. This suggests

A. Nonunion of the fracture

B. Delayed union of the fracture

C. Malunion of the fracture

D. The fracture has healed

E. Refracture after healing

64. A 24-year-old male with a gunshot wound to the left lower chest 1 hour ago. Chest X-ray: Disappearance of the left costophrenic angle, bullet retained in the abdominal cavity. The primary diagnosis is

A. Left lung injury

B. Left diaphragmatic injury

C. Left chest injury

D. Combined left chest and abdominal injury

E. Left abdominal injury

65. A 12-year-old girl hit by a car, severe right knee pain. Physical examination: Positive patellar tap test, normal X-ray, stress X-ray shows a 3 cm opening of the medial knee joint space. The treatment for this injury is

A. Aspirate the hematoma and apply a pressure bandage

B. Long leg cast in 20° flexion

C. Repair of the medial collateral ligament

D. Reconstruction of the medial collateral ligament using the semitendinosus tendon

E. Aspirate the hematoma and medication

66. A 15-year-old male with severe pain in the lower right thigh for 2 days, high fever up to 40°C, diagnosed with acute purulent osteomyelitis. The most appropriate treatment is

A. Bed rest, strict immobilization

B. High-dose broad-spectrum antibiotics

C. Intravenous high-dose antibiotics and drilling for drainage

D. Amputation

E. Early conservative treatment, bacterial culture, and application of sensitive antibiotics after pathogen identification

67. A 56-year-old male with sudden hematemesis for 2 hours, approximately 800 ml of blood. History of heavy drinking for 20 years. Physical examination: P 110/min, BP 90/70 mmHg, liver and spleen not palpable below the costal margin. Lab tests: RBC 3.0×10¹²/L, Hb 70 g/L, PLT 56×10⁹/L. The most likely diagnosis is

A. Hemorrhagic gastritis

B. Bleeding peptic ulcer

C. Ruptured esophageal and gastric varices

D. Bleeding gastric cancer

E. Biliary tract bleeding

68. A 28-year-old male with a 25-year history of right elbow joint malunion fracture, right hand muscle atrophy, and ulnar side sensory loss for 5 years. The best treatment is

A. Ulnar nerve decompression surgery

B. Use of neurotrophic drugs

C. Correction of the malunion fracture

D. Ulnar tunnel decompression surgery

E. No treatment needed

69. A 56-year-old male with neck and shoulder pain for 6 weeks, pain radiating to the right hand, reduced sensation in the right thumb, and weakened biceps muscle strength. The preliminary diagnosis is

A. Cervical spondylosis

B. Frozen shoulder

C. Rotator cuff syndrome

D. Brachial plexus neuritis

E. Cervical strain

70. An 18-month-old girl with sudden onset of paroxysmal crying, each episode lasting about 15 minutes, accompanied by hand and foot movements, pale face, refusal to eat, and a painful expression. Vomited several times during episodes. After about 4 hours, passed a small amount of stool with a jam-like appearance. Physical examination during intermittent crying: Right lower abdomen feels empty, palpable sausage-like mass that can be moved along the colon. Based on the child's symptoms and signs, the most likely diagnosis is

A. Congenital hypertrophic pyloric stenosis (CHPS)

B. Intestinal spasm

C. Acute necrotizing enterocolitis

D. Cecal volvulus

E. Intussusception

Correct answer: E

71. A 30-year-old female with pain on the lateral side of the right elbow for six months. Physical examination: Positive Mills sign on the right side. X-ray: No abnormalities. The key to treatment and prevention of recurrence of this disease is

A. Functional exercise

B. Early surgery

C. Limiting wrist joint activity

D. Medication

E. Conservative treatment

72. A 15-year-old middle school student diagnosed with "acute lymphoblastic leukemia" after bone marrow aspiration, received standard treatment with no symptom relief. The doctor informed the parents that there is no ideal treatment for this disease currently, but the hospital is testing a new drug with uncertain efficacy and some risks. The parents agreed to the experimental treatment but did not complete the written consent. The patient’s condition worsened and died after two days of treatment. The family later denied agreeing to the treatment and claimed the doctor was "experimenting on the patient," leading to a medical dispute. The correct description is

A. The parents did not provide written consent, and the doctor did not respect the parents' reservations

B. Delayed resuscitation efforts

C. The doctor must bear responsibility for the patient's death as the parents did not sign

D. The doctor made an error in the informed consent process as the parents did not sign

E. The doctor conducted the experiment to collect clinical data, misleading the parents into giving informed consent

73. A 72-year-old female with bilateral knee pain for more than 10 years, worsening for 3 months. Physical examination: No swelling in both knees, joint space tenderness, and crepitus during joint movement. X-ray: Narrowing of bilateral knee joint spaces, formation of osteophytes around the joints. The most likely diagnosis is

A. Rheumatoid arthritis

B. Osteoarthritis

C. Osteoporosis

D. Ankylosing spondylitis

E. Gout

74. Mr. Jin, a 78-year-old male, treated for pneumonia at a nearby clinic with no improvement until he fell into a coma and was taken to a large hospital. Diagnosed with lobar pneumonia and secondary toxic encephalopathy, he was treated in the emergency room due to a lack of beds in the internal medicine department. High-cost antibiotics and serum albumin were used, and after a week, his temperature normalized, and he progressed from deep coma to shallow coma, but the week’s medical expenses totaled 8000 yuan. His two retired daughters found the continued treatment unaffordable and requested to stop treatment. The most ethical course of action for the doctor is

A. To save healthcare resources, it is acceptable not to continue resuscitation for a patient with such low quality of life

B. Advise the family to use relatively low-cost and effective drugs for a few more days before making a decision

C. Respect the family's decision and stop all treatment

D. Provide free treatment to save the patient

E. Continue treatment regardless of the family's wishes

75. A patient with neck stiffness after a head injury from a heavy object, needs an X-ray to determine if there is a fracture of the atlas arch. The X-ray view that can confirm the diagnosis is

A. Anteroposterior view

B. Lateral view

C. Oblique view

D. Open mouth view

E. Tangential view

76. A 20-year-old male worker fractures his right humeral upper end while swatting a fly. The first consideration should be

A. Avulsion fracture of the greater tuberosity of the humerus

B. Surgical neck fracture of the humerus

C. Anatomical neck fracture of the humerus

D. Pathological fracture

E. Stress fracture

77. A 30-year-old male who enjoys horseback riding complains of pain on the inner side of the left leg. Physical examination: Localized tenderness in the proximal medial tibia, approximately 6.0 cm × 6.0 cm, with no other abnormalities. The most likely diagnosis is

A. Pes anserine bursitis

B. Meniscal injury

C. Cruciate ligament injury

D. Medial collateral ligament injury

E. Infrapatellar bursitis

78. A 21-year-old male with a painless mass in the right popliteal region for 2 years, recently experiencing local pain. Combined with right femur X-rays, the most likely diagnosis is

A. Myositis ossificans

B. Chondrosarcoma

C. Ossifying fibroma

D. Parosteal osteosarcoma

E. Tumoral calcinosis

79. In the dermatology clinic, a male patient diagnosed with gonorrhea asks the doctor not to tell his wife waiting outside the examination room to avoid a divorce. To avoid harming the couple, the dermatologist should

A. Not tell the patient's wife unless she asks

B. Tell the truth if the patient's wife asks

C. Lie if the patient's wife asks

D. Refuse to answer if the patient's wife asks

E. Suggest the patient tell his wife about the sexually transmitted disease himself

Correct answer: E

80. A 51-year-old male with a mid-shaft tibia and fibula fracture, treated with long leg cast immobilization. After 4 months, the cast is removed, and the patient has knee joint dysfunction. The most likely cause is

A. Muscle atrophy

B. Joint stiffness

C. Joint ankylosis

D. Poor fracture reduction

E. Malunion of the fracture

81. An 8-year-old girl with a mid-shaft femur fracture from a car accident. The best treatment is

A. Bryant traction with vertical suspension of the lower limb

B. Braun frame skin traction

C. Open reduction with plate fixation

D. Open reduction with elastic nails fixation

E. Open reduction with intramedullary nailing

82. A patient with a forearm fracture, treated with manual reduction and small splint fixation for 5 hours, experiences severe pain, finger numbness, swelling, and limited movement. The main cause is

A. Nerve injury

B. Nerve and venous compression

C. Arterial and venous compression

D. Venous compression

E. Arterial compression

83. A 30-year-old male injured in a car accident, found at the scene with a large amount of fresh blood flowing from a wound near the left groin. After immediate hemostasis, the first examination should be

A. Palpation of the dorsalis pedis and posterior tibial arteries

B. Examination of limb sensation

C. Checking knee or Achilles reflex

D. Checking for any loss of voluntary foot movement

E. Immediate X-ray examination

84. A 30-year-old male, 2 hours after a car accident, in generally good condition, with a 14 cm laceration on the upper right leg, severe soft tissue contusion, exposed tibial fracture ends, minimal bleeding. The appropriate treatment before X-ray examination is

A. Simple external fixation and local dressing

B. Application of a pneumatic tourniquet

C. Immediate transfer to the operating room

D. Plaster cast immobilization

E. Calcaneal traction

85. A male with a 1 cm × 2 cm skin defect on the palmar side of the right thumb, exposing the tendon. The appropriate treatment is

A. Free skin graft

B. Z-plasty

C. Direct skin closure

D. Shortening of the phalanx and skin closure

E. Pedicled flap graft

Correct answer: E

86. A 22-year-old female with left lower leg discomfort for 1 month, worsening in the past week. Physical examination: Temperature 37.6°C, no significant weight loss in the past month, normal skin temperature in the left lower leg, no obvious abnormalities, normal knee and ankle joint movement. X-ray: 6 cm long shadow in the left lower leg with a ground glass appearance. The best treatment is

A. Chemotherapy

B. Chemotherapy plus segmental resection

C. Curettage and bone grafting

D. Chemotherapy plus amputation

E. Segmental resection and reimplantation after inactivation

87. A 28-year-old male fell while running, landing on his left knee, experiencing severe pain and inability to move the knee. Physical examination: Swelling of the knee joint, a feeling of emptiness in front of the patella. The primary diagnosis should be

A. Anterior cruciate ligament injury

B. Patellar fracture

C. Medial tibial plateau fracture

D. Medial collateral ligament injury

E. Lateral meniscus injury

88. A 22-year-old male with low back pain radiating to the buttocks for 10 months. Physical examination: Stiffness of the lower back, no neurological abnormalities. Lab tests: Low hemoglobin, high erythrocyte sedimentation rate, HLA-B27 positive. X-ray: "Bamboo spine" appearance, narrowing of the sacroiliac joint space. The primary diagnosis should be

A. Rheumatic arthritis

B. Spinal tuberculosis

C. Ankylosing spondylitis

D. Lumbar disc herniation

E. Rheumatoid arthritis

89. A 28-year-old male with a fracture of the left ulna and radius. Physical examination: Inability to oppose the thumb. The most likely nerve injury is

A. Ulnar nerve

B. Radial nerve

C. Median nerve

D. Axillary nerve

E. Musculocutaneous nerve

90. A hospital admitted several AIDS patients, and due to the lack of effective antiviral drugs, the doctors, unwilling to leave the patients untreated, had them take a newly developed antiviral drug from a research institute without informing the patients about the drug development process. The patients felt very uncomfortable after taking the drug and refused to continue after the first course of treatment. The correct evaluation of this case is

A. The doctors violated the principle of informed consent in human experimentation

B. The doctors violated the principle of having emergency measures in place to prevent accidents

C. The drug had not undergone sufficient, reliable animal testing

D. It is morally acceptable to use any experimental treatment for patients with no available cure

E. Subjects cannot withdraw from the experiment before it is completed

91. A 30-year-old female with an elbow injury 3 hours ago. Physical examination: Quadriplegia, complete loss of sensation, movement, and reflexes in the limbs, normal sensation above the acromion. The injury level is likely

A. Between C2 and C3

B. Between C3 and C4

C. Between C4 and C5

D. Between C5 and C6

E. Between C6 and C7

92. A 7-year-old boy with sudden chills and high fever (39.8°C), irritability, severe pain below the right knee, the knee joint in a semi-flexed position, refusing to move. Physical examination: High skin temperature and tenderness in the proximal right lower leg, aspiration of cloudy fluid from the affected area sent for bacterial culture. The most likely result is

A. Beta-hemolytic streptococcus

B. Staphylococcus aureus

C. Escherichia coli

D. Streptococcus

E. No bacterial growth

93. A young patient with a knife wound to the left wrist 1 hour ago. Reduced wrist flexor strength, impaired flexion of the index and middle fingers, weakened flexion of the ring and little fingers. Surgical exploration reveals injury to the flexor retinaculum and deep tendons. The most likely postoperative complication is

A. Ischemic contracture of the left hand

B. Compartment syndrome

C. Tendon adhesion

D. Vascular embolism

E. Claw hand deformity

94. A female with a stab wound to the middle of the left groin 1 hour ago, with significant bleeding at the wound site. The first examination should be

A. Checking for voluntary foot movement

B. Checking knee or Achilles reflex

C. Confirming stable vital signs, observing and palpating the dorsalis pedis and posterior tibial arteries

D. Checking for sensory loss in the limb

E. Immediate X-ray examination

95. A 31-year-old female with a lateral knee injury. Physical examination: Inability to actively dorsiflex the ankle. X-ray: Fracture of the left fibular head. The correct diagnosis is

A. Left fibular head fracture with tibial nerve injury

B. Left fibular head fracture with sciatic nerve injury

C. Left fibular head fracture with common peroneal nerve injury

D. Left fibular head fracture with anterior tibial muscle injury

E. Left fibular head fracture with tear of the fibular longus and brevis

96. A 20-year-old male with a right thigh mass for 2 months. During physical examination, the correct principles not followed were

A. Examine the healthy side first, then the affected side

B. Examine distal to proximal

C. Examine passive movement before active movement

D. Compare both sides when exposed

E. Be gentle to minimize patient discomfort

97. A 10-year-old child with chronic osteomyelitis of the proximal tibia, in good general condition, afebrile, with a draining sinus tract locally, and a 4 cm long sequestrum with a discontinuous involucrum on X-ray. The primary treatment is

A. Surgical removal of the sequestrum, muscle flap to fill the dead space

B. Surgical removal of the sequestrum, filling with gentamicin, and removal after 3 weeks, followed by cancellous bone grafting

C. Surgical removal of the sequestrum, insertion of fine plastic tubes, daily antibiotic irrigation through the tubes

D. Long leg cast immobilization, window dressing changes

E. Local dressing changes to maintain drainage

98. A 53-year-old female with acute diffuse peritonitis, observed for 2.5 hours with worsening symptoms, persistent abdominal distension and pain, and an undetermined cause. The immediate treatment measures should include all except

A. Fasting, gastric decompression

B. High-dose broad-spectrum antibiotics

C. Immediate exploratory laparotomy

D. Symptomatic treatment with antispasmodics and analgesics

E. Fluid resuscitation and correction of acidosis

99. A 50-year-old male with subcutaneous nodules on the palm for over 2 years, painless but with morning stiffness. Later developed cords extending to fingers, causing flexion contractures. The most likely diagnosis is

A. Subcutaneous cyst

B. Lipoma

C. Palmar scar contracture deformity

D. Dupuytren's contracture

E. Tenosynovitis

100. A 31-year-old female with a lateral knee injury. Physical examination: Inability to actively dorsiflex the ankle. X-ray: Fracture of the left fibular head. The correct diagnosis is

A. Left fibular head fracture with tibial nerve injury

B. Left fibular head fracture with sciatic nerve injury

C. Left fibular head fracture with common peroneal nerve injury

D. Left fibular head fracture with anterior tibial muscle injury

E. Left fibular head fracture with tear of the fibular longus and brevis

101. A 78-year-old male patient was admitted to the hospital due to an intertrochanteric fracture of the right femur and multiple rib fractures caused by a car accident. The patient was only treated with a chest band fixation and right lower limb skin traction. The most important complication to be vigilant about and prevent for this patient is:

A. Avascular necrosis of the femoral head

B. Fat embolism

C. Hypostatic pneumonia

D. Major vascular and nerve injury

E. Traumatic myositis ossificans

102. A 24-year-old male fell from an 8-meter height, landing on his arm, and was admitted to the hospital 12 hours after the injury. He has significantly reduced or lost sensation below three fingers from the navel, and except for slight movement of the right big toe, there is a loss of voluntary movement in both lower limbs, and urinary retention. X-ray shows compression of the T10 vertebra with bone fragments protruding into the spinal canal. The key early treatment is:

A. Active and aggressive antibiotic treatment

B. Catheterization with an indwelling catheter

C. Good nursing care and frequent repositioning to prevent bedsores

D. Early surgery to relieve compression

E. Reduction with the high-low table method followed by a plaster vest

103. An 18-year-old female cyclist has had pain under the patella for 4 months without obvious cause, which worsens with activity and is relieved by rest. The pain has progressively worsened, causing knee weakness and difficulty climbing stairs. The most likely diagnosis is:

A. Osgood-Schlatter disease

B. Chondromalacia patellae

C. Cruciate ligament injury

D. Meniscal injury

E. Collateral ligament injury

104. A 49-year-old male patient with advanced gastric cancer, who believes he has gastritis and hopes for a cure, frequently rings the bell for assistance when in discomfort. Medical staff have avoided him multiple times, causing great disappointment. When the patient requests pain relief and is refused, the best approach according to medical ethics is:

A. Fulfill all the patient's requests

B. Meet some of the patient's requests to alleviate pain

C. Explain the truth and inform the patient that treatment is not possible

D. Continue to conceal the diagnosis and provide passive treatment

E. Inform the patient of the truth with the family's consent at an appropriate time, fulfill the patient's requests as much as possible, and alleviate pain

105. A 60-year-old male laborer presents with intermittent claudication for one year, worsening over the past month, with no history of smoking, alcohol use, or trauma. The most likely physical finding is:

A. Positive straight leg raise test

B. Weakening of the dorsalis pedis pulse

C. Cyanosis of the lips

D. Significant tenderness in the lumbar and gluteal regions

E. No obvious positive signs in the lumbar and leg areas

106. A 41-year-old female was hit on the right knee with a blunt object, resulting in a fibular head fracture. She cannot actively dorsiflex her ankle, suggesting:

A. Common peroneal nerve injury

B. Sciatic nerve injury

C. Tibial nerve injury

D. Anterior tibialis muscle rupture

E. Gastrocnemius muscle tear

107. A 68-year-old female with left knee osteoarthritis and deformities underwent total knee replacement. Postoperatively, she experienced numbness in the dorsal foot and weakness in ankle dorsiflexion. The possible complication is:

A. Prosthesis dislocation

B. Tight dressing

C. Anterior tibial artery injury

D. Common peroneal nerve palsy

E. Tibial nerve injury

108. A 20-year-old male with a left scaphoid fracture treated with manual reduction and cast immobilization for 6 months shows non-union with sclerosis on follow-up X-ray, possibly due to:

A. Poor blood supply to the fracture site

B. Soft tissue interposition at the fracture site

C. Severe soft tissue injury

D. Improper treatment methods

E. Older age of the patient

109. A 36-year-old male had the distal phalanx of his right middle finger amputated by a machine, exposing the bone. The least suitable treatment is:

A. Bone shortening and skin suturing

B. Free split-thickness skin graft

C. Pedicle flap graft

D. Bilateral advancement flap formation

E. Temporary dressing and observation after debridement if the wound is severely contaminated

110. A 20-year-old badminton player has had lateral elbow pain for six months, worsening with activity. Examination reveals tenderness on the lateral elbow but normal joint function. The most likely diagnosis is:

A. Radial nerve injury

B. Old elbow dislocation

C. Old supracondylar fracture of the humerus

D. Chronic suppurative osteomyelitis of the humerus

E. Lateral epicondylitis

111. A 5-year-old boy with lumbar tuberculosis for one month is likely to have a positive result in:

A. McMurray test

B. Thomas sign

C. Gaenslen test

D. Picking-up test

E. Dugas sign

112. A 40-year-old female patient with tibial plateau or femoral condyle fractures has joint surface irregularities of 0.8 cm despite treatment. The most likely late complication is:

A. Myositis ossificans

B. Poor bone healing

C. Traumatic arthritis of the knee

D. Knee joint stiffness

E. Valgus deformity of the knee

113. A male patient with a right humeral shaft fracture caused by trauma shows a 4 cm shortening of the upper arm, slight angulation, and reduced bone conduction sound. The most likely type of fracture displacement is:

A. Shortening displacement

B. Mixed displacement

C. Lateral displacement

D. Angulation displacement

E. Separation displacement

114. A 28-year-old female with scar contracture deformity of the right hand for two years after a burn injury shows exposure of vessels, tendons, nerves, and joint capsules after scar excision. The preferred method of repairing the wound is:

A. Flap grafting

B. Split-thickness skin grafting

C. Forearm flap repair

D. Abdominal flap repair

E. Dorsal foot flap repair

115. A 20-year-old male with right shoulder pain after a fall shows swelling and tenderness at the acromioclavicular joint with no palpable elastic fixation and normal X-ray findings. The correct treatment is:

A. Sling suspension

B. Figure-of-eight bandage fixation

C. No treatment

D. Bed rest for 2 weeks

E. Small shoulder splint fixation

116. A 70-year-old male with a 10-year history of rheumatoid arthritis presents with left lower limb pain after walking for two years, worsening in the past month. Examination shows reduced sensation on the lateral left calf and decreased strength in the left extensor muscle, with a positive straight leg raise test at 35° and mild disc narrowing on X-ray. The most likely diagnosis is:

A. Lumbar muscle strain

B. Lumbar spondylosis

C. Lumbar spondylolysis

D. L4-5 disc herniation

E. Obliterative arteritis

117. A 68-year-old female with left knee osteoarthritis and deformities underwent total knee replacement. Postoperatively, she experienced numbness in the dorsal foot and weakness in ankle dorsiflexion. The possible complication is:

A. Prosthesis dislocation

B. Tight dressing

C. Anterior tibial artery injury

D. Common peroneal nerve palsy

E. Tibial nerve injury

118. A 28-year-old male with low back pain and left lower limb radiating pain for a week after lifting heavy objects shows loss of lumbar lordosis, restricted mobility, and a positive straight leg raise test at 30°. X-ray shows loss of lumbar lordosis. The most likely diagnosis is:

A. Acute lumbar facet joint dysfunction

B. Acute lumbar sprain

C. L4-5 disc herniation

D. L5-S1 disc herniation

E. Lumbar spondylolysis

119. A 25-year-old male with a closed fracture of the left femoral shaft without significant displacement shows a transverse fracture on X-ray. The first choice of treatment is:

A. Manual reduction, small splint fixation, and weight traction

B. Manual reduction and plaster fixation

C. External fixator fixation

D. Open reduction and internal fixation

E. Bed rest with strict immobilization

120. An 18-year-old female with low-grade fever, night sweats, and back pain for two months shows a mass in the right iliac fossa on ultrasound, with limited hip flexion and extension. The diagnosis is likely:

A. Vertebral metastasis

B. Pelvic tumor

C. Lumbar tuberculosis

D. Knee tuberculosis

E. Hip tuberculosis

121. A 25-year-old male with an open comminuted fracture of the right tibia and fibula underwent thorough debridement and traction fixation, but the fracture remains unhealed after 3 months. The most likely cause is:

A. Poor blood supply to the fracture site

B. Inadequate limb fixation

C. Insufficient functional exercises

D. Failure to perform timely open reduction and fixation

E. Excessive removal of bone fragments during debridement

122. A 30-year-old male with a left femoral shaft fracture shows posterior displacement of the distal fracture end on X-ray. The main factor causing this displacement is:

A. Muscle traction

B. Nature of the force

C. Magnitude of the force

D. Weight of the distal limb segment

E. Improper handling

123. A 41-year-old male with right knee pain and swelling for six months shows a 4 cm×6 cm translucent area with a "soap bubble" appearance on X-ray. The lesion has enlarged, and the "soap bubble" appearance has disappeared, showing a cloudy shadow. The incorrect treatment choice is:

A. Autologous bone joint transplantation

B. Curettage, bone grafting, and cement filling

C. Allograft and artificial joint composite transplantation

D. Amputation

E. Allograft partial and total joint replacement

124. A 25-year-old male with right knee instability and a sense of dislocation two weeks after a knee injury shows positive McMurray and negative drawer and side stress tests. The most likely diagnosis is:

A. Medial collateral ligament rupture

B. Medial meniscus injury

C. Anterior cruciate ligament rupture

D. Intra-articular loose body

E. Osteomalacia

125. A 38-year-old male with intermittent left low back pain for a year shows red blood cells in the urine. The best examination to confirm upper urinary tract stones and guide treatment is:

A. Ultrasound

B. Plain film of the urinary system (KUB)

C. Intravenous pyelography (IVP)

D. KUB + IVP

E. CT scan

126. An 8-year-old male with left upper arm pain after trauma shows a diagnosis of:

A. Osteosarcoma

B. Ewing's sarcoma

C. Bone cyst

D. Langerhans cell histiocytosis

E. Non-ossifying fibroma

127. An 18-year-old male with right knee and lower leg injuries from a car accident shows a loss of plantar flexion, inversion, and adduction functions, and loss of sensation on the sole. The most likely injured nerve is:

A. Sural nerve

B. Femoral nerve

C. Common peroneal nerve

D. Tibial nerve

E. Sciatic nerve

128. A 56-year-old male with right knee pain for six months, worsening over five days, especially when climbing stairs, is diagnosed with chondromalacia patellae. The initial non-surgical treatment should immobilize the knee for:

A. 1-2 weeks

B. 3-4 weeks

C. 4-5 weeks

D. 6-7 weeks

E. 8-9 weeks

129. A 20-year-old male laborer experienced sudden severe low back pain during loading and unloading, followed by bilateral buttock pain after three days of bed rest. Normal neurological examination suggests:

A. Lumbar disc herniation

B. Lumbar myofascitis

C. Lumbar muscle strain

D. Acute lumbar sprain

E. Lumbar vertebra fracture

130. A 45-year-old female patient with a head injury from a traffic accident became comatose immediately after the injury and was admitted an hour later in moderate coma with a dilated right pupil and absent light reflex, and positive pathological signs in the left limbs. The first measure to take is:

A. Administer hemostatic drugs

B. Infuse 250 ml of 20% mannitol

C. Administer antibiotics to prevent infection

D. Infuse 20 mg of dexamethasone

E. Administer respiratory stimulants

131. A 20-year-old male patient with a right thigh amputation due to a traffic accident needs to reduce stump swelling, minimize bed complications, and start early training. The most important treatment is:

A. Elastic bandage wrapping

B. ADL (activities of daily living) training

C. Enhance stump skin care

D. Stump muscle strength training

E. Fit a temporary prosthesis

132. A 21-year-old male with left upper leg swelling and pain for six months, worsening in the last month, affecting sleep, shows a 6 cm×7 cm hard, fixed, ill-defined mass with significant tenderness and engorged superficial veins on examination. X-ray reveals moth-eaten appearance and significant periosteal reaction with "Codman" triangle in the upper tibia. The first-line chemotherapy regimen should be:

A. Adriamycin + vincristine, cyclophosphamide + adriamycin

B. Vincristine + cyclophosphamide

C. Melphalan + prednisone

D. Adriamycin + cisplatin + methotrexate + ifosfamide

E. Cyclophosphamide + adriamycin + vincristine

133. An 18-year-old male with intermittent left thigh pain and swelling with fever for three years, diagnosed with chronic osteomyelitis of the femur, shows a thickened cortex and a 30 mm×25 mm×25 mm cavity with a 20 mm×15 mm×20 mm sequestrum on X-ray. The best treatment is:

A. Local heat application and oral antibiotics

B. Sequestrectomy with open wound dressing

C. Sequestrectomy with saucerization

D. Sequestrectomy with pedicled muscle flap filling and wound closure

E. Sequestrectomy with closed irrigation and suction therapy

134. A 20-year-old male with left knee pain and swelling after a soccer injury, showing joint locking and giving way, has a positive McMurray sign, and no positive drawer or side stress tests. The most likely diagnosis is:

A. Medial collateral ligament rupture

B. Medial meniscus injury

C. Anterior cruciate ligament rupture

D. Intra-articular loose body

E. Osteomalacia

135. A 9-year-old girl with sudden right hip pain and high fever of 39.5°C, with 22×10⁹/L white blood cells and 98% neutrophils, shows hip swelling and reluctance to move. The most likely diagnosis is:

A. Acute pyogenic arthritis

B. Acute rheumatic arthritis

C. Rheumatoid arthritis

D. Periarticular tissue inflammation

E. Hip tuberculosis

136. A 65-year-old male with an old femoral neck fracture and avascular necrosis of the femoral head should be treated with:

A. Hip joint fusion

B. Intertrochanteric osteotomy

C. Femoral head drilling and bone grafting

D. Total hip replacement

E. Medication

137. A 40-year-old female patient with a tibial plateau tumor resection should receive a knee replacement with:

A. Condylar-constrained knee prosthesis

B. Hinged knee prosthesis

C. Rotating-hinged knee prosthesis

D. Ball-and-socket knee prosthesis

E. Posterior-stabilized knee prosthesis

138. A 4-year-old boy with a transverse femoral shaft fracture and overlapping displacement should be treated with:

A. Vertical suspension skin traction

B. Manual reduction with small splint fixation

C. Manual reduction with small splint fixation and skin traction

D. Open reduction and internal fixation

E. Manual reduction with bone traction

139. A male with lower abdominal injury from a falling cart shows tenderness at the pubic symphysis and a positive compression test with a full bladder, but no urine can be drawn despite rubber catheter insertion with blood at the catheter tip. Consider:

A. Insufficient catheter insertion depth

B. Incorrect catheter insertion method

C. Catheter obstruction

D. Pelvic fracture with urethral rupture

E. Pelvic fracture with bladder injury

140. A 26-year-old male with sudden hematemesis and abdominal pain after drinking two bottles of cold beer is most likely diagnosed with:

A. Gastric mucosal prolapse

B. Gastric ulcer

C. Duodenal ulcer

D. Acute erosive hemorrhagic gastritis

E. Mallory-Weiss syndrome

141. A 30-year-old pregnant woman at 30 weeks with upper abdominal colic, nausea, vomiting, and lower abdominal pain shows elevated white blood cells and bilirubin, blood potassium of 3.1 mmol/L, and amylase of 4854 U/L. Ultrasound shows multiple gallstones and cholecystitis, with normal fetal development. The incorrect treatment is:

A. Immediate surgery upon diagnosis

B. Cesarean section

C. Conservative treatment

D. Conservative treatment after terminating pregnancy

E. Surgery after terminating pregnancy

142. A 30-year-old male with an upper abdominal mass and vomiting but no pain or fever shows a 9 cm×10 cm cystic mass on examination, diagnosed as a pancreatic pseudocyst. The best treatment is:

A. Aspiration of cyst contents

B. Surgical cyst excision

C. External cyst drainage

D. Internal cyst drainage

E. Observation without treatment

143. A 45-year-old male with right neck and shoulder pain and right upper limb numbness after exposure to cold shows positive right brachial plexus traction test and reduced pain sensation in the right middle finger. The affected nerve root is:

A. C4

B. C5

C. C6

D. C7

E. C8

144. A 6-year-old child with a greenstick fracture of the clavicle should be treated with:

A. Sling suspension for 3 weeks

B. Manual reduction and figure-of-eight bandage fixation

C. Surgical reduction and internal fixation

D. No treatment

E. Bed rest for 2 weeks

145. A 20-year-old male with incomplete quadriplegia from a bicycle fall shows a dens fracture with subluxation on X-ray. The first treatment measure is:

A. Plaster collar fixation

B. Skull traction fixation

C. Sling traction

D. Occipitocervical fusion

E. Open reduction and internal fixation

146. A 33-year-old male with sudden headache and right eyelid ptosis, right pupil dilation, and absent direct and consensual light reflex, but normal consensual light reflex on the left side. The likely site of injury is:

A. Left optic and oculomotor nerves

B. Left oculomotor nerve

C. Right oculomotor nerve

D. Specific projection system

E. Non-specific projection system

147. A 36-year-old female with a 1.6 cm×1.2 cm soft tissue defect on the ulnar side of the right thumb pad with exposed bone after a machine injury and 12 hours of wound contamination should be treated by:

A. Primary split-thickness skin graft after debridement

B. Primary closure with bone shortening after debridement

C. Primary island flap from the index finger after debridement

D. Temporary coverage with artificial skin or Vaseline gauze followed by debridement and island flap from the index finger after 72 hours

E. Free flap graft after debridement

148. A 50-year-old female with periumbilical pain for 12 hours radiating to the entire abdomen for 2 hours shows right-sided abdominal tenderness, rebound tenderness, muscle guarding, and purulent fluid with a slight odor on diagnostic abdominal puncture. The most likely diagnosis is:

A. Acute appendicitis with perforation

B. Strangulated intestinal obstruction

C. Tuberculous peritonitis

D. Severe acute pancreatitis

E. Intra-abdominal hemorrhage

149. A 30-year-old male with right elbow pain and limited movement for 2 hours after a car accident shows swelling, deformity, and tenderness in the elbow with disappeared olecranon triangle, but normal sensation and radial pulse in the right hand and forearm. The most likely diagnosis is:

A. Supracondylar fracture of the humerus (extension type)

B. Supracondylar fracture of the humerus (flexion type)

C. Terrible triad of the elbow

D. Radial head subluxation

E. Olecranon fracture

150. A 68-year-old male with urinary incontinence one month after transurethral resection of the prostate (TURP) shows continuous urine leakage without bladder distention. This condition most likely indicates:

A. Overflow incontinence

B. True incontinence

C. Urge incontinence

D. Psychogenic incontinence

E. Stress incontinence

151. A 48-year-old female with left shoulder pain for 3 months, worsening gradually, unable to comb her hair, shows limited abduction and extension of the left shoulder with deltoid muscle atrophy but no arm numbness. The most likely diagnosis is:

A. Cervical spondylosis

B. Shoulder tumor

C. Adhesive capsulitis

D. Shoulder joint tuberculosis

E. Rheumatoid arthritis

152. A 20-year-old male with an obvious displacement of the right midshaft clavicular fracture maintains his head tilted to the right side and supports his right elbow with his left hand due to:

A. Soft tissue sprain in the right neck and shoulder area causing a forced position

B. Brachial plexus injury with decreased muscle strength in the right upper limb

C. Subclavian vessel injury reducing blood supply to the upper limb

D. Temporary immobilization to reduce bleeding at the fracture site

E. Relaxation of muscles and reduction of upper limb weight traction to alleviate fracture pain

153. For the same patient, surgery for open reduction and internal fixation should be considered if:

A. The patient is young and has aesthetic demands

B. Significant overlapping and shortening of the fracture ends

C. Two-thirds lateral displacement of the fracture ends after reduction

D. Fracture ends cannot contact due to obstruction after reduction

E. Numbness in the affected limb appears after manual reduction and plaster fixation

154. During manual closed reduction for the same patient, the right upper limb should be pulled:

A. Upward, backward, and outward

B. Upward and outward

C. Backward and downward

D. Forward and downward

E. Forward and upward

155. For a 60-year-old male laborer with intermittent claudication for one year, worsening for one month with no smoking, alcohol, or trauma history, the statement is correct:

A. Lumbar disc herniation on the medial side of the nerve root can be relieved by spinal curvature towards the healthy side

B. Lumbar disc herniation on the lateral side of the nerve root can be relieved by spinal curvature towards the healthy side

C. Lumbar disc herniation outside the intervertebral foramen can be relieved by spinal curvature towards the affected side

D. Lumbar disc herniation outside the intervertebral foramen can be relieved by spinal curvature towards the healthy side

E. Lumbar disc herniation on the medial side of the nerve root cannot be relieved by spinal curvature on either side

156. The most common type of scoliosis is:

A. Idiopathic scoliosis

B. Congenital scoliosis

C. Neuromuscular scoliosis

D. Neurofibromatosis

E. Mesenchymal tissue disease

157. A 42-year-old female accountant with neck discomfort and soreness for 2 months, progressing to neck and shoulder pain radiating to the arm, with electric shock-like pain when turning the neck to a certain angle. The most likely diagnosis is:

A. Cervical spondylosis of the nerve root type

B. Adhesive capsulitis

C. Atlantoaxial subluxation

D. Cervical disc herniation

E. Cervical spinal stenosis

158. For the same patient, the most significant physical examination finding is:

A. Neck tenderness

B. Positive upper limb traction test

C. Limited neck movement

D. Abnormal sensation in the upper limb

E. Decreased muscle strength in the upper limb

159. The most significant imaging examination for the same patient is:

A. Open-mouth view

B. Anteroposterior view of the cervical spine

C. Three-view cervical spine X-ray

D. Cervical spine extension X-ray

E. Cervical spine flexion X-ray

160. The first-line treatment for the same patient is:

A. Oral non-steroidal anti-inflammatory drugs (NSAIDs)

B. Pain point injection

C. Manipulative therapy

D. Cervical traction

E. Physical therapy

161. A 35-year-old female with right shoulder pain and inability to move it after falling backward and landing on her palm shows a square shoulder deformity and positive Dugas sign. The clinical diagnosis to consider first is:

A. Soft tissue injury around the right shoulder

B. Anterior dislocation of the right shoulder

C. Fracture of the humeral lateral epicondyle

D. Fracture of the humeral anatomical neck

E. Acromioclavicular joint dislocation

162. For the same patient, the first auxiliary examination to perform is:

A. Anteroposterior X-ray

B. Anteroposterior and lateral X-ray

C. Anteroposterior and trans-thoracic X-ray

D. CT scan

E. Shoulder arthroscopy

163. A 50-year-old female with left shoulder pain radiating to the upper arm, restricted shoulder movement for more than a month, worsening with night pain affecting sleep, and a history of left shoulder trauma. Examination shows muscle atrophy, restricted shoulder movement, especially during abduction and rotation, with localized tenderness but no obvious deformity. The most likely diagnosis is:

A. Soft tissue contusion of the left shoulder

B. Rheumatic arthritis of the left shoulder

C. Muscle strain of the left shoulder

D. Periarthritis of the left shoulder

E. Cervical spondylosis

164. For the same patient, the most helpful examination to confirm the diagnosis is:

A. X-ray examination

B. Electromyography (EMG)

C. CT and MRI scans

D. Arthroscopy

E. CT scan

165. The best treatment for the same patient is:

A. Immobilization of the affected side

B. Traditional Chinese medicine

C. Oral pain relief medication

D. Local injection of prednisone acetate

E. Active shoulder joint exercises with auxiliary physical therapy and massage

166. A patient with right hip pain and movement restriction after a car's sudden stop causing a knee impact, presenting a flexion, adduction, and internal rotation deformity of the hip, likely has:

A. Femoral neck fracture

B. Intertrochanteric fracture of the femur

C. Subtrochanteric fracture of the femur

D. Posterior hip dislocation

E. Anterior hip dislocation

167. For the same patient, possible associated injuries include:

A. Sciatic nerve injury

B. Femoral nerve injury

C. Obturator nerve injury

D. Tibial nerve injury

E. Common peroneal nerve injury

168. The best treatment method for the same patient is:

A. Hippocrates method

B. Kocher method

C. Allis method

D. Bone traction

E. Skin traction

169. For a 25-year-old male with an open comminuted fracture of the right tibia and fibula after a bicycle accident, the most important action during emergency treatment is:

A. Bandage the wound and temporarily immobilize before rapid transport

B. Rapid transport

C. Attempt reduction

D. Proper immobilization

E. Correct use of a tourniquet

170. The most suitable fixation method for the same patient's fracture is:

A. Plaster splint

B. Windowed plaster cast with traction pin

C. External fixation frame

D. Plate and screw internal fixation

E. Intramedullary nail internal fixation

171. A 60-year-old male with a right hip injury from a fall shows shortening and 50° external rotation deformity of the right lower limb, with no obvious swelling but positive percussion pain. The first examination to perform is:

A. X-ray

B. CT scan

C. MRI scan

D. Bone scan

E. Arthrography

172. If the examination shows a Garden type I femoral neck fracture, the best treatment plan is:

A. Emergency open reduction and internal fixation with three cannulated screws

B. Right lower limb skin traction

C. Total hip replacement

D. Partial hip replacement

E. Reduction and plaster fixation

173. One year later, the same patient has right hip pain during movement, and X-ray shows increased femoral head density and unclear trabeculae. This suggests:

A. Proliferative changes in the femoral head

B. Traumatic arthritis

C. Avascular necrosis of the femoral head

D. Degenerative arthritis

E. Degenerative changes in the femoral head

174. For this patient undergoing total hip replacement, the acetabular anteversion angle should be:

A. 10°-15°

B. Greater than 60°

C. Less than 40°

D. 40°-60°

E. 40°-50°

175. A 20-year-old female with right lower leg pain for two months, worsening with fever for one week, shows significant pain and increased local temperature in the right tibia, with an X-ray revealing bone destruction and "onion skin" periosteal reaction. The diagnosis of Ewing's sarcoma needs differentiation from:

A. Bone tuberculosis

B. Pyogenic osteomyelitis

C. Bone cyst

D. Fibrosarcoma

E. Fibrous dysplasia

176. For the same patient, the most valuable diagnostic and differential diagnostic auxiliary examination is:

A. X-ray

B. Bone scan

C. CT scan

D. White blood cell count and differential

E. Tissue biopsy and pathological examination

177. The best treatment for the same patient is:

A. Chemotherapy

B. Radiotherapy

C. Surgery + chemotherapy

D. Surgery + radiotherapy

E. Surgery + chemotherapy + radiotherapy

178. A 45-year-old male with severe bilateral thigh swelling and deformity from a car accident shows normal sensation and blood supply below the knees, with X-ray revealing severe comminuted fractures of both femoral shafts. The most likely complication to monitor for is:

A. Shock

B. Vascular injury

C. Nerve injury

D. Compartment syndrome

E. Ischemic bone necrosis

179. One week after skeletal traction, X-ray shows poor alignment of both fractures. The best treatment approach is:

A. Adjust traction angle and weight

B. Change to small splint external fixation

C. Small splint external fixation + skeletal traction

D. Manual reduction + bilateral hip spica cast

E. Open reduction and internal fixation

180. One year after systematic physiotherapy and functional exercises, the patient shows good recovery of right knee function, but no improvement in the left knee, with no abnormalities on X-ray. The most likely cause is:

A. Traumatic arthritis

B. Joint stiffness

C. Joint ankylosis

D. Internal fixation affecting joint movement

E. Severe soft tissue injury

181. The best approach for the patient's current condition is:

A. Continue physiotherapy + functional exercises

B. Combine with herbal medicine treatment

C. Surgical release of periarticular adhesions

D. Left knee joint fusion

E. Total knee replacement

182. A 4-year-old boy refusing to use or move his left elbow after falling from a 50 cm high bed shows no obvious deformity. The first examination to perform is:

A. X-ray

B. Electrophysiological testing

C. CT scan

D. Ultrasound

E. MRI

F. ECG

183. If the same child shows elbow deformity on examination, the first examination to perform is:

A. Anteroposterior and lateral X-ray of the humerus

B. Anteroposterior and lateral X-ray of the elbow

C. Anteroposterior and lateral X-ray of the radius and ulna

D. CT scan of the humerus

E. MRI of the elbow

F. CT scan of the elbow

184. If X-ray is negative for the same child, the most likely diagnosis is:

A. Supracondylar fracture of the humerus

B. Shoulder dislocation

C. Elbow dislocation

D. Radial head subluxation

E. Soft tissue injury of the elbow

F. Olecranon fracture

185. The typical age of onset for the same condition is:

A. Under 5 years

B. 3-8 years

C. 5-10 years

D. 10-12 years

E. 12-15 years

F. 15-18 years

186. The most likely positive sign for the same condition is:

A. Abnormal relationship of the elbow triangle

B. Elbow swelling

C. Positive Dugas sign

D. Tenderness of the radial head

E. Elbow varus

F. Elbow valgus

187. Further treatment for the same condition is:

A. Shoulder CT

B. Shoulder X-ray

C. Elbow CT

D. Manual reduction without fixation

E. Manual reduction with sling fixation

F. Elbow MRI

188. The contributing factor for the same condition is:

A. Anterior tilt angle between the axis of the humerus and the axis of the condyle

B. Rotational external force on the forearm

C. Weak annular ligament around the radial neck

D. Abduction and external rotation forces acting simultaneously on the humeral head

E. Carrying angle between the humeral axis and the ulnar axis

F. Ligament rupture

189. If the same condition recurs after three months due to pulling on the left hand, the best treatment is:

A. Cast fixation

B. Splint fixation

C. Sling suspension

D. Manual reduction followed by sling suspension

E. Surgical open reduction

F. Observation

190. A 36-year-old male with left knee pain for one year, worsening in the last month with swelling and limited movement, shows a clear boundary, elastic bone shell (ping-pong ball) mass below the knee on examination, with X-ray findings as shown (image not provided). The most likely diagnosis is:

A. Giant cell tumor of bone

B. Ewing's sarcoma

C. Osteoid osteoma

D. Chondrosarcoma

E. Osteosarcoma

F. Osteochondroma

191. About giant cell tumor of bone, correct statements include:

A. It is a malignant tumor

B. Commonly occurs between ages 20-40

C. Can metastasize to the lungs

D. Some patients present with pathological fractures

E. Often associated with joint dysfunction

F. Commonly occurs at the epiphyseal end of long bones and vertebrae

192. Further auxiliary examinations for the same patient should include:

A. ECG

B. Complete blood count

C. Blood biochemistry

D. MRI

E. CT scan

F. Pathological examination

193. Correct statements about treatment for the same patient include:

A. The goal is to completely remove the tumor

B. Prevent recurrence

C. Preserve limb function

D. Focus on radiotherapy

E. Focus on chemotherapy

F. Focus on surgical treatment

194. A 20-year-old male with right knee pain, swelling, and limited movement after a basketball injury shows a positive floating patella test. To confirm the diagnosis, the examination should include:

A. Thomas sign

B. Drawer test and Lachman test

C. "4" sign test

D. Single leg stand test

E. Straight leg raise test

F. Femoral nerve traction test

195. A positive lateral stress test for the same patient indicates:

A. Medial collateral ligament injury

B. Anterior cruciate ligament injury

C. Posterior cruciate ligament injury

D. Meniscal injury

E. Lateral collateral ligament injury

F. Discoid meniscus

196. If X-ray shows no obvious fracture of the right knee, the examination to confirm the diagnosis is:

A. X-ray

B. MRI

C. Ultrasound

D. CT scan

E. Electrophysiological testing

F. ECG

197. A positive drawer test and Lachman test for the same patient suggest:

A. Right knee meniscal injury

B. Right knee anterior cruciate ligament injury

C. Right knee two-ligament injury

D. Right knee posterior cruciate ligament injury

E. Right knee three-ligament injury

F. Right knee discoid meniscus

198. O'Donoghue's triad for knee injury does not include:

A. Anterior cruciate ligament

B. Medial meniscus

C. Medial collateral ligament

D. Lateral collateral ligament

E. Tibial plateau

F. Distal femur

199. Common types of knee injuries do not include:

A. Flexion, abduction, and external rotation injury

B. Anteroposterior displacement injury

C. Hyperextension injury

D. Flexion, adduction, and external rotation injury

E. Excessive abduction

F. Flexion, abduction, and internal rotation injury

200. The function of the posterior cruciate ligament does not include:

A. Limiting posterior displacement of the tibia

B. Preventing hyperflexion and external rotation of the knee

C. Preventing hyperextension and internal rotation of the knee

D. Controlling abduction and adduction

E. Limiting hyperextension of the knee

F. Limiting hyperflexion of the knee

201. For a patient with a combined injury of the medial collateral ligament, the most appropriate treatment is:

A. Plaster splint external fixation

B. Arthroscopic exploration and repair

C. Oral pain relief medication and bed rest

D. Local injection

E. Splint external fixation

F. Acupuncture and physical therapy

202. An 11-year-old male with right hip pain and limited movement for six months, worsening over the past week, with low-grade fever, night sweats, and fatigue, shows right hip tenderness without swelling and joint stiffness. X-ray reveals slightly narrowed joint space and no significant bone destruction. The patient had a history of primary pulmonary tuberculosis. The main consideration is:

A. Right hip pyogenic arthritis

B. Ankylosing spondylitis with hip involvement

C. Transient synovitis of the hip

D. Right hip tuberculosis

E. Right hip osteoarthritis

F. Right hip traumatic arthritis

203. To further clarify the diagnosis for the same patient, the most valuable examination is:

A. Complete blood count and erythrocyte sedimentation rate (ESR)

B. CT scan

C. ECT (bone scintigraphy)

D. Arthroscopic synovial biopsy of the hip

E. Joint aspiration and smear examination

F. MRI

204. The early X-ray manifestations of hip tuberculosis primarily include:

A. Predominantly bone proliferation

B. Coexistence of bone proliferation and destruction

C. Predominantly bone resorption

D. Localized decalcification

E. Disappearance of joint space

F. Hip joint fusion

205. Hip tuberculosis often presents as:

A. Single joint involvement

B. Acute onset with rapid disease progression

C. Multiple joint involvement

D. Primarily involving small joints of the spine

E. Common in the elderly

F. Self-limiting

206. The following is not a commonly used physical examination method for diagnosing hip tuberculosis:

A. Hip hyperextension test

B. "4" sign test

C. Thomas test

D. Picking-up test

E. Hip rolling test

F. Straight leg raise test

207. The most common early form of hip tuberculosis is:

A. Simple synovial tuberculosis

B. Mixed-type tuberculosis

C. Simple bone tuberculosis

D. Total joint tuberculosis

E. Systemic toxic symptoms

F. None of the above

208. Late-stage pathological changes of hip tuberculosis exclude:

A. Cold abscess

B. Pathological dislocation

C. Narrowing of joint space

D. Joint space still present

E. Joint stiffness

F. Possible sinus tract formation

209. The key points for differentiating hip tuberculosis from transient synovitis of the hip are:

A. The latter is transient without low-grade fever, night sweats, etc.

B. The former is more common in adults

C. The latter is more common in adults

D. The latter shows bone destruction on X-ray

E. Difficult to differentiate

F. The latter is persistent

210. The preferred sites for the primary lesion in simple bone tuberculosis of the hip are:

A. Ligamentum teres of the femoral head

B. Edge of the acetabulum

C. Marginal area of the femoral head

D. Marginal area of the femoral head or iliac bone portion of the acetabulum

E. Central area of the femoral head

F. None of the above are correct
