# Supplementary material for: Integrating ChatGPT in Orthopedic Education for Medical Undergraduates: Randomized Controlled Trial
Source: J Med Internet Res. 2024 Aug 20;26:e57037. doi: 10.2196/57037 (PMC11372336; doi:10.2196/57037)
Supplement: Multimedia Appendix 4 [file jmir_v26i1e57037_app4.docx]

Multimedia Appendix 4.

|  | Control Group | ChatGPT Group |
| --- | --- | --- |
| Orthopedics course (Total 7 days) | ✓ | ✓ |
| Orthopedics practice test (Total 5 hours) | ✓ | ✓ |
| Review assistance with the Internet (7 days) | ✓ |  |
| Review assistance with the ChatGPT (7 days) |  | ✓ |
| Orthopedics exam | ✓ | ✓ |
| Examinations in other subjects | ✓ | ✓ |
